# A chemically contiguous hapten approach for a heroin–fentanyl vaccine

**DOI:** 10.3762/bjoc.15.100

**Published:** 2019-05-03

**Authors:** Yoshihiro Natori, Candy S Hwang, Lucy Lin, Lauren C Smith, Bin Zhou, Kim D Janda

**Affiliations:** 1 Departments of Chemistry, Immunology and Microbial Science, Skaggs Institute for Chemical Biology; The Scripps Research Institute, 10550 N Torrey Pines Rd, La Jolla, CA, 92037, USA; 2 Faculty of Pharmaceutical Sciences, Tohoku Medical and Pharmaceutical University, Komatsushima 4-4-1, Aoba-ku, Sendai, 981-8558, Japan; 3 Department of Chemistry, Southern Connecticut State University, 501 Crescent St, New Haven, CT, 06515, USA

**Keywords:** antinociception, fentanyl, hapten, heroin, vaccine

## Abstract

**Background:** Increased death due to the opioid epidemic in the United States has necessitated the development of new strategies to treat addiction. Monoclonal antibodies and antidrug vaccines provide a tool that both aids addiction management and reduces the potential for overdose. Dual drug vaccines formulated by successive conjugation or by mixture have certain drawbacks. The current study examines an approach for combatting the dangers of fentanyl-laced heroin, by using a hapten with one epitope that has domains for both fentanyl and heroin.

**Results:** We evaluated a series of nine vaccines developed from chemically contiguous haptens composed of both heroin- and fentanyl-like domains. Analysis of the results obtained by SPR and ELISA revealed trends in antibody affinity and titers for heroin and fentanyl based on epitope size and linker location. In antinociception studies, the best performing vaccines offered comparable protection against heroin as our benchmark heroin vaccine, but exhibited attenuated protection against fentanyl compared to our fentanyl vaccine.

**Conclusion:** After thorough investigation of this strategy, we have identified key considerations for the development of a chemically contiguous heroin–fentanyl vaccine. Importantly, this is the first report of such a strategy in the opioid–drug–vaccine field.

## Introduction

In 2016, the Centers for Disease Control (CDC) estimated that approximately 20.4% of the U.S. population (e.g., 50 million people) was suffering from chronic pain [1]. While using opioids to treat chronic pain is exceedingly effective in mitigating pain, the over prescription of opioids has substantially contributed to a rise in opioid abuse and a growing overdose crisis in the United States (CDC). In 2015, 63% of all drug overdose deaths involved an opioid drug (CDC). Although the overall rate of opioid prescription has declined in the past few years, heroin has become an increasingly common substitute for prescription opioids due to the accessibility and inexpensiveness of the illicit drug. This upsurge is also attributed to an increase in illicitly manufactured fentanyl, a powerful synthetic opioid commonly used for treatment of pain in cancer and postoperative patients (CDC). The recent emergence of fentanyl resulted in a 264% increase in synthetic opioid deaths within the U.S. from 2012 to 2015, due to the fact that users are often unaware of the presence of fentanyl contaminating the supply of heroin and cocaine to enhance their overall potencies. This trend is particularly disconcerting due to the observation that the combinatorial consumption of 10% fentanyl in heroin significantly enhances the risk of overdose by prolonging the detrimental effects of respiratory depression and brain hypoxia [2].

Respiratory depression is a common side effect of opioid overdose and can lead to serious complications, including death. The current treatment for overdose is naloxone (NARCAN^®^), a competitive antagonist of µ-opioid receptors. Other treatments for opioid substance abuse focus on mitigating cravings and managing long-term addiction. However, these treatments have an inherent risk for continued abuse. Combatting the complex nature of addiction requires a multifaceted strategy – several elements of the opioid crisis that need to be addressed include, but are not limited to, new non-addictive opioid drugs, innovative treatments to prevent relapse, and preventing and/or reversing overdose.

Monoclonal antibodies and antidrug vaccines are immunopharmacological treatments that address two of these highlighted needs: addiction management and a potential for reducing overdose. By chemically attaching a non-immunogenic drug-like molecule to an immunogenic carrier protein, a vaccine is formed that has the capacity to stimulate an immune response, generating antibodies capable of binding the targeted drug. Moreover, when a user consumes the drug, the antibodies circulating in the blood stream can block the “high” by forming a drug–antibody complex incapable of passing through the blood brain barrier. Ancillary, the vaccine’s immune response can alleviate the potential for overdose by reducing the total amount of drug exposed to µ-opioid receptors that induce respiratory depression. Indeed, anti-drug vaccines for individual conjugate vaccines of fentanyl and heroin have already exhibited protection in several animal models [3–8].

**Figure 1:**
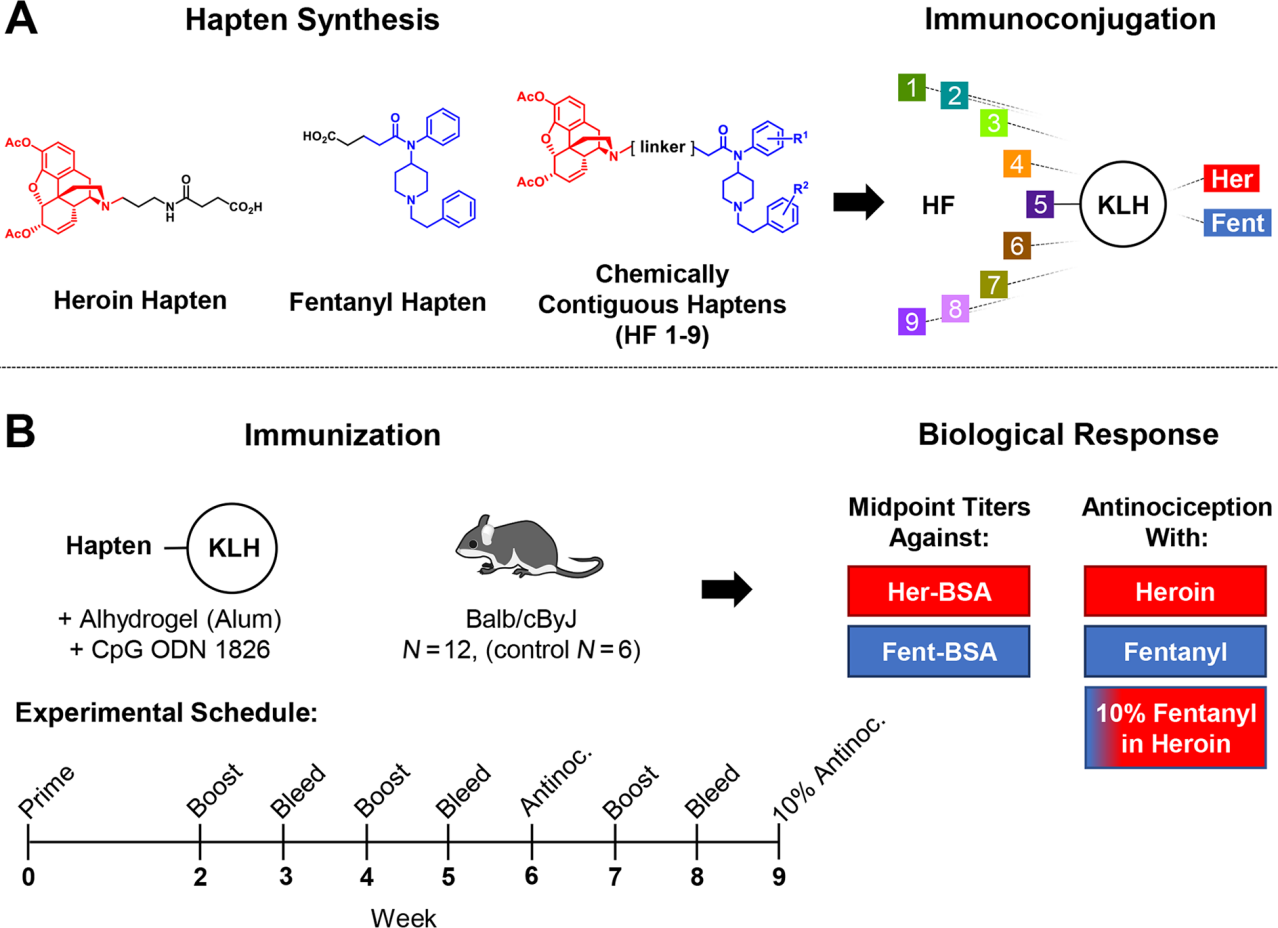
Graphical summary of chemically contiguous opioid vaccine approach. A) Illustration of chemically contiguous heroin-fentanyl concept and immunoconjugation strategy. B) Description of vaccine immunization schedule and metrics used to evaluate each vaccine’s efficacy.

Antidrug vaccines targeting multiple, structurally distinct species are not common and have utilized two tactics: 1) mixing two individual vaccines together (admixture vaccine) [9–10]; or 2) successive conjugation steps of one hapten and then the second to a carrier protein [11–12]. To specifically address the escalating incidence of fentanyl-contamination in heroin, we investigated the efficacy of an admixture of individual heroin and fentanyl conjugate vaccines against challenges of 10–20% fentanyl in heroin [13–14]. Gratifyingly, we found these admixture vaccines were able to produce antibodies with excellent affinity toward both drugs, as well as six other analogues of fentanyl. Moreover, the heroin–fentanyl admixture vaccine was able to blunt nociception incurred from either drug, however, we also realized the challenges of navigating through FDA approval of a combination vaccine. As such we pondered whether singular antigen presentation of both drugs in close proximity would still be able to entice a competent antibody–drug response and memory generation. To test this notion, we envisioned two drug haptens chemically joined to produce a colocalized epitope by chemical design. While success of such an approach would have undeniable challenges, all data gathered would at the very least provide insight into several immunochemical questions including: (1) the impact of multivalent hapten scaffolding; (2) the influence on increasing hapten density as compared to successive hapten-conjugations and (3) how concomitant hapten-presentation would impact immune response to each drug [15]. Herein, we describe the synthesis of nine unique chemically contiguous heroin–fentanyl haptens and their evaluation as conjugate vaccines to induce a polyclonal antibody response, as measured by ELISA, surface plasmon resonance (SPR), and in a behavioral assay (Figure 1).

## Results

**Figure 2:**
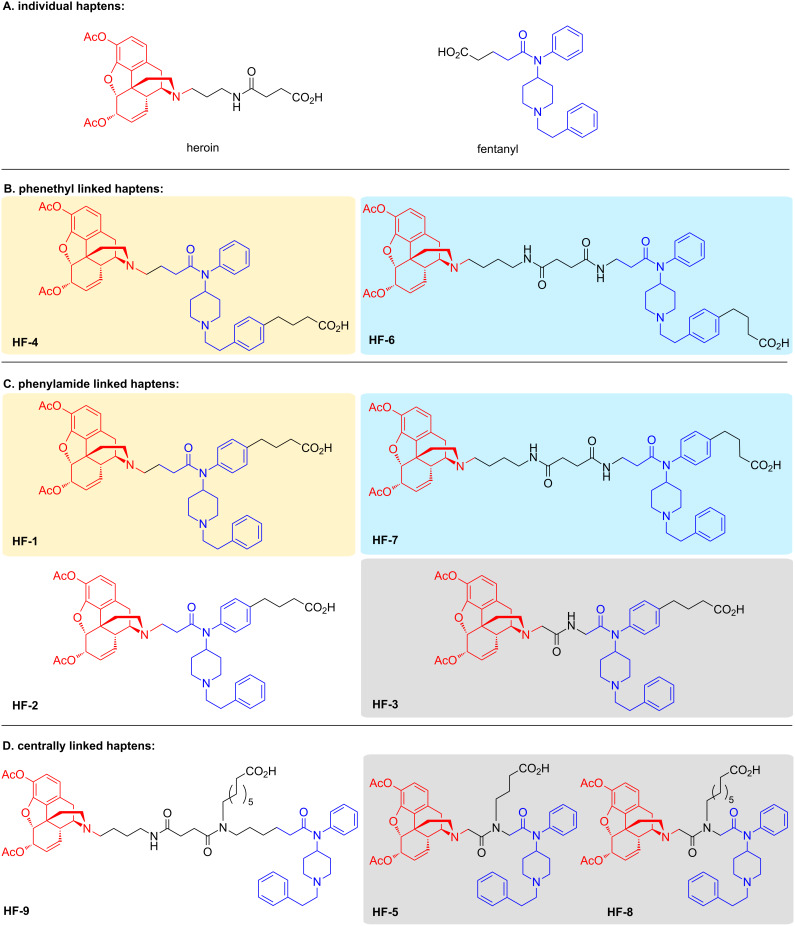
The chemically contiguous heroin–fentanyl haptens designed in this study. Grouping was based on the linker-carrier protein attachment. A) The individual drug haptens with an acid moiety for hapten-carrier protein attachment. B) Haptens to be linked to the carrier protein at the 4-position on the phenyethyl group. C) Drug haptens to be attached to the carrier protein *para* to the phenylamide. D) Haptens conjoined centrally between each drug hapten. Background color indicates haptens with structurally identical epitopes. Heroin and fentanyl molecules are highlighted in red and blue, respectively, in the hapten structures.

As a starting point for our exploration of chemically contiguous drug–hapten conjugates, we envisioned generating a series of structurally diverse heroin–fentanyl haptens with a focus on linker placement and bond distance between each drug’s chemical connections in order to probe the effect of the drug-epitope structure on eliciting an immune response. Figure 2 illustrates the nine haptens, as well as the original individual heroin and fentanyl haptens. To facilitate the preparation of a singular hapten that preserves the structure of each parent drug, the heroin- and fentanyl-like haptens were chemically joined by a series of what we hoped to be silent spacers. In sum, the feasibility of a singular heroin–fentanyl vaccine would be evaluated through five spatial presentations, differentiated in Figure 2 by background color.

The second consideration in our chemically contiguous hapten design was the location of the linker to the carrier protein. We chose three arrangements: 1) extending the linker off the phenylamide ring of fentanyl; 2) extending the linker from the phenethyl ring of fentanyl; or 3) anchoring the linker in between the two drug-like portions of our singular epitope. Again, Figure 2 provides a visual guide to our approach, with the heroin and fentanyl chemical-epitopes in each drug–hapten highlighted in red and blue, respectively. Based on this logic, we posit that the centrally linked haptens (Figure 2D) might be the most successful at producing an equivalent response to both drugs, as they are equidistant from the carrier protein, and should reduce structural masking of fentanyl.

In synthesizing the planned series of haptens, it was useful to consider each in terms of carrier-protein linker attachment as well as the size of the epitope as delineated in Figure 2. Based upon these restrictions, retention of a tertiary amine in the heroin domain was the primary constraint in constructing the epitope itself, while the protein carrier linker locations necessitated different syntheses of the fentanyl-like portion of the singular epitope. For example, the heroin-like domains of **HF-3**, **HF-5**, and **HF-8** could share an intermediate, while the fentanyl-like domains of **HF-1**, **HF-2**, **HF-3**, and **HF-7** (Figure 2) might be accessed from the same synthesis. Our other consideration was the general strategy for joining the two drug domains to form our chemically contiguous epitope. We envisioned synthesizing **HF-1**, **HF-2**, and **HF-4** by *N*-alkylation of the heroin domain via reductive amination. For the remaining drug–haptens, the use of repeated amide bonds naturally led us to employ EDC-mediated coupling to join the heroin and fentanyl domains. For the sake of brevity, detailed synthetic procedures, and structures of all intermediates can be found in Supporting Information File 1.

### Heroin domain syntheses

**Figure 3:**
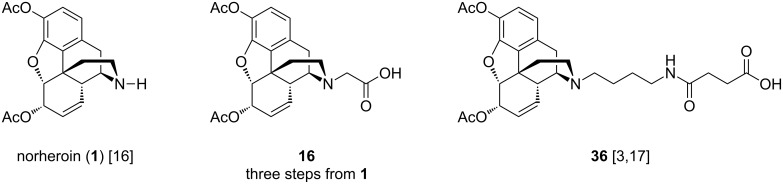
Heroin intermediates used to synthesize **HF-1** through **HF-9**.

All nine haptens in the series were constructed from one of the three heroin intermediates shown in Figure 3. Haptens to be prepared via reductive amination (**HF-1**, **HF-2**, **HF-4**, Figure 2 yellow background) required the secondary amine form, norheroin (**1**, Figure 3). Thus, norheroin (**1**) was synthesized according to literature procedure and purified by recrystallization [16]. Alkylation of **1** with *tert*-butyl bromoacetate and subsequent deprotection with trifluoroacetic acid yielded the *N*-glycine derivative of norheroin, **16**. This critical intermediate was used to construct **HF-3**, **HF-5**, and **HF-8** (Figure 2, gray background). Finally, the lengthy **HF-6**, **HF-7**, and **HF-9** haptens (Figure 2, blue background) were synthesized using intermediate **36**, prepared according to literature procedures [3,17].

### Fentanyl domain syntheses

**Scheme 1:**
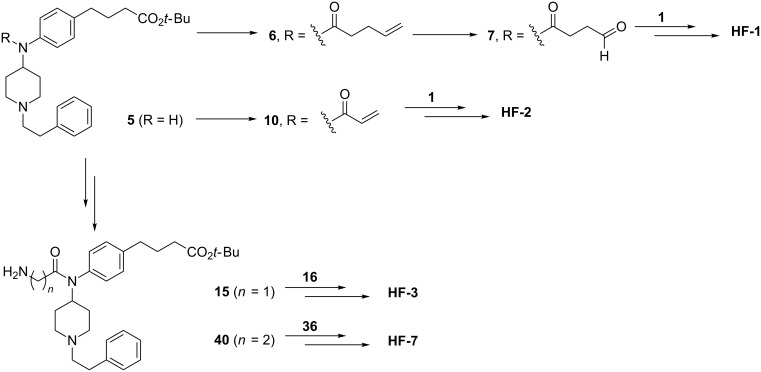
General outline of **HF-1**, **HF-2**, **HF-3**, **HF-7** synthesis from fentanyl intermediate **5** and heroin intermediates **1**, **16**, and **36**.

The scaffold variations in the fentanyl domain necessitated a more methodical approach to mapping out essential intermediates. We first considered the phenylamide-linked haptens **HF-1**, **HF-2**, **HF-3**, and **HF-7** (Figure 2B) and determined that they could be synthesized from intermediate **5** (Scheme 1).

**Scheme 2:**
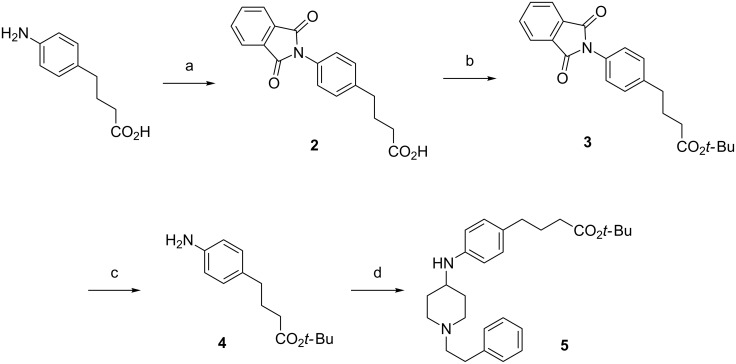
Synthesis of fentanyl intermediate **5**. Reaction conditions: a) phthalic anhydride, AcOH, reflux, 81%; b) *t*-BuOH, EDC, Et_3_N, DMAP, CH_2_Cl_2_, 0 °C to rt, 86%; c) H_2_NNH_2_, EtOH, 89%; d) 1-phenethylpiperidin-4-one, NaBH(OAC)_3_, AcOH, 0 °C to rt, 92%.

To prepare this intermediate, the *tert*-butyl ester protected derivative **4** of the commercially available 4-(4-aminophenyl)butanoic acid was accessed via protection and deprotection of the amine with a phthaloyl group (intermediates **2**, **3**). Reductive amination of **4** with commercially available phenethylpiperidin-4-one furnished intermediate **5** in good yield (92%, Scheme 2).

With intermediate **5** in hand, further elaboration was necessary to obtain the aldehyde required for the proposed reductive amination to access **HF-1**. Intermediate **5** was acylated with pentenoyl chloride to give alkene **6**, which was then oxidatively cleaved using potassium osmate(IV) hydrate followed by sodium periodate to obtain aldehyde **7** (Scheme 1) [18–19]. Buoyed by this success, we tried a similar approach to obtain the fentanyl-like domain aldehyde required for **HF-2**. To our surprise, oxidative cleavage both by ozonolysis and osmium tetroxide on the corresponding alkene resulted in a mixture of products. We opted to try the synthesis of **HF-2** by 1,4-addition, rather than reductive amination. Thus, intermediate **5** was acylated with acryloyl chloride to give the α,β-unsaturated intermediate **10** (Scheme 1).

Condensation with a *tert*-butyl-protected glycine residue to intermediate **5** yielded **15** (Scheme 1), which we used en route to **HF-3**. To reach **HF-7** in a similar manner, a homoglycine residue was condensed with **5** to give **40** (Scheme 1).

**Scheme 3:**
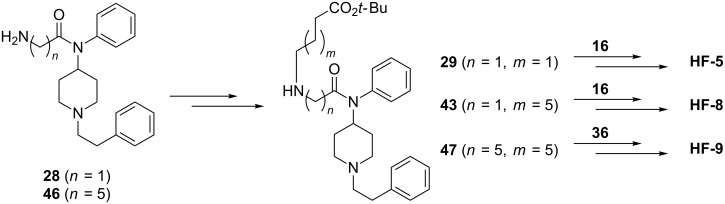
General outline of **HF-5**, **HF-8**, **HF-9** synthesis from fentanyl intermediates **28** and **46**, and heroin intermediates **16**, and **36**.

We then considered systematic preparation of the branched haptens **HF-5**, **HF-8**, and **HF-9** (Figure 2). It appeared that *N*-acylated fentanyl intermediate **28** could be used to access **HF-5** and **HF-8**, while the related intermediate **46** could be used for **HF-9** (Scheme 3). Both of these *N*-acylated fentanyl intermediates were prepared according to published procedures [20–21]. Coupling **28** to either a four- or eight-carbon linker yielded the desired intermediates **29** and **43**, for **HF-5** and **HF-8**, respectively (Scheme 3). Alkylation of **46** with an eight-carbon linker gave intermediate **47**, procuring the fentanyl-like domain of **HF-9** (Scheme 3).

**Scheme 4:**
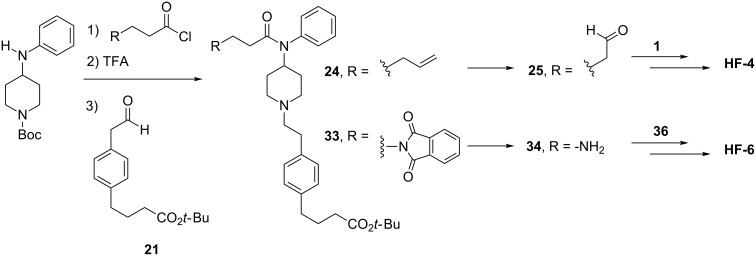
Parallel synthesis of fentanyl domains **25** and **34**, for **HF-4** and **HF-6**, respectively.

As with **HF-1** and **HF-2**, the goal for the synthesis of the **HF-4** fentanyl-like domain was to obtain an aldehyde that could then be used in a reductive amination. Beginning with the commercially available 4-(4-bromophenyl)butanoic acid, an alkene was installed with a Suzuki reaction, which in turn underwent ozonolysis to give **21**, shown in Scheme 4. In parallel, a commercially available piperidine was acylated and Boc-deprotected to yield the amine that was then reductively aminated with **21** to give **24**, completing the fentanyl-like domain (Scheme 4). Oxidative cleavage of **24** yielded the aldehyde **25**, ready for reductive amination with the heroin domain intermediate.

While **HF-6** was designed with the same linker as **HF-4**, its lengthy epitope actually required a different, but parallel synthetic strategy. The same commercially available piperidine used to obtain **24** was instead acylated with phthaloyl-protected aminopropanoyl chloride, deprotected, and reductively aminated with **21** to give **33** (Scheme 4). Intermediate **33** was then deprotected to give **34**, leading to facile EDC-mediated coupling with **36** to give **HF-6** (Scheme 4).

### Heroin and fentanyl epitope coupling

**Scheme 5:**
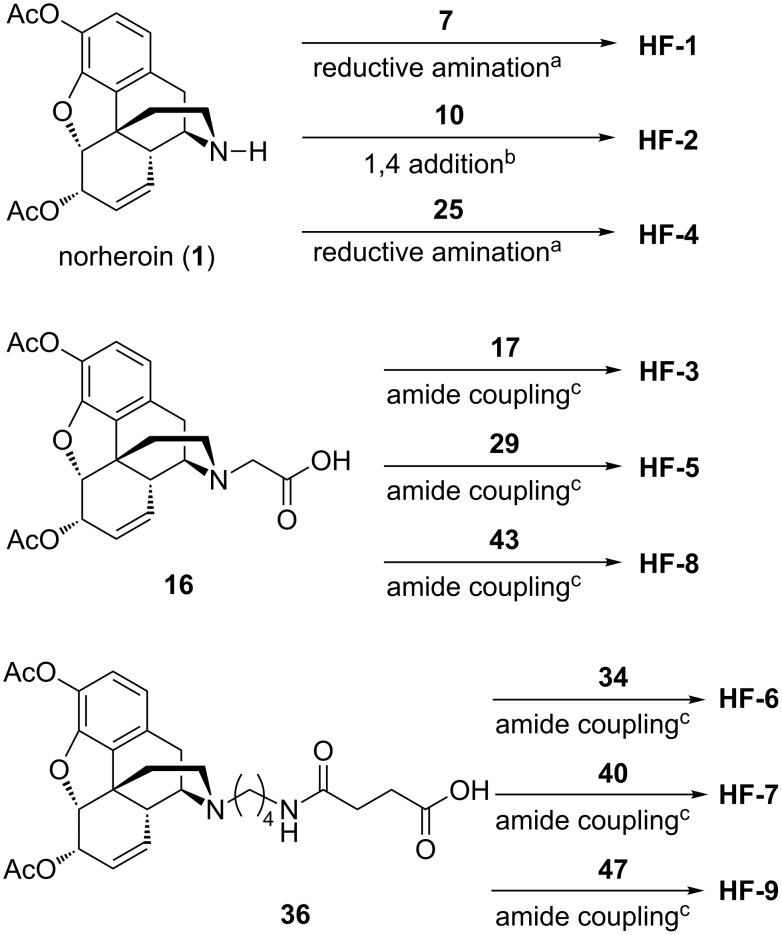
General strategy and coupling partners for the chemically contiguous series. ^a^General conditions for reductive amination of **1** with indicated aldehyde: NaBH(OAc)_3_ (2.5 equiv), AcOH, 0 °C → rt. ^b^Conditions for 1,4-addition of **1** to **10**: Zn(OTf)_2_ (0.2 equiv), CH_3_CN, rt. ^c^General conditions for amide coupling: EDC·HCl (1 equiv), Et_3_N (3 equiv), DMAP (0.1 equiv), CH_2_Cl_2_, 0 °C → rt.

With the various drug-like domains prepared, we set to work forging them into a single epitope by employing our proposed strategies. **HF-1** and **HF-4** were obtained from reductive amination of heroin intermediate **1** to key fentanyl intermediates **7** and **25**, respectively (Scheme 5). In synthesizing **HF-2**, the aforementioned failure to obtain the necessary aldehyde for reductive amination necessitated a strategic modification to 1,4-addition. After screening acid catalysts (Figure S1, Supporting Information File 2), successful 1,4-addition of heroin intermediate **1** to fentanyl intermediate **10** yielded **HF-2** (Scheme 5).

Heroin intermediate **16** gave access to the phenylamide-linked haptens **HF-3**, **HF-5**, **HF-8** via EDC-mediated coupling to their respective fentanyl-like domains. Similarly, **HF-6**, **HF-7**, **HF-9** were obtained by coupling heroin domain **36** to **34**, **40**, or **47**, respectively (Scheme 5). All haptens were synthesized with the carrier protein linker protected as a *tert*-butyl ester. Deprotection in the presence of trifluoracetic acid unmasked the carboxylic acids, allowing direct coupling of haptens to the carrier protein.

### Hapten conjugation reaction with carrier proteins BSA and KLH

Test reactions on submilligram scale (0.1 mg hapten:0.1 mg BSA, 1 mg/mL protein) were performed with activated **HF-1** and BSA, resulting in a moderate number of haptens coating the protein’s surface (i.e., 10.0 copies). However, increasing the conjugation to a 1 mg scale caused the protein to precipitate immediately upon addition of **HF-1** in 9:1 DMF/H_2_O solution, eliminating the possibility of conjugation. To mitigate this precipitation problem, glycerol was added to the soluble KLH or BSA solution (Figures S2–S7, Supporting Information File 2). Concerned by the possibility that the alcohols on glycerol would outcompete amidation, we tested several concentrations of glycerol in the range of 10–50% (w/w). Based on MALDI–TOF data and as expected, the conjugation with the least amount of glycerol had the highest number of hapten copies (i.e., 11.1 copies). Addition of 50% w/w glycerol reduced the hapten density by one-half (i.e., 5.2 copies). After dialysis, the solutions remained translucent, but the protein conjugates did not crash out of the solution. As a reference, copy number for the individual heroin and fentanyl haptens were 7.3 and 12.4, respectively.

Another trend observed for the chemically contiguous haptens was the increased amount of time needed to activate the carboxylic acids. Typically, fentanyl and heroin haptens reach optimal threshold activation (above 80%) within thirty minutes to several hours. However, these haptens required a minimum of four to twelve hours for at least 50% activation. In addition, bulkier haptens, including **HF-4**, had solubility issues upon addition to carrier protein and therefore needed higher glycerol content to ameliorate protein precipitation and conjugation issues. The linker placement at the phenethyl position greatly improved conjugation compared to the other phenylamide group; **HF-4** had a hapten density of 21.1, drastically higher than **HF-2** (hapten density: 12.6) and **HF-3** (hapten density: 8.3). Gratifyingly, we observed copy numbers on BSA for every synthesized hapten. Immunoconjugates were then prepared with keyhole limpet hemocyanin (KLH) for murine immunization studies.

### Vaccination schedule, SPR and nociception analysis

The nine haptens were formulated with Alhydrogel (alum) and murine CpG ODN as previously described [13,17]. Briefly, vaccines were formulated with 25% (v/v) glycerol, 50 µg of CpG/dose per mouse and stored at −80 °C. On the day of vaccination, 0.8 mg of alum/dose per mouse was added and mixed for at least thirty minutes after thawing.

**Figure 4:**
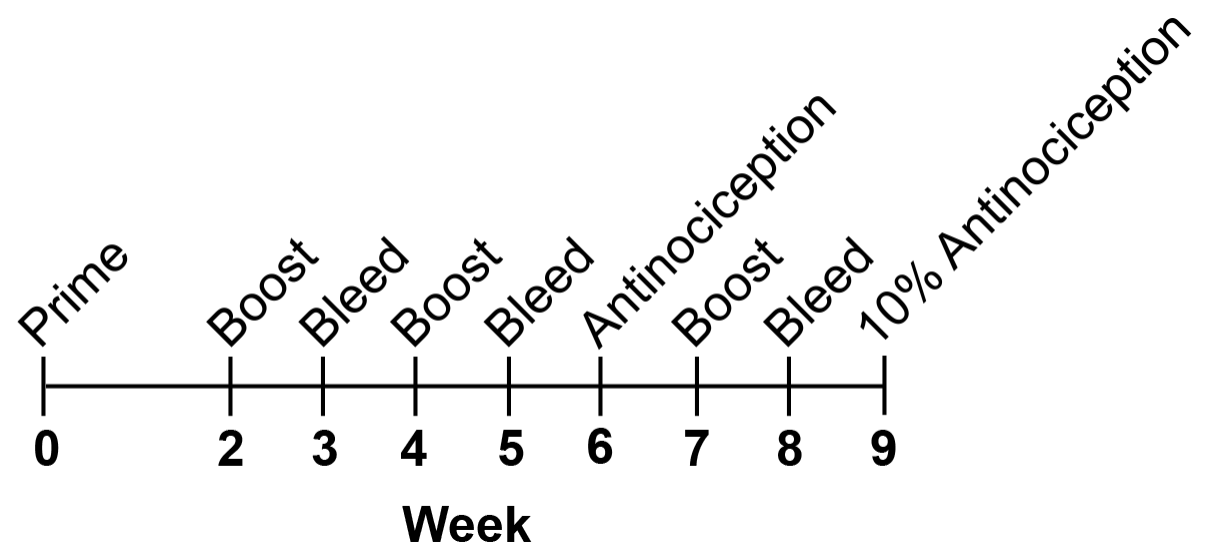
Vaccination, titer assessment, and bleed schedule.

Female BALB/cByJ mice were immunized and bled using the protocol detailed in our previous study, except this study incorporated an extra boost at week 7, bleed at week 8, and antinociception at week 9 [13]. The vaccination schedule is shown in Figure 4. Mice were bled at weeks 3, 5, and 8 using submandibular puncture with a single-use lancet to collect 100–500 µL whole blood. All sera were tested against both Her-BSA and Fent-BSA antigens, and sera from each chemically contiguous vaccine group were tested against their own antigen. For heroin analysis, 6-acetylmorphine (6-AM) was used as the primary analyte in surface plasmon resonance (SPR) analysis as it has a longer half-life than heroin at pH 7.4.

**Figure 5:**
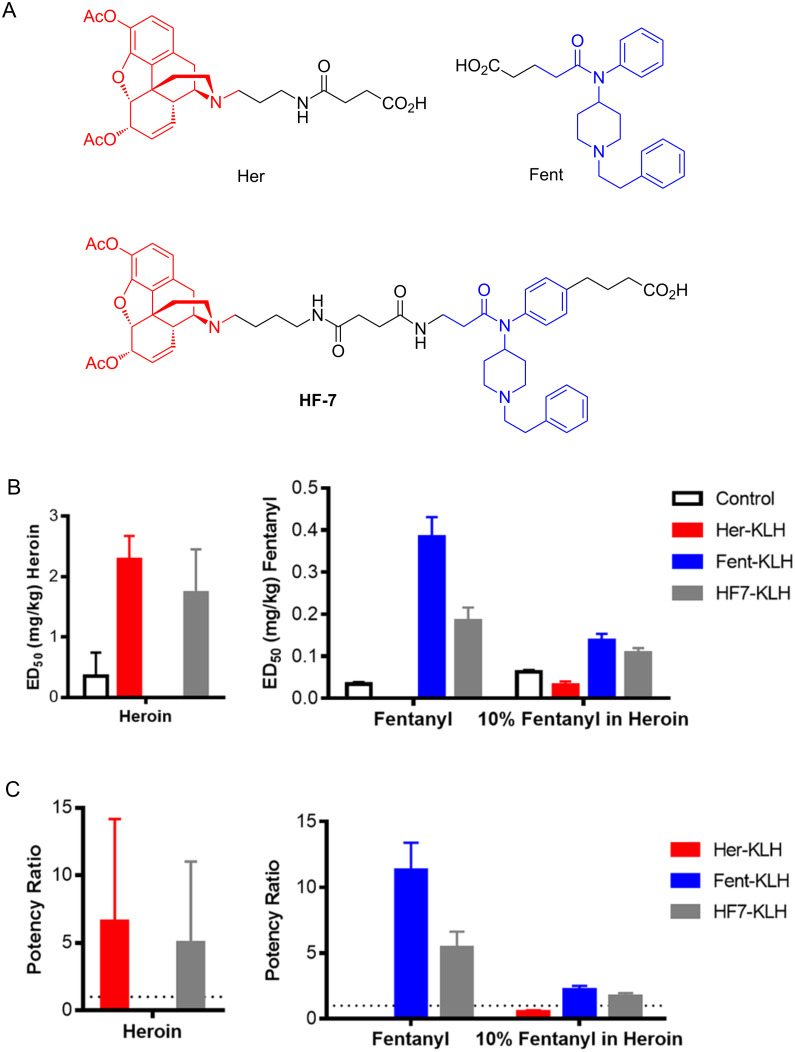
Summary of behavioral data for most promising chemically contiguous vaccine **HF-7**, compared to singular hapten-vaccines and controls. A) Structures of haptens; B) ED_50_ values for heroin and fentanyl; C) potency values. Bars represent means + SEM.

To begin epitope interpretation, we ran the haptens in numerical order, and observed midpoint titers, which were highest for all groups at week 5. In contrast to titer, IC_50_ affinity continued to improve up to week 8. Individual conjugate vaccines had the highest level of midpoint titers of all groups as well as the best affinity values (Figures S8–S11, Supporting Information File 2). Of the chemically contiguous vaccines only a handful of them elicited single-digit nanomolar affinity to either 6-AM or fentanyl. Interestingly, the centrally linked haptens (**HF-5, HF-8**, and **HF-9**) had affinity only toward one of the two drugs, not both as initially anticipated. Also of note was that, while **HF-4** and **HF-6** had excellent affinity toward 6-AM, this did not translate to antibody-fentanyl affinity, which was 40 and 70-fold poorer. As expected this poor affinity to fentanyl was recapitulated as a nonstarter in behavior as seen in the tail flick antinociception assay (Figure S12, Supporting Information File 2). Finally, from all nine vaccine designs, HF-7 provided most compelling antinociception efficacy in terms of the 10% fentanyl in heroin challenge (Figure 5).

## Discussion

Unfortunately, analyzing data by chemical epitope, i.e., each drug, did not reveal any foretelling trends for immune response against said drug. With that said, it seems that the chemical linkage providing continuity between the heroin and fentanyl-like portions of the hapten had less impact on vaccine performance. Rather, the regiochemical placement of the hapten-linker when coupled to the carrier protein seemed to provide a greater influence on the immune response (grouping in Figure 2 and Figure S13, Supporting Information File 2).

We were taken aback to observe that the centrally-linked vaccines comprised of **HF-5**, **HF-8**, **HF-9**, Figure 2D performed so poorly in ELISA analysis (Figure S9, Supporting Information File 2), given that they most closely mirror the individual conjugate hapten vaccines. It is possible that the branched structure is not ideal for MHC-presentation or processing, so regardless of affinity for 6-AM and/or fentanyl, these vaccines were not efficacious as too little antibody was produced. Indeed, in the tail flick antinociceptive experiment against 10% fentanyl in heroin, mice vaccinated with these haptens were not significantly better than the control group.

**Figure 6:**
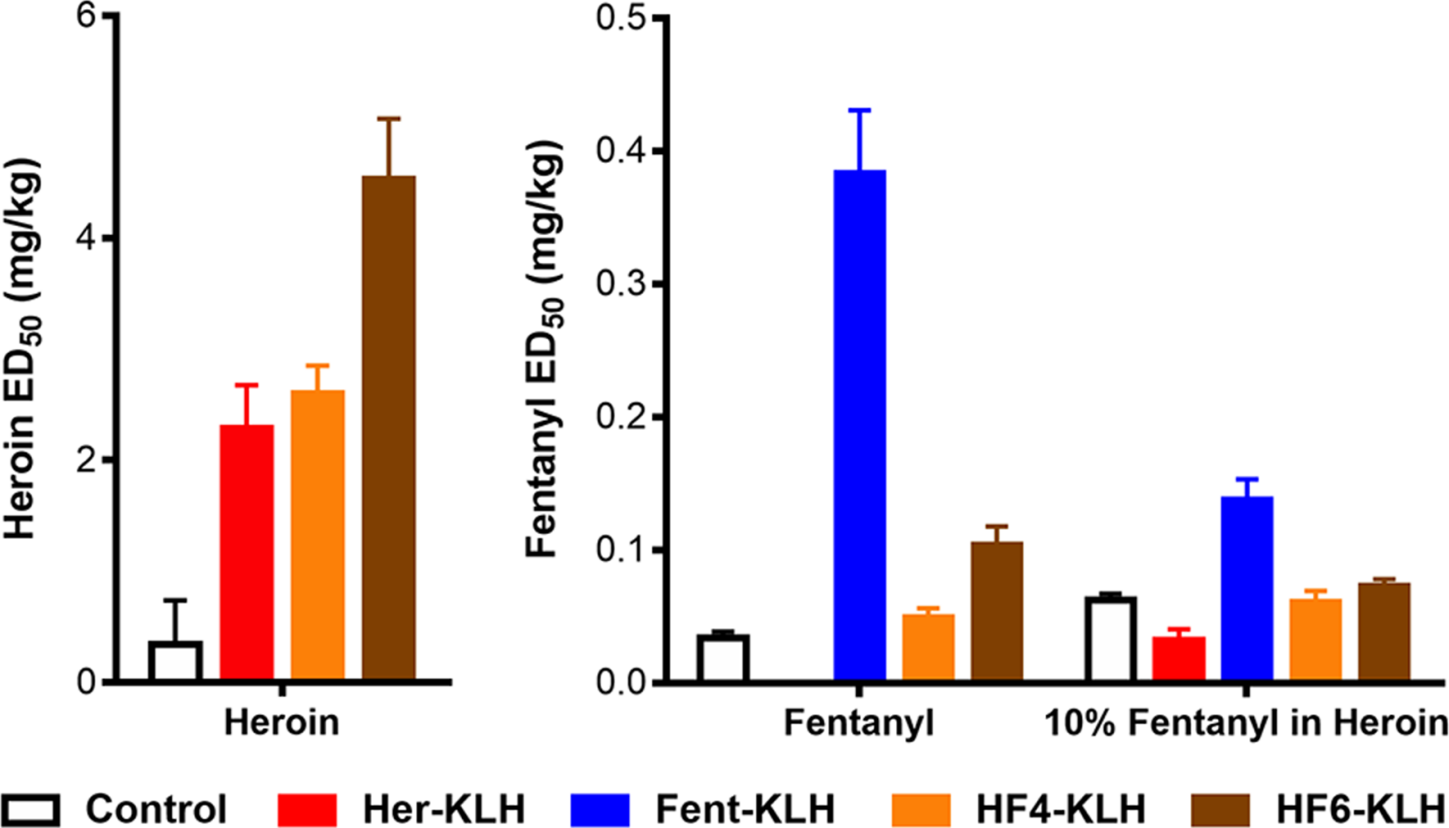
Summary of behavioral data for phenethyl-linked haptens **HF-4** and **HF-6**. Bars represent mean ± SEM.

The phenethyl-linked hapten vaccines (**HF-4**, **HF-6**, Figure 2) performed similarly or better than the Her-KLH vaccine as seen in the antinociception assay when mice were challenged with heroin, but performed poorly against fentanyl challenged mice (Figure 6). In line with this result is the SPR data, which by week 8 showed that **HF-4** and **HF-6** displayed the best IC_50_s for 6-AM (Figure S8, Supporting Information File 2). The phenethyl-linked conjugate-vaccines also produced the highest titers against Her-BSA and the lowest against Fent-BSA (Figure S9, Supporting Information File 2). We hypothesize that these phenethyl-linked haptens mask the fentanyl-like domain epitope, resulting in poor titers. Poor titers against fentanyl as expected translated to little protection against 10% fentanyl in heroin.

It is difficult to extrapolate any definitive conclusions regarding phenylamide-linked vaccines, (Figure 2C) other than that their ability to enhance heroin’s nociception effects was modest, and only worked marginally better against fentanyl. That being said, overall it appears that the **HF-7** vaccine had the most “balanced” protection against heroin and fentanyl. Possibly, fentanyl’s epitope domain is better displayed in **HF-7**, compared to **HF-6**, which is identical except for protein carrier attachment. In the antinociceptive studies, the **HF-7** vaccine provided protection against heroin comparable to Her-KLH vaccine (Figure 5B), but did relatively poorly against a fentanyl challenge compared to the Fent-KLH vaccine. Although the **HF-7** vaccine demonstrated limited protection against fentanyl challenge, its superior immune response against heroin permitted it to be as efficacious in nociception examination as the Fent-KLH vaccine when examined in the 10% fentanyl in heroin.

**Figure 7:**
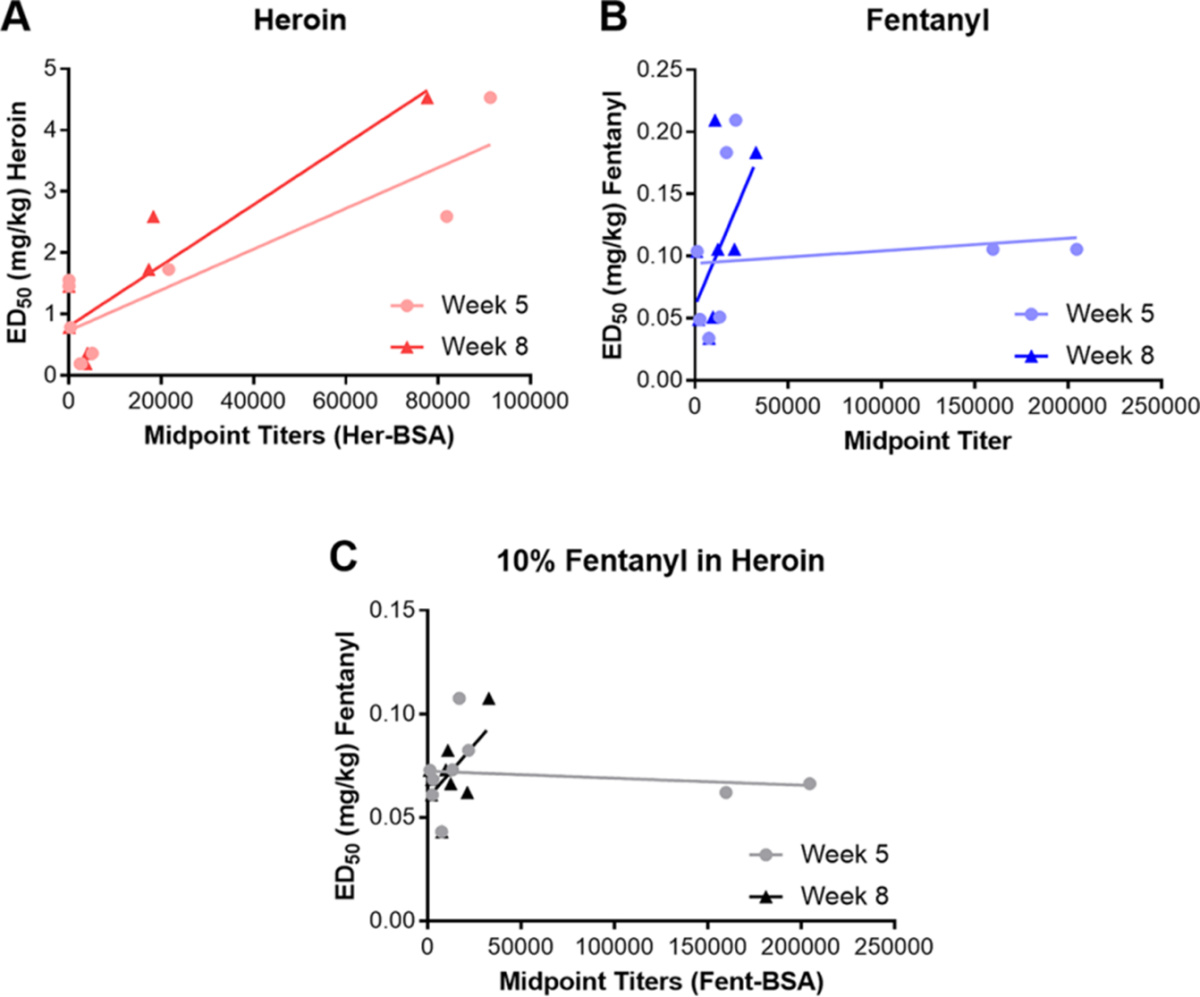
Correlation plots of dual hapten vaccines comparing week 5 and 8 ELISA midpoint titers to ED_50_ values. Linear regression analysis was performed and R^2^ values for week 8 are A) heroin week 8 R^2^ = 0.7803; B) fentanyl week 8 R^2^ = 0.3459; C) 10% fentanyl in heroin week 8 R^2^ = 0.3715.

We also sought to determine if there was any predictive correlation between the in vitro SPR and ELISA results, and the behavioral results. To this end, nociception ED_50_ values obtained from each vaccine challenged with either heroin, fentanyl, or 10% fentanyl in heroin were plotted against either IC_50_ or midpoint titers obtained for that analyte. Linear regression analysis was used to determine correlation. Initially we plotted ED_50_ versus SPR IC_50_s, but we found no correlation. Similarly, no correlation was found between IC_50_ and midpoint titers. However, when we plotted ED_50_ against midpoint titers (Figure 7), we saw a positive correlation in the heroin group and this correlation improved by week 8. Because there was no relationship between antibody affinity for 6-AM and in vivo efficacy, it is tempting to conclude that the quantity of antibody is more important for protection against a heroin challenge. However, the interplay between antibody concentration and affinity for this series is difficult to delineate. That being said, the two vaccines most successful against heroin challenge, **HF-4** and **HF-6**, had the highest titers for Her-BSA and displayed the greatest affinity for 6-AM. Interestingly, it appears that when compared to fentanyl or 10% fentanyl in heroin groups, the week 5 midpoint titers have no correlation with efficacy (R^2^ < 0.03). Week 8 titers show a slight correlation, although it is was not found to be significant. Moreover, nearly all of the vaccines produced very low titers and affinity to fentanyl compared to fentanyl-KLH vaccine (Figures S8 and S9, Supporting Information File 2).

## Conclusion

Our chemically contiguous hapten campaign was initiated to investigate the possibility of taking two structurally different opioids, heroin and fentanyl, and combining them into a singular, unique opioid hapten that, when formulated with an adjuvant, would present us with a vaccine capable of generating antibodies to both drugs. Based on this idea, a series of nine vaccines were prepared that enabled us to detect subtle influences on antibody response to each drug. In sum, we found little traction with each hapten’s vaccine efficacy from the standpoint of the chemical connection between the two drugs. Interestingly, immune response seemed to be more influenced by linker-carrier protein regiochemical placement. Although none of the nine haptens outperformed the individual conjugate vaccines against their assigned drug, the **HF-7** conjugate vaccine did show promising results as its heroin response was on par with Her-hapten immune response and it provided some efficacy against the fentanyl in heroin mixture. As linker-carrier protein regiochemical placement seemed more controlling for a balanced immune response against both drugs, it will be of interest to explore linker-carrier-protein display off the 3 or 6-position of heroin, thereby more prominently displaying the fentanyl moiety. These studies will be reported in due course.

## Supporting Information

Supporting information features SPR and ELISA experimental results, and additional experimental data. It also includes detailed procedures for all reported intermediates and final compounds, as well as their ^1^H NMR, ^13^C NMR, and high-resolution mass-spectrometry signals.

File 1 Supporting experimental results.

File 2 Experimental procedures for compounds **1–53**.

File 3 NMR spectra of compounds **1–53**.
