# Supplementary material for: A chemically contiguous hapten approach for a heroin–fentanyl vaccine
Source: Beilstein J Org Chem. 2019 May 3;15:1020–31. doi: 10.3762/bjoc.15.100 (PMC6541359; doi:10.3762/bjoc.15.100)

BEILSTEIN JOURNAL OF ORGANIC CHEMISTRY

Supporting Information

for

A chemically contiguous hapten approach for a heroin–fentanyl vaccine

Yoshihiro Natori, Candy S. Hwang, Lucy Lin, Lauren C. Smith, Bin Zhou and Kim D. Janda

Beilstein J. Org. Chem. **2019,** *15,* 1020–1031. doi:10.3762/bjoc.15.100

Supporting experimental results

License and Terms: This is a supporting information file under the terms of the Creative Commons Attribution License (http://creativecommons.org/licenses/by/4.0). Please note that the reuse, redistribution and reproduction in particular requires that the authors and source are credited.
The license is subject to the *Beilstein Journal of Organic Chemistry* terms and conditions: (https://www.beilstein-journals.org/bjoc)

Figure S1. Catalyst screening for synthesis of **11** S2
Figure S2. MALDI-TOF MS spectrum of HF1-BSA S3
Figure S3. MALDI-TOF MS spectrum of HF1-BSA, DMF reduced S4
Figure S4. MALDI-TOF MS spectrum of HF1-BSA, 10% glycerol S5
Figure S5. MALDI-TOF MS spectrum of HF1-BSA, 20% glycerol S6
Figure S6. MALDI-TOF MS spectrum of HF1-BSA, 25% glycerol S7
Figure S7. MALDI-TOF MS spectrum of HF1-BSA, 50% glycerol S8
Figure S8. IC_{50} values of vaccines from SPR S9
Figure S9. ELISA midpoint titer values for vaccination groups S10
Figure S10. Fent-BSA ELISA midpoint titers arranged by linker type. S11
Figure S11. Her-BSA ELISA midpoint titers arranged by linker type. S12
Figure S12. Summary of tail-flick antinociception data S13
Figure S13. Antinociception data clustered according to linker type S14

Figure S1) Catalyst screening for synthesis of 11

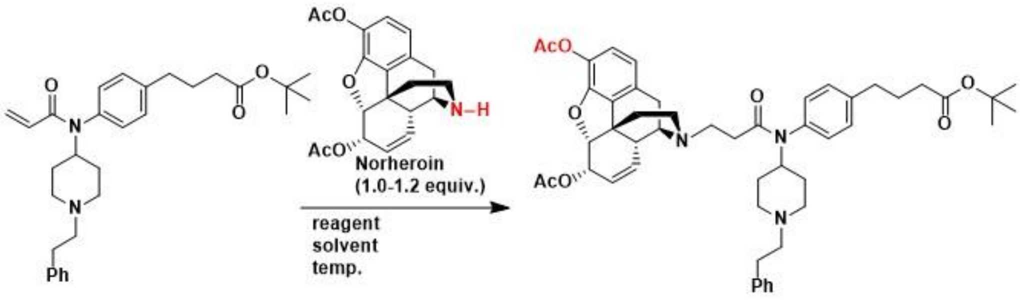

entry	reagent	solvent	temp.	results
1	DBU (1 eq.)	CH_3CN	rt to 50 °C	partial deprotection of Ac group
2	$Zn(OTf)_2$ (20 mol%)	CH_3CN	rt	20%
3	$Bi(OTf)_3$ (20 mol%)	CH_3CN	rt	not detected
4	Tf_2NH (20 mol%)	CH_3CN	rt	not detected (SM decomp.)
5	Tf_2NH (20 mol%)	CH_2Cl_2	rt	not detected (SM decomp.)
6	$Cu(OTf)_2$ (20 mol%)	CH_3CN	rt	not detected
7	$Pd(CH_3CN)_2Cl_2$ (20 mol%)	CH_3CN	rt	not detected
8	$MnCl_2\cdot4H_2O$ (20 mol%)	H_2O/MeOH (1/1)	rt	not detected

Figure S2) MALDI-TOF MS spectrum of HF1-BSA

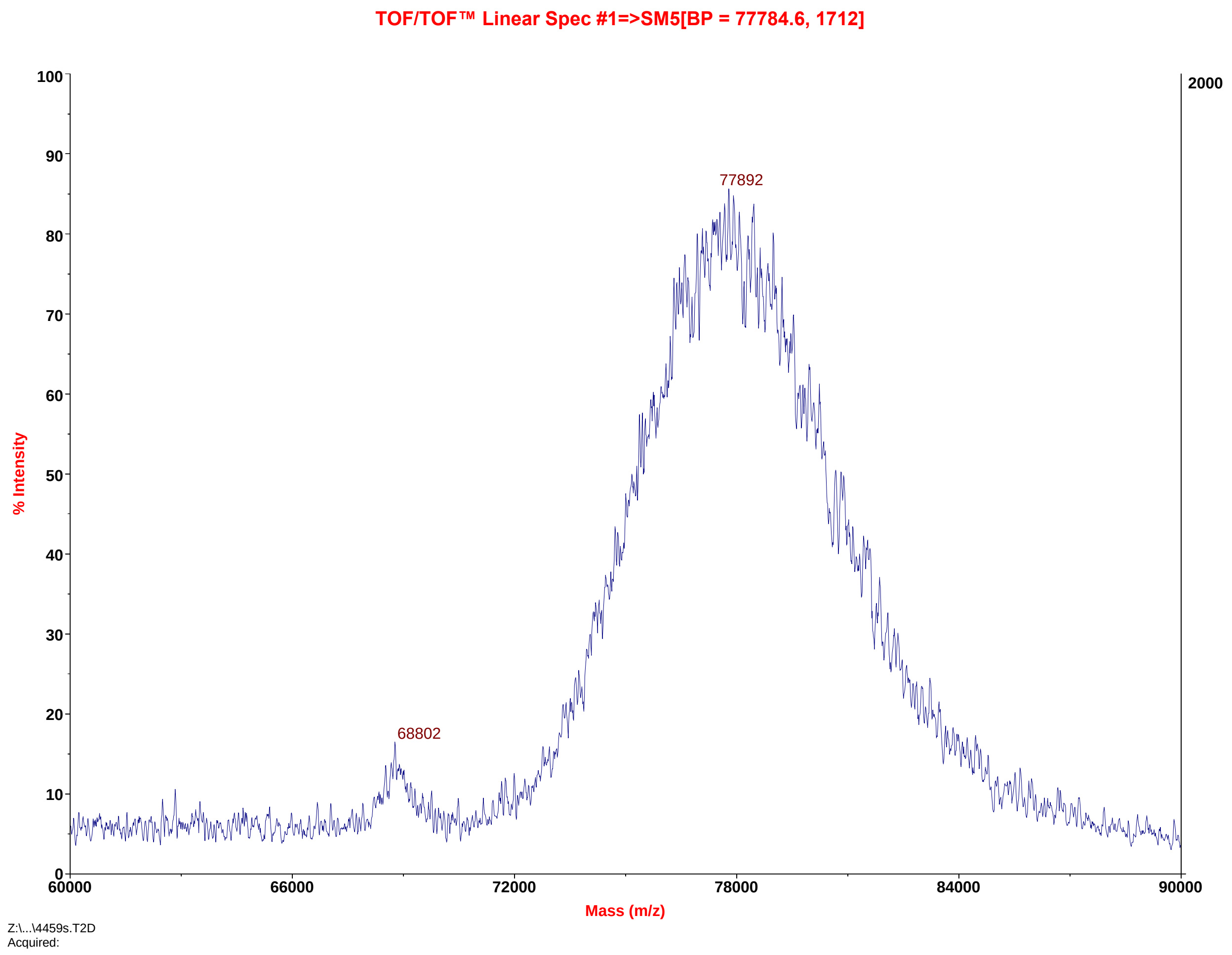

Figure S3) MALDI-TOF MS spectrum of HF1-BSA, DMF reduced

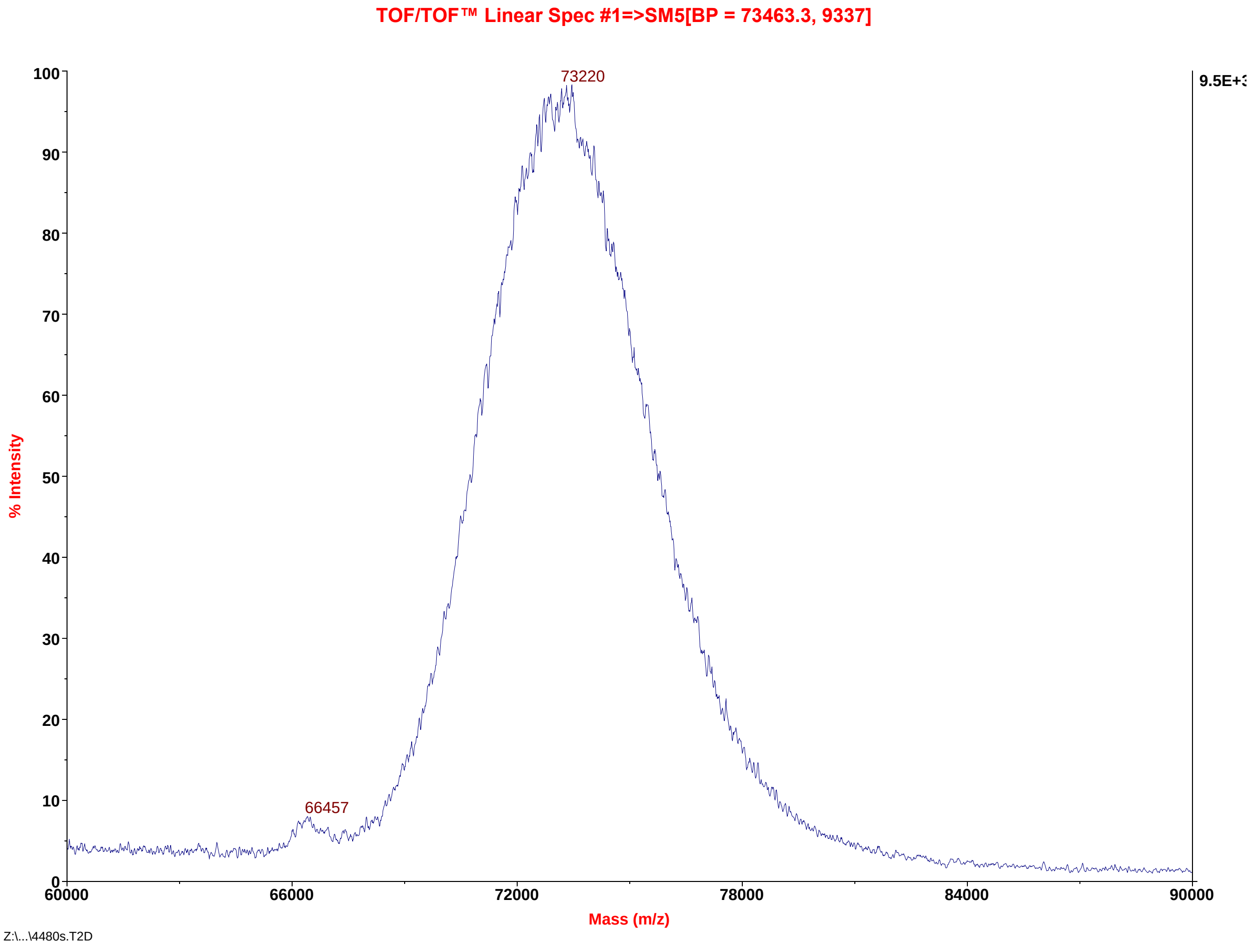

Z:\...\4480s.T2D
Acquired:

Figure S4) MALDI-TOF MS spectrum of HF1-BSA, 10% glycerol

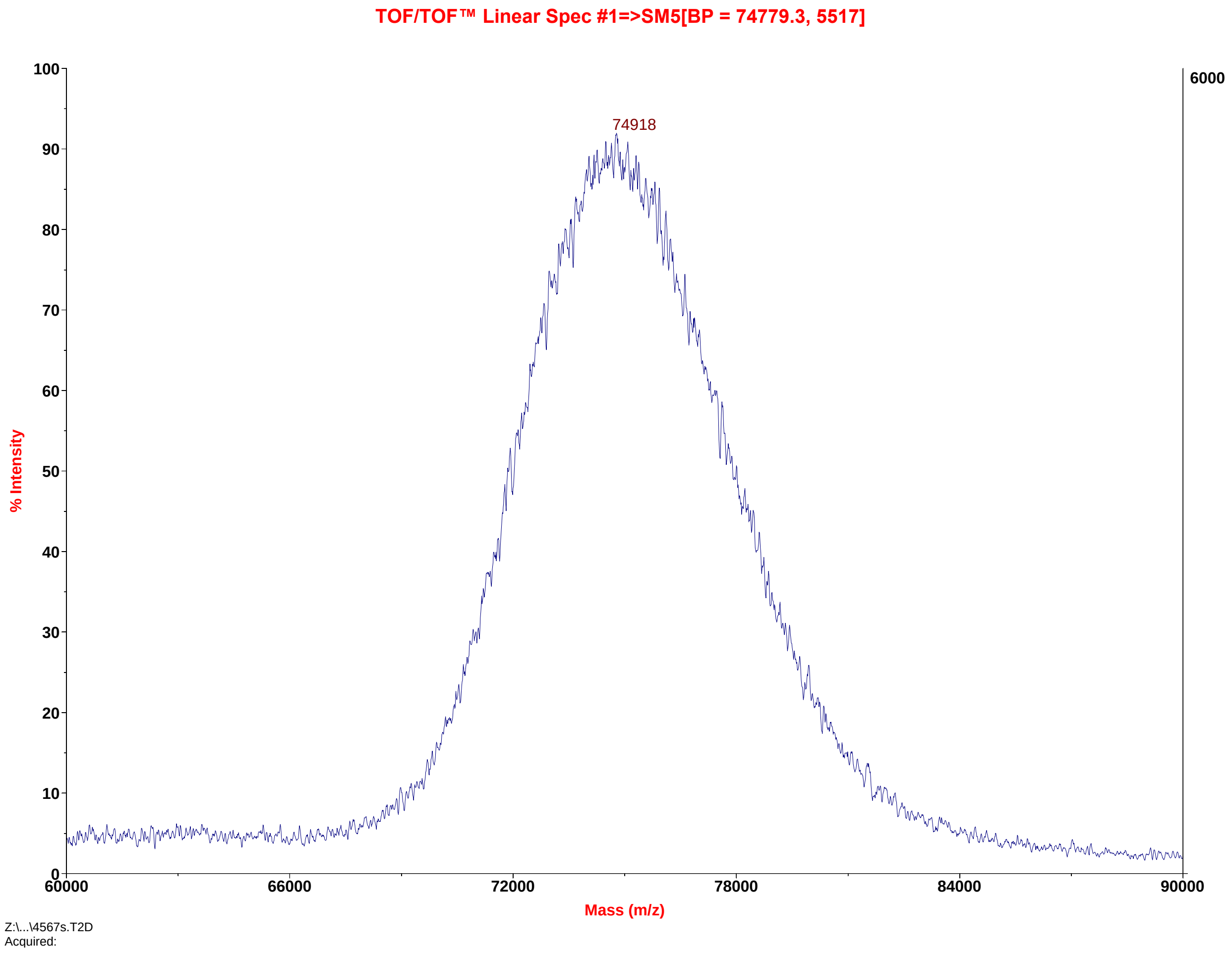

Figure S5) MALDI-TOF MS spectrum of HF1-BSA, 20% glycerol

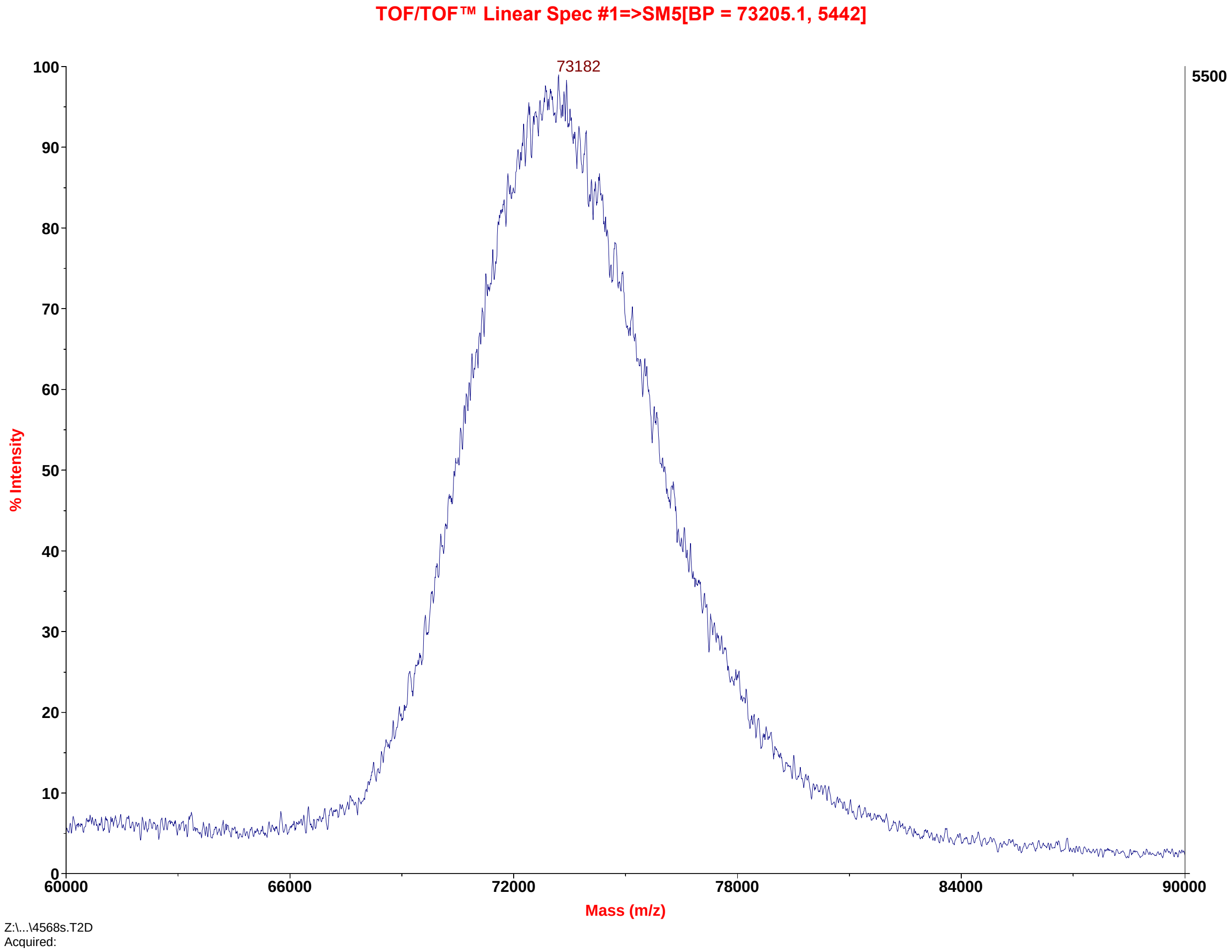

Figure S6) MALDI-TOF MS spectrum of HF1-BSA, 25% glycerol

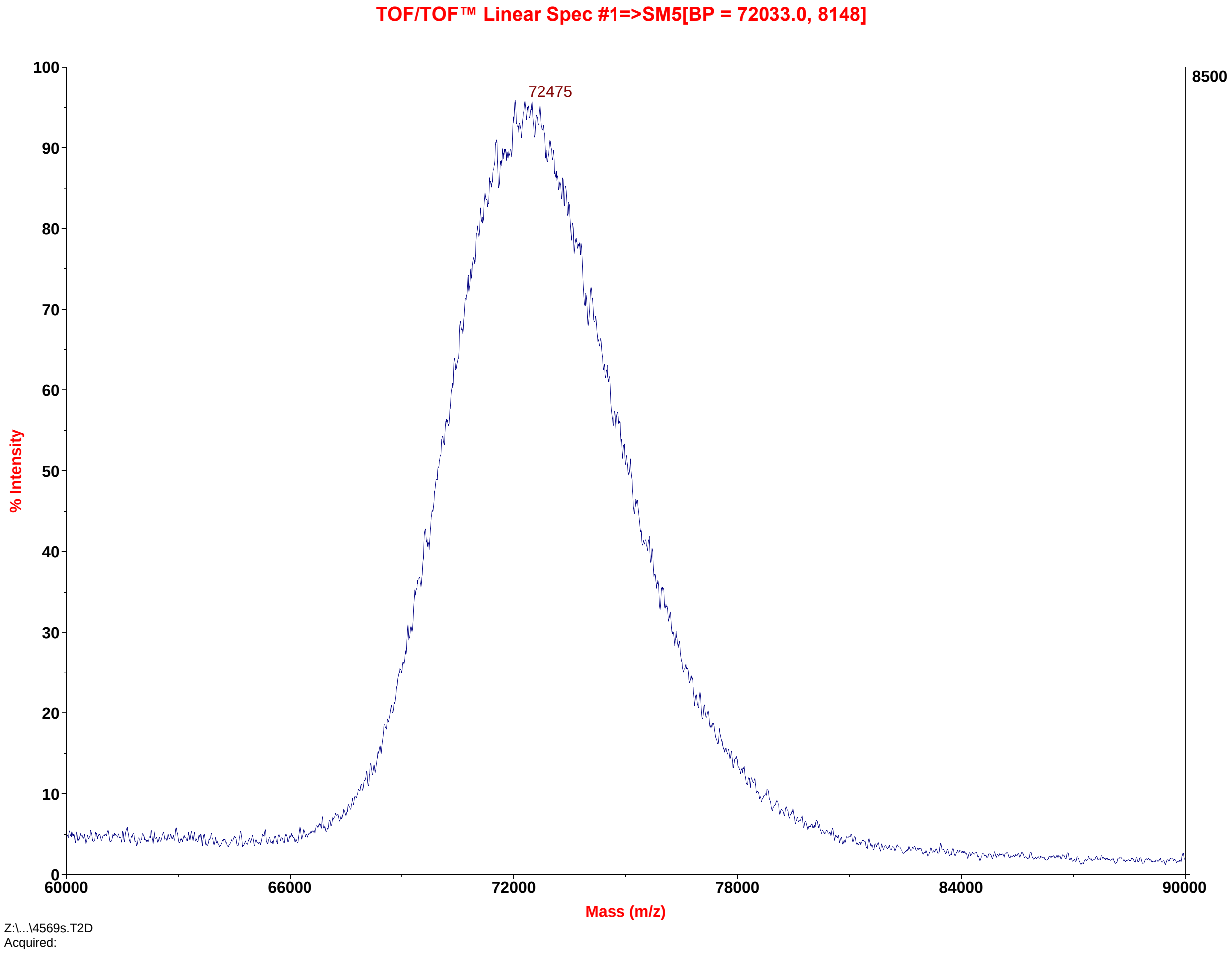

Figure S7) MALDI-TOF MS spectrum of HF1-BSA, 50% glycerol

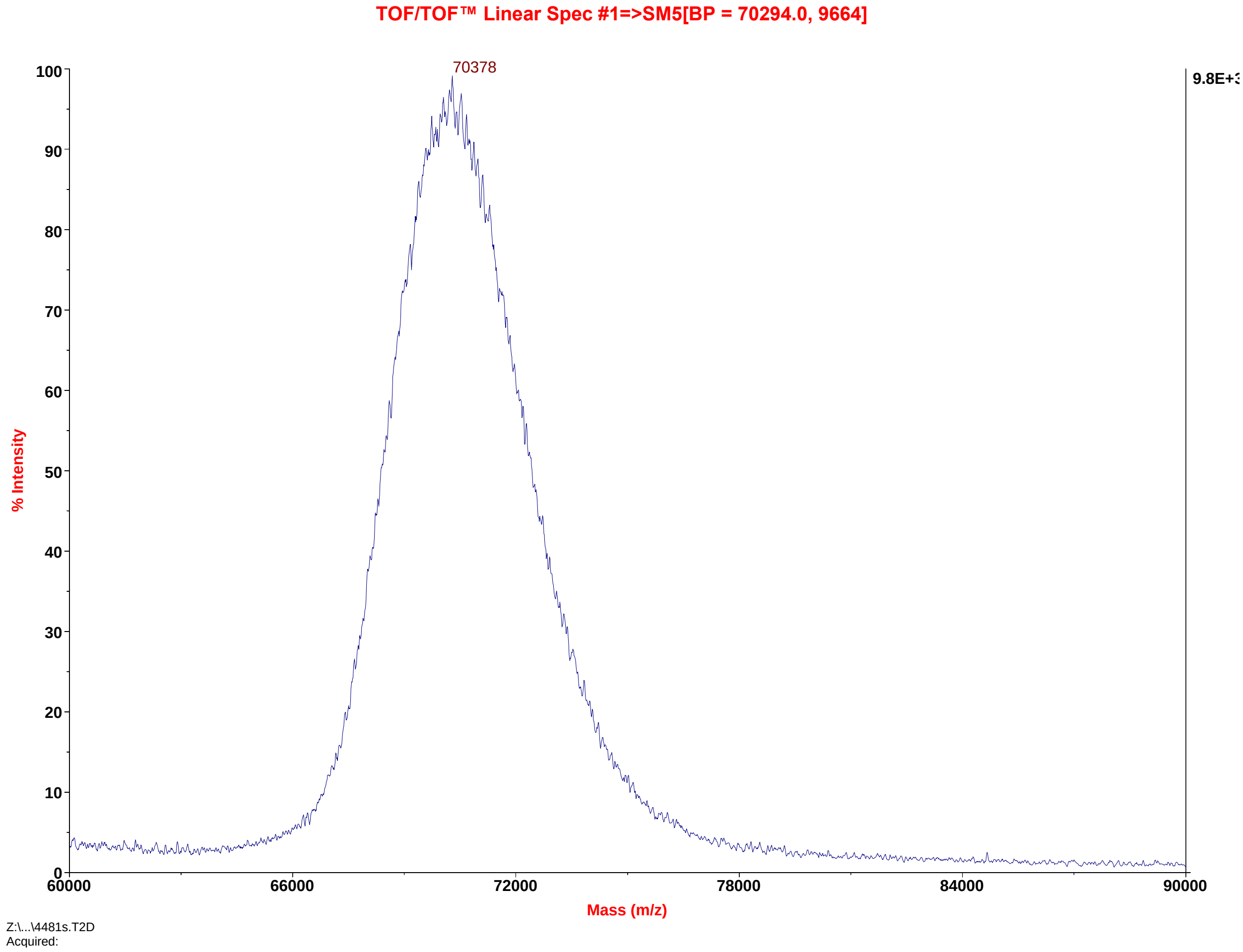

Figure S8) IC$_{50}$ values of vaccines from SPR

Vaccination Group	Analyte	Week 3	Week 5	Week 8
Fent-KLH	Fentanyl	67 (52 to 85)	15 (12 to 19)	12 (8 to 18)
	6-AM [a]	--	--	--
Her-KLH	Fentanyl [a]	--	--	--
	6-AM	200 (134 to 244)	17 (16 to 18)	2 (1 to 5)
HF-1	Fentanyl	1369 (715 to 2619)	n.d.[d]	n.d.[d]
	6-AM	4044 (989 to 16530)	312 (very large) [e]	479 (264 to 867)
HF-2	Fentanyl	513 (205 to 1268)	32 (12 to 87)	147 (20 to 1063)
	6-AM	85 (30 to 239)	306 (very large) [e]	214 (very large)
HF-3	Fentanyl	793 (494 to 1272)	128 (13 to 1243)	n.d.[d]
	6-AM	n.d.[d]	5221 (1151 to 23690)	289 (very large) [e]
HF-4	Fentanyl	2799 (1656 to 4733)	1543 (850 to 2800)	818 (488 to 1371)
	6-AM	305 (216 to 430)	203 (153 to 270)	22 (16 to 31)
HF-5	Fentanyl	69 (58 to 82)	12 (7 to 23)	n.d.[d]
	6-AM	291 (very large)[e]	n.d.[d]	469 (very large) [e]
HF-6	Fentanyl	8343 (993 to 70040)	5019 (1608 to 15670)	6746 (1629 to 27940)
	6-AM	280 (219 to 360)	337 (268 to 424)	98 (86 to 111)
HF-7	Fentanyl	864 (634 to 1177)	526 (331 to 835)	1043 (318 to 3418)
	6-AM	516 (372 to 714)	273 (217 to 343)	56 (41 to 77)
HF-8	Fentanyl	423 (123 to 1447)	408 (265 to 629)	120 (94 to 152)
	6-AM	4877 (very large) [e]	501 (39 to 6294)	780400 (very large) [e]
HF-9	Fentanyl	155 (44 to 548)	218 (128 to 371)	174 (132 to 231)
	6-AM	156 (72 to 340)	n.d.[d]	20 (very large) [e]

IC$_{50}$ values determined by surface plasmon resonance (SPR). IC$_{50}$ values are reported in nanomolar as a mean, with 95% confidence interval in parentheses. 6-AM was used as the primary analyte in SPR as it has a longer half-life than heroin at pH 7.4. [a] 6-AM not run for Fent-KLH, fentanyl not run for Her-KLH. [d] Groups were analyzed by SPR but data did not converge due to lack of detected antibody binding. [e] Poor curve fitting of data resulted in ambiguous and very wide confidence intervals.

Figure S9) ELISA midpoint titer values for vaccination groups

Vaccination Group	Antigen	Week 5	Week 8
Fent-KLH	Fent-BSA	762965	91079
	Her-BSA	5570	3351
Her-KLH	Fent-BSA	291	414
	Her-BSA	241424	100837
HF1-KLH	Fent-BSA	13265	9708
	Her-BSA	2412	3342
HF2-KLH	Fent-BSA	159780	21171
	Her-BSA	2709	3688
HF3-KLH	Fent-BSA	204648	12309
	Her-BSA	5080	4044
HF4-KLH	Fent-BSA	2446	1958
	Her-BSA	81840	18280
HF5-KLH	Fent-BSA	7747	3157
	Her-BSA	2816	980.1
HF6-KLH	Fent-BSA	1126	968.9
	Her-BSA	91325	77661
HF7-KLH	Fent-BSA	16855	32641
	Her-BSA	21657	17313
HF8-KLH	Fent-BSA	21912	10805
	Her-BSA	--[a]	--[a]
HF9-KLH	Fent-BSA	7410	7571
	Her-BSA	--[a]	--[a]
Control	Fent-BSA	--[a]	--[a]
	Her-BSA	--[a]	--[a]

Midpoint titers are reported as the mean of three runs of pooled sera samples. [a] Vaccination group was analyzed, but did not show any titers.

Figure S10) Fent-BSA ELISA midpoint titers arranged by linker type.

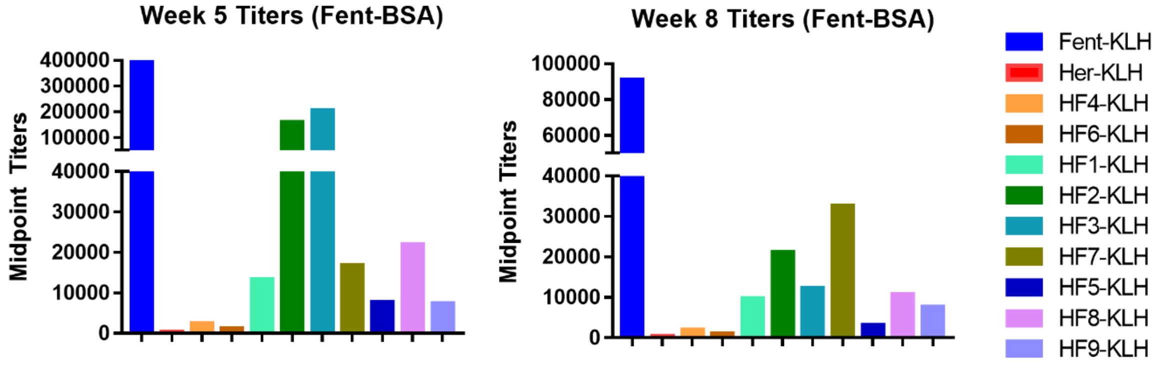

Figure S11) Her-BSA ELISA midpoint titers arranged by linker type.

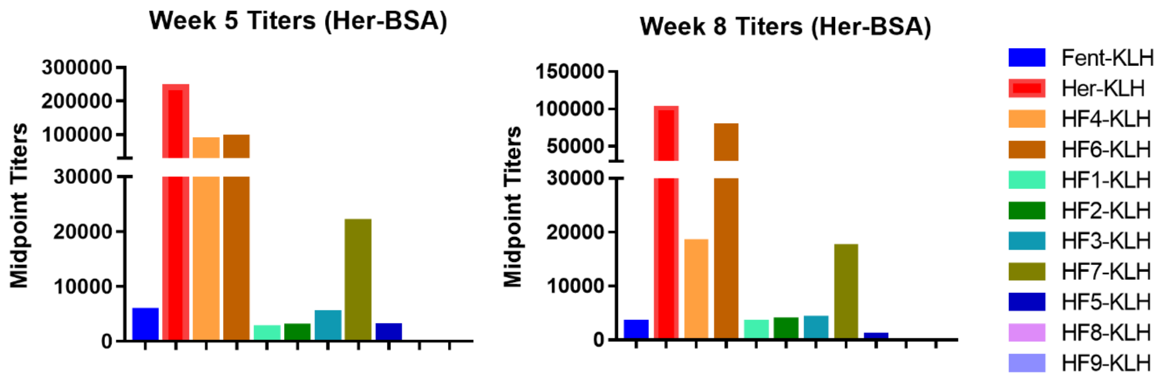

Figure S12) Summary of tail-flick antinociception data

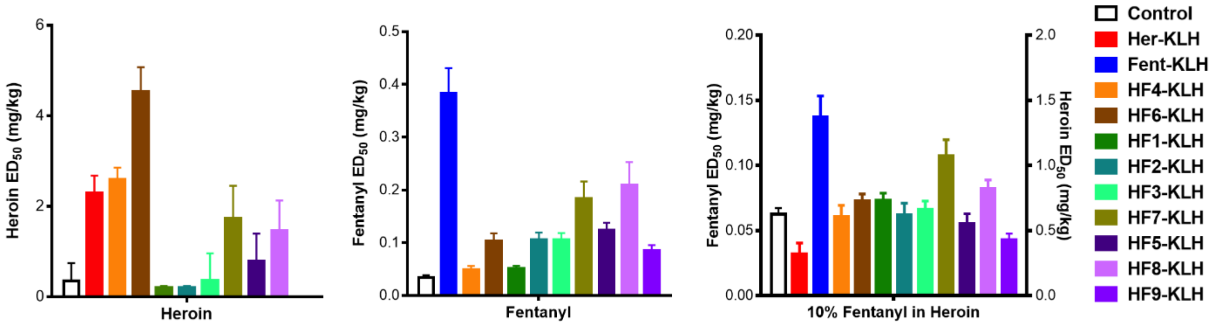

Figure S13) Antinociception data clustered according to linker type

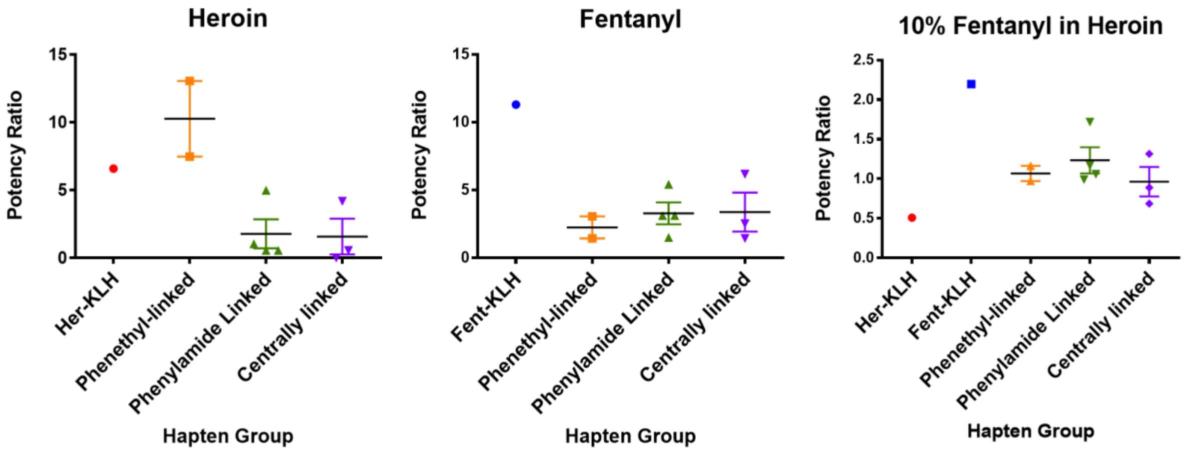

Supplement: File 1 — Supporting experimental results. [file Beilstein_J_Org_Chem-15-1020-s001.pdf]
