# Supplementary material for: A chemically contiguous hapten approach for a heroin–fentanyl vaccine
Source: Beilstein J Org Chem. 2019 May 3;15:1020–31. doi: 10.3762/bjoc.15.100 (PMC6541359; doi:10.3762/bjoc.15.100)
Supplement: File 2 — Experimental procedures for compounds 1–53. [file Beilstein_J_Org_Chem-15-1020-s002.pdf]

BEILSTEIN JOURNAL OF ORGANIC CHEMISTRY

Supporting Information

for

A chemically contiguous hapten approach for a heroin–fentanyl vaccine

Yoshihiro Natori, Candy S. Hwang, Lucy Lin, Lauren C. Smith, Bin Zhou and Kim D. Janda

Beilstein J. Org. Chem. **2019,** *15,* 1020–1031. doi:10.3762/bjoc.15.100

Experimental procedures for compounds 1–53

License and Terms: This is a supporting information file under the terms of the Creative Commons Attribution License (http://creativecommons.org/licenses/by/4.0). Please note that the reuse, redistribution and reproduction in particular requires that the authors and source are credited.
The license is subject to the *Beilstein Journal of Organic Chemistry* terms and conditions: (https://www.beilstein-journals.org/bjoc)

General procedure

Nuclear magnetic resonance (NMR) spectra were recorded on Bruker DRX-600, DRX-500 and DPX-400 instruments. The chemical shifts for NMR are reported in parts per million (ppm) using undeuterated solvents or tertramethylsilane (chloroform-*d*: $CHCl_3$ at ^{1}H NMR δ = 7.26 ppm, ^{13}C NMR δ = 77.16 ppm or TMS at ^{1}H NMR δ = 0 ppm; methanol-d_4: ^{1}H NMR δ = 3.31 ppm, ^{13}C NMR δ = 49.00 ppm). The following abbreviations are used for spin multiplicity: s = singlet, d = doublet, t = triplet, q = quartet, m = multiplet, br = broad. Analytical thin layer chromatography (TLC) was performed on Merck precoated analytical plates, 0.25 mm thick, silica gel 60 F254. Preparative TLC (PTLC) separations were performed on Merck analytical plates (0.50 mm thick) precoated with silica gel 60 F254. Flash column chromatography was performed using the TELEDYNE ISCO (Combiflash Rf) purification system with silica gel (standard grade, 60 Å) unless otherwise noted.

All reactions were carried out under argon unless otherwise noted. Anhydrous dichloromethane was distilled from calcium hydride under positive pressure of nitrogen. Anhydrous tetrahydrofuran (THF) was distilled from sodium benzophenone ketyl under positive pressure of nitrogen. Other commercially available solvents or reagents were used without further purification unless otherwise noted.

Abbreviations:

DMAP = 4-dimethylaminopyridine
DMF = *N*,*N*-dimethylformamide
DMT-MM = 4-(4,6-dimethoxy-1,3,5-triazin-2-yl)-4-methylmorpholinium chloride
EDCI = 1-(3-dimethylaminopropyl)-3-ethylcarbodiimide
NMO = 4-methylmorpholine *N*-oxide
TFA = trifluoroacetic acid
THF = tetrahydrofuran

HF-1 Synthesis (compounds **1–9**)

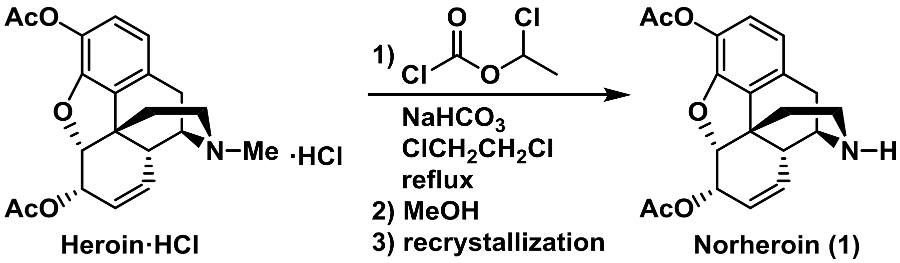

Norheroin (1)

To a solution of heroin·HCl (150 mg, 0.370 mmol) in 1,2-dichloroethane (7.4 mL) were added $NaHCO_3$ (233 mg, 2.77 mmol) and 1-chloroethylchloroformate (0.20 mL, 1.85 mmol) at 0 °C. The reaction mixture was heated to reflux and stirred for 48 h. After cooling to room temperature, the reaction mixture was filtered through a pad of Celite and the filter cake was washed with CH_2Cl_2. After evaporation, the residue was dissolved in MeOH (7.4 mL). The reaction mixture was stirred for 5 h at room temperature, and the solvent was removed under reduced pressure. The residue was dissolved in CH_2Cl_2 (30 mL), and the solution was transferred to a separating funnel. The organic layer was washed with saturated $NaHCO_3$ aq. (10 mL), and dried with Na_2SO_4. After filtration and evaporation, the residue was filtered through a short pad of silica gel eluting with CH_2Cl_2/MeOH (4:1). The eluent was concentrated under reduced pressure, and the residue was purified by recrystallization from CH_2Cl_2/MeOH to give norheroin (**1**) as a pale yellow solid (40.9 mg, 31%).

^{1}H NMR (500 MHz, Methanol-d_4) δ 6.87 (d, J = 8.2 Hz, 1H), 6.75 (d, J = 8.2 Hz, 1H), 5.79 – 5.72 (m, 1H), 5.53 (d, J = 10.0 Hz, 1H), 5.21 (s, 2H), 4.30 (s, 1H), 3.37 – 3.31 (m, 2H), 3.17 – 2.97 (m, 4H), 2.26 (s, 3H), 2.29-2.23 (m, 1H), 2.11 (s, 3H), 2.17-2.05 (m, 1H).

^{13}C NMR (126 MHz, Methanol-d_4) δ 171.91, 170.05, 150.99, 133.91, 131.63, 130.88, 130.47, 126.94, 124.35, 121.09, 89.33, 68.58, 53.32, 43.03, 38.85, 38.14, 33.00, 27.38, 20.48, 20.36.

$[\alpha]_D^{22}$ = -130.2 (*c*. 0.29, CH_3OH)

High resolution mass spectrometry (ESI) found 356.1505 [calculated for $C_{20}H_{22}NO_5$ (M + H^+) 356.1492].

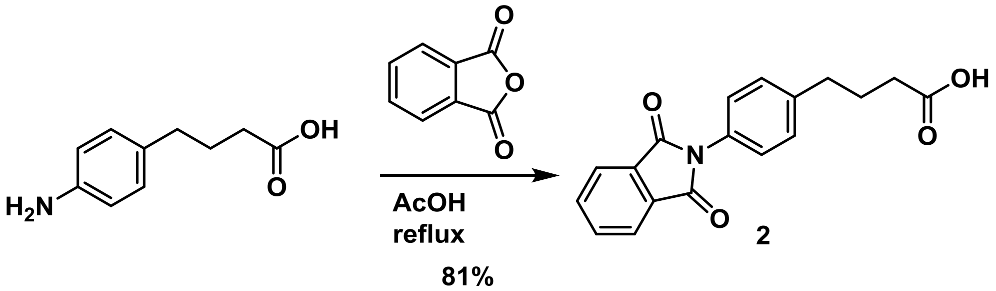

4-(4-(1,3-Dioxoisoindolin-2-yl)phenyl)butanoic acid (2)

To a solution of 4-(4-aminophenyl)butanoic acid (1.77 g, 9.90 mmol) in AcOH (66 mL) was added phthalic anhydride (1.54 g, 10.4 mmol). The reaction mixture was heated to reflux and stirred for 5 h. After cooling to room temperature, the solvent was removed under reduced pressure. Purification by recrystallization from *n*-hexane/EtOAc gave compound **2** as a white solid (2.49 g, 81%).

^{1}H NMR (500 MHz, Chloroform-*d*) δ 7.95 (dd, *J* = 5.4, 3.0 Hz, 2H), 7.79 (dd, *J* = 5.4, 3.0 Hz, 2H), 7.39 – 7.30 (m, 4H), 2.74 (dd, *J* = 8.4, 6.9 Hz, 2H), 2.43 (t, *J* = 7.4 Hz, 2H), 2.07 – 1.96 (m, 2H).

^{13}C NMR (126 MHz, Chloroform-*d*) δ 178.73, 167.54, 141.50, 134.51, 131.94, 129.76, 129.36, 126.72, 123.88, 34.83, 33.26, 26.19.

High resolution mass spectrometry (ESI) found 310.1074 [calculated for $C_{18}H_{16}NO_4$ (M + H^+) 310.1074].

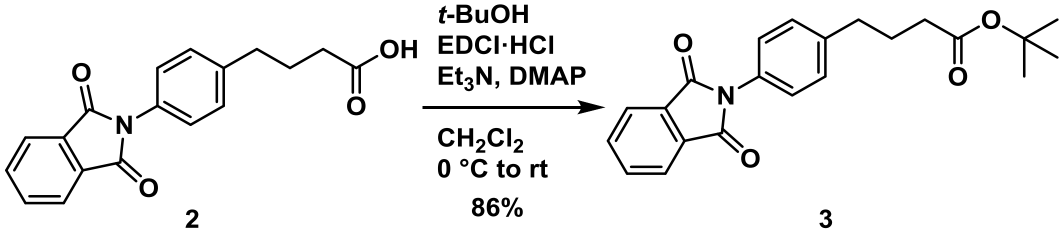

***tert*-Butyl 4-(4-(1,3-dioxoisoindolin-2-yl)phenyl)butanoate (3)**

To a solution of compound **2** (990 mg, 3.20 mmol), DMAP (782 mg, 6.40 mmol) and *t*-BuOH (3.04 mL, 32.0 mmol) in CH_2Cl_2 (11 mL) was added EDCI·HCl (920 mg, 4.80 mmol) at 0 °C. The reaction mixture was stirred at 0 °C for 30 min and at room temperature for 20 h. The mixture was diluted with CH_2Cl_2 (50 mL), washed with 0.5 mol/L HCl aq. (2 ×15 mL) and saturated $NaHCO_3$ aq. (15 mL), and dried with Na_2SO_4. After filtration and evaporation, the crude was purified by silica gel chromatography (*n*-hexane/EtOAc 8:1 to 4:1) to give compound

3 as a white solid (1.01 g, 86%).

^{1}H NMR (500 MHz, Chloroform-*d*) δ 7.96 (dd, *J* = 5.4, 3.0 Hz, 2H), 7.79 (dd, *J* = 5.4, 3.0 Hz, 2H), 7.38 – 7.29 (m, 4H), 2.70 (t, *J* = 7.7 Hz, 2H), 2.27 (t, *J* = 7.5 Hz, 2H), 2.00 – 1.90 (m, 2H), 1.46 (s, 9H).

^{13}C NMR (126 MHz, Chloroform-*d*) δ 172.87, 167.51, 141.91, 134.47, 131.92, 129.59, 129.35, 126.63, 123.83, 80.34, 34.99, 34.92, 28.27, 26.70.

High resolution mass spectrometry (ESI) found 388.1526 [calculated for $C_{22}H_{23}NNaO_4$ (M + Na^+) 388.1519].

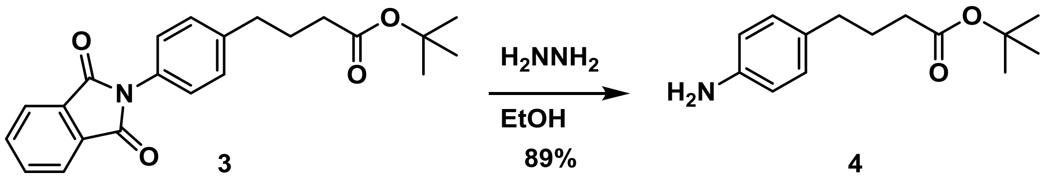

tert-Butyl 4-(4-aminophenyl)butanoate (4)

To a solution of compound **3** (434 mg, 1.19 mmol) in EtOH (20 mL) was added hydrazine (102 µL, 3.20 mmol) at 0 °C. The reaction mixture was stirred at 0 °C for 30 min and at room temperature for 10 h. The reaction mixture was filtered through a pad of Celite and the filter cake was washed with EtOH. After evaporation, the residue was purified by silica gel chromatography (*n*-hexane/EtOAc 8:1 to 4:1) to give compound **4** as a pale yellow oil (250 mg, 89%).

^{1}H NMR (400 MHz, Chloroform-*d*) δ 6.97 (d, *J* = 8.4 Hz, 2H), 6.63 (d, *J* = 8.4 Hz, 2H), 3.56 (s, 2H), 2.52 (t, *J* = 7.5 Hz, 2H), 2.21 (t, *J* = 7.5 Hz, 2H), 1.91 – 1.78 (m, 2H), 1.44 (s, 9H).

^{13}C NMR (126 MHz, Chloroform-*d*) δ 173.20, 144.44, 131.87, 129.41, 115.37, 80.14, 35.07, 34.41, 28.27, 27.22.

High resolution mass spectrometry (ESI) found 236.1653 [calculated for $C_{14}H_{22}NO_2$ (M + H^+) 236.1645].

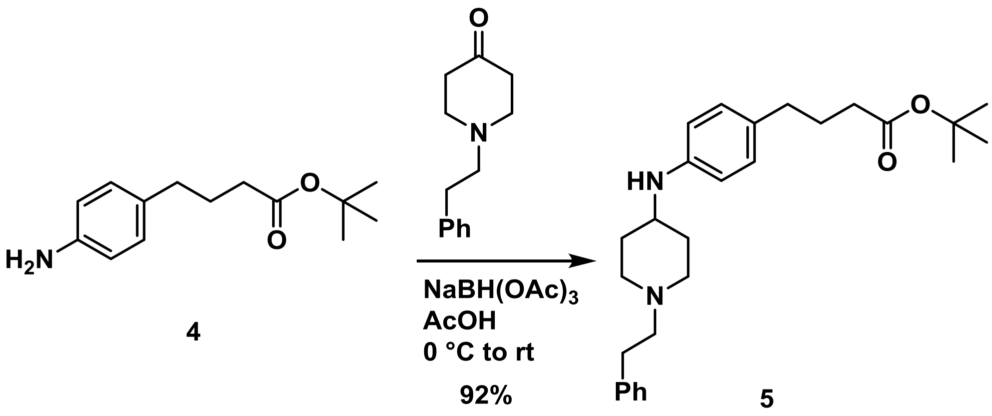

***tert*-Butyl 4-(4-((1-phenethylpiperidin-4-yl)amino)phenyl)butanoate (5)**

To a solution of compound **4** (278 mg, 1.18 mmol) in CH_2Cl_2 (12 mL) were added AcOH (68 μL, 1.18 mmol) and 1-phenethylpiperidin-4-one (264 mg, 1.30 mmol). After stirring for 10 min at room temperature, the reaction mixture was cooled to 0 °C. $NaBH(OAc)_3$ (500 mg, 2.36 mmol) was added to the reaction mixture. The reaction mixture was stirred at 0 °C for 30 min and room temperature for 15 h. The mixture was diluted with CH_2Cl_2 (40 mL), washed with saturated $NaHCO_3$ aq. (10 mL), and dried with Na_2SO_4. After filtration and evaporation, the crude was purified by silica gel chromatography (*n*-hexane/EtOAc 1:1 to 0:1) to give compound **5** as a pale yellow solid (457 mg, 92%).

^{1}H NMR (500 MHz, Chloroform-*d*) δ 7.33 – 7.27 (m, 2H), 7.24 – 7.17 (m, 3H), 7.02 – 6.95 (m, 2H), 6.58 – 6.51 (m, 2H), 3.39 (s, 1H), 3.33 – 3.25 (m, 1H), 2.96 (d, *J* = 12.2 Hz, 2H), 2.86 – 2.79 (m, 2H), 2.65 – 2.58 (m, 2H), 2.52 (t, *J* = 7.6 Hz, 2H), 2.25 – 2.16 (m, 4H), 2.12 – 2.05 (m, 2H), 1.90 – 1.80 (m, 2H), 1.56 – 1.47 (m, 2H), 1.45 (s, 9H).
^{13}C NMR (126 MHz, Chloroform-*d*) δ 173.24, 145.36, 140.53, 130.53, 129.46, 128.83, 128.52, 126.17, 113.54, 80.11, 60.80, 52.64, 50.31, 35.12, 34.35, 34.05, 32.79, 28.27, 27.25.
High resolution mass spectrometry (ESI) found 423.3009 [calculated for $C_{27}H_{39}N_2O_2$ (M + H$^+$) 423.3006].

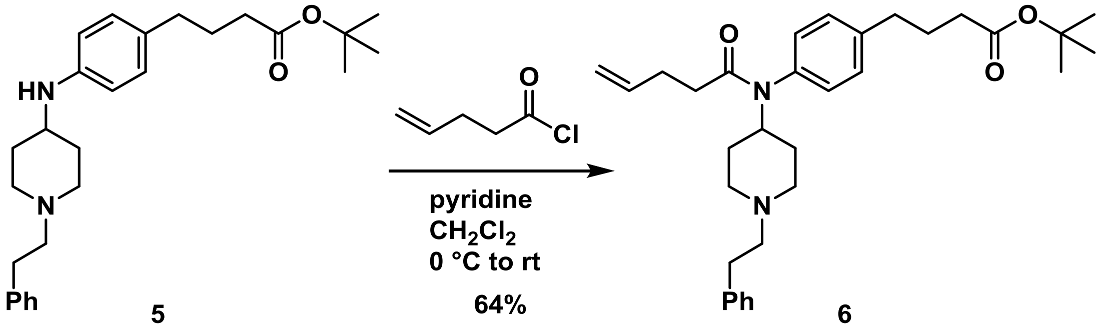

***tert*-Butyl 4-(4-(*N*-(1-phenethylpiperidin-4-yl)pent-4-enamido)phenyl)butanoate (6)**

To a solution of compound **5** (250 mg, 0.592 mmol) and pyridine (190 μL, 2.37 mmol) in CH_2Cl_2 (4 mL) was added 4-pentenoyl chloride (131 μL, 1.18 mmol) at 0 °C. The reaction mixture was stirred at 0 °C for 30 min and at room temperature for 12 h. After cooling to 0 °C, the reaction mixture was quenched by addition of saturated $NaHCO_3$ aq. (10 mL). The whole mixture was extracted with CH_2Cl_2 (2 × 25 mL), and the combined organic layers were dried with Na_2SO_4. After filtration and evaporation, the crude was purified by silica gel chromatography (*n*-hexane/EtOAc 3:1 to 1:1)to give compound **6** as a pale yellow oil (191 mg, 64%).

^{1}H NMR (500 MHz, Chloroform-*d*) δ 7.29 – 7.22 (m, 2H), 7.22 – 7.12 (m, 5H), 7.01 – 6.95 (m, 2H), 5.78 – 5.65 (m, 1H), 4.97 – 4.86 (m, 2H), 4.67 (tt, *J* = 12.2, 4.1 Hz, 1H), 3.03 – 2.96 (m, 2H), 2.77 – 2.69 (m, 2H), 2.65 (t, *J* = 7.7 Hz, 2H), 2.57 – 2.50 (m, 2H), 2.35 – 2.23 (m, 4H), 2.20 – 2.11 (m, 2H), 2.01 (t, *J* = 7.5 Hz, 2H), 1.96 – 1.86 (m, 2H), 1.83 – 1.75 (m, 2H), 1.47 (s, 9H), 1.46 – 1.36 (m, 2H).

^{13}C NMR (126 MHz, Chloroform-*d*) δ 172.68, 172.03, 142.08, 140.22, 137.68, 136.45, 130.31, 129.34, 128.64, 128.39, 126.04, 114.88, 80.28, 60.52, 53.12, 52.17, 34.91, 34.69, 34.39, 33.85, 30.56, 29.46, 28.18, 26.71.

High resolution mass spectrometry (ESI) found 505.3434 [calculated for $C_{32}H_{45}N_2O_3$ (M + H^+) 505.3425].

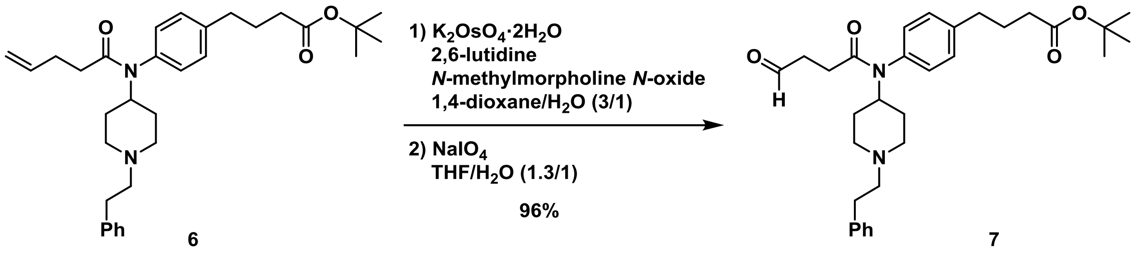

***tert*-Butyl 4-(4-(4-oxo-*N*-(1-phenethylpiperidin-4-yl)butanamido)phenyl)butanoate (7)**

To a solution of compound **6** (44.0 mg, 0.0872 mmol) in 1,4-dioxane/H_2O (3:1, 1.7 mL) were added 2,6-lutidine (30 μL, 0.262 mmol), $K_2OsO_4·2H_2O$ (0.32 mg, 0.872 μmol) and NMO (20.4 mg, 0.174 mmol), and the reaction was stirred at room temperature for 10 h. 10% aq. $Na_2S_2O_3$ (1 mL) was added to the reaction mixture and the mixture was stirred for 10 min. The whole mixture was extracted with CH_2Cl_2 (2 × 10 mL). The combined organic layers were dried with Na_2SO_4 and concentrated.

To a solution of the above crude product in THF/H_2O (1.3:1, 1.7 mL) was added $NaIO_4$ (37.2 mg, 0.174 mmol). The mixture was stirred for 8 h at room temperature. 10% aq. $Na_2S_2O_3$ (1 mL) was added to the reaction mixture and the mixture was stirred for 10 min. The whole mixture was extracted with CH_2Cl_2 (2 × 10 mL). The combined organic layers were dried with Na_2SO_4. After filtration and evaporation, the crude was purified by silica gel chromatography (CH_2Cl_2/MeOH 19:1 to 9:1) to give compound **7** as a pale yellow oil (42.3 mg, 96%, 2 steps).

^{1}H NMR (500 MHz, Chloroform-*d*) δ 9.78 (s, 1H), 7.31 – 7.10 (m, 7H), 7.07 – 6.88 (m, 2H), 4.62 (tt, *J* = 12.2, 3.9 Hz, 1H), 3.11 – 2.92 (m, 2H), 2.80 – 2.60 (m, 6H), 2.58 – 2.51 (m, 2H), 2.31 – 2.20 (m, 4H), 2.20 – 2.12 (m, 2H), 1.95 – 1.87 (m, 2H), 1.82 – 1.73 (m, 2H), 1.47 (s, 9H), 1.28 – 1.23 (m, 2H).
^{13}C NMR (126 MHz, Chloroform-*d*) δ 201.29, 172.81, 171.16, 142.47, 140.14, 136.23, 130.31, 129.61, 128.75, 128.53, 126.21, 80.45, 60.47, 53.09, 52.57, 39.04, 35.07, 34.82, 33.77, 30.42, 29.83, 28.29, 26.82.
High resolution mass spectrometry (ESI) found 507.3217 [calculated for $C_{31}H_{43}N_2O_4$ (M + H$^+$) 507.3217].

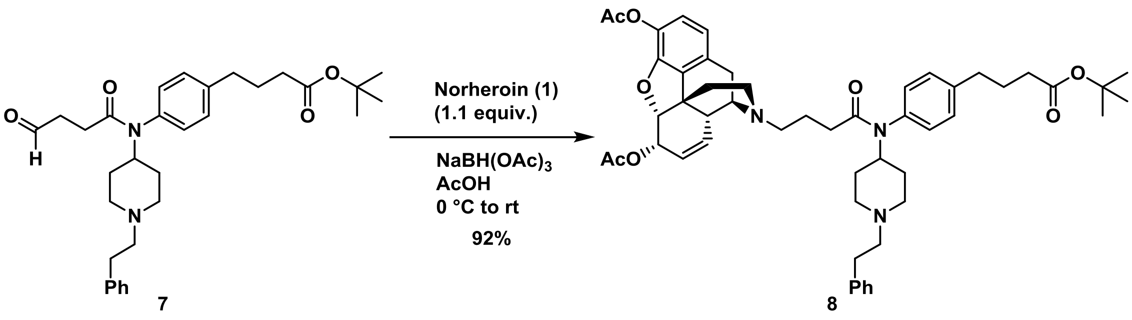

(4*R*,4a*R*,7*S*,7a*R*,12b*S*)-3-(4-((4-(4-(*tert*-butoxy)-4-oxobutyl)phenyl)(1-phenethylpiperidin-4-yl)amino)-4-oxobutyl)-2,3,4,4a,7,7a-hexahydro-1*H*-4,12-methanobenzofuro[3,2-*e*]isoquinoline-7,9-diyl diacetate (8, HF-1 *tert*-Bu ester)

To a solution of compound **7** (14.0 mg, 0.0276 mmol) in CH_2Cl_2 (0.5 mL) were added norheroin (**1**, 10.8 mg, 0.0304 mmol) and AcOH (3.9 μL, 0.0690 mmol). After stirring for 10 min at room temperature, the reaction mixture was cooled to 0 °C and $NaBH(OAc)_3$ (14.6 mg, 0.0690 mmol) was added to the reaction mixture. The reaction mixture was stirred at 0 °C for 30 min and room temperature for 8 h. The mixture was diluted with CH_2Cl_2 (20 mL), washed with saturated $NaHCO_3$ aq. (5 mL), and dried with Na_2SO_4. After filtration and evaporation, the crude was purified by silica gel chromatography (CH_2Cl_2/MeOH 19:1 to 9:1) to give compound **8** as a pale

yellow oil (21.5 mg, 92%).

^{1}H NMR (500 MHz, Chloroform-*d*) δ 7.29 – 7.13 (m, 7H), 6.99 (d, *J* = 8.3 Hz, 2H), 6.74 (d, *J* = 8.2 Hz, 1H), 6.54 (d, *J* = 8.2 Hz, 1H), 5.60 (d, *J* = 10.1 Hz, 1H), 5.39 (dt, *J* = 9.9, 2.9 Hz, 1H), 5.17 – 5.10 (m, 1H), 5.07 (d, *J* = 5.7 Hz, 1H), 4.67 (tt, *J* = 12.2, 4.1 Hz, 1H), 3.41 – 3.32 (m, 1H), 3.01 (d, *J* = 10.4 Hz, 2H), 2.92 (d, *J* = 18.7 Hz, 1H), 2.77 – 2.70 (m, 2H), 2.69 – 2.59 (m, 3H), 2.58 – 2.51 (m, 3H), 2.50 – 2.42 (m, 1H), 2.42 – 2.35 (m, 1H), 2.31 – 2.24 (m, 6H), 2.21 – 2.11 (m, 5H), 2.05 – 1.86 (m, 6H), 1.85 – 1.75 (m, 5H), 1.47 (s, 9H), 1.49 – 1.39 (m, 2H).
^{13}C NMR (126 MHz, Chloroform-*d*) δ 172.81, 172.76, 170.62, 168.59, 149.50, 142.27, 140.32, 136.65, 131.82, 131.72, 130.38, 129.90, 129.47, 128.75, 128.52, 128.41, 126.18, 121.95, 119.45, 88.95, 80.45, 68.32, 60.63, 56.64, 54.27, 53.22, 52.29, 44.93, 43.54, 40.67, 35.32, 35.06, 34.84, 33.93, 32.92, 30.67, 28.30, 26.87, 23.48, 21.85, 20.84, 20.79.
$[\alpha]_D^{22} = -79.2$ (*c*. 0.50, MeOH)
High resolution mass spectrometry (ESI) found 846.4694 [calculated for $C_{51}H_{64}N_3O_8$ (M + H^+) 846.4688].

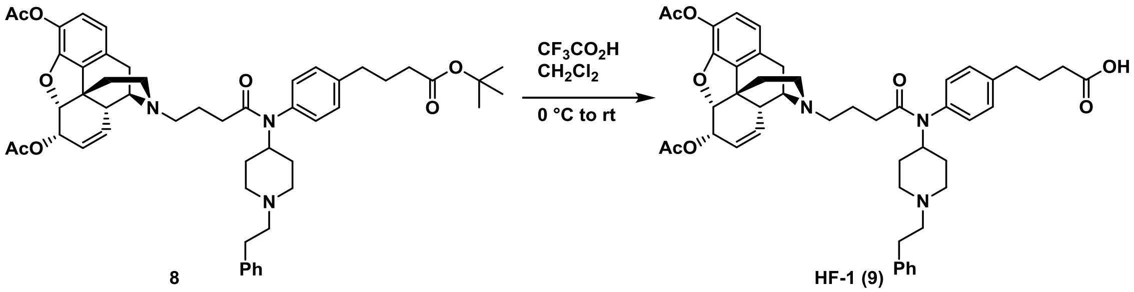

4-(4-(4-((4*R*,4a*R*,7*S*,7a*R*,12b*S*)-7,9-diacetoxy-1,2,4,4a,7,7a-hexahydro-3*H*-4,12-methanobenzofuro[3,2-*e*]isoquinolin-3-yl)-*N*-(1-phenethylpiperidin-4-yl)butanamido)phenyl)butanoic acid (9, HF-1)

To a solution of compound **8** (11.2 mg, 0.0132 mmol) in CH_2Cl_2 (0.5 mL) was added TFA (0.5 mL). The reaction mixture was stirred at 0 °C for 30 min and at room temperature for 30 min. The solvents were removed under reduced pressure. Traces of TFA were removed from a reaction mixture by azeotropic distillations with toluene (2 × 0.5 mL) under reduced pressure. The residue was used as HF-1 (**9**)·TFA without further purification.

HF-2 Synthesis (compounds **10–12**)

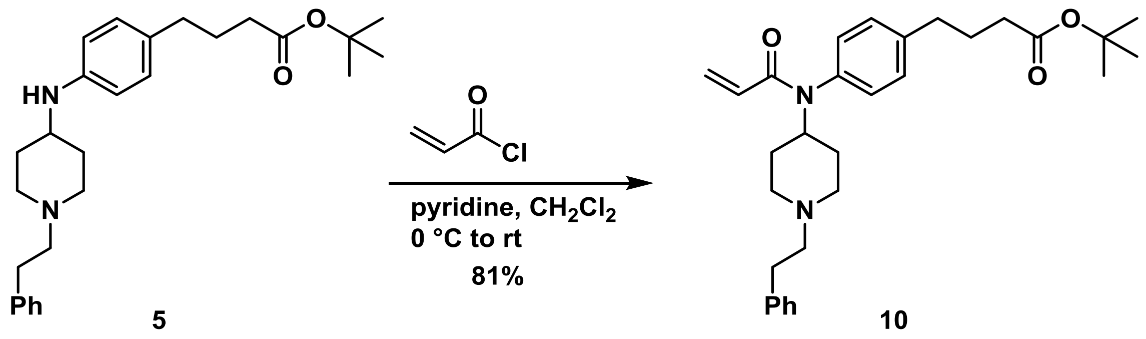

***tert*-Butyl 4-(4-(*N*-(1-phenethylpiperidin-4-yl)acrylamido)phenyl)butanoate (10)**

To a solution of compound **5** (100 mg, 0.237 mmol) and pyridine (57 μL, 0.710 mmol) in CH_2Cl_2 (1.2 mL) was added acryloyl chloride (38 μL, 0.473 mmol) at 0 °C. The reaction mixture was stirred at 0 °C for 30 min and at room temperature for 12 h. After cooling to 0 °C, pyridine (28 μL, 0.348 mmol) and acryloyl chloride (19 μL, 0.235 mmol) were added to the reaction mixture. The reaction mixture was stirred at 0 °C for 30 min and room temperature for 1.5 h. After cooling to 0 °C, the reaction mixture was quenched by addition of saturated $NaHCO_3$ aq. (10 mL). The whole mixture was extracted with CH_2Cl_2 (2 × 30 mL), and the combined organic layers were dried with Na_2SO_4. After filtration and evaporation, the crude was purified by silica gel chromatography (CH_2Cl_2/MeOH 50:1 to 19:1) to give compound **10** as a pale yellow oil (91.0 mg, 81%).

^{1}H NMR (500 MHz, Chloroform-*d*) δ 7.29 – 7.22 (m, 2H), 7.22 – 7.13 (m, 5H), 7.02 – 6.96 (m, 2H), 6.32 (dd, *J* = 16.7, 2.1 Hz, 1H), 5.83 (dd, *J* = 16.7, 10.3 Hz, 1H), 5.45 (dd, *J* = 10.3, 2.1 Hz, 1H), 4.76 – 4.68 (m, 1H), 3.02 (d, *J* = 11.2 Hz, 2H), 2.77 – 2.70 (m, 2H), 2.69 – 2.62 (m, 2H), 2.59 – 2.51 (m, 2H), 2.27 (t, *J* = 7.4 Hz, 2H), 2.23 – 2.14 (m, 2H), 1.96 – 1.87 (m, 2H), 1.87 – 1.79 (m, 2H), 1.47 (s, 9H), 1.56 – 1.41 (m, 2H).
^{13}C NMR (126 MHz, Chloroform-*d*) δ 172.83, 165.62, 142.33, 140.33, 136.02, 130.62, 129.42, 128.77, 128.53, 127.38, 126.19, 80.46, 60.63, 53.18, 52.60, 35.07, 34.84, 33.94, 30.54, 28.30, 26.85.
High resolution mass spectrometry (ESI) found 477.3124 [calculated for $C_{30}H_{41}N_2O_3$ (M + H$^+$) 477.3112].

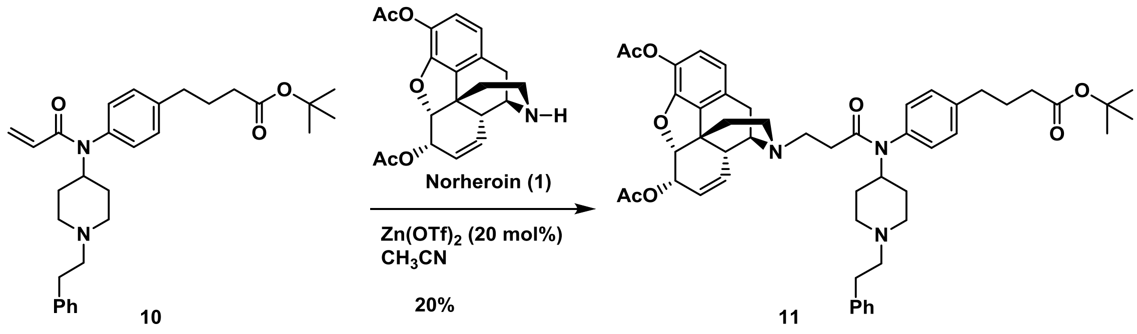

(4*R*,4a*R*,7*S*,7a*R*,12b*S*)-3-(3-((4-(4-(*tert*-butoxy)-4-oxobutyl)phenyl)(1-phenethylpiperidin-4-yl)amino)-3-oxopropyl)-2,3,4,4a,7,7a-hexahydro-1*H*-4,12-methanobenzofuro[3,2-*e*]isoquinoline-7,9-diyl diacetate (11, HF-2 *tert*-Bu ester)

To a solution of compound **10** (8.0 mg, 0.0168 mmol) and norheroin (**1**, 7.1 mg, 0.0201 mmol) in CH_3CN (0.34 mL) was added $Zn(OTf)_2$ (1.2 mg, 0.00336 mmol). After stirring for 12 h, the reaction mixture was cooled to 0 °C. The reaction mixture was quenched by addition of saturated $NaHCO_3$ aq (1 mL). The whole mixture was extracted with CH_2Cl_2 (2× 5 mL), and the combined organic layers were dried with Na_2SO_4. After filtration and evaporation, the crude was purified by preparative TLC (CH_2Cl_2/MeOH 8:1) to give compound **11** as a pale yellow oil (2.7 mg, 20%).

^{1}H NMR (600 MHz, Chloroform-*d*) δ 7.29 – 7.23 (m, 2H), 7.23 – 7.13 (m, 5H), 7.04 – 6.96 (m, 2H), 6.74 (d, *J* = 8.2 Hz, 1H), 6.55 (d, *J* = 8.2 Hz, 1H), 5.59 (dt, *J* = 9.9, 3.0 Hz, 1H), 5.37 (dt, *J* = 9.9, 2.7 Hz, 1H), 5.15 – 5.10 (m, 1H), 5.06 (dd, *J* = 6.6, 1.1 Hz, 1H), 4.67 (tt, *J* = 11.8, 3.8 Hz, 1H), 3.33 – 3.29 (m, 1H), 3.02 (d, *J* = 10.9 Hz, 2H), 2.96 – 2.86 (m, 2H), 2.77 – 2.70 (m, 2H), 2.68 – 2.60 (m, 4H), 2.58 – 2.52 (m, 2H), 2.40 (dd, *J* = 12.1, 3.8 Hz, 1H), 2.34 – 2.22 (m, 7H), 2.21 – 2.06 (m, 7H), 1.96 – 1.86 (m, 3H), 1.80 (d, *J* = 13.0 Hz, 3H), 1.47 (s, 9H), 1.48 – 1.41 (m, 2H).
^{13}C NMR (151 MHz, Chloroform-*d*) δ 172.81, 171.80, 170.68, 168.62, 149.48, 142.38, 140.32, 136.55, 132.54, 131.81, 131.66, 130.39, 129.82, 129.54, 128.78, 128.54, 128.44, 126.20, 121.99, 119.50, 88.92, 80.49, 68.33, 60.62, 58.12, 53.19, 52.39, 51.65, 44.66, 43.44, 40.65, 35.25, 35.07, 34.84, 33.96, 30.65, 29.85, 28.31, 26.86, 22.51, 20.86, 20.80.
High resolution mass spectrometry (ESI) found 832.4545 [calculated for $C_{50}H_{62}N_3O_8$ (M + H^+) 832.4531].

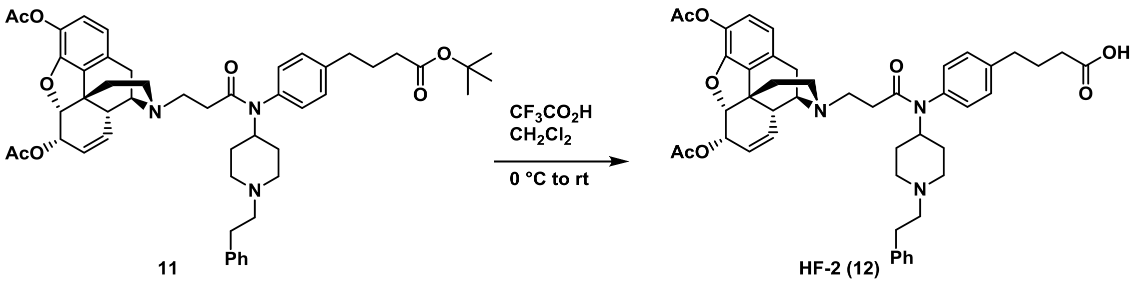

11 HF-2 (12)

4-(4-(3-((4*R*,4a*R*,7*S*,7a*R*,12b*S*)-7,9-diacetoxy-1,2,4,4a,7,7a-hexahydro-3*H*-4,12-methanobenzofuro[3,2-*e*]isoquinolin-3-yl)-*N*-(1-phenethylpiperidin-4-yl)propanamido)phenyl)butanoic acid (12, HF-2)

To a solution of compound **11** (2.7 mg, 0.00324 mmol) in CH_2Cl_2 (0.3 mL) was added TFA (0.3 mL). The reaction mixture was stirred at 0 °C for 30 min and at room temperature for 30 min. The solvents were removed under reduced pressure. Traces of TFA were removed from a reaction mixture by azeotropic distillations with toluene (2 × 0.5 mL) under reduced pressure. The residue was used as HF-2 (**12**)·TFA without further purification.

HF-3 Synthesis (compounds **13–18**)

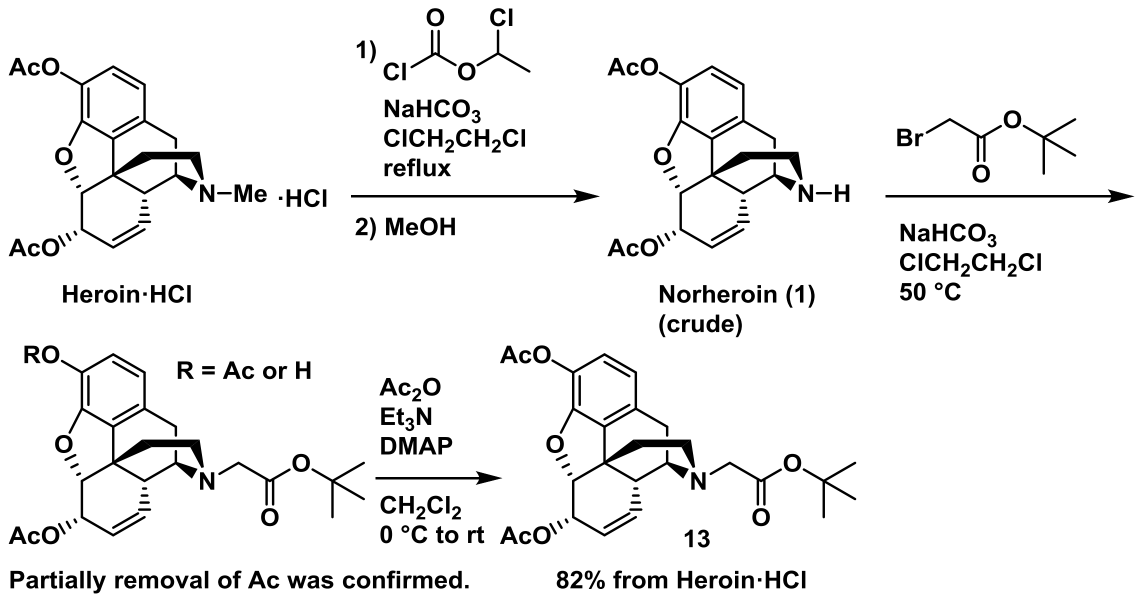

(4*R*,4a*R*,7*S*,7a*R*,12b*S*)-3-(2-(*tert*-Butoxy)-2-oxoethyl)-2,3,4,4a,7,7a-hexahydro-1*H*-4,12-methanobenzofuro[3,2-*e*]isoquinoline-7,9-diyl diacetate (13)

To a solution of Heroin·HCl (150 mg, 0.370 mmol) in 1,2-dichloroethane (7.4 mL) were added $NaHCO_3$ (233 mg, 2.77 mmol) and chloroethyl chloroformate (0.20 mL, 1.85 mmol) at 0 °C. The reaction mixture was heated to reflux and stirred for 48 h. After cooling to room temperature, the reaction mixture was filtered through a pad of Celite and the filter cake was washed with CH_2Cl_2. After evaporation, the residue was dissolved in MeOH (7.4 mL). The reaction mixture was stirred for 5 h at room temperature, and the solvent was removed under reduced pressure. The residue was dissolved in CH_2Cl_2, and the solution was transferred to a separating funnel. The organic layer was washed with saturated $NaHCO_3$ aq., and dried with Na_2SO_4. After filtration and evaporation, the crude product was used for next step without further purification.

To a solution of the above crude product in 1,2-dichloroethane (3.7 mL) were added $NaHCO_3$ (93.3 mg, 1.11 mmol) and *tert*-butyl 2-bromoacetate (81.4 μL, 0.555 mmol) at room temperature. The reaction mixture was stirred for 14 h at 50 °C. After cooling to room temperature, the reaction mixture was diluted with CH_2Cl_2. The organic layer was washed with saturated $NaHCO_3$ aq., and dried with Na_2SO_4. After filtration and evaporation, the residue was filtered through a short pad of silica gel eluting with (*n*-hexane/EtOAc 1:1). The eluent was concentrated under reduced pressure to obtain the mixture of compound **13** and mono-Ac-deprotected compound (131 mg).

To a solution of the above mixture, Et_3N (85 μL, 0.612 mmol) and DMAP (3.7 mg, 0.0306 mmol) in CH_2Cl_2 (3.1 mL) were added Ac_2O (29 μL, 0.306 mmol) at 0 °C. The reaction mixture was stirred at 0 °C for 30 min at room temperature for 30 min. The mixture was diluted with CH_2Cl_2, washed with saturated $NaHCO_3$ aq., and dried with Na_2SO_4. After filtration and evaporation, the crude was purified by silica gel chromatography (*n*-hexane/EtOAc 3:1 to 3:2) to give compound **13** as a pale yellow solid (142 mg, 82% from heroin·HCl).

^{1}H NMR (600 MHz, Chloroform-*d*) δ 6.76 (d, *J* = 8.2 Hz, 1H), 6.56 (d, *J* = 8.2 Hz, 1H), 5.64 – 5.58 (m, 1H), 5.41 (dt, *J* = 10.0, 2.6 Hz, 1H), 5.17 – 5.09 (m, 2H), 3.51 (dd, *J* = 6.1, 3.3 Hz, 1H), 3.32 (d, *J* = 16.7 Hz, 1H), 3.21 (d, *J* = 16.7 Hz, 1H), 2.94 (d, *J* = 18.8 Hz, 1H), 2.90 – 2.86 (m, 1H), 2.77 – 2.70 (m, 1H), 2.48 – 2.36 (m, 2H), 2.26 (s, 3H), 2.15-2.10 (m, 1H), 2.13 (s, 3H), 1.89 – 1.83 (m, 1H), 1.47 (s, 9H).
^{13}C NMR (151 MHz, Chloroform-*d*) δ 170.59, 170.09, 168.58, 149.51, 132.05, 131.94, 131.67, 129.55, 128.58, 122.09, 119.43, 88.86, 81.32, 68.19, 57.61, 57.44, 45.33, 43.04, 40.43, 35.12, 28.28, 22.58, 20.81, 20.78.
$[\alpha]_D^{22}$ = -144.07 (*c*. 1.4, CH_3OH)
High resolution mass spectrometry (ESI) found 470.2183 [calculated for $C_{26}H_{32}NO_7$ ($M + H^+$) 470.2173].

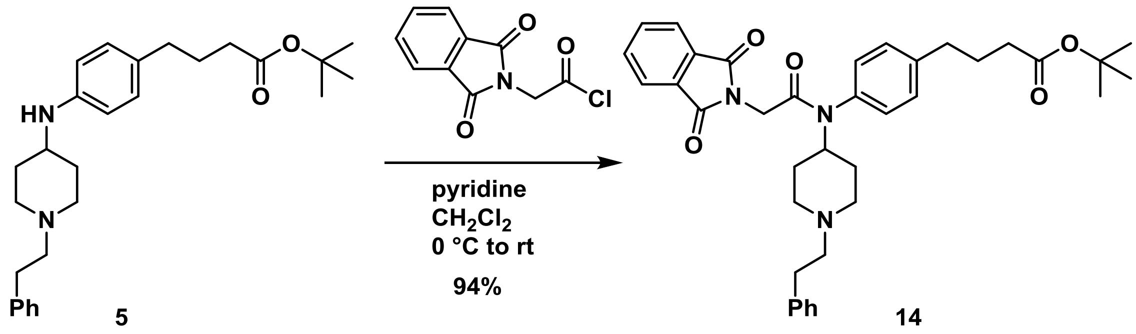

tert-Butyl 4-(4-(2-(1,3-dioxoisoindolin-2-yl)-*N*-(1-phenethylpiperidin-4-yl)acetamido)phenyl)butanoate (14)

To a solution of *N*-phthaloylglycine (36.3 mg, 0.177 mmol) in CH_2Cl_2 (1.8 mL) were added $(COCl)_2$ (46 μL, 0.532 mmol) and DMF (1 drop) at 0 °C. The reaction mixture was stirred at 0 °C for 10 min and at room temperature for 2 h. The solvents were removed under reduced

pressure. Azeotropic distillations were carried out with toluene (2 × 2 mL) under reduced pressure. The residue (acid chloride) was used for next step without further purification.

To a solution of compound **5** (25.0 mg, 0.0592 mmol) and pyridine (43 μL, 0.532 mmol) in CH_2Cl_2 (0.3 mL) was added a solution of the above crude product (acid chloride) in CH_2Cl_2 (0.6 mL) at 0 °C. The reaction mixture was stirred at 0 °C for 30 min and at room temperature for 9 h. After cooling to 0 °C, the reaction mixture was quenched by addition of saturated $NaHCO_3$ aq. (5 mL). The whole mixture was extracted with CH_2Cl_2 (2 × 10 mL), and the combined organic layers were dried with Na_2SO_4. After filtration and evaporation, the crude was purified by silica gel chromatography (*n*-hexane/EtOAc 1:1 to 1:2) to give compound **14** as a pale yellow oil (34.0 mg, 94%).

^{1}H NMR (500 MHz, Chloroform-*d*) δ 7.88 – 7.80 (m, 2H), 7.74 – 7.66 (m, 2H), 7.31 – 7.11 (m, 9H), 4.59 (tt, *J* = 12.2, 4.1 Hz, 1H), 4.03 (s, 2H), 3.00 (d, *J* = 12.0 Hz, 2H), 2.76 – 2.65 (m, 4H), 2.56 – 2.49 (m, 2H), 2.28 (t, *J* = 7.4 Hz, 2H), 2.15 – 2.06 (m, 2H), 1.98 – 1.88 (m, 2H), 1.88 – 1.80 (m, 2H), 1.52-1.45 (m, 2H), 1.48 (s, 9H).

^{13}C NMR (126 MHz, Chloroform-*d*) δ 172.69, 168.08, 165.61, 143.07, 140.24, 134.87, 134.02, 132.34, 130.41, 129.89, 128.69, 128.45, 126.11, 123.47, 80.41, 60.49, 53.36, 53.03, 40.53, 34.98, 34.79, 33.90, 30.34, 28.24, 26.77.

High resolution mass spectrometry (ESI) found 610.3279 [calculated for $C_{37}H_{44}N_3O_5$ (M + H$^+$) 610.3275].

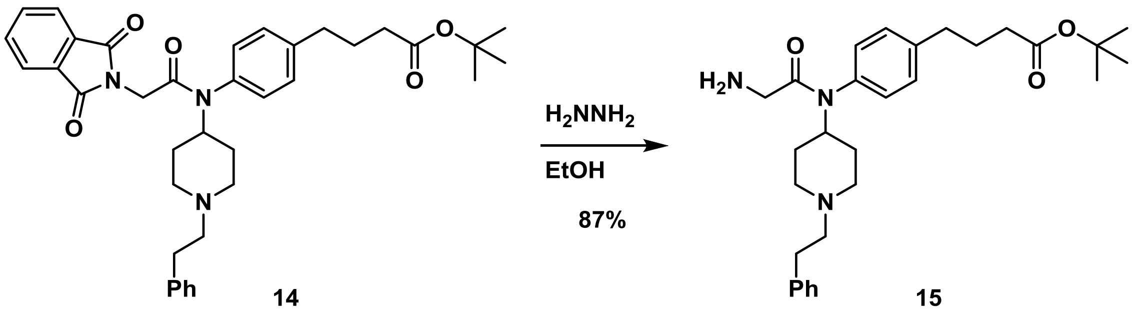

14 15

tert-Butyl 4-(4-(2-amino-*N*-(1-phenethylpiperidin-4-yl)acetamido)phenyl)butanoate (15)

A solution of compound **14** (34.0 mg, 0.0558 mmol) in EtOH (0.6 mL) was added hydrazine (4 μL, 0.126 mmol) at 0 °C. The reaction mixture was stirred at 0 °C for 30 min and at room temperature for 12 h. Hydrazine (8 μL, 0.252 mmol) was added to the reaction mixture. After stirring for 12 h, the reaction mixture was filtered through a pad of Celite and the filter cake was washed with EtOH. After evaporation, the residue was purified by silica gel chromatography

(CH_2Cl_2/MeOH 9:1 to 6:1) to give compound **15** as a pale yellow oil (23.3 mg, 87%).

^{1}H NMR (500 MHz, Chloroform-*d*) δ 7.29 – 7.22 (m, 2H), 7.22 – 7.12 (m, 5H), 7.04 – 6.95 (m, 2H), 4.65 (tt, *J* = 12.2, 4.1 Hz, 1H), 3.07 – 2.96 (m, 4H), 2.77 – 2.70 (m, 2H), 2.65 (t, *J* = 7.7 Hz, 2H), 2.59 – 2.51 (m, 2H), 2.26 (t, *J* = 7.4 Hz, 2H), 2.22 – 2.13 (m, 2H), 2.05 (brs, 2H), 1.96 – 1.85 (m, 2H), 1.84 – 1.77 (m, 2H), 1.51-1.45 (m, 2H), 1.47 (s, 9H).
^{13}C NMR (126 MHz, Chloroform-*d*) δ 172.74, 172.56, 142.66, 140.21, 135.06, 130.27, 129.62, 128.72, 128.49, 126.16, 80.41, 60.55, 53.10, 52.76, 44.77, 34.97, 34.76, 33.88, 30.44, 28.26, 26.77.
High resolution mass spectrometry (ESI) found 480.3227 [calculated for $C_{29}H_{42}N_3O_3$ (M + H^+) 480.3221].

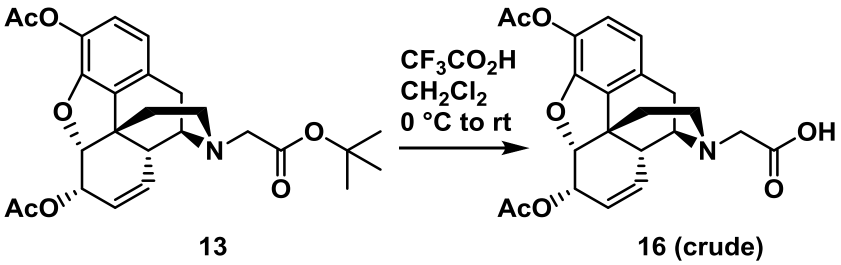

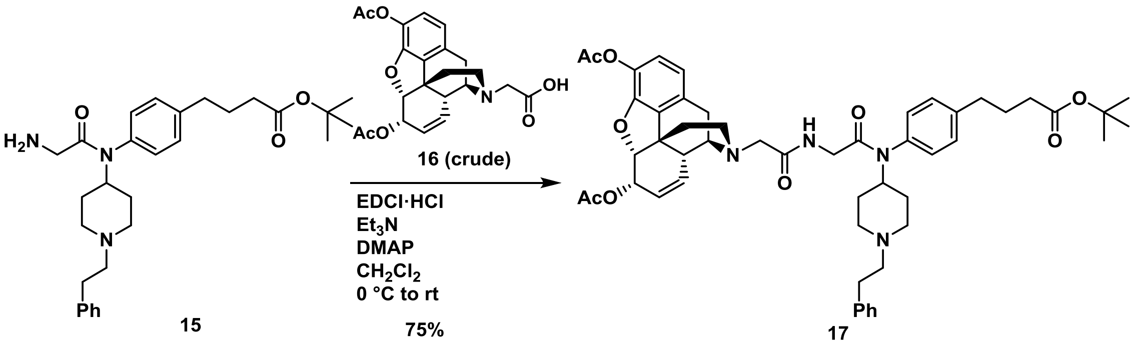

(4*R*,4a*R*,7*S*,7a*R*,12b*S*)-3-(2-((2-((4-(4-(*tert*-butoxy)-4-oxobutyl)phenyl)(1-phenethylpiperidin-4-yl)amino)-2-oxoethyl)amino)-2-oxoethyl)-2,3,4,4a,7,7a-hexahydro-1*H*-4,12-methanobenzofuro[3,2-*e*]isoquinoline-7,9-diyl diacetate (17, HF-3 *tert*-Bu ester)

To a solution of compound **13** (19.2 mg, 0.0410 mmol) in CH_2Cl_2 (0.7 mL) was added TFA (0.7 mL). The reaction mixture was stirred at 0 °C for 30 min and at room temperature for 4 h. The solvents were removed under reduced pressure. Traces of TFA were removed from a reaction mixture by azeotropic distillations with toluene (2 × 1 mL) under reduced pressure.

To a solution of the above crude product (**16**) in CH_2Cl_2 (0.3 mL) were added Et_3N (17.1 μL,

0.123 mmol), a solution of amine **15** in CH_2Cl_2 (0.25 mL) and DMAP (1 crop). After cooling to 0 °C, EDCI·HCl (7.9 mg, 0.0410 mmol) was added to the mixture. The reaction mixture was stirred at 0 °C for 30 min and at room temperature for 8 h. The mixture was diluted with CH_2Cl_2 (10 mL), washed with saturated $NaHCO_3$ aq. (3 mL), and dried with Na_2SO_4. After filtration and evaporation, the crude was purified by silica gel chromatography (CH_2Cl_2/MeOH 40:1 to 15:1) to give compound **17** as a colorless oil (17.8 mg, 75%).

^{1}H NMR (500 MHz, Chloroform-*d*) δ 8.23 (t, *J* = 4.4 Hz, 1H), 7.29 – 7.20 (m, 4H), 7.20 – 7.12 (m, 3H), 7.06 – 6.98 (m, 2H), 6.77 (d, *J* = 8.2 Hz, 1H), 6.59 (d, *J* = 8.2 Hz, 1H), 5.68 – 5.61 (m, 1H), 5.40 (dt, *J* = 10.0, 2.9 Hz, 1H), 5.22 – 5.12 (m, 2H), 4.66 (tt, *J* = 11.8, 3.8 Hz, 1H), 3.74 – 3.52 (m, 2H), 3.41 (dd, *J* = 5.9, 3.3 Hz, 1H), 3.24 (d, *J* = 16.6 Hz, 1H), 3.10 – 2.98 (m, 3H), 2.95 – 2.85 (m, 2H), 2.80 – 2.61 (m, 5H), 2.61 – 2.46 (m, 4H), 2.32-2.25 (m, 2H), 2.27 (s, 3H), 2.21 – 2.11 (m, 3H), 2.13(s, 3H), 2.01 – 1.87 (m, 3H), 1.82 (d, *J* = 10.6 Hz, 2H), 1.57 – 1.41 (m, 2H), 1.48 (s, 9H).

^{13}C NMR (126 MHz, Chloroform-*d*) δ 172.78, 170.60, 170.31, 168.53, 168.23, 149.58, 143.25, 134.22, 132.04, 131.83, 131.47, 130.22, 130.00, 129.23, 128.84, 128.75, 128.53, 126.21, 122.23, 119.59, 88.76, 80.46, 68.24, 60.56, 59.30, 58.73, 53.12, 53.03, 45.56, 43.02, 42.38, 41.03, 35.48, 35.16, 34.85, 30.43, 29.84, 28.30, 26.77, 24.23, 20.82, 20.77.

$[\alpha]_D^{22}$ = -53.9 (*c*. 1.1, CH_3OH)

High resolution mass spectrometry (ESI) found 875.4592 [calculated for $C_{51}H_{63}N_4O_9$ (M + H^+) 875.4589].

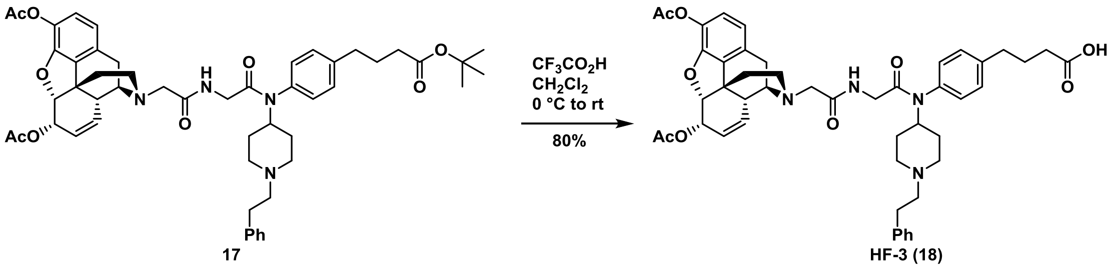

4-(4-(2-(2-((4*R*,4a*R*,7*S*,7a*R*,12b*S*)-7,9-diacetoxy-1,2,4,4a,7,7a-hexahydro-3*H*-4,12-methanobenzofuro[3,2-*e*]isoquinolin-3-yl)acetamido)-*N*-(1-phenethylpiperidin-4-yl)acetamido)phenyl)butanoic acid (18, HF-3)

To a solution of compound **17** (12.5 mg, 0.0143 mmol) in CH_2Cl_2 (0.3 mL) was added TFA (0.3 mL) at 0 °C. The reaction mixture was stirred at 0 °C for 30 min and at room temperature for 30 min. The solvents were removed under reduced pressure. Traces of TFA were removed from a reaction mixture by azeotropic distillations with toluene (2 × 0.5 mL) under reduced pressure.

The residue was purified by silica gel chromatography (CH_2Cl_2/MeOH 8:1) to give HF-3 (**18**) as a colorless oil (9.4 mg, 80%).

^{1}H NMR (600 MHz, Chloroform-*d*) δ 8.33 (t, *J* = 4.6 Hz, 1H), 7.29 – 7.23 (m, 4H), 7.22 – 7.13 (m, 3H), 7.04 – 6.98 (m, 2H), 6.77 (d, *J* = 8.1 Hz, 1H), 6.58 (d, *J* = 8.2 Hz, 1H), 5.68 – 5.62 (m, 1H), 5.40 (dt, *J* = 10.0, 2.8 Hz, 1H), 5.22 – 5.12 (m, 2H), 4.72 (tt, *J* = 12.7, 4.1 Hz, 1H), 3.66 – 3.54 (m, 2H), 3.42 – 3.37 (m, 1H), 3.26 – 3.18 (m, 3H), 3.09 – 3.03 (m, 1H), 2.95 – 2.46 (m, 12H), 2.37 – 2.10 (m, 4H), 2.27 (s, 3H), 2.15 (s, 3H), 1.97 – 1.90 (m, 3H), 1.90 – 1.84 (m, 2H), 1.71 – 1.64 (m, 2H).
^{13}C NMR (151 MHz, Chloroform-*d*) δ 175.72, 170.75, 170.62, 168.54, 168.18, 149.61, 142.95, 133.97, 132.06, 131.77, 131.43, 130.65, 130.11, 130.06, 129.19, 128.89, 128.78, 128.67, 126.49, 122.27, 119.62, 88.77, 68.25, 59.73, 59.03, 58.70, 52.48, 52.05, 45.60, 43.02, 42.65, 41.06, 35.56, 33.62, 32.73, 29.85, 29.45, 25.82, 24.33, 20.84, 20.79.
High resolution mass spectrometry (ESI) found 819.3964 [calculated for $C_{47}H_{55}N_4O_9$ (M + H^+) 819.3963].

HF-4 Synthesis (compounds **19**–**27**)

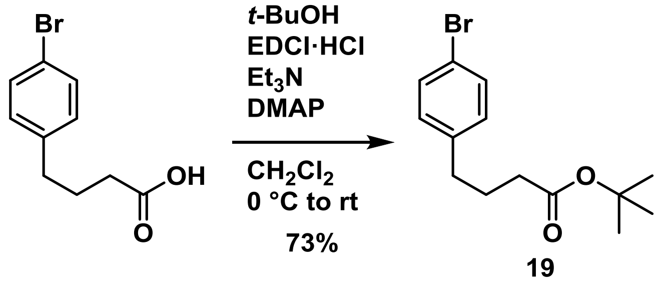

***tert*-Butyl 4-(4-bromophenyl)butanoate (19)**

To a solution of 4-(4-bromophenyl)butanoic acid (3.00 g, 12.3 mmol), DMAP (3.02 g, 24.7 mmol) and *t*-BuOH (11.7 mL, 123 mmol) in CH_2Cl_2 (31 mL) was added EDCI·HCl (3.55 g, 18.5 mmol) at 0 °C. The reaction mixture was stirred at 0 °C for 30 min and at room temperature for 12 h. The mixture was diluted with EtOAc (100 mL). The organic layer was washed with 0.5 mol/L HCl aq. (2 × 30 mL) and saturated $NaHCO_3$ aq. (30 mL), and dried with Na_2SO_4. After filtration and evaporation, the crude was purified by silica gel chromatography (*n*-hexane/EtOAc 19:1 to 9:1) to give compound **19** as a colorless oil (2.70 g, 73%).

^{1}H NMR (400 MHz, Chloroform-*d*) δ 7.44 – 7.35 (m, 2H), 7.09 – 7.00 (m, 2H), 2.63 – 2.55 (m, 2H), 2.22 (t, J = 7.4 Hz, 2H), 1.94 – 1.81 (m, 2H), 1.44 (s, 9H).
^{13}C NMR (126 MHz, Chloroform-*d*) δ 172.80, 140.71, 131.53, 130.38, 119.78, 80.36, 34.85, 34.64, 28.26, 26.67.
High resolution mass spectrometry (ESI) found 321.0453 [calculated for $C_{14}H_{19}BrNaO_2$ (M + Na^+) 321.0461].

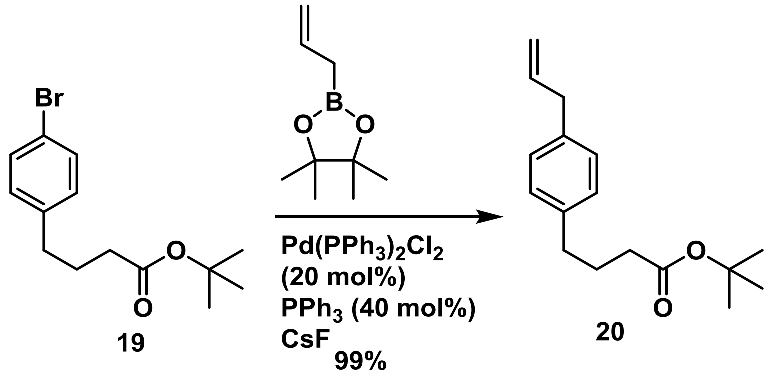

***tert*-Butyl 4-(4-allylphenyl)butanoate (20)**

To a solution of compound **19** (1.00 g, 3.34 mmol), allylboronic acid pinacol ester (1.68 g, 10.0 mmol), pre-dried CsF (2.04 g, 13.4 mmol) and PPh_3 (351 mg, 1.34 mmol) in THF (67 mL) was added $Pd(PPh_3)_2Cl_2$ (469 mg, 0.668 mmol). The reaction mixture was heated to reflux and stirred for 16 h. The reaction mixture was diluted with EtOAc (120 mL), and the solution was transferred to separating funnel. The organic layer was washed with H_2O (30 mL) and brine (30 mL), and dried with Na_2SO_4. After filtration and evaporation, the crude was purified by silica gel chromatography (*n*-hexane/EtOAc 19:1) to give compound **20** as a colorless oil (860 mg, 99%).

^{1}H NMR (500 MHz, Chloroform-*d*) δ 7.11 (s, 4H), 6.02 – 5.90 (m, 1H), 5.12 – 5.02 (m, 2H), 3.36 (dt, *J* = 6.7, 1.4 Hz, 2H), 2.64 – 2.57 (m, 2H), 2.23 (t, *J* = 7.5 Hz, 2H), 1.94 – 1.84 (m, 2H), 1.44 (s, 9H).
^{13}C NMR (126 MHz, Chloroform-*d*) δ 173.08, 139.52, 137.76, 137.73, 128.69, 115.77, 80.24, 40.00, 35.13, 34.89, 28.28, 26.97.
High resolution mass spectrometry (ESI) found 283.1654 [calculated for $C_{17}H_{24}NaO_2$ (M + Na^+) 283.1668].

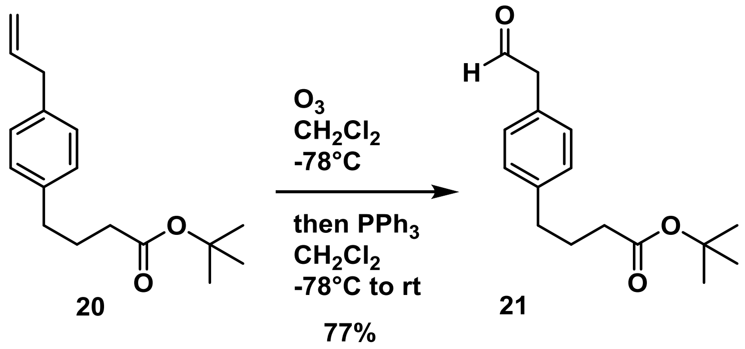

***tert*-Butyl 4-(4-(2-oxoethyl)phenyl)butanoate (21)**
A solution of compound **20** (200 mg, 0.768 mmol) in CH_2Cl_2(10 mL) was cooled to −78 °C and treated with a stream of ozone until a blue color persisted. A stream of argon was passed through the solution for 5 min to remove the excess of ozone from the reaction solution. A solution of PPh_3 (404 mg, 1.54 mmol) in CH_2Cl_2 (5 mL) was added slowly to the reaction solution at −78 °C and allowed to warm to room temperature. After 12 h, the solvent was removed under reduced pressure the crude was purified by silica gel chromatography (*n*-hexane/EtOAc 8:1) to give compound **21** as a colorless oil (156 mg, 77%).

^{1}H NMR (600 MHz, Chloroform-*d*) δ 9.74 (t, *J* = 2.4 Hz, 1H), 7.19 (d, *J* = 8.2 Hz, 2H), 7.14 (d, *J* = 8.3 Hz, 2H), 3.65 (d, *J* = 2.4 Hz, 2H), 2.66 – 2.60 (m, 2H), 2.24 (t, *J* = 7.4 Hz, 2H), 1.95 – 1.86 (m, 2H), 1.45 (s, 9H).
^{13}C NMR (126 MHz, Chloroform-*d*) δ 199.72, 172.93, 141.03, 129.75, 129.43, 129.27, 80.31, 50.33, 35.02, 34.87, 28.26, 26.82.
High resolution mass spectrometry (ESI) found 263.1653 [calculated for $C_{16}H_{23}O_3$ (M + H^+) 263.1642].

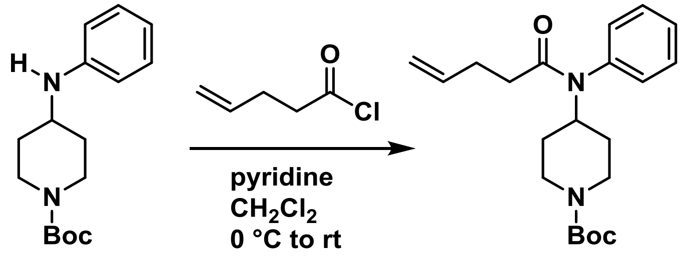

22

***tert*-Butyl 4-(*N*-phenylpent-4-enamido)piperidine-1-carboxylate (22)**

To a solution of 1-*N*-Boc-4-(phenylamino)piperidine (400 mg, 1.45 mmol) and pyridine (280 μL, 3.47 mmol) in CH_2Cl_2 (7.3 mL) was added 4-pentenoyl chloride (192 μL, 1.74 mmol) at 0 °C. The reaction mixture was stirred at 0 °C for 30 min and at room temperature for 5 h. After cooling to 0 °C, pyridine (280 μL, 3.47 mmol) and 4-pentenoyl chloride (192 μL, 1.74 mmol) were added to a reaction mixture. The reaction mixture was stirred at 0 °C for 30 min and room temperature for 10 h. The mixture was diluted with CH_2Cl_2 (50 mL), washed with 0.5 mol/L HCl aq. (2 × 15 mL), saturated $NaHCO_3$ aq. (15 mL) and brine, and dried with Na_2SO_4. After filtration and evaporation, the crude was purified by silica gel chromatography (*n*-hexane/EtOAc 1:1 to 1:2) to give compound **22** as a white solid (440 mg, 85%).

^{1}H NMR (500 MHz, Chloroform-*d*) δ 7.46 – 7.37 (m, 3H), 7.09 – 7.02 (m, 2H), 5.70 (ddt, *J* = 16.8, 10.1, 6.6 Hz, 1H), 4.97 – 4.86 (m, 2H), 4.78 (tt, *J* = 12.0, 3.7 Hz, 1H), 4.11 (brs, 2H), 2.79 (brs, 2H), 2.34 – 2.25 (m, 2H), 2.00 (dd, *J* = 8.1, 6.9 Hz, 2H), 1.83 – 1.73 (m, 2H), 1.39 (s, 9H), 1.28 – 1.16 (m, 2H).
^{13}C NMR (126 MHz, Chloroform-*d*) δ 172.01, 154.68, 138.73, 137.62, 130.41, 129.56, 128.65, 115.10, 79.65, 52.43, 43.32, 34.49, 30.66, 29.50, 28.49.
High resolution mass spectrometry (ESI) found 359.2332 [calculated for $C_{21}H_{31}N_2O_3$ (M + H^+) 359.2329].

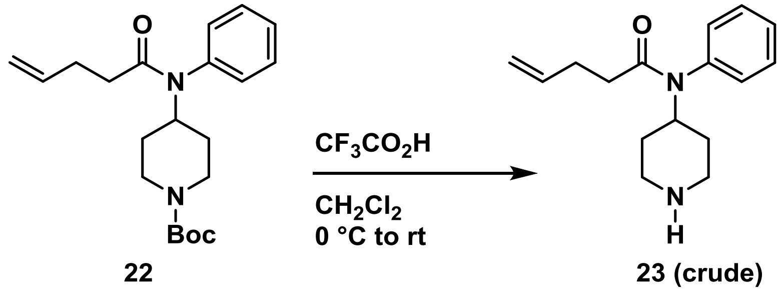

N-Phenyl-N-(piperidin-4-yl)pent-4-enamide (23)

To a solution of compound **22** (200 mg, 0.558 mmol) in CH_2Cl_2 (0.5 mL) was added TFA (2.0 mL) at 0 °C. The reaction mixture was stirred at 0 °C for 30 min and at room temperature for 1 h. After cooling to 0 °C, the reaction was neutralized by slow addition of saturated $NaHCO_3$ aq. (15 mL). The whole mixture was extracted with CH_2Cl_2 (2× 30 mL), and the combined organic layers were dried with Na_2SO_4. After filtration and evaporation, the crude product (compound **23**) was used for next step without further purification.

The ^{1}H and ^{13}C NMR data were collected after purification by silica gel chromatography (CH_2Cl_2/MeOH 9:1 to 1:1).

^{1}H NMR (500 MHz, Chloroform-*d*) δ 7.46 – 7.30 (m, 3H), 7.10 – 7.01 (m, 2H), 5.75 – 5.61 (m, 1H), 5.00 – 4.82 (m, 2H), 4.77 – 4.66 (m, 1H), 3.06 – 2.98 (m, 2H), 2.73 – 2.65 (m, 2H), 2.32 – 2.22 (m, 2H), 2.01 – 1.93 (m, 2H), 1.80 – 1.72 (m, 2H), 1.29 – 1.16 (m, 2H).

^{13}C NMR (126 MHz, Chloroform-*d*) δ 171.81, 138.93, 137.70, 130.49, 129.38, 128.43, 114.97, 77.41, 77.16, 76.90, 52.59, 46.24, 34.52, 32.06, 29.52.

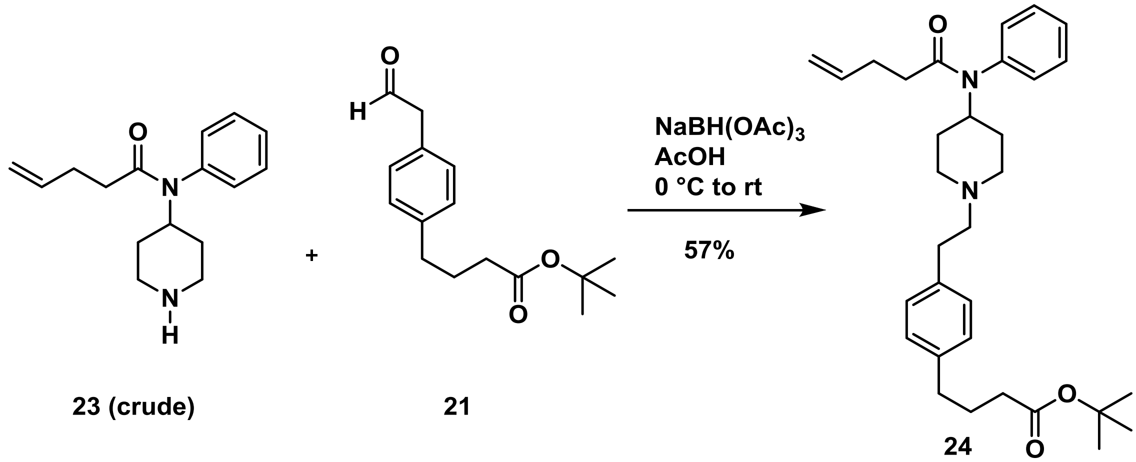

***tert*-Butyl 4-(4-(2-(4-(N-phenylpent-4-enamido)piperidin-1-yl)ethyl)phenyl)butanoate (24)**

To a solution of aldehyde **21** (50.6 mg, 0.191 mmol) in CH_2Cl_2 (1.9 mL) were added crude

amine **23** (59.2 mg, 0.229 mmol) and AcOH (13 μL, 0.229 mmol). After stirring for 10 min at room temperature, the reaction mixture was cooled to 0 °C. $NaBH(OAc)_3$ (96.9 mg, 0.457 mmol) was added to the reaction mixture. The reaction mixture was stirred at 0 °C for 30 min and at room temperature for 8 h. The mixture was diluted with CH_2Cl_2 (15 mL), washed with saturated $NaHCO_3$ aq. (5 mL), and dried with Na_2SO_4. After filtration and evaporation, the crude was purified by silica gel chromatography (*n*-hexane/EtOAc 1:1 to 1:2) to give compound **24** as a colorless oil (55.0 mg, 57%).

^{1}H NMR (500 MHz, Chloroform-*d*) δ 7.42 – 7.33 (m, 3H), 7.13 – 7.04 (m, 6H), 5.76 – 5.64 (m, 1H), 4.96 – 4.85 (m, 2H), 4.68 (tt, *J* = 12.2, 6.1 Hz, 1H), 3.00 (d, *J* = 11.6 Hz, 2H), 2.73 – 2.65 (m, 2H), 2.58 (t, *J* = 7.6 Hz, 2H), 2.55 – 2.48 (m, 2H), 2.34 – 2.25 (m, 2H), 2.24 – 2.11 (m, 4H), 2.00 (t, *J* = 7.5 Hz, 2H), 1.91 – 1.83 (m, 2H), 1.83 – 1.76 (m, 2H), 1.50-1.37 (m, 2H), 1.43 (s, 9H).

^{13}C NMR (126 MHz, Chloroform-*d*) δ 172.93, 171.96, 139.43, 138.74, 137.70, 137.66, 130.50, 129.40, 128.68, 128.59, 128.42, 114.97, 80.13, 60.58, 53.15, 52.28, 35.02, 34.78, 34.49, 33.40, 30.58, 29.49, 28.20, 26.86.

High resolution mass spectrometry (ESI) found 505.3432 [calculated for $C_{32}H_{45}N_2O_3$ (M + H^+) 505.3425].

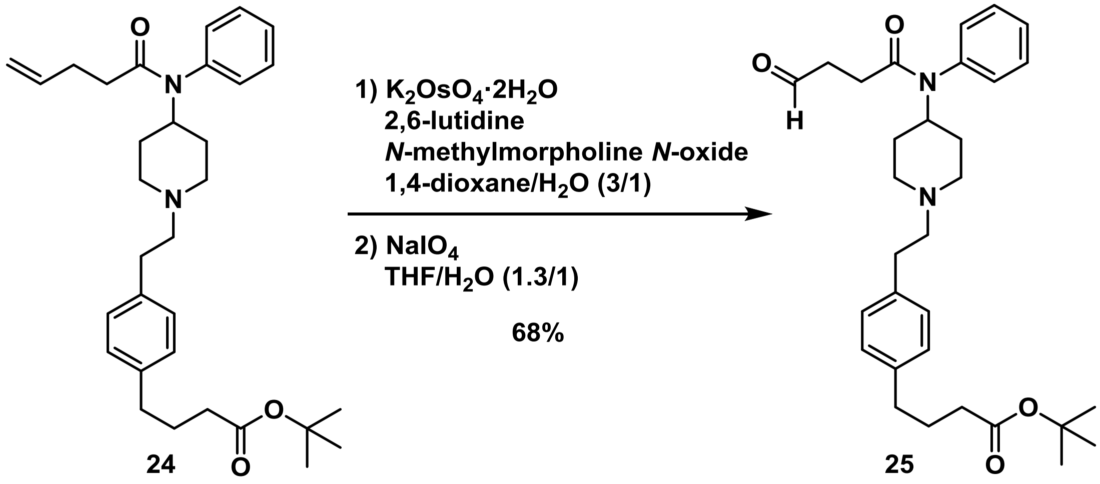

24 25

tert-Butyl 4-(4-(2-(4-(4-oxo-_N_-phenylbutanamido)piperidin-1-yl)ethyl)phenyl)butanoate (25)

To a solution of compound **24** (50.0 mg, 0.0991 mmol) in 1,4-dioxane/H_2O (3:1, 1.3 mL) were added 2,6-lutidine (34.2 μL, 0.297 mmol), K_2OsO_4·$2H_2O$ (0.37 mg, 0.991 μmol) and NMO (23.2 mg, 0.198 mmol), and the reaction was stirred at room temperature for 15 h. 10% aq. $Na_2S_2O_3$ (1

mL) was added to the reaction mixture and the mixture was stirred for 10 min. The whole mixture was extracted with CH_2Cl_2 (2 × 5 mL). The combined organic layers were dried with Na_2SO_4 and concentrated.

To a solution of the above crude product in THF/H_2O (1.3:1, 1.0 mL) was added $NaIO_4$ (42.4 mg, 0.198 mmol). The mixture was stirred for 15 h at room temperature. 10% aq. $Na_2S_2O_3$ (1 mL) was added to the reaction mixture and the mixture was stirred for 10 min. The whole mixture was extracted with CH_2Cl_2 (2 × 5 mL). The combined organic layers were dried with Na_2SO_4. After filtration and evaporation, the crude was purified by silica gel chromatography (CH_2Cl_2/MeOH 50:1 to 15:1) to give compound **25** as a pale brown oil (34.1 mg, 68%, 2 steps).

^{1}H NMR (400 MHz, Chloroform-*d*) δ 9.78 (s, 1H), 7.45 – 7.34 (m, 3H), 7.20 – 7.11 (m, 2H), 7.06 (s, 4H), 4.63 (tt, *J* = 12.2, 3.8 Hz, 1H), 3.01 (d, *J* = 11.9 Hz, 2H), 2.74 – 2.65 (m, 4H), 2.62 – 2.47 (m, 4H), 2.26 – 2.09 (m, 6H), 1.92 – 1.76 (m, 4H), 1.50-1.43 (m, 2H) 1.43 (s, 9H).

^{13}C NMR (126 MHz, Chloroform-*d*) δ 201.22, 173.02, 170.99, 139.50, 138.50, 137.72, 130.49, 129.61, 128.73, 128.69, 128.65, 80.21, 77.42, 77.16, 76.91, 60.57, 53.14, 52.70, 39.03, 35.09, 34.84, 33.41, 30.52, 28.25, 28.23, 26.92.

High resolution mass spectrometry (ESI) found 507.3216 [calculated for $C_{31}H_{43}N_2O_4$ (M + H^+) 507.3217].

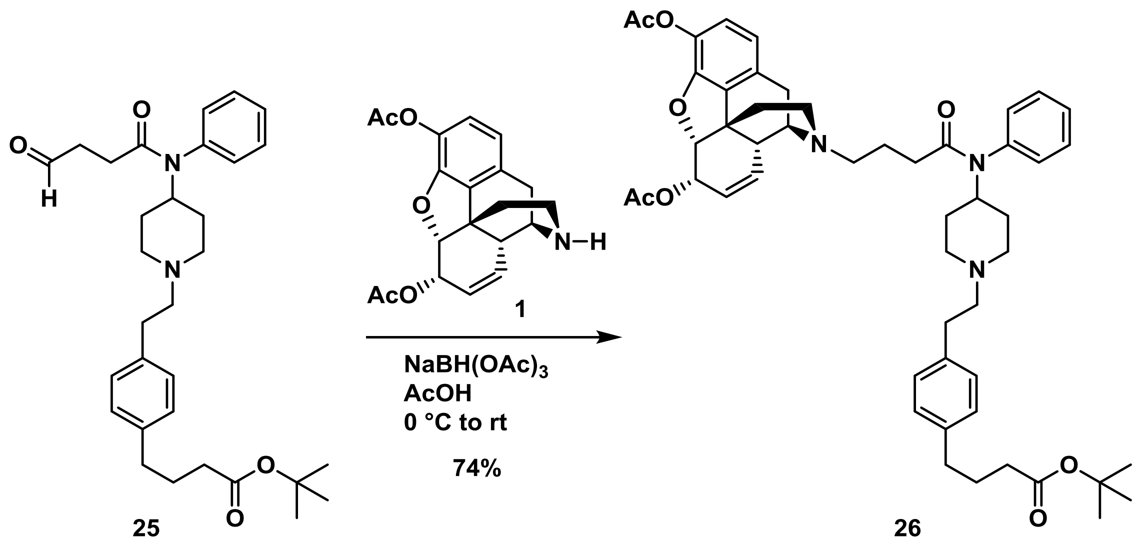

(4*R*,4a*R*,7*S*,7a*R*,12b*S*)-3-(4-((1-(4-(4-(*tert*-butoxy)-4-oxobutyl)phenethyl)piperidin-4-yl)(phenyl)amino)-4-oxobutyl)-2,3,4,4a,7,7a-hexahydro-1*H*-4,12-methanobenzofuro[3,2-*e*]isoquinoline-7,9-diyl diacetate (26, HF-4 *tert*-Bu ester)

To a solution of compound **25** (16.9 mg, 0.0334 mmol) in CH_2Cl_2 (0.7 mL) were added

norheroin **1** (13.0 mg, 0.0367 mmol) and AcOH (5 μL, 0.0834 mmol). After stirring for 10 min at room temperature, the reaction mixture was cooled to 0 °C. $NaBH(OAc)_3$ (17.7 mg, 0.0834 mmol) was added to the reaction mixture. The reaction mixture was stirred at 0 °C for 30 min and at room temperature for 25 h. The mixture was diluted with CH_2Cl_2 (15 mL), washed with saturated $NaHCO_3$ aq. (5 mL), and dried with Na_2SO_4. After filtration and evaporation, the crude was purified by silica gel chromatography (CH_2Cl_2/MeOH 19:1 to 9:1) to give compound **26** as a colorless oil (20.8 mg, 74%).

^{1}H NMR (500 MHz, Chloroform-*d*) δ 7.44 – 7.33 (m, 3H), 7.12 – 7.08 (m, 2H), 7.06 (s, 4H), 6.73 (d, *J* = 8.2 Hz, 1H), 6.53 (d, *J* = 8.2 Hz, 1H), 5.63 – 5.56 (m, 1H), 5.38 (dt, *J* = 10.1, 3.0 Hz, 1H), 5.12 (td, *J* = 5.0, 4.3, 2.5 Hz, 1H), 5.06 (dd, *J* = 6.6, 1.1 Hz, 1H), 4.68 (tt, *J* = 12.0, 4.0 Hz, 1H), 3.33 (dt, *J* = 6.0, 3.3 Hz, 1H), 3.01 (d, *J* = 11.6 Hz, 2H), 2.90 (d, *J* = 18.8 Hz, 1H), 2.73 – 2.66 (m, 2H), 2.62 – 2.10 (m, 14H), 2.26 (s, 3H), 2.13 (s, 3H), 2.03 – 1.68 (m, 10H), 1.50 – 1.38 (m, 2H), 1.43 (s, 9H).
^{13}C NMR (126 MHz, Chloroform-*d*) δ 173.00, 172.65, 170.64, 168.57, 149.48, 139.48, 138.90, 137.74, 132.49, 131.78, 131.71, 130.52, 129.88, 129.46, 128.72, 128.64, 128.51, 128.36, 121.93, 119.42, 88.93, 80.20, 77.42, 77.16, 76.91, 68.33, 60.60, 56.61, 54.17, 53.16, 52.33, 44.80, 43.53, 40.61, 35.28, 35.07, 34.83, 33.42, 33.01, 30.63, 29.81, 28.24, 26.90, 23.46, 21.82, 20.83, 20.77.
$[\alpha]_D^{22}$ = –105.9 (*c*. 0.54, MeOH)
High resolution mass spectrometry (ESI) found 846.4690 [calculated for $C_{51}H_{64}N_3O_8$ (M + H^+) 846.4688].

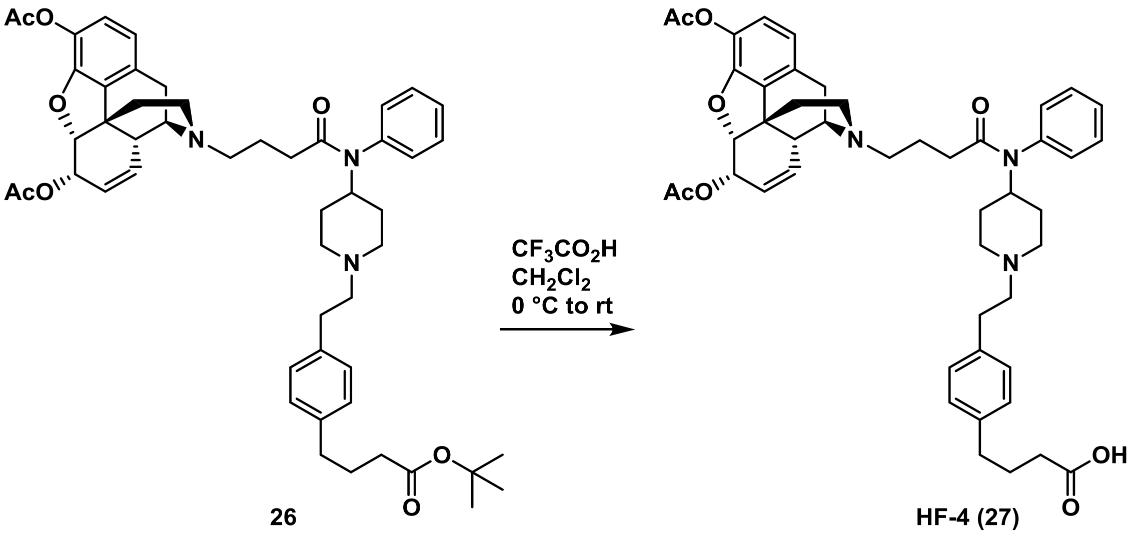

4-(4-(2-(4-(4-((4*R*,4a*R*,7*S*,7a*R*,12b*S*)-7,9-diacetoxy-1,2,4,4a,7,7a-hexahydro-3*H*-4,12-methanobenzofuro[3,2-*e*]isoquinolin-3-yl)-*N*-phenylbutanamido)piperidin-1-yl)ethyl)phenyl)butanoic acid (27, HF-4)

To a solution of compound **26** (10.0 mg, 0.0118 mmol) in CH_2Cl_2 (0.5 mL) was added TFA (0.5 mL). The reaction mixture was stirred at 0 °C for 30 min and at room temperature for 30 min. The solvents were removed under reduced pressure. Traces of TFA were removed from a reaction mixture by azeotropic distillations with toluene (2 × 0.5 mL) under reduced pressure. The residue was used as HF-4 (**27**)·TFA without further purification.

HF-5 Synthesis (compounds **28**–**31**)

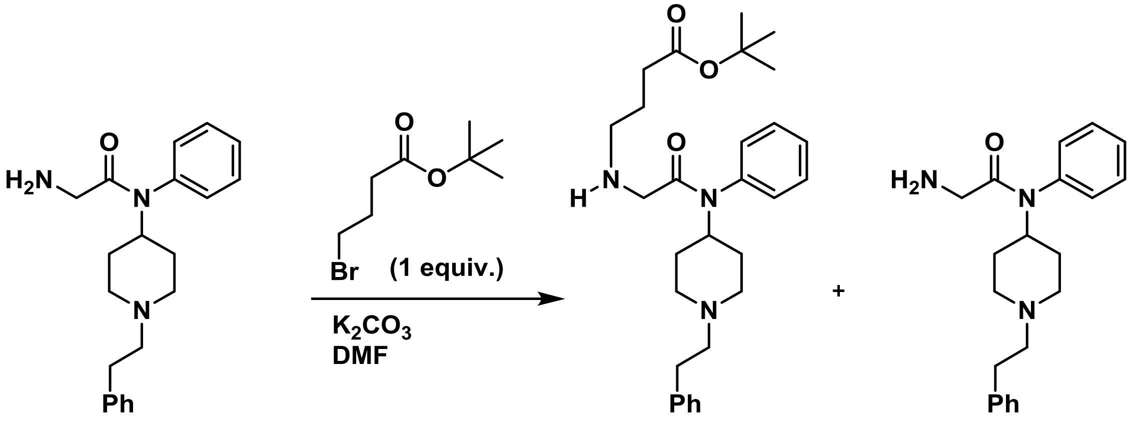

28 **29 (39%)** **28 (50%)**

Starting material (primary amine **28**) was prepared according known procedure. (*Bioorg. Med. Chem. Lett*. **2013**, *23*, 3434-3437.)

tert-Butyl 4-((2-oxo-2-((1-phenethylpiperidin-4-yl)(phenyl)amino)ethyl)amino)butanoate (29)

To a solution of primary amine **28** (42.8 mg, 0.127 mmol) in DMF (0.3 mL) was added a solution of *tert*-butyl 4-bromobutanoate (28.3 mg, 0.127 mmol) in DMF (1.0 mL) and K_2CO_3 (35.1 mg, 0.254 mmol). The reaction mixture was stirred at room temperature for 10 h. The mixture was diluted with CH_2Cl_2 (10 mL), washed with saturated $NaHCO_3$ aq. (3 mL), and dried with Na_2SO_4. After filtration and evaporation, the crude was purified by silica gel chromatography (CH_2Cl_2/MeOH 19:1 to 9:1) to give compound **29** as a colorless oil (23.6 mg, 39%) and starting material (21.3 mg, 50%)

^{1}H NMR (500 MHz, Chloroform-*d*) δ 7.45 – 7.34 (m, 3H), 7.25 (d, *J* = 7.6 Hz, 2H), 7.21 – 7.12 (m, 3H), 7.09 (dd, *J* = 7.7, 2.0 Hz, 2H), 4.67 (tt, *J* = 12.2, 3.9 Hz, 1H), 3.04 – 2.97 (m, 2H), 2.94 (s, 2H), 2.76 – 2.69 (m, 2H), 2.58 – 2.45 (m, 4H), 2.26 – 2.12 (m, 4H), 2.07 (brs, 1H), 1.85 – 1.77 (m, 2H), 1.73 – 1.63 (m, 2H), 1.52 – 1.41 (m, 2H), 1.41 (s, 9H).
^{13}C NMR (126 MHz, Chloroform-*d*) δ 172.96, 170.90, 140.28, 137.52, 130.42, 129.59, 128.82, 128.73, 128.49, 126.15, 80.15, 60.56, 53.12, 52.70, 51.89, 49.06, 33.93, 33.41, 30.53, 28.20, 25.56.
High resolution mass spectrometry (ESI) found 480.3226 [calculated for $C_{29}H_{42}N_3O_3$ (M + H^+) 480.3221].

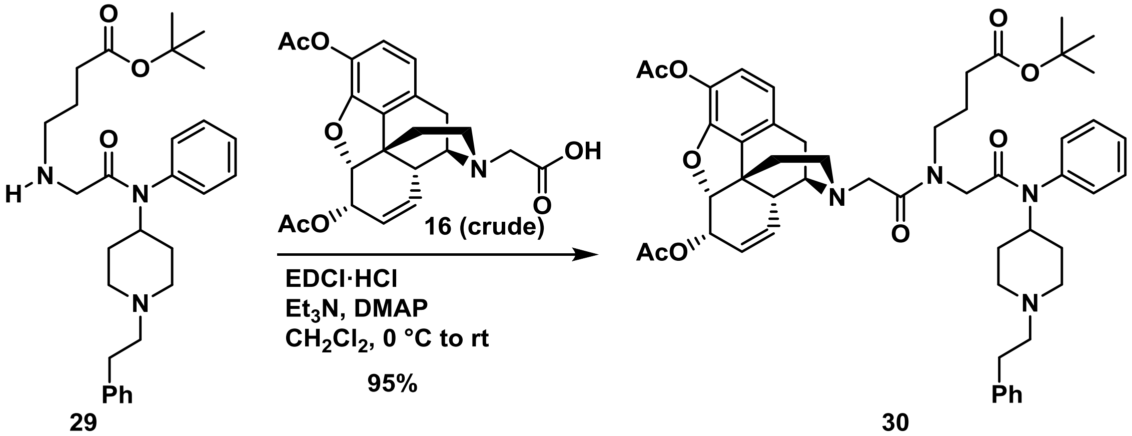

(4*R*,4a*R*,7*S*,7a*R*,12b*S*)-3-(2-((4-(*tert*-butoxy)-4-oxobutyl)(2-oxo-2-((1-phenethylpiperidin-4-yl)(phenyl)amino)ethyl)amino)-2-oxoethyl)-2,3,4,4a,7,7a-hexahydro-1*H*-4,12-methanobenzofuro[3,2-*e*]isoquinoline-7,9-diyl diacetate (30, HF-5 *tert*-Bu ester)

To a solution of compound **13** (44.0 mg, 0.0938 mmol) in CH_2Cl_2 (0.7 mL) was added TFA (0.7 mL). The reaction mixture was stirred at 0 °C for 30 min and room temperature for 4 h. The solvents were removed under reduced pressure. Traces of TFA were removed from a reaction mixture by azeotropic distillations with toluene (2 × 1 mL) under reduced pressure.

To a solution of the above crude product (**16**) in CH_2Cl_2 (0.6 mL) were added Et_3N (39 μL, 0.281 mmol), secondary amine **29** (30.0 mg, 0.0625 mmol) and DMAP (1 crop). After cooling to 0 °C, EDCI·HCl (18.0 mg, 0.0938 mmol) was added to the mixture. The reaction mixture was stirred at 0 °C for 30 min and at room temperature for 8 h. The mixture was diluted with CH_2Cl_2 (10 mL), washed with saturated $NaHCO_3$ aq. (3 mL), and dried with Na_2SO_4. After filtration and evaporation, the crude was purified by silica gel chromatography (CH_2Cl_2/MeOH 40:1 to 19:1) to give compound **30** as a colorless oil (52.0 mg, 95%).

Two rotamers were observed in NMR analysis.

^{1}H NMR (500 MHz, Chloroform-*d*) δ 7.49 – 7.35 (m, 3H), 7.25 – 7.19 (m, 3H), 7.19 – 7.08 (m, 4H), 6.80 – 6.72 (m, 1H), 6.60 – 6.54 (m, 1H), 5.66 – 5.56 (m, 1H), 5.46 – 5.38 (m, 1H), 5.16 – 5.03 (m, 2H), 4.68 – 4.56 (m, 1H), 4.06 – 3.84 (m, 1H), 3.71 – 3.50 (m, 3H), 3.50 – 3.30 (m, 3H), 3.30 – 3.17 (m, 1H), 3.07 – 2.95 (m, 3H), 2.82 – 2.64 (m, 3H), 2.58 – 2.32 (m, 5H), 2.29 – 2.01 (m, 11H), 1.93 – 1.74 (m, 5H), 1.74 – 1.64 (m, 1H), 1.47 – 1.35 (m, 9H).

^{13}C NMR (126 MHz, Chloroform-*d*) δ 172.66, 172.26, 170.59, 170.23, 169.97, 168.52, 167.96, 167.52, 149.52, 149.46, 140.30, 140.23, 137.62, 137.31, 132.36, 132.17, 131.88, 131.83, 131.58, 131.23, 130.56, 130.52, 130.28, 130.24, 130.07, 130.01, 129.82, 129.69, 129.48, 129.35, 128.85, 128.72, 128.63, 128.63, 128.47, 128.34, 126.16, 126.12, 122.16, 121.98, 119.59, 119.53, 88.88,

88.79, 80.73, 80.36, 68.27, 68.22, 60.54, 60.47, 59.03, 57.99, 56.74, 56.62, 53.23, 53.11, 53.01, 52.85, 50.54, 48.58, 48.33, 46.79, 45.34, 45.16, 43.14, 43.12, 40.66, 40.59, 35.26, 35.21, 33.92, 32.75, 32.31, 30.59, 30.48, 30.45, 28.23, 28.21, 23.89, 22.73, 21.94, 21.79, 20.81, 20.75.
$[\alpha]_D^{22}$ = -67.5 (*c*. 0.16, CH_3OH)
High resolution mass spectrometry (ESI) found 875.4570 [calculated for $C_{51}H_{63}N_4O_9$ (M + H^+) 875.4589].

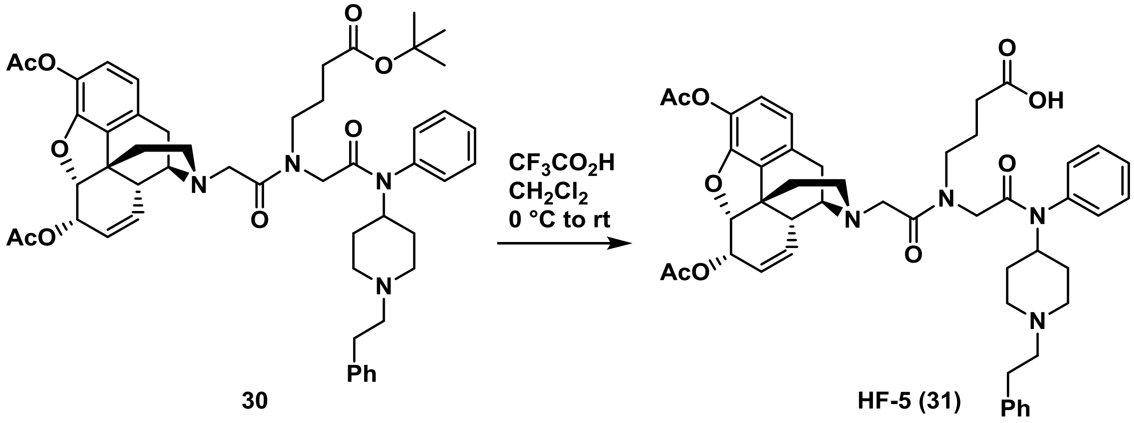

30 HF-5 (31)

4-(2-((4*R*,4a*R*,7*S*,7a*R*,12b*S*)-7,9-diacetoxy-1,2,4,4a,7,7a-hexahydro-3*H*-4,12-methanobenzofuro[3,2-*e*]isoquinolin-3-yl)-*N*-(2-oxo-2-((1-phenethylpiperidin-4-yl)(phenyl)amino)ethyl)acetamido)butanoic acid (31, HF-5)

To a solution of compound **30** (25.1 mg, 0.0287 mmol) in CH_2Cl_2 (0.5 mL) was added TFA (0.5 mL). The reaction mixture was stirred at 0 °C for 30 min and room temperature for 30 min. The solvents were removed under reduced pressure. Traces of TFA were removed from a reaction mixture by azeotropic distillations with toluene (2 × 0.5 mL) under reduced pressure. The residue was purified by silica gel chromatography (CH_2Cl_2/MeOH 9:1 to 3:1) to give HF-5 (**31**) as a colorless oil (20.3 mg, 86%).

Two rotamers were observed in NMR analysis.
^{1}H NMR (600 MHz, Methanol-*d*$_4$) δ 7.60 – 7.47 (m, 3H), 7.40 – 7.34 (m, 2H), 7.33 – 7.26 (m, 2H), 7.26 – 7.19 (m, 3H), 6.77 – 6.72 (m, 1H), 6.65 – 6.60 (m, 1H), 5.66 – 5.56 (m, 1H), 5.56 – 5.44 (m, 1H), 5.18 – 5.03 (m, 2H), 4.80 – 4.70 (m, 1H), 4.29 – 3.92 (m, 1H), 3.79 – 3.23 (m, 6H), 3.21 – 2.88 (m, 8H), 2.83 – 2.57 (m, 2H), 2.53 – 2.38 (m, 2H), 2.31 – 2.06 (m, 11H), 2.05 – 1.96 (m, 1H), 1.90 – 1.65 (m, 5H).
^{13}C NMR (151 MHz, Methanol-*d*$_4$) δ 172.54, 172.16, 172.11, 172.08, 170.27, 169.95, 169.68, 150.83, 150.64, 138.28, 138.19, 138.05, 137.99, 133.52, 133.12, 133.10, 132.65, 132.49, 131.42,

131.37, 131.09, 130.83, 130.58, 130.33, 130.31, 129.86, 129.85, 129.77, 129.74, 129.73, 129.57, 128.07, 128.05, 123.10, 123.00, 120.55, 90.15, 90.04, 69.66, 69.50, 64.34, 59.50, 59.16, 59.06, 58.90, 58.75, 58.05, 53.19, 53.15, 52.53, 52.04, 51.87, 50.18, 49.94, 48.35, 46.24, 45.95, 44.19, 43.98, 41.57, 40.89, 35.96, 35.62, 31.86, 31.78, 29.14, 29.00, 25.59, 24.20, 23.21, 22.97, 20.67, 20.62, 20.45.

High resolution mass spectrometry (ESI) found 819.3970 [calculated for $C_{47}H_{55}N_4O_9$ $(M + H^+)$ 819.3963].

HF-6 Synthesis (compounds **32**–**38**)

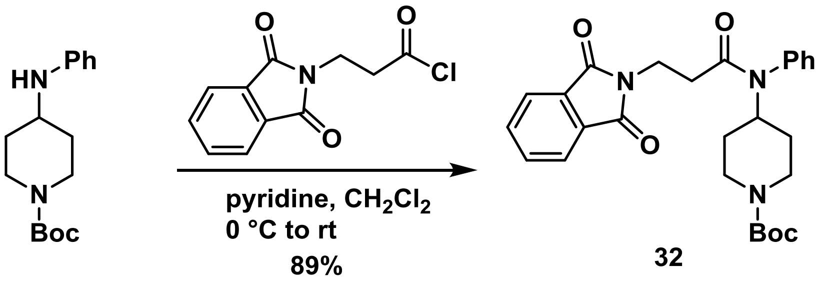

tert-Butyl 4-(3-(1,3-dioxoisoindolin-2-yl)-_N_-phenylpropanamido)piperidine-1-carboxylate (32)

To a solution of 3-(1,3-dioxoisoindolin-2-yl)propanoic acid (256 mg, 1.17 mmol) in CH_2Cl_2 (5.9 mL) were added $(COCl)_2$ (200 μL, 2.33 mmol) and DMF (1 drop) at 0 °C.

The reaction mixture was stirred at 0 °C for 30 min and at room temperature for 3 h. The solvents were removed under reduced pressure. Azeotropic distillations were carried out with toluene (2 × 3 mL) under reduced pressure. The residue (acid chloride) was used for next step without further purification.

To a solution of 1-*N*-Boc-4-(phenylamino)piperidine (71.6 mg, 0.259 mmol) and pyridine (188 μL, 2.33 mmol) in CH_2Cl_2 (1.6 mL) was added a solution of the above crude product (acid chloride) in CH_2Cl_2 (1.0 mL) at 0 °C. The reaction mixture was stirred at 0 °C for 30 min and at room temperature for 3 h. After cooling to 0 °C, the reaction mixture was quenched by addition of saturated $NaHCO_3$ aq. (5 mL). The whole mixture was extracted with CH_2Cl_2 (2 × 20 mL), and the combined organic layers were dried with Na_2SO_4. After filtration and evaporation, the crude was purified by silica gel chromatography (*n*-hexane/EtOAc 1:1 to 1:2) to give compound **32** as a white solid (110 mg, 89%).

^{1}H NMR (500 MHz, Chloroform-*d*) δ 7.81 – 7.74 (m, 2H), 7.69 – 7.63 (m, 2H), 7.37 (d, *J* = 7.0 Hz, 3H), 7.07 (d, *J* = 6.6 Hz, 2H), 4.71 (t, *J* = 12.2 Hz, 1H), 4.19 – 3.98 (m, 2H), 3.89 (t, *J* = 7.4 Hz, 2H), 2.75 (t, *J* = 13.2 Hz, 2H), 2.30 (t, *J* = 7.4 Hz, 2H), 1.74 (d, *J* = 11.9 Hz, 2H), 1.36 (s, 9H), 1.27 – 1.13 (m, 2H).

^{13}C NMR (126 MHz, Chloroform-*d*) δ 169.68, 168.17, 154.60, 138.19, 133.96, 132.18, 130.32, 129.69, 128.81, 123.28, 79.62, 52.39, 43.18, 34.45, 33.54, 30.42, 28.44.

High resolution mass spectrometry (ESI) found 478.2331 [calculated for $C_{27}H_{32}N_3O_5$ (M + H^+) 478.2336].

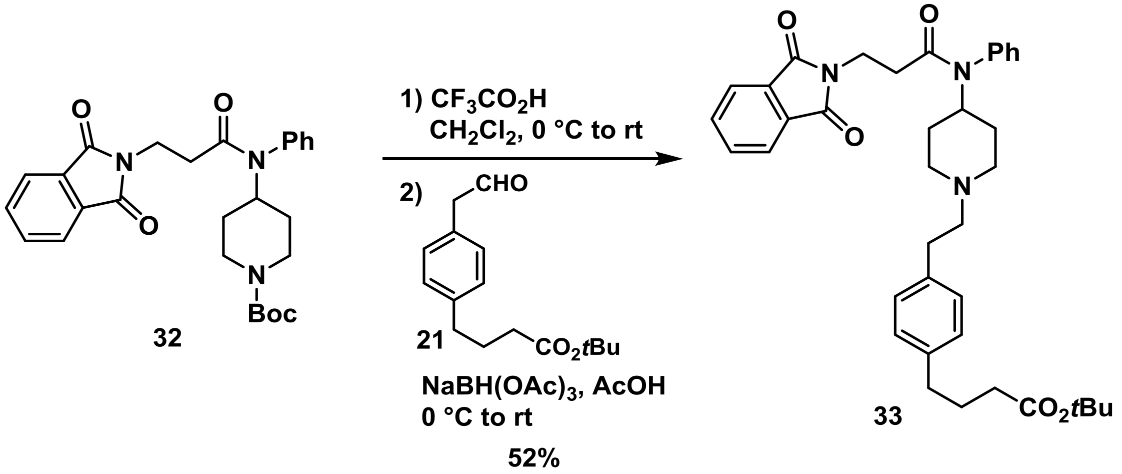

tert-Butyl 4-(4-(2-(4-(3-(1,3-dioxoisoindolin-2-yl)-*N*-phenylpropanamido)piperidin-1-yl)ethyl)phenyl) butanoate (33)

A solution of compound **32** (90.0 mg, 0.188 mmol) in CH_2Cl_2 (1.0 mL) was added TFA/CH_2Cl_2 (1/1, 1.0 mL) at 0 °C. The reaction mixture was stirred at 0 °C for 30 min and at room temperature for 7 h. The solvents were removed under reduced pressure. Traces of TFA were removed from a reaction mixture by azeotropic distillations with toluene (2 × 1 mL) under reduced pressure. The residue was dissolved in CH_2Cl_2 (1.9 mL). Aldehyde **21** (74.0 mg, 0.282 mmol) and AcOH (22 μL, 0.188 mmol) were added to the solution. After stirring for 5 min at room temperature, the reaction mixture was cooled to 0 °C. NaBH(OAc)$_3$ (80.0 mg, 0.376 mmol) was added to the reaction mixture. The reaction mixture was stirred at 0 °C for 30 min and room temperature for 4 h. The mixture was diluted with CH_2Cl_2 (20 mL), washed with saturated $NaHCO_3$ aq. (5 mL), and dried with Na_2SO_4. After filtration and evaporation, the crude was purified by silica gel chromatography (*n*-hexane/EtOAc 1:1 to 0:1) to give compound **33** as a colorless oil (61.1 mg, 52%).

^{1}H NMR (500 MHz, Chloroform-*d*) δ 7.79 (dd, *J* = 5.5, 3.1 Hz, 2H), 7.67 (dd, *J* = 5.5, 3.1 Hz, 2H), 7.40 – 7.31 (m, 3H), 7.13 – 7.09 (m, 2H), 7.06 (s, 4H), 4.64 (tt, *J* = 12.4, 4.1 Hz, 1H), 3.91 (t, *J* = 7.5 Hz, 2H), 2.98 (d, *J* = 11.7 Hz, 2H), 2.72 – 2.64 (m, 2H), 2.61 – 2.54 (m, 2H), 2.54 – 2.47 (m, 2H), 2.32 (t, *J* = 7.5 Hz, 2H), 2.21 (t, *J* = 7.5 Hz, 2H), 2.18 – 2.09 (m, 2H), 1.91 – 1.83 (m, 2H), 1.80 (d, *J* = 12.4 Hz, 2H), 1.48-1.38 (m, 2H), 1.43 (s, 9H).

^{13}C NMR (126 MHz, Chloroform-*d*) δ 173.01, 169.72, 168.22, 139.47, 138.30, 137.82, 133.97, 132.26, 130.53, 129.62, 128.73, 128.67, 128.64, 123.30, 80.21, 60.65, 53.15, 52.35, 35.09, 34.84, 34.54, 33.64, 33.50, 30.52, 28.25, 26.91.

High resolution mass spectrometry (ESI) found 624.3441 [calculated for $C_{38}H_{46}N_3O_5$ (M + H^+) 624.3432].

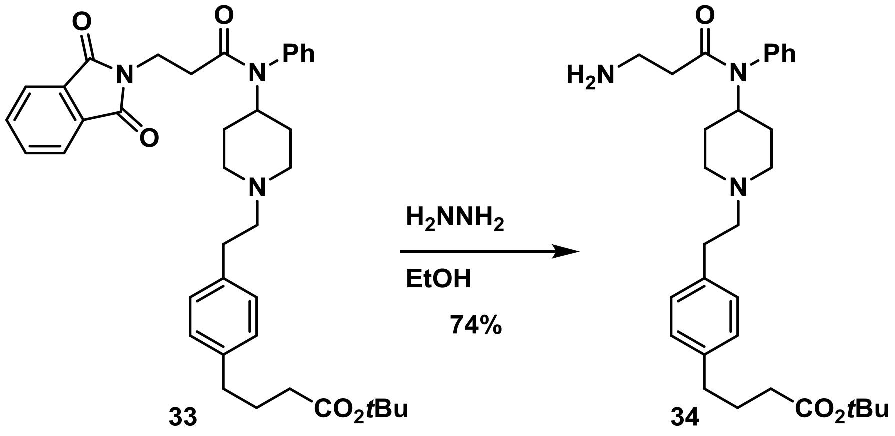

***tert*-Butyl**
4-(4-(2-(4-(3-amino-*N*-phenylpropanamido)piperidin-1-yl)ethyl)phenyl)butanoate (34)

A solution of compound **33** (280 mg, 0.449 mmol) in EtOH (9.0 mL) was added hydrazine (72 μL, 2.24 mmol) at 0 °C. The reaction mixture was stirred at 0 °C for 30 min and at room temperature for 12 h. After stirring for 12 h, the reaction mixture was filtered through a pad of Celite and the filter cake was washed with EtOH. After evaporation, the residue was purified by silica gel chromatography (CH_2Cl_2/MeOH 5:1 to 1:1) to give compound **34** as a pale yellow oil (164 mg, 74%).

^{1}H NMR (500 MHz, Chloroform-*d*) δ 7.43 – 7.33 (m, 3H), 7.14 (d, *J* = 7.0 Hz, 2H), 7.07 (s, 4H), 4.61 (s, 1H), 3.21 – 2.99 (m, 4H), 2.82 – 2.69 (m, 2H), 2.67 – 2.54 (m, 4H), 2.45 – 2.25 (m, 4H), 2.20 (t, *J* = 7.5 Hz, 2H), 1.91 – 1.76 (m, 4H), 1.66 – 1.49 (m, 2H), 1.43 (s, 9H).

^{13}C NMR (126 MHz, Chloroform-*d*) δ 172.99, 171.18, 139.74, 137.44, 137.05, 130.17, 129.98, 129.19, 128.75, 80.23, 60.14, 52.83, 52.60, 36.73, 35.08, 34.84, 32.92, 31.82, 29.93, 28.26, 26.91.

High resolution mass spectrometry (ESI) found 494.3391 [calculated for $C_{30}H_{44}N_3O_3$ (M + H^+) 494.3377].

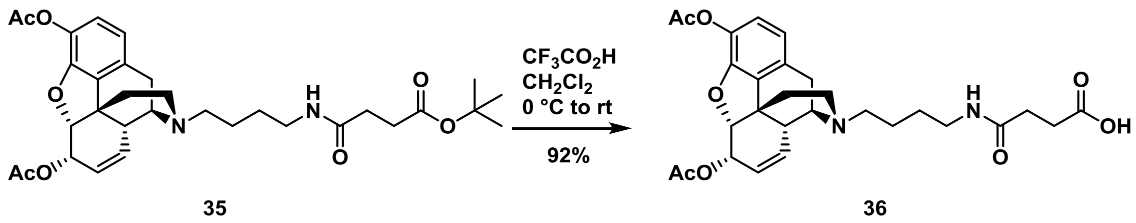

tert-Butyl ester (**35**) was prepared according known procedure.
(*J. Am. Chem. Soc.* **2017**, *139*, 8601-8611.)

4-((4-((4*R*,4a*R*,7*S*,7a*R*,12b*S*)-7,9-Diacetoxy-1,2,4,4a,7,7a-hexahydro-3*H*-4,12-methanobenzo furo[3,2-*e*]isoquinolin-3-yl)butyl)amino)-4-oxobutanoic acid (36)

To a solution of *tert*-butyl ester **35** (141 mg, 0.242 mmol) in CH_2Cl_2 (1.0 mL) was added TFA (1.0 mL). The reaction mixture was stirred at 0 °C for 30 min and at room temperature for 30 min. The solvents were removed under reduced pressure. Traces of TFA were removed from a reaction mixture by azeotropic distillations with toluene (2 × 1 mL) under reduced pressure. The residue was purified by silica gel chromatography (CH_2Cl_2/MeOH 9:1 to 5:1) to give compound **36** as a colorless oil (117 mg, 92%).

^{1}H NMR (500 MHz, Methanol-d_4) δ 6.87 (d, *J* = 8.2 Hz, 1H), 6.76 (d, *J* = 8.2 Hz, 1H), 5.75 (dd, *J* = 10.2, 1.6 Hz, 1H), 5.54 (d, *J* = 10.4 Hz, 1H), 5.23 (s, 2H), 4.32 (s, 1H), 3.45 (dd, *J* = 13.5, 5.2 Hz, 1H), 3.42 – 3.11 (m, 5H), 3.06 – 2.93 (m, 2H), 2.61 (t, *J* = 7.0 Hz, 2H), 2.48 (t, *J* = 6.8 Hz, 2H), 2.43-2.37 (m, 1H), 2.26 (s, 3H), 2.15 – 2.07 (m, 1H), 2.11 (s, 3H), 1.90 – 1.77 (m, 2H), 1.68 – 1.59 (m, 2H), 1.31 (t, *J* = 7.3 Hz, 2H).
^{13}C NMR (126 MHz, Methanol-d_4) δ 175.02, 171.91, 170.06, 150.86, 133.86, 131.48, 130.84, 130.25, 127.01, 124.33, 121.15, 89.04, 68.66, 59.97, 54.88, 47.87, 47.06, 42.90, 38.90, 32.82, 31.55, 30.36, 27.75, 22.48, 20.50, 20.38.
$[\alpha]_D^{22}$ = -69.6 (*c*. 0.59, CH_3OH)
High resolution mass spectrometry (ESI) found 527.2398 [calculated for $C_{28}H_{35}N_2O_8$ (M + H^+) 527.2388].

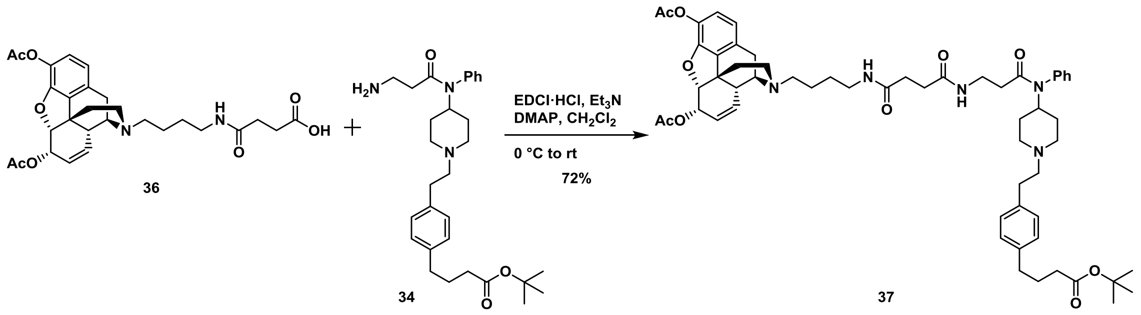

(4*R*,4a*R*,7*S*,7a*R*,12b*S*)-3-(4-(4-((3-((1-(4-(4-(*tert*-butoxy)-4-oxobutyl)phenethyl)piperidin-4-yl)(phenyl)amino)-3-oxopropyl)amino)-4-oxobutanamido)butyl)-2,3,4,4a,7,7a-hexahydro-1*H*-4,12-methanobenzofuro[3,2-*e*]isoquinoline-7,9-diyl diacetate (37, HF-6 *tert*-Bu ester)

To a solution of the heroin derivative **36** (15.1 mg, 0.0286 mmol) and fentanyl derivative **34** (14.1 mg, 0.0286 mmol) in DMF (0.6 mL) was added Et_3N (12 μL, 0.0858 mmol). After cooling to 0 °C, DMT-MM (23.7 mg, 0.0858 mmol) was added to the mixture. The reaction mixture was stirred at 0 °C for 30 min and at room temperature for 8 h. The mixture was diluted with CH_2Cl_2 (10 mL), washed with saturated $NaHCO_3$ aq. (3 mL), and dried with Na_2SO_4. After filtration and evaporation, the crude was purified by silica gel chromatography (CH_2Cl_2/MeOH 9:1 to 5:1) to give compound **37** as a pale yellow oil (20.5 mg, 72%).

^{1}H NMR (500 MHz, Chloroform-*d*) δ 7.37 (t, *J* = 7.7 Hz, 3H), 7.06 (s, 4H), 6.75 (d, *J* = 8.1 Hz, 1H), 6.62 (t, *J* = 6.2 Hz, 1H), 6.56 (d, *J* = 8.2 Hz, 1H), 6.44 (s, 1H), 5.61 (d, *J* = 9.9 Hz, 1H), 5.41 (d, *J* = 9.9 Hz, 1H), 5.18 – 5.07 (m, 2H), 4.62 (tt, *J* = 16.0, 12.0, 3.8 Hz, 1H), 3.48 (s, 1H), 3.39 (q, *J* = 5.8 Hz, 2H), 3.25 (q, *J* = 6.6 Hz, 2H), 3.04 (d, *J* = 11.9 Hz, 2H), 2.97 (d, *J* = 18.6 Hz, 1H), 2.86 – 2.64 (m, 5H), 2.62 – 2.51 (m, 6H), 2.51 – 2.42 (m, 4H), 2.42 – 2.30 (m, 3H), 2.26 (s, 3H), 2.24 – 2.14 (m, 4H), 2.12 (s, 3H), 2.11 – 1.98 (m, 3H), 1.94 – 1.75 (m, 5H), 1.52 – 1.35 (m, 2H), 1.43 (s, 9H), 1.29 – 1.19 (m, 2H).

^{13}C NMR (126 MHz, Chloroform-*d*) δ 173.00, 172.28, 171.98, 171.63, 170.57, 168.56, 149.52, 139.58, 138.07, 137.51, 132.02, 131.94, 131.53, 130.34, 129.71, 129.43, 128.83, 128.71, 128.68, 122.12, 119.48, 88.78, 80.22, 68.18, 60.49, 57.01, 54.34, 53.10, 52.49, 45.00, 43.37, 40.35, 39.30, 35.33, 35.10, 35.08, 34.83, 33.30, 31.91, 31.88, 30.41, 29.82, 28.24, 27.44, 26.90, 24.73, 21.79, 20.80, 20.77.

$[\alpha]_D^{22}$ = -44.3 (*c*. 0.44, CH_3OH)

High resolution mass spectrometry (ESI) found 1002.5590 [calculated for $C_{58}H_{76}N_5O_{10}$ (M + H^+) 1002.5586].

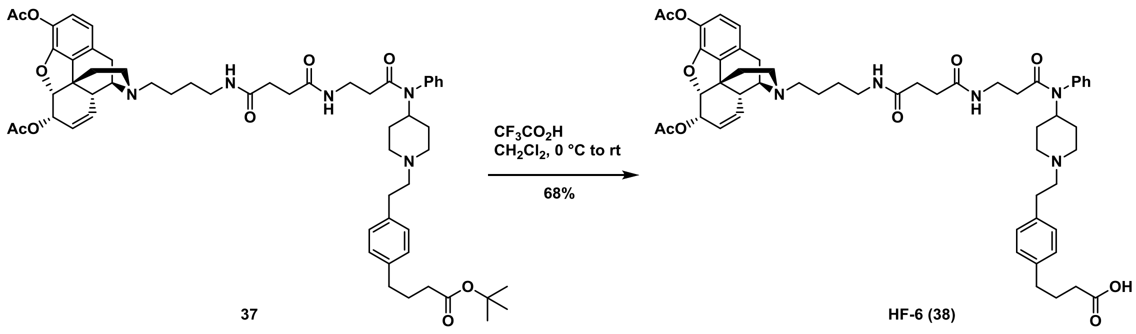

4-(4-(2-(4-(3-(4-((4-((4*R*,4a*R*,7*S*,7a*R*,12b*S*)-7,9-diacetoxy-1,2,4,4a,7,7a-hexahydro-3*H*-4,12-methanobenzofuro[3,2-*e*]isoquinolin-3-yl)butyl)amino)-4-oxobutanamido)-*N*-phenylpropanamido)piperidin-1-yl)ethyl)phenyl)butanoic acid (38, HF-6)

To a solution of compound **37** (20.5 mg, 0.205 mmol) in CH_2Cl_2 (0.5 mL) was added TFA (0.5 mL) at 0 °C. The reaction mixture was stirred at 0 °C for 30 min and at room temperature for 30 min. The solvents were removed under reduced pressure. Traces of TFA were removed from a reaction mixture by azeotropic distillations with toluene (2 × 0.5 mL) under reduced pressure. The residue was purified by silica gel chromatography (CH_2Cl_2/MeOH 9:1 to 5:1) to give HF-6 (**38**) as a colorless oil (13.2 mg, 68%).

^{1}H NMR (600 MHz, Methanol-d_4) δ 7.51 – 7.41 (m, 3H), 7.21 (d, *J* = 6.9 Hz, 2H), 7.09 (q, *J* = 8.2 Hz, 4H), 6.74 (d, *J* = 8.2 Hz, 1H), 6.62 (d, *J* = 8.1 Hz, 1H), 5.61 (dt, *J* = 10.1, 2.0 Hz, 1H), 5.50 (dt, *J* = 10.1, 2.6 Hz, 1H), 5.16 (ddd, *J* = 6.6, 5.0, 3.0 Hz, 1H), 5.13 – 5.08 (m, 1H), 4.64 (tt, *J* = 12.2, 4.0 Hz, 1H), 3.62 (dd, *J* = 6.3, 3.3 Hz, 1H), 3.36 – 3.32 (m, 2H), 3.25 – 3.14 (m, 4H), 3.07 (d, *J* = 19.0 Hz, 1H), 2.88 – 2.67 (m, 7H), 2.67 – 2.61 (m, 1H), 2.58 (t, *J* = 7.3 Hz, 2H), 2.51 – 2.36 (m, 8H), 2.24 (s, 3H), 2.18 – 2.11 (m, 3H), 2.10 (s, 3H), 1.94 – 1.78 (m, 5H), 1.65 – 1.45 (m, 6H), 1.37 – 1.23 (m, 2H).

^{13}C NMR (151 MHz, Methanol-d_4) δ 174.53, 174.41, 173.85, 172.71, 172.06, 170.22, 150.70, 141.69, 139.42, 137.49, 133.42, 133.17, 132.57, 131.53, 130.78, 130.15, 130.04, 129.80, 129.75, 129.58, 123.10, 120.51, 89.98, 69.47, 64.42, 60.70, 58.25, 55.39, 53.72, 53.15, 46.19, 44.28, 40.75, 40.00, 36.71, 36.24, 35.83, 35.54, 33.02, 32.22, 32.17, 30.77, 30.49, 28.31, 25.19, 22.53, 20.62, 20.44.

High resolution mass spectrometry (ESI) found 946.4947 [calculated for $C_{54}H_{68}N_5O_{10}$ (M + H^+) 946.4960].

HF-7 Synthesis (compounds **39**–**42**)

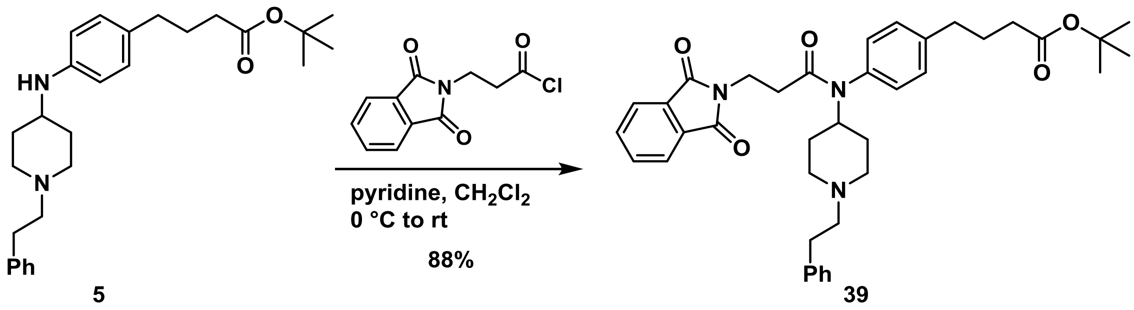

tert-Butyl 4-(4-(3-(1,3-dioxoisoindolin-2-yl)-_N_-(1-phenethylpiperidin-4-yl)propanamido)phenyl)butanoate (39)

To a solution of 3-(1,3-dioxoisoindolin-2-yl)propanoic acid (281 mg, 1.28 mmol) in CH_2Cl_2 (12.8 mL) were added $(COCl)_2$ (328 μL, 3.83 mmol) and DMF (1 drop) at 0 °C.

The reaction mixture was stirred at 0 °C for 30 min and at room temperature for 2 h. The solvents were removed under reduced pressure. Azeotropic distillations were carried out with toluene (2 × 3 mL) under reduced pressure. The residue (acid chloride) was used for next step without further purification.

To a solution of compound **5** (270 mg, 0.639 mmol) and pyridine (309 μL, 3.83 mmol) in CH_2Cl_2 (3.2 mL) was added a solution of the above crude product (acid chloride) in CH_2Cl_2 (3.2 mL) at 0 °C. The reaction mixture was stirred at 0 °C for 30 min and at room temperature for 2 h. After cooling to 0 °C, the reaction mixture was quenched by addition of saturated $NaHCO_3$ aq. (5 mL). The whole mixture was extracted with CH_2Cl_2 (2 × 20 mL), and the combined organic layers were dried with Na_2SO_4. After filtration and evaporation, the crude was purified by silica gel chromatography (*n*-hexane/EtOAc 1:1 to 0:1) to give compound **39** as a white solid (350 mg, 88%).

^{1}H NMR (500 MHz, Chloroform-*d*) δ 7.74 (dd, *J* = 5.4, 3.0 Hz, 2H), 7.62 (dd, *J* = 5.5, 3.1 Hz, 2H), 7.20 (t, *J* = 7.3 Hz, 2H), 7.16 – 7.07 (m, 5H), 6.97 (d, *J* = 8.2 Hz, 2H), 4.60 (tt, *J* = 12.2, 4.0 Hz, 1H), 3.87 (t, *J* = 7.5 Hz, 2H), 2.97 (d, *J* = 11.3 Hz, 2H), 2.72 – 2.65 (m, 2H), 2.62 – 2.55 (m, 2H), 2.53 – 2.46 (m, 2H), 2.29 (t, *J* = 7.5 Hz, 2H), 2.21 (t, *J* = 7.4 Hz, 2H), 2.11 (t, *J* = 11.3 Hz, 2H), 1.90 – 1.81 (m, 2H), 1.75 (d, *J* = 11.5 Hz, 2H), 1.45 – 1.33 (m, 2H), 1.42 (s, 9H).

^{13}C NMR (126 MHz, Chloroform-*d*) δ 172.59, 169.70, 168.02, 142.26, 140.06, 135.86, 133.79, 132.10, 130.18, 129.45, 128.58, 128.50, 128.33, 126.00, 123.12, 80.18, 60.33, 52.92, 52.04, 34.87, 34.62, 34.40, 33.65, 33.39, 30.23, 28.11, 26.61.
High resolution mass spectrometry (ESI) found 624.3442 [calculated for $C_{38}H_{46}N_3O_5$ (M + H^+) 624.3432].

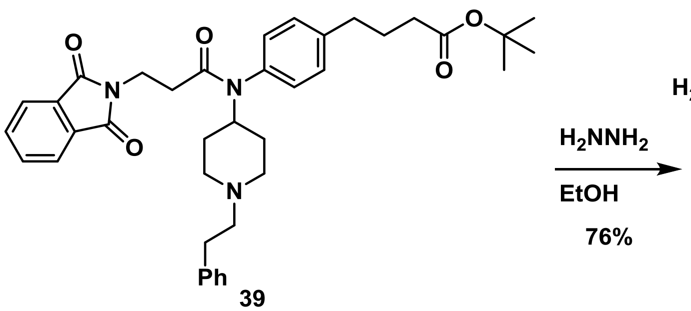

39 40

***tert*-Butyl 4-(4-(3-amino-*N*-(1-phenethylpiperidin-4-yl)propanamido)phenyl)butanoate (40)**
A solution of compound **39** (200 mg, 0.321 mmol) in EtOH (6.4 mL) was added hydrazine (51 μL, 1.60 mmol) at 0 °C. The reaction mixture was stirred at 0 °C for 30 min and at room temperature for 12 h. After stirring for 12 h, the reaction mixture was filtered through a pad of Celite and the filter cake was washed with EtOH. After evaporation, the residue was purified by silica gel chromatography (CH_2Cl_2/MeOH 5:1 to 0:1) to give compound **40** as a colorless oil (120 mg, 76%).

^{1}H NMR (600 MHz, Chloroform-*d*) δ 7.27 – 7.13 (m, 7H), 7.03 (d, *J* = 8.3 Hz, 2H), 4.61 – 4.54 (m, 1H), 3.21 – 3.04 (m, 4H), 2.83 – 2.74 (m, 2H), 2.71 – 2.55 (m, 4H), 2.43 – 2.30 (m, 4H), 2.26 (t, *J* = 7.4 Hz, 2H), 1.93 – 1.80 (m, 4H), 1.63 – 1.51 (m, 2H), 1.47 (s, 9H).
^{13}C NMR (151 MHz, Chloroform-*d*) δ 172.77, 171.41, 143.10, 139.39, 135.18, 129.99, 129.93, 128.78, 128.64, 126.44, 80.47, 60.00, 52.77, 52.57, 36.73, 35.10, 34.82, 33.21, 31.60, 29.76, 28.30, 26.76.
High resolution mass spectrometry (ESI) found 494.3391 [calculated for $C_{30}H_{44}N_3O_3$ (M + H^+) 494.3377].

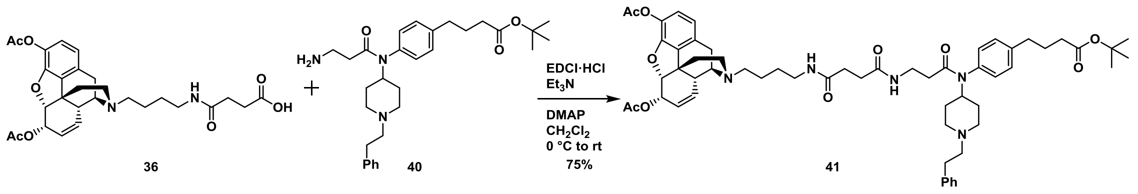

(4*R*,4a*R*,7*S*,7a*R*,12b*S*)-3-(4-(4-((3-((4-(4-(*tert*-butoxy)-4-oxobutyl)phenyl)(1-phenethylpiperidin-4-yl)amino)-3-oxopropyl)amino)-4-oxobutanamido)butyl)-2,3,4,4a,7,7a-hexahydro-1*H*-4,12-methanobenzofuro[3,2-*e*]isoquinoline-7,9-diyl diacetate (41, HF-7 *tert*-Bu ester)

To a solution of the heroin derivative **36** (33.0 mg, 0.0627 mmol) and fentanyl derivative **40** (31.0 mg, 0.0627 mmol) in DMF (1.3 mL) was added Et_3N (26 µL, 0.188 mmol). After cooling to 0 °C, DMT-MM (52.0 mg, 0.188 mmol) was added to the mixture. The reaction mixture was stirred at 0 °C for 30 min and at room temperature for 8 h. The mixture was diluted with CH_2Cl_2 (20 mL), washed with saturated $NaHCO_3$ aq. (5 mL), and dried with Na_2SO_4. After filtration and evaporation, the crude was purified by silica gel chromatography (CH_2Cl_2/MeOH 9:1 to 5:1) to give compound **41** as a pale, yellow oil (47.3 mg, 75%).

^{1}H NMR (600 MHz, Chloroform-*d*) δ 7.24 (d, *J* = 7.6 Hz, 2H), 7.21 – 7.12 (m, 5H), 6.96 (d, *J* = 8.2 Hz, 2H), 6.75 (d, *J* = 8.1 Hz, 1H), 6.61 (t, *J* = 6.0 Hz, 1H), 6.56 (d, *J* = 8.2 Hz, 1H), 6.42 (s, 1H), 5.60 (d, *J* = 10.0 Hz, 1H), 5.41 (d, *J* = 10.0 Hz, 1H), 5.17 – 5.07 (m, 2H), 4.60 (tt, *J* = 12.2, 4.0 Hz, 1H), 3.57 – 3.31 (m, 3H), 3.31 – 3.15 (m, 2H), 3.10 – 2.88 (m, 3H), 2.84 – 2.60 (m, 6H), 2.60 – 2.41 (m, 8H), 2.41 – 2.30 (m, 2H), 2.30 – 2.21 (m, 2H), 2.26 (s, 3H), 2.21 – 2.14 (m, 2H), 2.12 (s, 3H), 2.10 – 1.98 (m, 3H), 1.95 – 1.83 (m, 3H), 1.79 (d, *J* = 12.3 Hz, 2H), 1.61 – 1.50 (m, 2H), 1.50 – 1.38 (m, 11H), 1.33 – 1.17 (m, 2H).

^{13}C NMR (151 MHz, Chloroform-*d*) δ 172.76, 172.24, 171.93, 171.76, 170.55, 168.53, 149.50, 142.60, 140.10, 135.82, 132.11, 131.90, 131.56, 130.19, 129.67, 129.49, 128.72, 128.64, 128.52, 126.21, 122.08, 119.45, 88.79, 80.43, 68.18, 60.49, 57.00, 54.36, 53.11, 52.43, 44.97, 43.39, 40.43, 39.33, 35.32, 35.03, 35.02, 34.78, 33.81, 32.03, 31.91, 31.88, 30.46, 29.80, 29.46, 28.27, 27.47, 26.79, 24.83, 21.77, 20.79, 20.75.

$[\alpha]_D^{22}$ = -30.8 (*c*. 0.59, CH_3OH)

High resolution mass spectrometry (ESI) found 1002.5553 [calculated for $C_{58}H_{76}N_5O_{10}$ (M + H^+) 1002.5586].

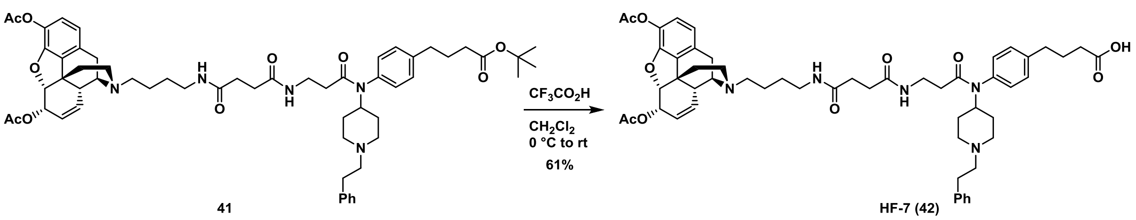

4-(4-(3-(4-((4-((4*R*,4a*R*,7*S*,7a*R*,12b*S*)-7,9-diacetoxy-1,2,4,4a,7,7a-hexahydro-3*H*-4,12-methanobenzofuro[3,2-*e*]isoquinolin-3-yl)butyl)amino)-4-oxobutanamido)-*N*-(1-phenethylpiperidin-4-yl)propanamido)phenyl)butanoic acid (42, HF-7)

To a solution of compound **41** (20.9 mg, 0.209 mmol) in CH_2Cl_2 (0.5 mL) was added TFA (0.5 mL) at 0 °C. The reaction mixture was stirred at 0 °C for 30 min and at room temperature for 30 min. The solvents were removed under reduced pressure. Traces of TFA were removed from a reaction mixture by azeotropic distillations with toluene (2 × 0.5 mL) under reduced pressure. The residue was purified by silica gel chromatography (CH_2Cl_2/MeOH 9:1 to 5:1) to give HF-7 (**42**) as a colorless oil (12.0 mg, 61%).

^{1}H NMR (600 MHz, Methanol-d_4) δ 7.32 (d, *J* = 8.1 Hz, 2H), 7.29 – 7.22 (m, 2H), 7.21 – 7.14 (m, 3H), 7.11 (d, *J* = 8.3 Hz, 2H), 6.76 (d, *J* = 8.1 Hz, 1H), 6.64 (d, *J* = 8.2 Hz, 1H), 5.63 (d, *J* = 10.0 Hz, 1H), 5.51 (dt, *J* = 10.0, 2.8 Hz, 1H), 5.20 – 5.15 (m, 1H), 5.15 – 5.10 (m, 1H), 4.68 – 4.59 (m, 1H), 3.73 (s, 1H), 3.37 – 3.32 (m, 2H), 3.24 – 3.16 (m, 4H), 3.11 (d, *J* = 19.3 Hz, 1H), 2.93 – 2.87 (m, 2H), 2.83 – 2.76 (m, 3H), 2.76 – 2.64 (m, 5H), 2.59 – 2.37 (m, 8H), 2.30 – 2.13 (m, 4H), 2.24 (s, 3H), 2.10 (s, 3H), 1.93 – 1.84 (m, 4H), 1.67 – 1.60 (m, 2H), 1.60 – 1.48 (m, 4H), 1.36 – 1.24 (m, 2H).
^{13}C NMR (151 MHz, Methanol-d_4) δ 174.52, 174.39, 172.94, 172.02, 170.18, 150.73, 144.79, 140.21, 136.93, 133.27, 132.98, 132.34, 131.29, 130.85, 130.02, 129.74, 129.67, 129.59, 127.45, 123.26, 120.60, 89.86, 73.86, 69.36, 64.42, 60.59, 58.37, 55.24, 53.69, 52.95, 46.25, 44.07, 40.31, 39.93, 36.78, 36.22, 35.80, 35.14, 33.34, 32.22, 32.13, 30.46, 28.17, 24.80, 22.65, 20.60, 20.43.
High resolution mass spectrometry (ESI) found 946.4956 [calculated for $C_{54}H_{68}N_5O_{10}$ (M + H^+) 946.4960].

HF-8 Synthesis (compounds **43–45**)

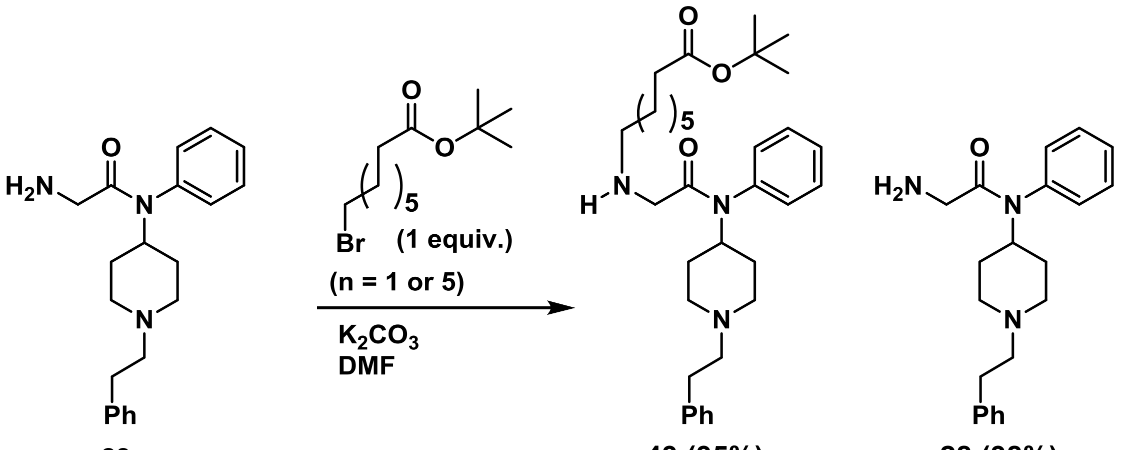

tert-Butyl 8-bromooctanoate was prepared according known procedure.
(*Org. Biomol. Chem.*, **2008**, *6*, 4242-4252.)

tert-Butyl 8-((2-oxo-2-((1-phenethylpiperidin-4-yl)(phenyl)amino)ethyl)amino)octanoate (43)

To a solution of primary amine **28** (150 mg, 0.444 mmol) in DMF (1.4 mL) was added a solution of *tert*-butyl 8-bromooctanoate (124 mg, 0.444 mmol) in DMF (3.0 mL) and K_2CO_3 (123 mg, 0.888 mmol). The reaction mixture was stirred at room temperature for 10 h. The mixture was diluted with CH_2Cl_2 (30 mL), washed with saturated $NaHCO_3$ aq. (10 mL), and dried with Na_2SO_4. After filtration and evaporation, the crude was purified by silica gel chromatography (CH_2Cl_2/MeOH 19:1 to 7:1) to give compound **43** as a colorless oil (83.8 mg, 35%) and starting material **28** (57.7 mg, 38%)

^{1}H NMR (500 MHz, Chloroform-*d*) δ 7.43 – 7.37 (m, 3H), 7.29 – 7.22 (m, 2H), 7.21 – 7.12 (m, 3H), 7.12 – 7.06 (m, 2H), 4.67 (tt, *J* = 12.2, 4.2 Hz, 1H), 3.00 (d, *J* = 11.8 Hz, 2H), 2.94 (s, 2H), 2.76 – 2.69 (m, 2H), 2.58 – 2.50 (m, 2H), 2.44 (t, *J* = 7.2 Hz, 2H), 2.24 – 2.12 (m, 5H), 1.81 (d, *J* = 11.8 Hz, 2H), 1.58 – 1.51 (m, 2H), 1.51 – 1.38 (m, 4H), 1.43 (s, 9H), 1.33 – 1.23 (m, 6H).
^{13}C NMR (126 MHz, Chloroform-*d*) δ 173.38, 170.94, 140.30, 137.54, 130.43, 129.59, 128.83, 128.74, 128.50, 126.17, 80.01, 60.57, 53.13, 52.71, 51.99, 49.90, 35.70, 33.94, 30.54, 30.13, 29.31, 29.13, 28.25, 27.23, 25.16.
High resolution mass spectrometry (ESI) found 536.3852 [calculated for $C_{33}H_{50}N_3O_3$ (M + H^+) 536.3846].

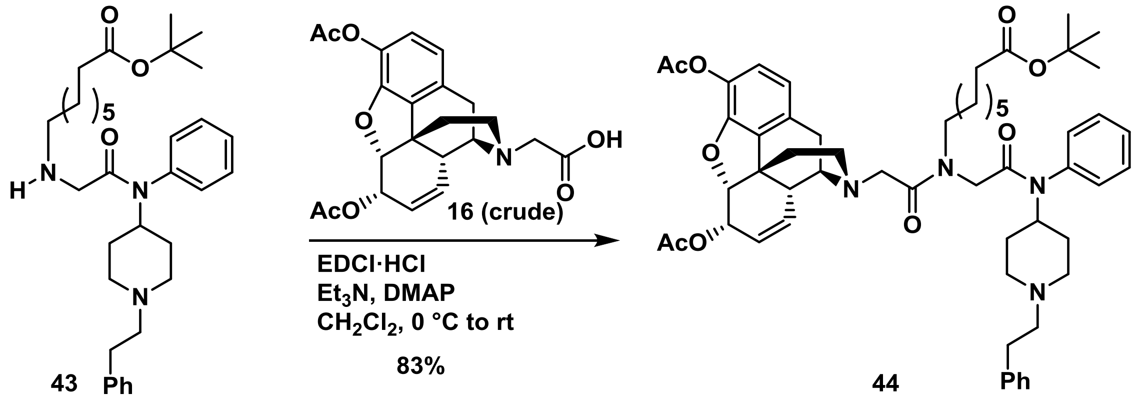

(4*R*,4a*R*,7*S*,7a*R*,12b*S*)-3-(2-((8-(*tert*-butoxy)-8-oxooctyl)(2-oxo-2-((1-phenethylpiperidin-4-yl)(phenyl)amino)ethyl)amino)-2-oxoethyl)-2,3,4,4a,7,7a-hexahydro-1*H*-4,12-methanobenzofuro[3,2-*e*]isoquinoline-7,9-diyl diacetate (44, HF-8 *tert*-Bu ester)

To a solution of compound **13** (51.5 mg, 0.110 mmol) in CH_2Cl_2 (0.5 mL) was added TFA (0.5 mL) The reaction mixture was stirred at 0 °C for 30 min and at room temperature for 7 h. The solvents were removed under reduced pressure. Traces of TFA were removed from a reaction mixture by azeotropic distillations with toluene (2 × 1 mL) under reduced pressure.

To a solution of the above crude product (compound **16**) in CH_2Cl_2 (0.9 mL) were added Et_3N (57 μL, 0.412 mmol), secondary amine **43** (49.0 mg, 0.0915 mmol) and DMAP (2.2 mg, 00183 mmol). After cooling to 0 °C, EDCI·HCl (21.1 mg, 0.110 mmol) was added to the mixture. The reaction mixture was stirred at 0 °C for 30 min and room temperature for 5 h. The mixture was diluted with CH_2Cl_2 (15 mL), washed with saturated $NaHCO_3$ aq. (5 mL), and dried with Na_2SO_4. After filtration and evaporation, the crude was purified by silica gel chromatography (CH_2Cl_2/MeOH 40:1 to 19:1) to give compound **44** as a colorless oil (71.1 mg, 83%).

Two rotamers were observed in NMR analysis.

^{1}H NMR (500 MHz, Chloroform-*d*) δ 7.50 – 7.32 (m, 3H), 7.25 – 7.17 (m, 3H), 7.17 – 7.08 (m, 4H), 6.78 – 6.70 (m, 1H), 6.59 – 6.52 (m, 1H), 5.64 – 5.55 (m, 1H), 5.45 – 5.35 (m, 1H), 5.15 – 5.01 (m, 2H), 4.68 – 4.55 (m, 1H), 4.03 – 3.82 (m, 1H), 3.68 – 3.48 (m, 2H), 3.43 – 3.15 (m, 4H), 3.09 – 2.91 (m, 3H), 2.81 – 2.59 (m, 3H), 2.59 – 2.31 (m, 5H), 2.31 – 1.75 (m, 14H), 1.62 – 1.33 (m, 15H), 1.33 – 1.11 (m, 6H).

^{13}C NMR (126 MHz, Chloroform-*d*) δ 173.19, 173.08, 170.45, 169.89, 169.64, 168.40, 167.94, 167.47, 149.44, 149.38, 140.19, 140.11, 137.57, 137.32, 132.22, 132.12, 131.77, 131.75, 131.43, 131.13, 130.43, 130.38, 130.15, 130.09, 129.97, 129.90, 129.65, 129.58, 129.40, 129.24, 128.61, 128.38, 128.37, 128.31, 126.06, 126.02, 122.05, 121.91, 119.49, 119.44, 88.75, 88.71, 79.99, 79.90, 68.16, 60.43, 60.36, 59.01, 57.99, 56.56, 56.45, 53.09, 53.01, 52.91, 52.73, 50.34, 48.97,

48.41, 47.35, 45.20, 43.05, 43.03, 40.59, 40.53, 35.52, 35.45, 35.16, 33.81, 30.53, 30.50, 30.38, 30.35, 29.12, 29.02, 29.01, 28.68, 28.14, 27.14, 26.87, 26.76, 24.99, 24.95, 21.84, 21.67, 20.71, 20.70, 20.65.

High resolution mass spectrometry (ESI) found 931.5219 [calculated for $C_{55}H_{71}N_4O_9$ (M + H^+) 931.5215].

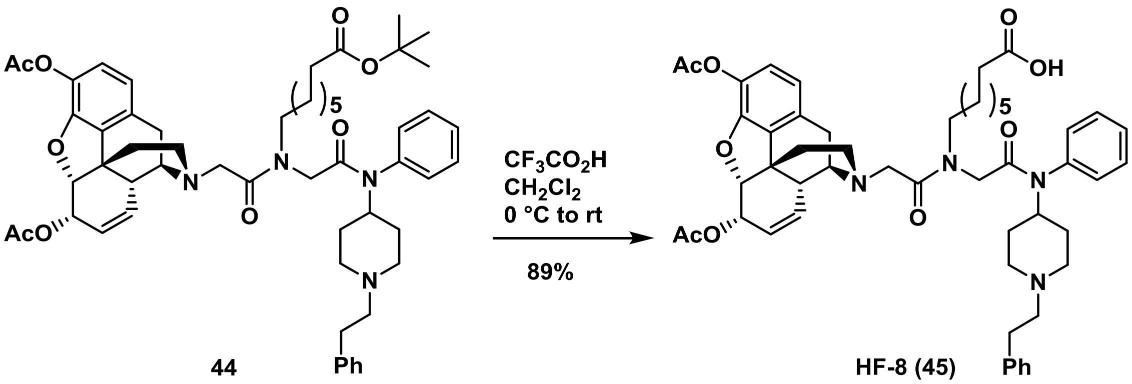

8-(2-((4*R*,4a*R*,7*S*,7a*R*,12b*S*)-7,9-diacetoxy-1,2,4,4a,7,7a-hexahydro-3*H*-4,12-methanobenzofuro[3,2-*e*]isoquinolin-3-yl)-*N*-(2-oxo-2-((1-phenethylpiperidin-4-yl)(phenyl)amino)ethyl)acetamido)octanoic acid (45, HF-8)

To a solution of compound **44** (60.1 mg, 0.0645 mmol) in CH_2Cl_2 (0.8 mL) was added TFA (0.8 mL). The reaction mixture was stirred at 0 °C for 30 min and at room temperature for 30 min. The solvents were removed under reduced pressure. Traces of TFA were removed from a reaction mixture by azeotropic distillations with toluene (2 × 1 mL) under reduced pressure. The residue was purified by silica gel chromatography (CH_2Cl_2/MeOH 9:1 to 4:1) to give HF-8 (**45**) as a pale yellow oil (50.1 mg, 89%).

Two rotamers were observed in NMR analysis.

^{1}H NMR (600 MHz, Methanol-d_4) δ 7.61 – 7.50 (m, 3H), 7.41 – 7.36 (m, 2H), 7.35 – 7.28 (m, 2H), 7.27 – 7.22 (m, 3H), 6.82 – 6.77 (m, 1H), 6.70 – 6.64 (m, 1H), 5.71 – 5.64 (m, 1H), 5.55 – 5.47 (m, 1H), 5.22 – 5.09 (m, 2H), 4.84 – 4.75 (m, 1H), 4.09 – 3.82 (m, 3H), 3.78 – 3.58 (m, 5H), 3.47 – 3.34 (m, 2H), 3.30 – 3.24 (m, 2H), 3.24 – 3.11 (m, 3H), 3.09 – 2.93 (m, 3H), 2.92 – 2.60 (m, 3H), 2.34 – 2.06 (m, 10H), 1.98 – 1.86 (m, 1H), 1.85 – 1.68 (m, 2H), 1.65 – 1.51 (m, 3H), 1.48 – 1.40 (m, 1H), 1.40 – 1.19 (m, 6H).

^{13}C NMR (151 MHz, Methanol-d_4) δ 172.11, 172.02, 170.20, 170.19, 169.79, 169.78, 169.66, 169.65, 150.89, 150.79, 138.15, 137.94, 137.51, 137.47, 133.43, 133.37, 132.40, 132.24, 131.95,

131.91, 131.50, 131.38, 131.28, 131.21, 131.00, 130.75, 130.42, 129.97, 129.73, 129.72, 129.18, 128.95, 128.28, 123.56, 123.52, 120.75, 120.71, 89.86, 89.70, 69.39, 69.22, 64.40, 60.11, 59.63, 58.81, 58.24, 57.15, 53.15, 53.11, 52.20, 51.76, 51.36, 51.35, 50.23, 50.21, 47.04, 46.76, 43.79, 43.57, 40.39, 39.91, 34.58, 31.48, 31.47, 30.10, 30.07, 30.02, 29.37, 28.73, 28.08, 27.61, 27.59, 25.98, 23.91, 20.61, 20.56, 20.42, 20.41.

$[\alpha]_D^{22}$ = -60.7 (*c*. 0.27, CH_3OH)

High resolution mass spectrometry (ESI) found 875.4587 [calculated for $C_{51}H_{63}N_4O_9$ $(M + H^+)$ 875.4589].

HF-9 Synthesis (compound **46–49**)

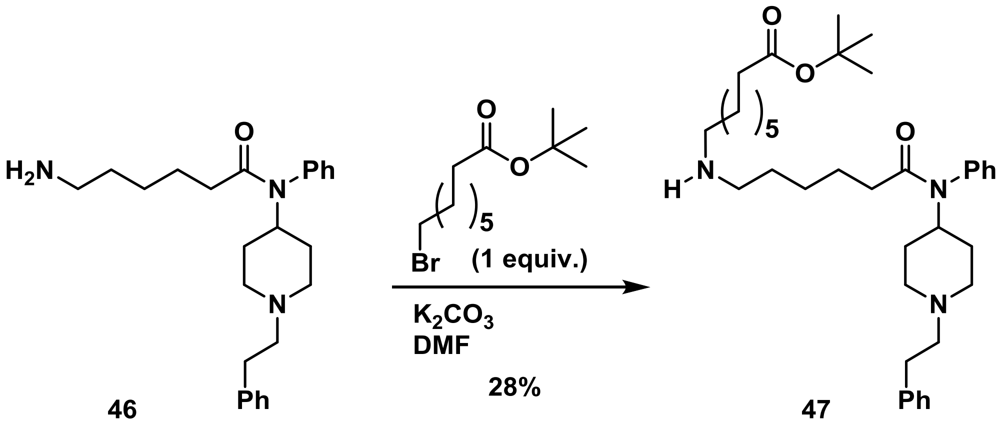

Compound **46** was prepared according known procedure.
(*Bioorg. Med. Chem. Lett.* **2013**, *23*, 3434-3437.)

***tert*-Butyl 8-((6-oxo-6-((1-phenethylpiperidin-4-yl)(phenyl)amino)hexyl)amino)octanoate (47)**

To a solution of primary amine **46** (980 mg, 2.49 mmol) in DMF (20 mL) were added a solution of *tert*-butyl 8-bromooctanoate (730 mg, 2.61 mmol) in DMF (5.0 mL) and K_2CO_3 (123 mg, 0.888 mmol). The reaction mixture was stirred at room temperature for 10 h. The mixture was diluted with CH_2Cl_2 (50 mL), washed with saturated $NaHCO_3$ aq. (15 mL), and dried with Na_2SO_4. After filtration and evaporation, the crude was purified by silica gel chromatography (CH_2Cl_2/MeOH 9:1 to 5:1) to give compound **47** as a pale yellow oil (407 mg, 28%).

^{1}H NMR (500 MHz, Chloroform-*d*) δ 7.43 – 7.32 (m, 3H), 7.30 – 7.21 (m, 2H), 7.21 – 7.13 (m, 3H), 7.13 – 7.03 (m, 2H), 4.68 (tt, *J* = 12.5, 3.9 Hz, 1H), 2.99 (d, *J* = 11.7 Hz, 2H), 2.76 – 2.69 (m, 2H), 2.57 – 2.48 (m, 6H), 2.23 – 2.11 (m, 4H), 1.91 (t, *J* = 7.5 Hz, 2H), 1.84 – 1.76 (m, 2H), 1.60 – 1.51 (m, 4H), 1.48 – 1.34 (m, 5H), 1.44 (s, 9H), 1.34 – 1.15 (m, 10H).
^{13}C NMR (126 MHz, Chloroform-*d*) δ 173.38, 172.80, 140.38, 138.94, 130.56, 129.40, 128.74, 128.49, 128.40, 126.13, 80.02, 60.63, 53.24, 52.28, 50.22, 50.00, 35.70, 35.18, 33.99, 30.73, 30.24, 29.99, 29.37, 29.16, 28.25, 27.37, 27.17, 25.41, 25.17.
High resolution mass spectrometry (ESI) found 592.4485 [calculated for $C_{37}H_{58}N_3O_{53}$ (M + H$^+$) 592.4472].

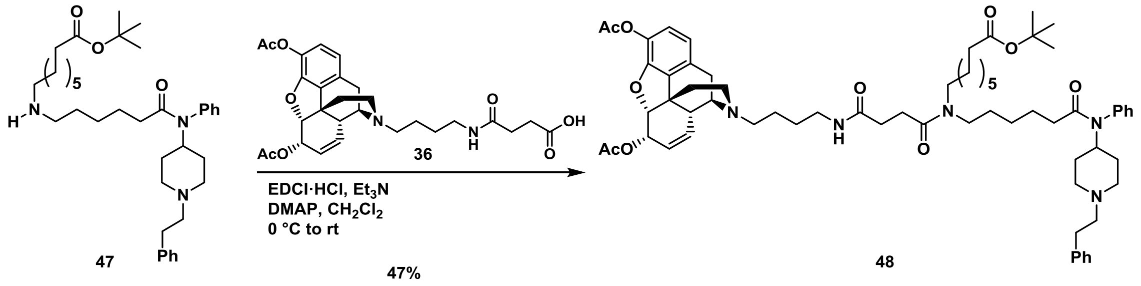

(4*R*,4a*R*,7*S*,7a*R*,12b*S*)-3-(4-(4-((8-(*tert*-butoxy)-8-oxooctyl)(6-oxo-6-((1-phenethylpiperidin-4-yl)(phenyl)amino)hexyl)amino)-4-oxobutanamido)butyl)-2,3,4,4a,7,7a-hexahydro-1*H*-4,12-methanobenzofuro[3,2-*e*]isoquinoline-7,9-diyl diacetate (48, HF-9 *tert*-butyl ester)

To a solution of the heroin derivative **36** (33.7 mg, 0.0683 mmol), fentanyl derivative **47** (48.5 mg, 0.0819 mmol), Et_3N (43 μL, 0.307 mmol) and DMAP (1.7 mg, 0.0137 mmol) in CH_2Cl_2 (0.7 mL) was added EDCI·HCl (19.6 mg, 0.102 mmol) at 0 °C. The reaction mixture was stirred at 0 °C for 30 min and at room temperature for 12 h. The mixture was diluted with CH_2Cl_2, washed with saturated $NaHCO_3$ aq., and dried with Na_2SO_4. After filtration and evaporation, the crude was purified by silica gel chromatography (CH_2Cl_2/MeOH 10:1 to 6:1) to give compound **48** as a colorless oil (35.3 mg, 47%).

Two rotamers were observed in NMR analysis.

^{1}H NMR (600 MHz, Chloroform-*d*) δ 7.44 – 7.32 (m, 3H), 7.25 (t, *J* = 7.8 Hz, 2H), 7.20 – 7.12 (m, 3H), 7.10 – 7.03 (m, 2H), 6.75 (d, *J* = 8.2 Hz, 1H), 6.56 (d, *J* = 8.2 Hz, 1H), 6.40 – 6.32 (m, 1H), 5.61 (d, *J* = 9.9 Hz, 1H), 5.42 (dt, *J* = 10.0, 2.8 Hz, 1H), 5.17 – 5.12 (m, 1H), 5.10 (d, *J* = 6.0 Hz, 1H), 4.69-4.63 (m, 1H), 3.44 (s, 1H), 3.28 – 2.92 (m, 9H), 2.77 – 2.44 (m, 11H), 2.39 – 2.08 (m, 6H), 2.26 (s, 3H), 2.13 (s, 3H), 2.08 – 1.71 (m, 7H), 1.64 – 1.35 (m, 20H), 1.35 – 1.04 (m, 11H).

^{13}C NMR (151 MHz, Chloroform-*d*) δ 173.36, 173.29, 172.70, 172.67, 172.43, 171.47, 171.46, 170.60, 168.58, 149.52, 140.24, 138.88, 138.84, 132.38, 131.88, 131.71, 130.52, 130.50, 129.73, 129.53, 129.47, 128.76, 128.54, 128.52, 128.47, 126.19, 122.02, 119.46, 88.89, 80.13, 80.07, 68.28, 60.57, 57.03, 54.47, 54.46, 53.22, 52.35, 52.27, 48.05, 47.85, 46.27, 46.15, 44.94, 43.52, 43.51, 40.66, 39.41, 39.39, 35.68, 35.63, 35.26, 35.13, 34.99, 33.90, 33.88, 31.96, 31.94, 30.64, 30.63, 29.84, 29.27, 29.21, 29.18, 29.16, 29.04, 29.02, 28.87, 28.27, 27.88, 27.79, 27.57, 27.56, 27.02, 26.91, 26.90, 26.70, 25.44, 25.21, 25.14, 25.09, 25.03, 21.77, 20.83, 20.79.

$[\alpha]_D^{22}$ = -51.1 (*c*. 0.29, CH_3OH)

High resolution mass spectrometry (ESI) found 1100.6689 [calculated for $C_{65}H_{90}N_5O_{10}$ (M + H^+) 1100.6682].

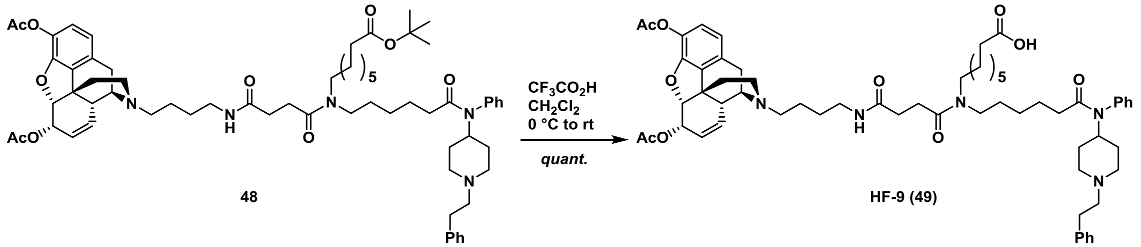

8-(4-((4-((4*R*,4a*R*,7*S*,7a*R*,12b*S*)-7,9-diacetoxy-1,2,4,4a,7,7a-hexahydro-3*H*-4,12-methanobenzofuro[3,2-*e*]isoquinolin-3-yl)butyl)amino)-4-oxo-*N*-(6-oxo-6-((1-phenethylpiperidin-4-yl)(phenyl)amino)hexyl)butanamido)octanoic acid (49, HF-9)

To a solution of compound **48** (12.0 mg, 0.0109 mmol) in CH_2Cl_2 (0.5 mL) was added TFA (0.5 mL). The reaction mixture was stirred at 0 °C for 30 min and room temperature for 30 min. The solvents were removed under reduced pressure. Traces of TFA were removed from a reaction mixture by azeotropic distillations with toluene (2 × 1 mL) under reduced pressure. The residue was purified by silica gel chromatography (CH_2Cl_2/MeOH = 9:1 to 2:1) to give HF-9 (**49**) as a colorless oil (11.2 mg, quantitive yield).

Two rotamers were observed in NMR analysis.

^{1}H NMR (600 MHz, Methanol-d_4) δ 7.55 – 7.43 (m, 3H), 7.33 – 7.16 (m, 7H), 6.83 – 6.78 (m, 1H), 6.71 – 6.67 (m, 1H), 5.71 – 5.65 (m, 1H), 5.56 – 5.50 (m, 1H), 5.23 – 5.15 (m, 2H), 4.76 – 4.68 (m, 1H), 4.01 (s, 1H), 3.50 – 3.40 (m, 2H), 3.30 – 3.10 (m, 7H), 3.10 – 2.56 (m, 13H), 2.49 (t, J = 6.7 Hz, 2H), 2.40 – 1.86 (m, 14H), 1.86 – 1.43 (m, 13H), 1.43 – 1.03 (m, 11H).

^{13}C NMR (151 MHz, Methanol-d_4) δ 174.99, 174.90, 173.62, 173.55, 172.01, 171.99, 170.11, 163.19, 162.96, 150.76, 139.55, 138.76, 133.52, 131.77, 131.66, 131.41, 130.88, 130.86, 130.62, 130.14, 130.11, 129.76, 129.72, 129.71, 128.55, 127.86, 123.73, 120.85, 120.84, 119.16, 117.22, 89.49, 69.05, 64.33, 59.57, 59.09, 55.11, 55.07, 53.43, 52.20, 52.16, 47.23, 47.03, 46.60, 46.56, 43.55, 39.57, 39.32, 37.62, 35.80, 34.12, 32.30, 32.28, 32.06, 30.72, 30.45, 30.20, 29.83, 29.61, 29.60, 29.54, 29.40, 28.42, 27.94, 27.85, 27.76, 27.53, 27.36, 26.92, 26.27, 26.15, 23.84, 23.11, 20.57, 20.43.

High resolution mass spectrometry (ESI) found 1044.6039 [calculated for $C_{61}H_{82}N_5O_{10}$ (M + H^+) 1044.6056].

Fentanyl hapten synthesis (**53**)

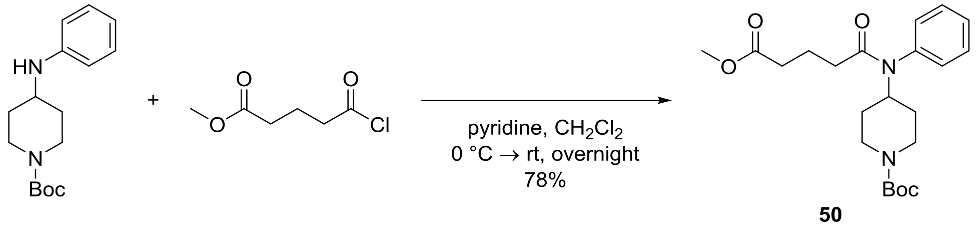

tert-Butyl 4-(5-methoxy-5-oxo-_N_-phenylpentanamido)piperidine-1-carboxylate (50)

To a solution of N-Boc-4-(phenylamino)piperidine (276 mg, 1.0 mmol) in CH_2Cl_2 (5 mL) cooled to 0 °C was added pyridine (438 µL, 2.4 mmol), followed by glutaric acid monomethyl ester chloride (330 µL, 2.0 mmol). The reaction was stirred at 0 °C and allowed to warm to room temperature. After 16 h, the reaction was diluted with CH_2Cl_2 (15 mL), washed with 1 N HCl (2 × 10 mL), dried over Na_2SO_4 and concentrated. Purification by silica gel chromatography (hexanes/EtOAc 1:0 to 1:1) yielded **50** as a colorless oil (315 mg, 78.0%).

^{1}H NMR (500 MHz, Chloroform-*d*) δ 7.44 – 7.34 (m, 3H), 7.03 (dd, *J* = 7.8, 1.8 Hz, 2H), 4.74 (tt, *J* = 12.2, 3.8 Hz, 1H), 4.09 (d, *J* = 5.5 Hz, 2H), 3.58 (s, 3H), 2.75 (t, *J* = 14.1 Hz, 2H), 2.25 (t, *J* = 7.2 Hz, 2H), 1.97 – 1.91 (m, 2H), 1.88 – 1.81 (m, 2H), 1.74 (ddd, *J* = 12.2, 4.3, 2.3 Hz, 2H), 1.37 (s, 9H), 1.19 (qd, *J* = 13.4, 5.1 Hz, 2H).

^{13}C NMR (151 MHz, Chloroform-*d*) δ 173.01, 171.23, 154.00, 137.98, 129.69, 128.96, 128.04, 78.98, 51.82, 50.89, 33.42, 32.67, 32.36, 29.97, 27.83, 20.07.

High resolution mass spectrometry (ESI) found 405.2414 [calculated for $C_{22}H_{32}N_2O_5$ (M + H$^+$): 405.23].

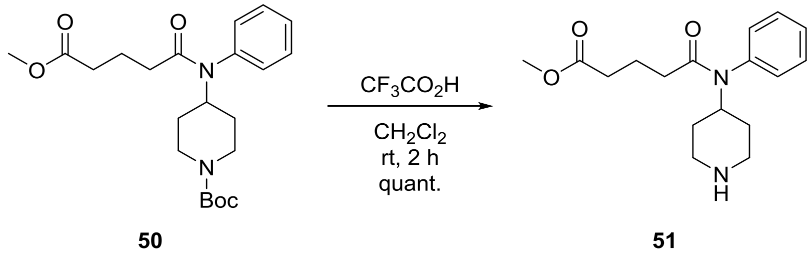

Methyl 5-oxo-5-(phenyl(piperidin-4-yl)amino)pentanoate (51)

To a solution of **50** (240 mg, 0.594 mmol) dissolved in 0.5 mL CH_2Cl_2 was added 2 mL trifluoroacetic acid. The solution was stirred at room temperature for 2 hours, then concentrated. **51** was obtained as a colorless oil (181 mg, quantitative).

^{1}H NMR (600 MHz, Chloroform-*d*) δ 7.52 – 7.47 (m, 3H), 7.12 – 7.07 (m, 2H), 4.83 (tt, *J* = 12.1, 3.8 Hz, 1H), 3.63 (s, 3H), 3.57 – 3.51 (m, 2H), 3.12 (dtd, *J* = 13.9, 11.2, 2.7 Hz, 2H), 2.28 (t, *J* = 7.2 Hz, 2H), 2.12 – 2.05 (m, 4H), 1.86 (p, *J* = 7.3 Hz, 2H), 1.70 (qd, *J* = 13.5, 4.1 Hz, 2H).
^{13}C NMR (151 MHz, Chloroform-*d*) δ 173.88, 173.64, 136.21, 129.69, 129.27, 128.72, 51.43, 50.00, 43.84, 33.40, 32.53, 26.58, 20.08.
High resolution mass spectrometry (ESI) found 305.1946 [calculated for $C_{17}H_{24}N_2O_3$ (M + H$^+$): 305.18].

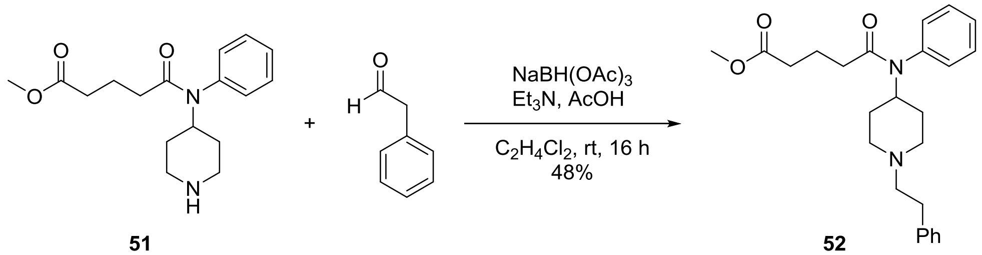

51 **52**

Methyl 5-oxo-5-((1-phenethylpiperidin-4-yl)(phenyl)amino)pentanoate (52)

In an oven dried flask under argon, triethylamine (124 µL, 0.891 mmol), acetic acid (54 µL, 0.950 mmol), and 2-phenylacetaldehyde (73 µL, 0.653 mmol) were added to a solution of **51** (181 mg, 0.594 mmol) in dichloroethane (4 mL). The solution was stirred at room temperature for 15 min. Sodium triacetoxyborohydride (189 mg, 0.891 mmol) was added in 3 portions. The white suspension was stirred at room temperature for 16 hours. The reaction mixture was quenched by slow addition, then dilution with water. The reaction mixture was extracted with CH_2Cl_2 (10 mL × 3), and the combined organic extracts were washed with sat. $NaHCO_3$, dried over Na_2SO_4, and concentrated. Purification by amine-functionalized silica gel chromatography (hexanes/EtOAc 1:0 to 1:1) yielded **52** as a colorless oil (109 mg, 45.0%).
^{1}H NMR (600 MHz, Chloroform-*d*) δ 7.43 – 7.36 (m, 3H), 7.30 – 7.25 (m, 2H), 7.22 – 7.19 (m, 1H), 7.17 (dt, *J* = 7.9, 1.3 Hz, 2H), 7.11 – 7.07 (m, 2H), 4.69 (tt, *J* = 12.2, 4.0 Hz, 1H), 3.62 (s, 3H), 3.05 – 2.98 (m, 2H), 2.78 – 2.71 (m, 2H), 2.59 – 2.52 (m, 2H), 2.29 (t, *J* = 7.3 Hz, 2H), 2.17 (td, *J* = 12.0, 2.3 Hz, 2H), 1.98 (td, *J* = 7.2, 0.9 Hz, 2H), 1.92 – 1.85 (m, 2H), 1.85 – 1.78 (m, 2H), 1.45 (qd, *J* = 12.3, 3.9 Hz, 2H).
^{13}C NMR (151 MHz, Chloroform-*d*) δ 173.18, 171.30, 139.76, 138.14, 129.92, 128.90, 128.16, 127.91, 125.56, 60.02, 52.62, 51.81, 50.98, 33.56, 33.38, 32.82, 30.10, 20.20.
High resolution mass spectrometry (ESI) found 409.25746 [calculated for $C_{25}H_{32}N_2O_3$ (M + H$^+$): 409.24].

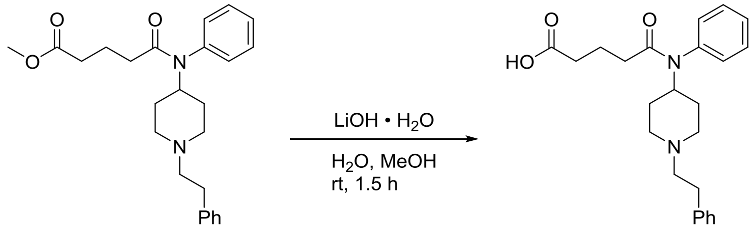

52 **53**

5-Oxo-5-((1-phenethylpiperidin-4-yl)(phenyl)amino)pentanoic acid (53, Fentanyl Hapten)

To a solution of **52** (58 mg, 0.142 mmol) in MeOH (1 mL) and H_2O (0.5 mL) was added LiOH · H_2O (42mg, 0.995 mmol). The reaction mixture was stirred at room temperature. After 1.5 hours, the reaction mixture was acidified by the addition of 1 N HCl (2 mL), then extracted with EtOAc (3 mL × 2). Combined organic extracts were dried over Na_2SO_4, then concentrated. Purification by reverse phase C18 chromatography (HCOOH/MeCN 95:5 to 0:100) yielded **53** as a white solid (13.0 mg, 23.2%).

^{1}H NMR (600 MHz, DMSO-d_6) δ 7.47 (dd, J = 8.3, 6.5 Hz, 2H), 7.44 – 7.40 (m, 1H), 7.26 – 7.21 (m, 2H), 7.20 – 7.12 (m, 5H), 4.44 (tt, J = 12.1, 3.9 Hz, 1H), 2.94 – 2.88 (m, 2H), 2.64 (dd, J = 9.4, 6.4 Hz, 2H), 2.45 – 2.41 (m, 2H), 2.06 (t, J = 7.3 Hz, 2H), 2.03 – 1.97 (m, 2H), 1.85 (t, J = 7.3 Hz, 2H), 1.71 – 1.66 (m, 2H), 1.63 (p, J = 7.4 Hz, 2H), 1.18 (qd, J = 12.4, 3.9 Hz, 2H).

^{13}C NMR (151 MHz, DMSO-d_6) δ 174.29, 170.61, 140.45, 138.65, 130.36, 129.27, 128.53, 128.22, 128.18, 125.76, 59.58, 52.54, 51.83, 33.59, 33.27, 32.99, 30.12, 20.42.

High resolution mass spectrometry (ESI) found 395.23358 [calculated for $C_{24}H_{30}N_2O_3$ (M + H$^+$): 395.23].

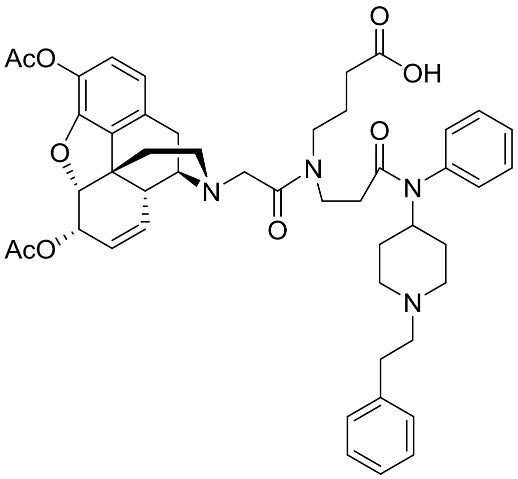

Chemical Formula: $C_{48}H_{56}N_4O_9$
Exact Mass: 832.4047
Molecular Weight: 832.9950

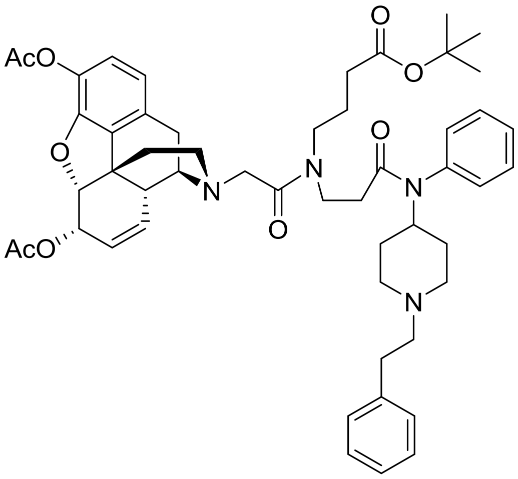

Chemical Formula: $C_{52}H_{64}N_4O_9$
Exact Mass: 888.4673
Molecular Weight: 889.1030

JAYN5463

High resolution mass spectrometry (ESI) found 889.4754 [calculated for $C_{47}H_{55}N_4O_9$ (M + H^+) 889.4746].
