# Supplementary material for: A chemically contiguous hapten approach for a heroin–fentanyl vaccine
Source: Beilstein J Org Chem. 2019 May 3;15:1020–31. doi: 10.3762/bjoc.15.100 (PMC6541359; doi:10.3762/bjoc.15.100)
Supplement: File 3 — NMR spectra of compounds 1–53. [file Beilstein_J_Org_Chem-15-1020-s003.pdf]

BEILSTEIN JOURNAL OF ORGANIC CHEMISTRY

Supporting Information

for

A chemically contiguous hapten approach for a heroin–fentanyl vaccine

Yoshihiro Natori, Candy S. Hwang, Lucy Lin, Lauren C. Smith, Bin Zhou and Kim D. Janda

Beilstein J. Org. Chem. **2019,** *15,* 1020–1031. doi:10.3762/bjoc.15.100

NMR spectra of compounds 1–53

License and Terms: This is a supporting information file under the terms of the Creative Commons Attribution License (http://creativecommons.org/licenses/by/4.0). Please note that the reuse, redistribution and reproduction in particular requires that the authors and source are credited.
The license is subject to the *Beilstein Journal of Organic Chemistry* terms and conditions: (https://www.beilstein-journals.org/bjoc)

A chemically contiguous hapten approach for a heroin-fentanyl vaccine

Yoshihiro Natori,[‡,1,2] Candy S. Hwang,[‡,1,3] Lucy Lin,[1] Lauren C. Smith,[1] Bin Zhou,[1] and Kim D. Janda*[,1]

[1] Departments of Chemistry, Immunology and Microbial Science, Skaggs Institute for Chemical Biology; The Scripps Research Institute, 10550 N Torrey Pines Rd, La Jolla, CA, 92037 USA

*Corresponding author email: Kim D. Janda - kdjanda@scripps.edu

‡ Authors had equal contribution

Keywords: Antinociception; heroin; fentanyl; vaccine; hapten

NMR Spectra of Compounds 1-53

^{1}H NMR Spectra of Norheroin (1) S1
^{13}C NMR Spectra of Norheroin (1) S2
^{1}H NMR Spectra of Compound 2 .... S3
^{13}C NMR Spectra of Compound 2 .... S4
^{1}H NMR Spectra of Compound 3 .... S5
^{13}C NMR Spectra of Compound 3 .... S6
^{1}H NMR Spectra of Compound 4 .... S7
^{13}C NMR Spectra of Compound 4 .... S8
^{1}H NMR Spectra of Compound 5 .... S9
^{13}C NMR Spectra of Compound 5 .... S10
^{1}H NMR Spectra of Compound 6 .... S11
^{13}C NMR Spectra of Compound 6 .... S12
^{1}H NMR Spectra of Compound 7 .... S13
^{13}C NMR Spectra of Compound 7 .... S14
^{1}H NMR Spectra of Compound 8 .... S15
^{13}C NMR Spectra of Compound 8 .... S16
^{1}H NMR Spectra of Compound 10 .... S17
^{13}C NMR Spectra of Compound 10 .... S18
^{1}H NMR Spectra of Compound 11 .... S19
^{13}C NMR Spectra of Compound 11 .... S20
^{1}H NMR Spectra of Compound 13 .... S21
^{13}C NMR Spectra of Compound 13 .... S22

^{1}H NMR Spectra of Compound 14 .... S23
^{13}C NMR Spectra of Compound 14 .... S24
^{1}H NMR Spectra of Compound 15 .... S25
^{13}C NMR Spectra of Compound 15 .... S26
^{1}H NMR Spectra of Compound 17 .... S27
^{13}C NMR Spectra of Compound 17 .... S28
^{1}H NMR Spectra of HF-3 (18) S29
^{13}C NMR Spectra of HF-3 (18) S30
^{1}H NMR Spectra of Compound 19 .... S31
^{13}C NMR Spectra of Compound 19 .... S32
^{1}H NMR Spectra of Compound 20 .... S33
^{13}C NMR Spectra of Compound 20 .... S34
^{1}H NMR Spectra of Compound 21 .... S35
^{13}C NMR Spectra of Compound 21 .... S36
^{1}H NMR Spectra of Compound 22 .... S37
^{13}C NMR Spectra of Compound 22 .... S38
^{1}H NMR Spectra of Compound 23 .... S39
^{13}C NMR Spectra of Compound 23 .... S40
^{1}H NMR Spectra of Compound 24 .... S41
^{13}C NMR Spectra of Compound 24 .... S42
^{1}H NMR Spectra of Compound 25 .... S43
^{13}C NMR Spectra of Compound 25 .... S44
^{1}H NMR Spectra of Compound 26 .... S45
^{13}C NMR Spectra of Compound 26 .... S46
^{1}H NMR Spectra of Compound 29 .... S47
^{13}C NMR Spectra of Compound 29 .... S48
^{1}H NMR Spectra of Compound 30 .... S49
^{13}C NMR Spectra of Compound 30 .... S50
^{1}H NMR Spectra of HF-5 (31) S51
^{13}C NMR Spectra of HF-5 (31) S52
^{1}H NMR Spectra of Compound 32 .... S53
^{13}C NMR Spectra of Compound 32 .... S54
^{1}H NMR Spectra of Compound 33 .... S55
^{13}C NMR Spectra of Compound 33 .... S56
^{1}H NMR Spectra of Compound 34 .... S57
^{13}C NMR Spectra of Compound 34 .... S58
^{1}H NMR Spectra of Compound 36 .... S59
^{13}C NMR Spectra of Compound 36 .... S60
^{1}H NMR Spectra of Compound 37 .... S61
^{13}C NMR Spectra of Compound 37 .... S62
^{1}H NMR Spectra of HF-6 (38) S63
^{13}C NMR Spectra of HF-6 (38) S64
^{1}H NMR Spectra of Compound 39 .... S65
^{13}C NMR Spectra of Compound 39 .... S66

^{1}H NMR Spectra of Compound 40 .... S67
^{13}C NMR Spectra of Compound 40 .... S68
^{1}H NMR Spectra of Compound 41 .... S69
^{13}C NMR Spectra of Compound 41 .... S70
^{1}H NMR Spectra of HF-7 (42) S71
^{13}C NMR Spectra of HF-7 (42) S72
^{1}H NMR Spectra of Compound 43 .... S73
^{13}C NMR Spectra of Compound 43 .... S74
^{1}H NMR Spectra of Compound 44 .... S75
^{13}C NMR Spectra of Compound 44 .... S76
^{1}H NMR Spectra of HF-8 (45) S77
^{13}C NMR Spectra of HF-8 (45) S78
^{1}H NMR Spectra of Compound 47 .... S79
^{13}C NMR Spectra of Compound 47 .... S80
^{1}H NMR Spectra of Compound 48 .... S81
^{13}C NMR Spectra of Compound 48 .... S82
^{1}H NMR Spectra of HF-9 (49) S83
^{13}C NMR Spectra of HF-9 (49) S84
^{1}H NMR Spectra of Compound 50 .... S85
^{13}C NMR Spectra of Compound 50 .... S86
^{1}H NMR Spectra of Compound 51 .... S87
^{13}C NMR Spectra of Compound 51 .... S88
^{1}H NMR Spectra of Compound 52 .... S89
^{13}C NMR Spectra of Compound 52 .... S90
^{1}H NMR Spectra of Fentanyl Hapten (53) S91
^{13}C NMR Spectra of Fentanyl Hapten (53) S92

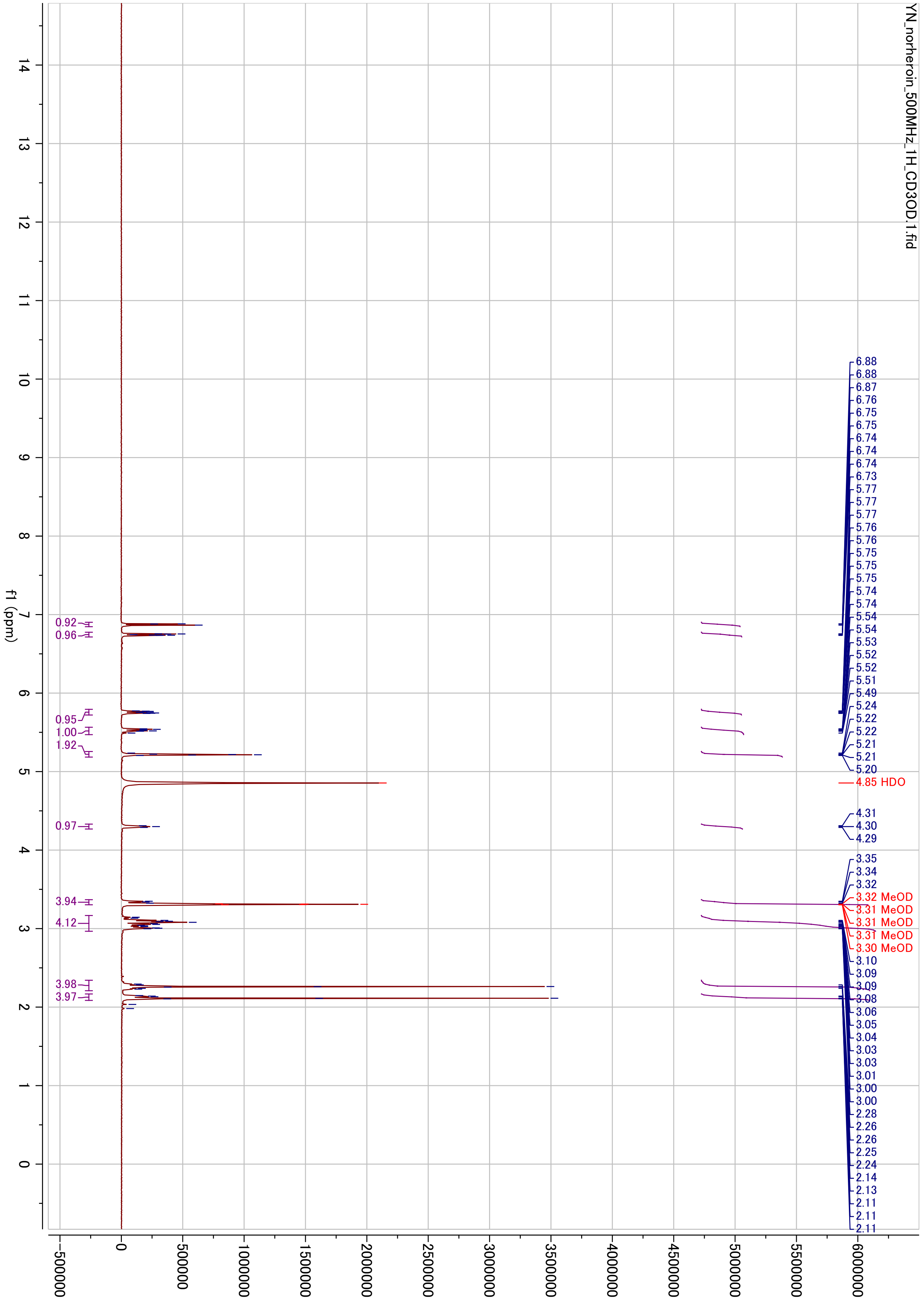
YN_norheroin_500MHz_1H_CD3OD.1.fid
f1 (ppm)

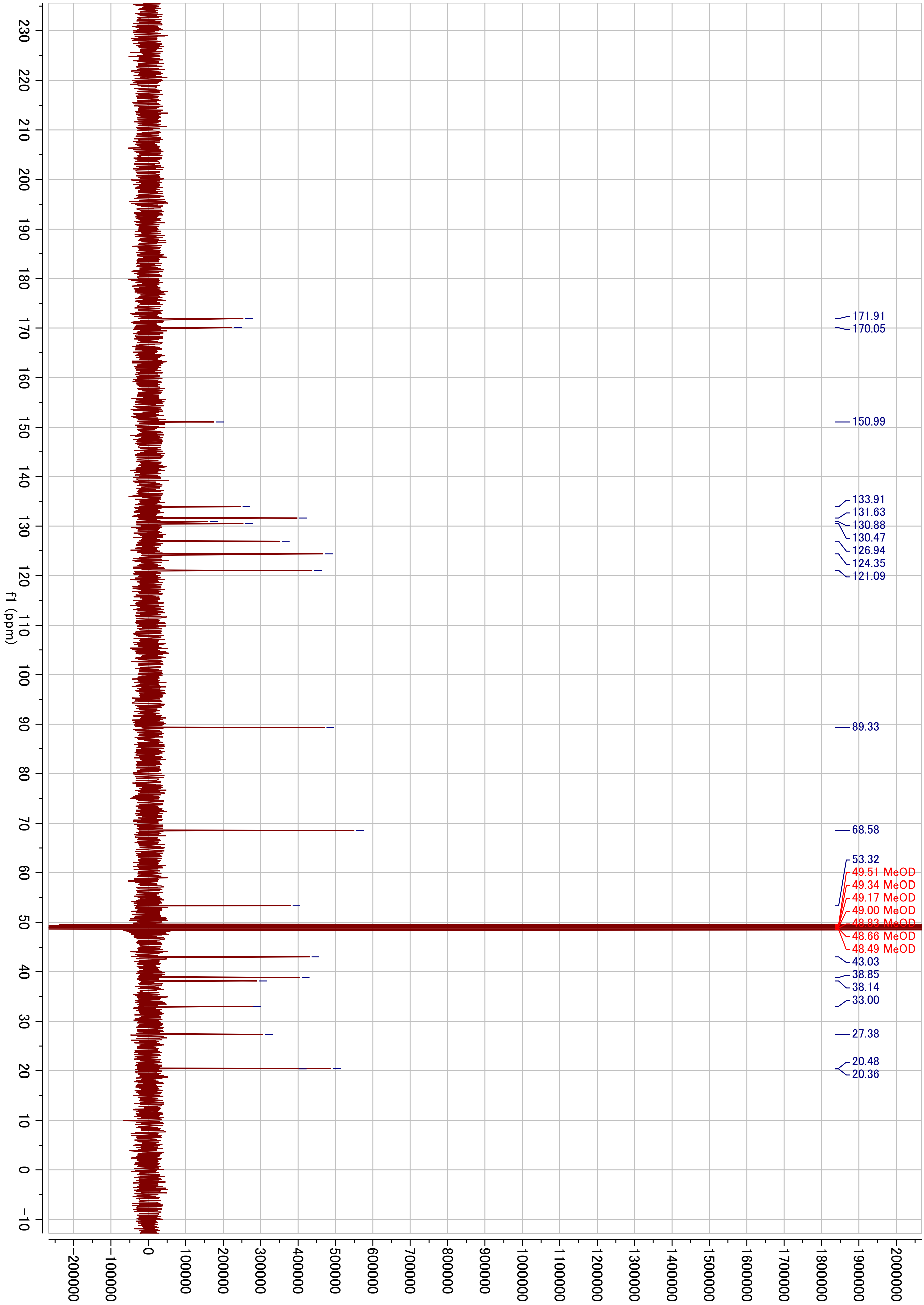
171.91
170.05
150.99
133.91
131.63
130.88
130.47
126.94
124.35
121.09
89.33
68.58
53.32
49.51 MeOD
49.34 MeOD
49.17 MeOD
49.00 MeOD
48.83 MeOD
48.66 MeOD
48.49 MeOD
43.03
38.85
38.14
33.00
27.38
20.48
20.36
f1 (ppm)

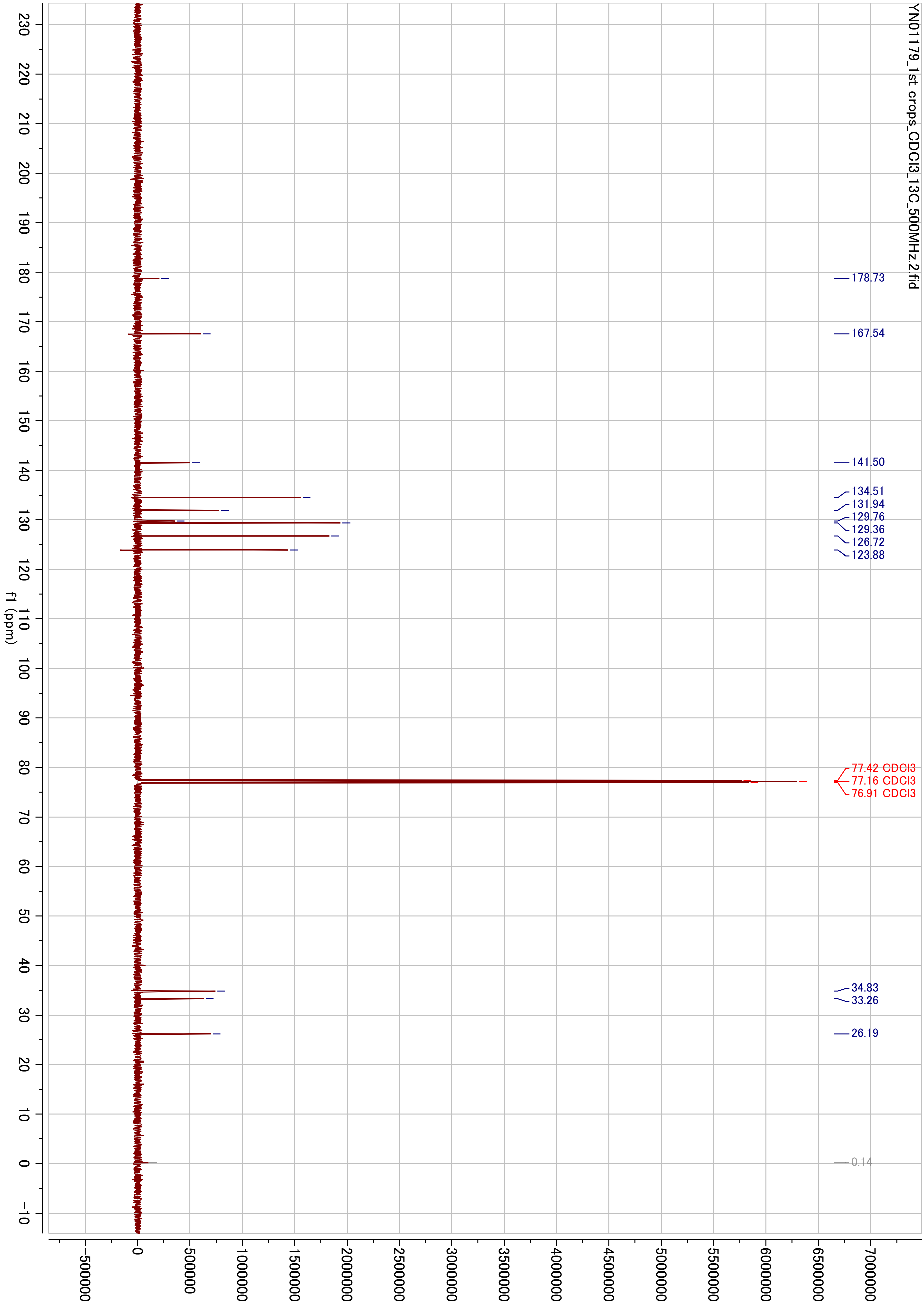

YN01179_1st crops_CDCl3_13C_500MHz.2.fid
178.73
167.54
141.50
134.51
131.94
129.76
129.36
126.72
123.88
77.42 CDCl3
77.16 CDCl3
76.91 CDCl3
34.83
33.26
26.19
0.14
f1 (ppm)

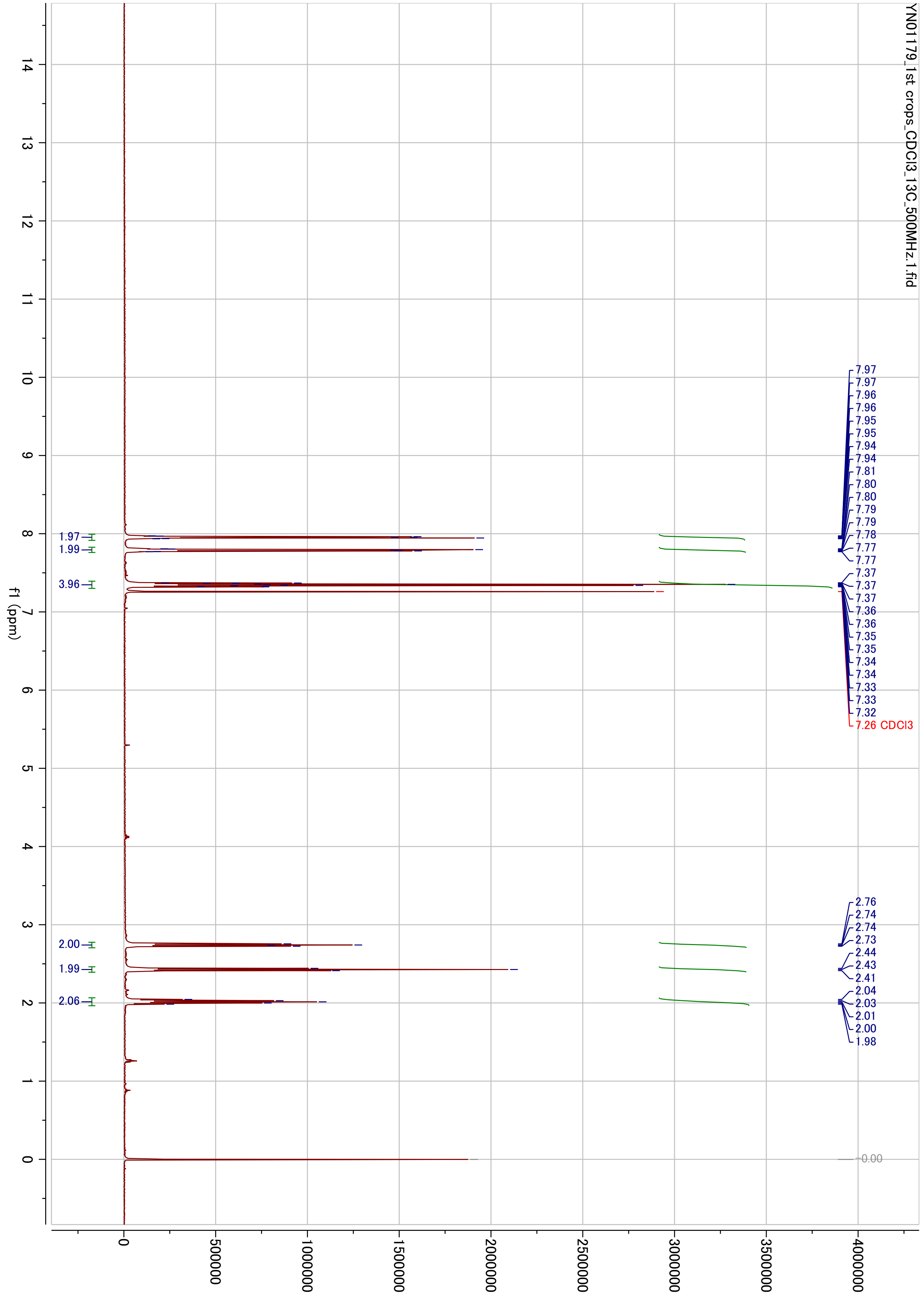
YN01179_1st crops_CDCl3_13C_500MHz.1.fid
7.97
7.97
7.96
7.96
7.95
7.95
7.94
7.94
7.81
7.80
7.80
7.79
7.79
7.78
7.77
7.77
7.37
7.37
7.37
7.36
7.36
7.35
7.35
7.34
7.34
7.33
7.33
7.32
7.26 CDCl3
2.76
2.74
2.74
2.73
2.44
2.43
2.41
2.04
2.03
2.01
2.00
1.98
0.00
1.97
1.99
3.96
2.00
1.99
2.06
f1 (ppm)

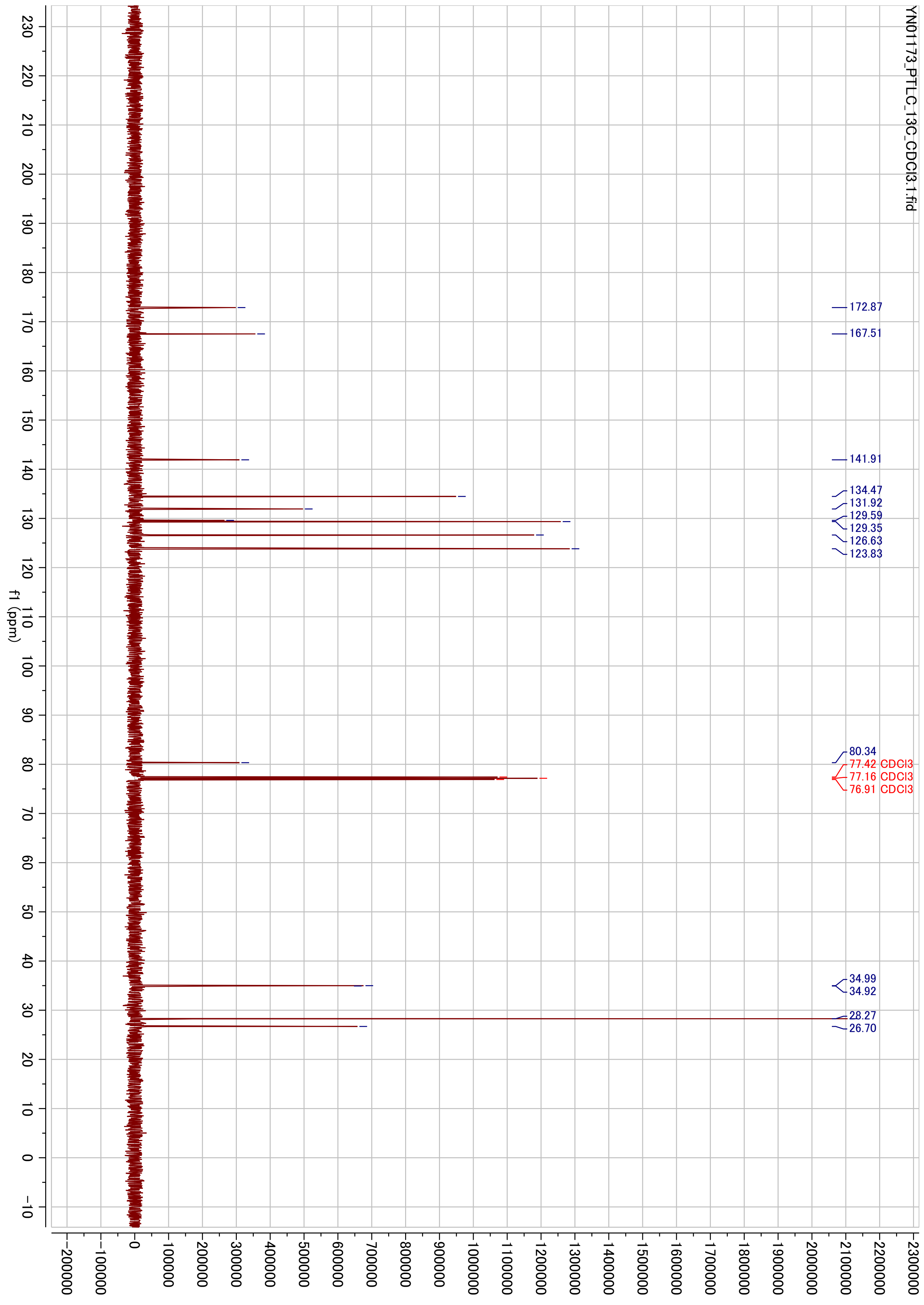
YN01173_PTLC_13C_CDCl3.1.fid
172.87
167.51
141.91
134.47
131.92
129.59
129.35
126.63
123.83
80.34
77.42 CDCl3
77.16 CDCl3
76.91 CDCl3
34.99
34.92
28.27
26.70
f1 (ppm)

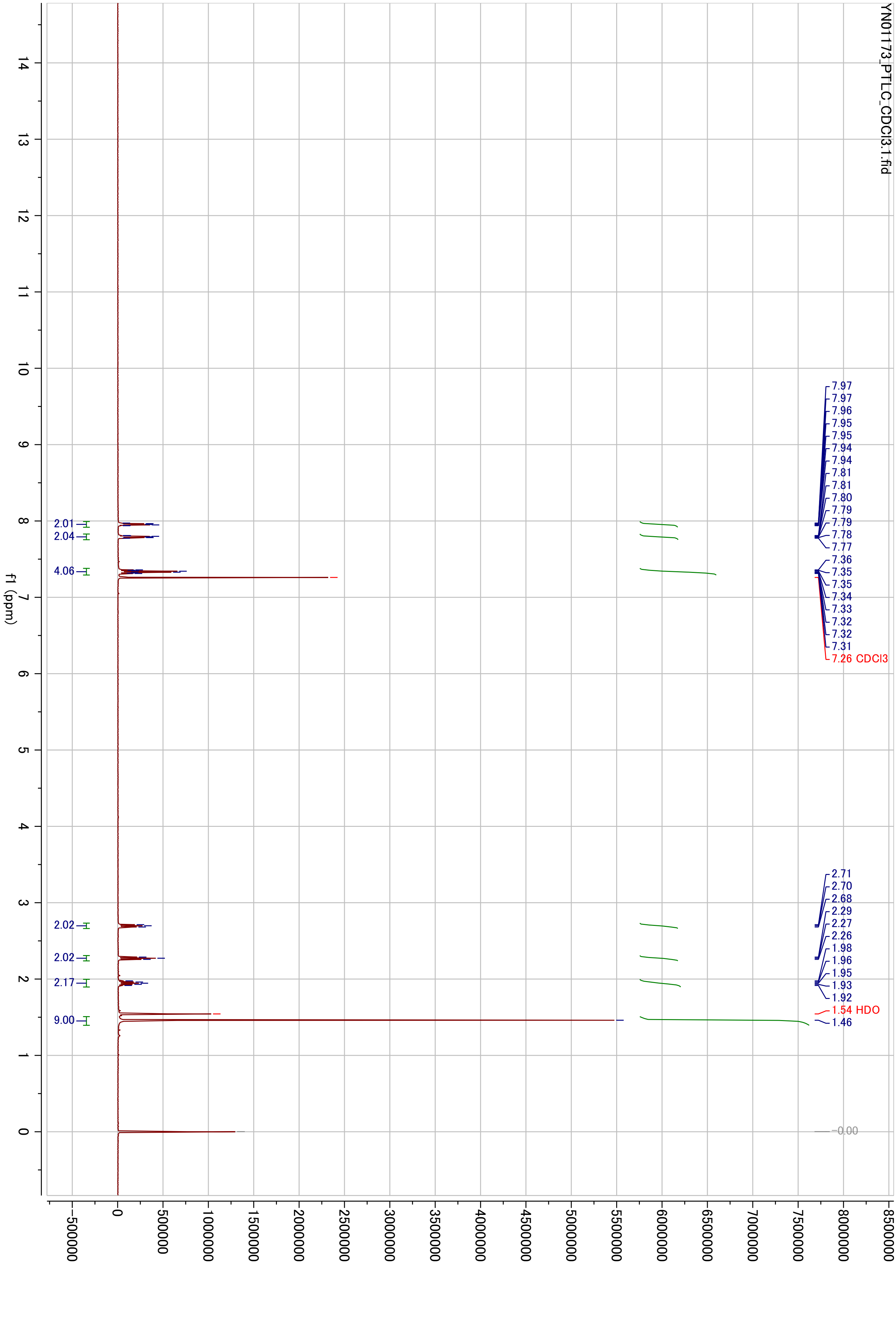
YN01173_PTLC_CDCl3.1.fid
7.97
7.97
7.96
7.95
7.95
7.94
7.94
7.81
7.81
7.80
7.79
7.79
7.78
7.77
7.36
7.35
7.35
7.34
7.33
7.32
7.32
7.31
7.26 CDCl3
2.71
2.70
2.68
2.29
2.27
2.26
1.98
1.96
1.95
1.93
1.92
1.54 HDO
1.46
-0.00
2.01
2.04
4.06
2.02
2.02
2.17
9.00
f1 (ppm)

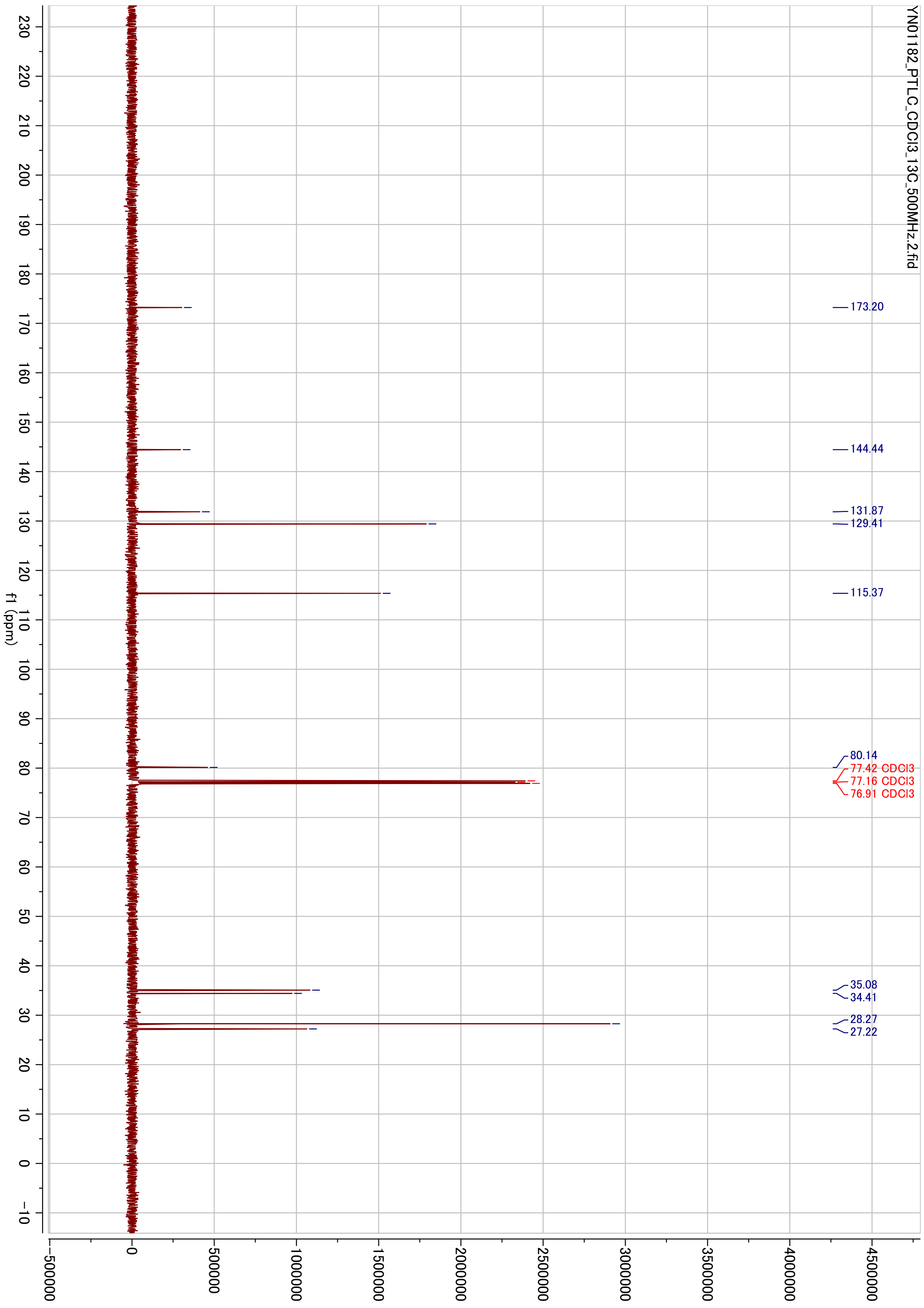
YN01182_PTLC_CDCl3_13C_500MHz.2.fid
173.20
144.44
131.87
129.41
115.37
80.14
77.42 CDCl3
77.16 CDCl3
76.91 CDCl3
35.08
34.41
28.27
27.22
f1 (ppm)

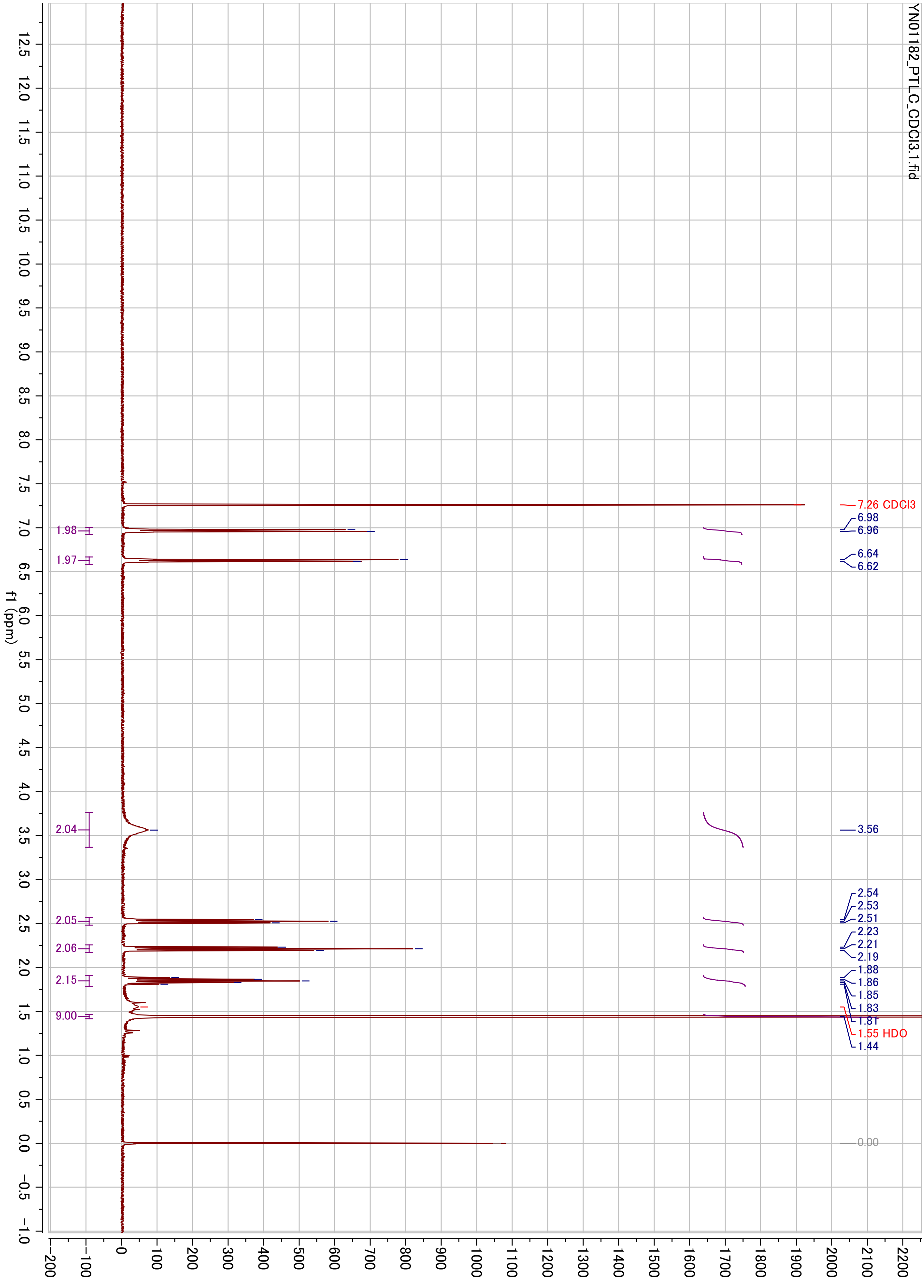
YN01182_PTLC_CDCl3.fid
7.26 CDCl3
6.98
6.96
6.64
6.62
3.56
2.54
2.53
2.51
2.23
2.21
2.19
1.88
1.86
1.85
1.83
1.81
1.55 HDO
1.44
0.00
1.98
1.97
2.04
2.05
2.06
2.15
9.00
f1 (ppm)

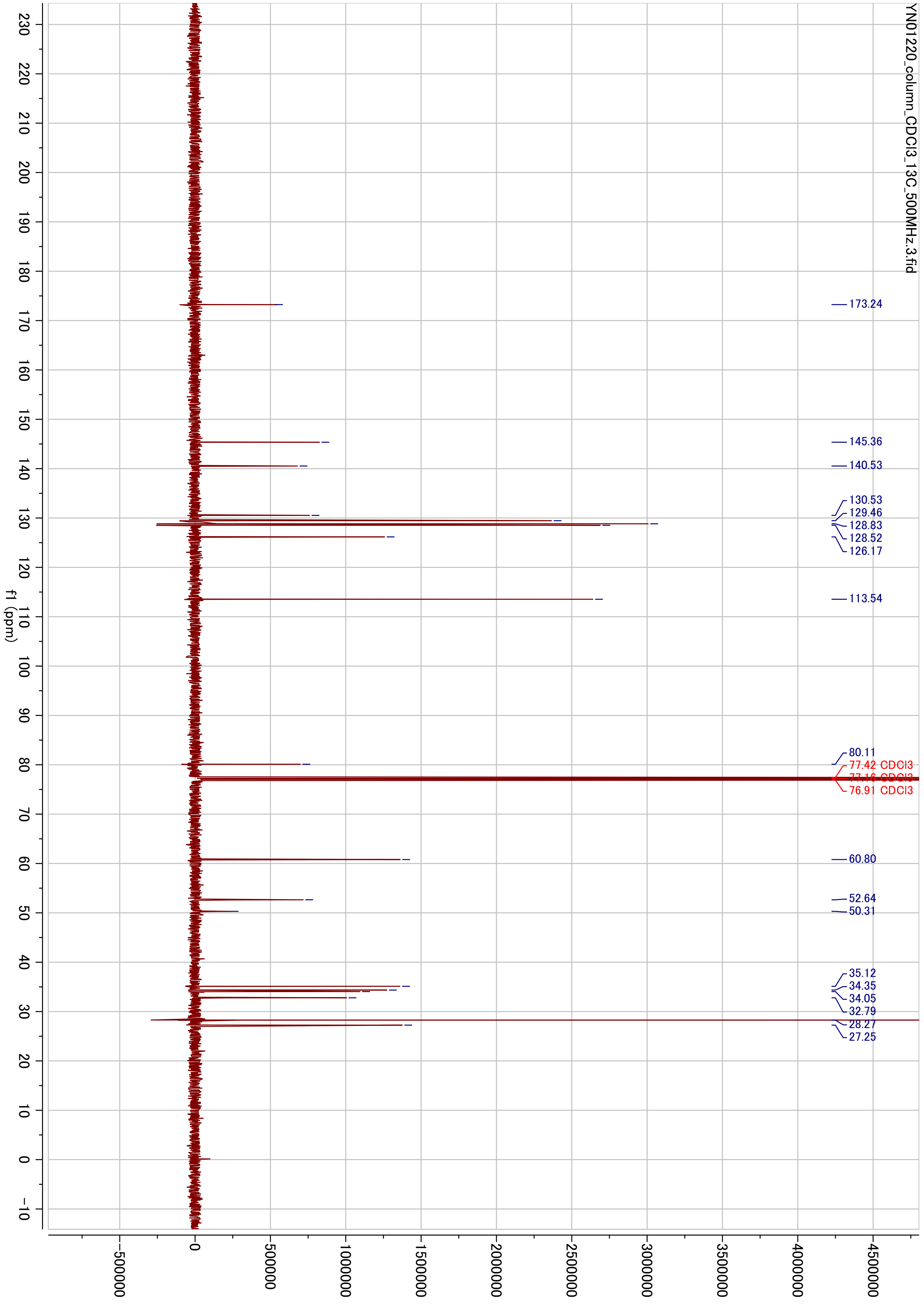
YN01220_column_CDCl3_13C_500MHz.3.fid
173.24
145.36
140.53
130.53
129.46
128.83
128.52
126.17
113.54
80.11
77.42 CDCl3
77.16 CDCl3
76.91 CDCl3
60.80
52.64
50.31
35.12
34.35
34.05
32.79
28.27
27.25
f1 (ppm)

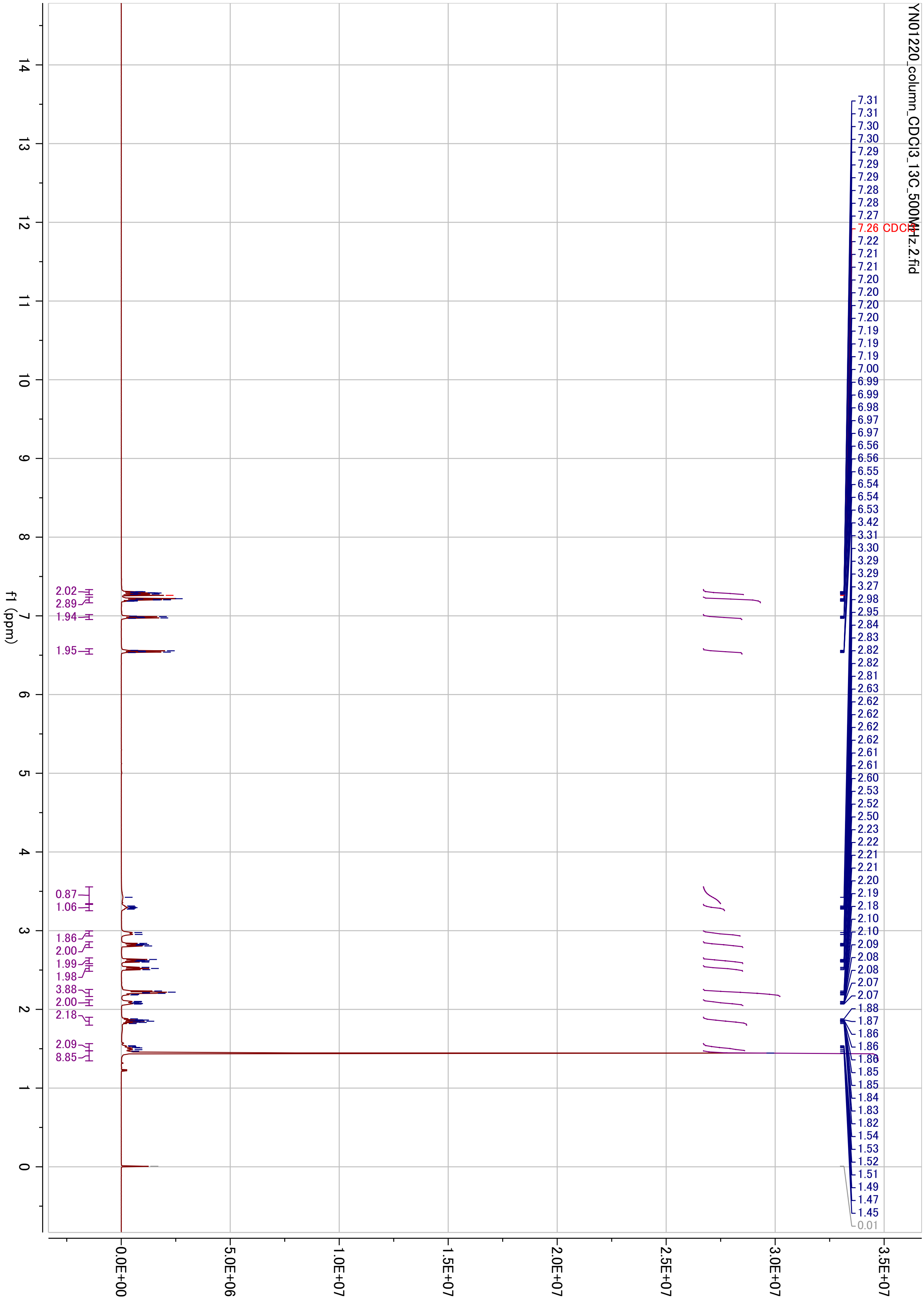
YN01220_column_CDCl3_13C_500MHz.2.fid
f1 (ppm)
7.31 7.31 7.30 7.30 7.29 7.29 7.29 7.28 7.28 7.27 7.26 CDCl3 7.22 7.21 7.21 7.20 7.20 7.20 7.20 7.19 7.19 7.19 7.00 6.99 6.99 6.98 6.97 6.97 6.56 6.56 6.55 6.54 6.54 6.53 3.42 3.31 3.30 3.29 3.29 3.27 2.98 2.95 2.84 2.83 2.82 2.82 2.81 2.63 2.62 2.62 2.62 2.62 2.61 2.61 2.60 2.53 2.52 2.50 2.23 2.22 2.21 2.21 2.20 2.19 2.18 2.10 2.10 2.09 2.08 2.08 2.07 2.07 1.88 1.87 1.86 1.86 1.86 1.85 1.85 1.84 1.83 1.82 1.54 1.53 1.52 1.51 1.49 1.47 1.45 0.01
2.02 2.89 1.94 1.95 0.87 1.06 1.86 2.00 1.99 1.98 3.88 2.00 2.18 2.09 8.85
14 13 12 11 10 9 8 7 6 5 4 3 2 1 0
0.0E+00 5.0E+06 1.0E+07 1.5E+07 2.0E+07 2.5E+07 3.0E+07 3.5E+07

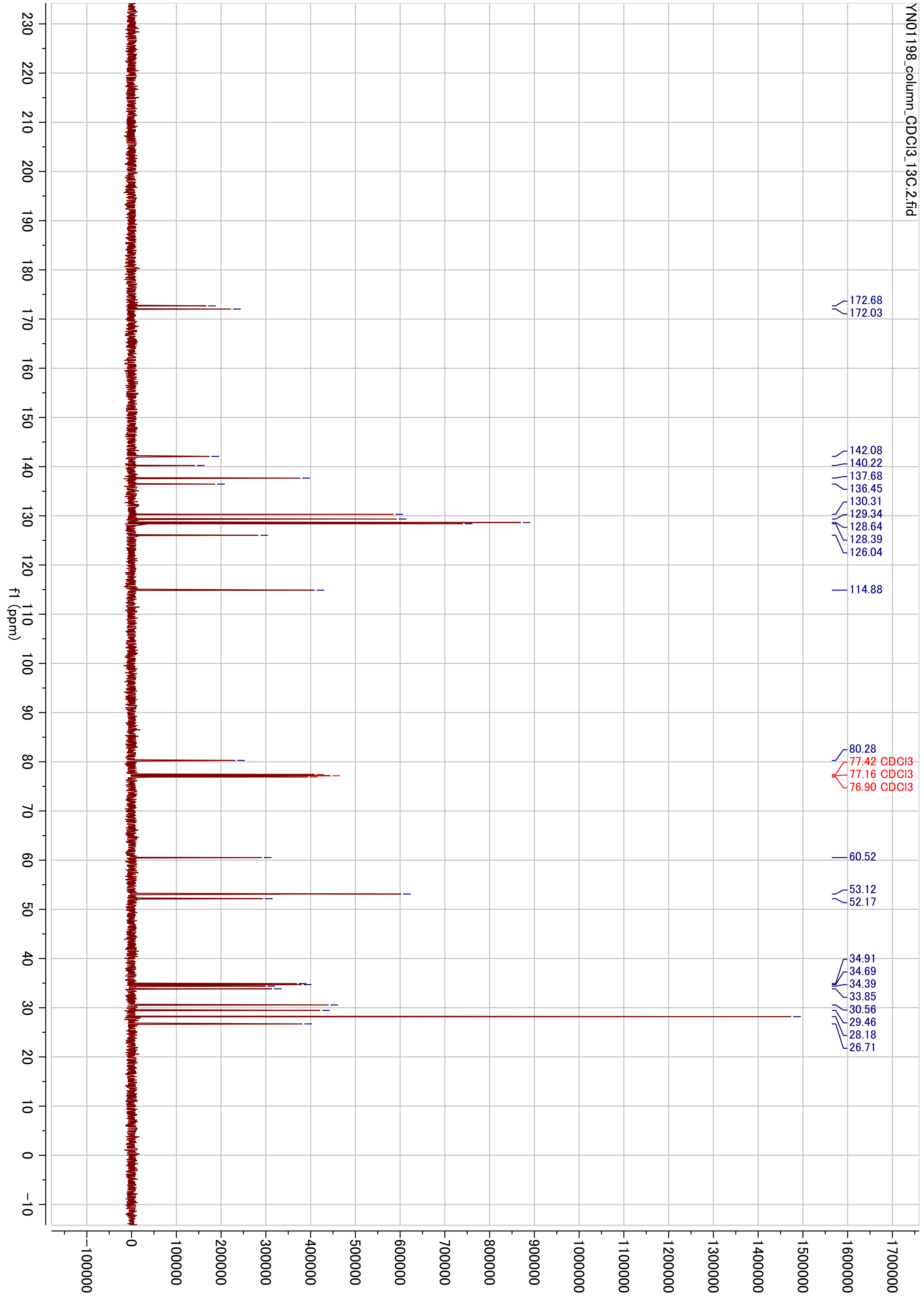
YN01198_column_CDCl3_13C.2.fid
172.68
172.03
142.08
140.22
137.68
136.45
130.31
129.34
128.64
128.39
126.04
114.88
80.28
77.42 CDCl3
77.16 CDCl3
76.90 CDCl3
60.52
53.12
52.17
34.91
34.69
34.39
33.85
30.56
29.46
28.18
26.71
f1 (ppm)

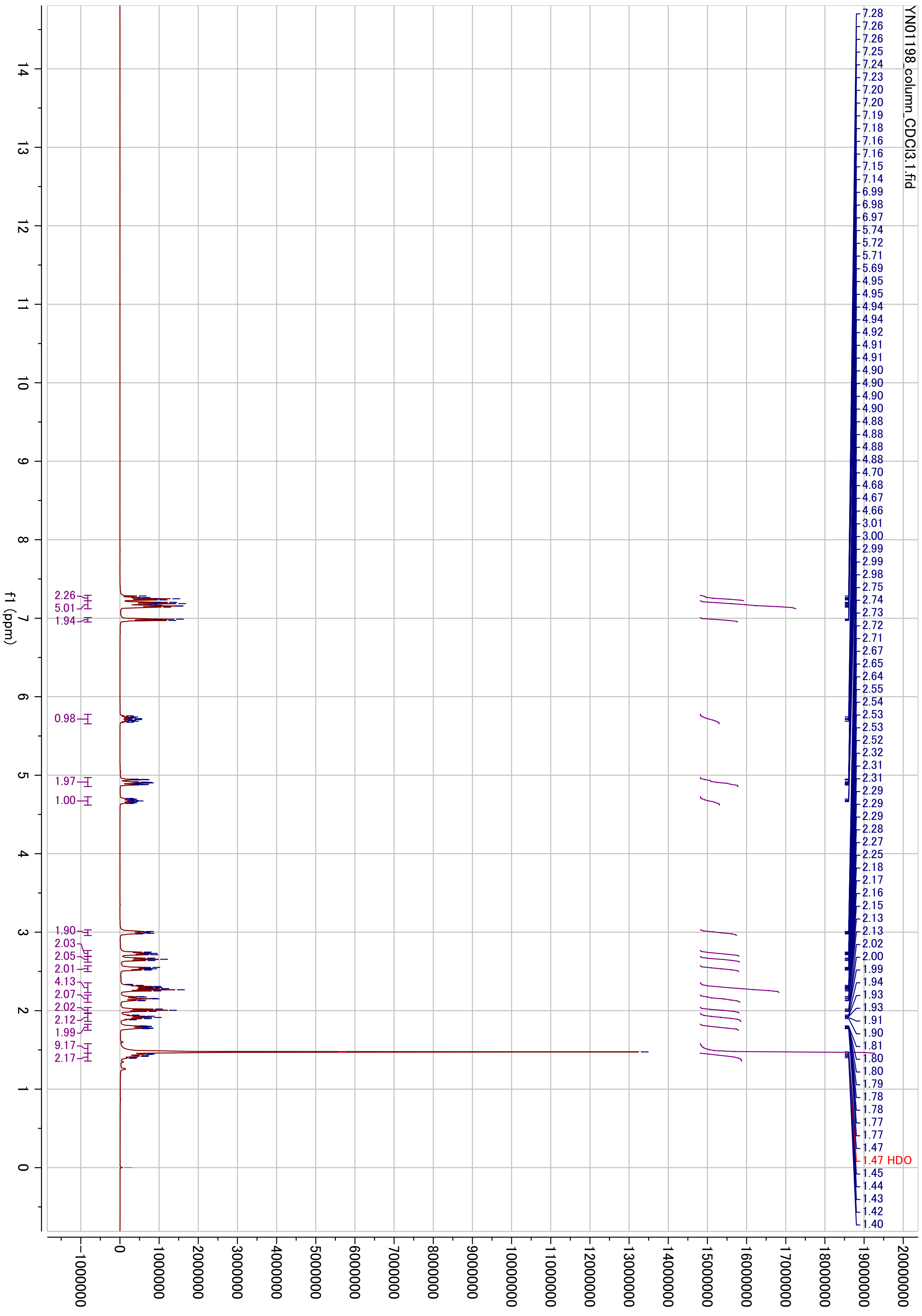
YN01198_column_CDCl3.1.fid
7.28
7.26
7.26
7.25
7.24
7.23
7.20
7.20
7.19
7.18
7.16
7.16
7.15
7.14
6.99
6.98
6.97
5.74
5.72
5.71
5.69
4.95
4.95
4.94
4.94
4.92
4.91
4.91
4.90
4.90
4.90
4.90
4.88
4.88
4.88
4.88
4.70
4.68
4.67
4.66
3.01
3.00
2.99
2.99
2.98
2.75
2.74
2.73
2.72
2.71
2.67
2.65
2.64
2.55
2.54
2.53
2.53
2.52
2.32
2.31
2.31
2.29
2.29
2.29
2.28
2.27
2.25
2.18
2.17
2.16
2.15
2.13
2.13
2.02
2.00
1.99
1.94
1.93
1.93
1.91
1.90
1.81
1.80
1.80
1.79
1.78
1.78
1.77
1.77
1.47
1.47 HDO
1.45
1.44
1.43
1.42
1.40
2.26
5.01
1.94
0.98
1.97
1.00
1.90
2.03
2.05
2.01
4.13
2.07
2.02
2.12
1.99
9.17
2.17
f1 (ppm)

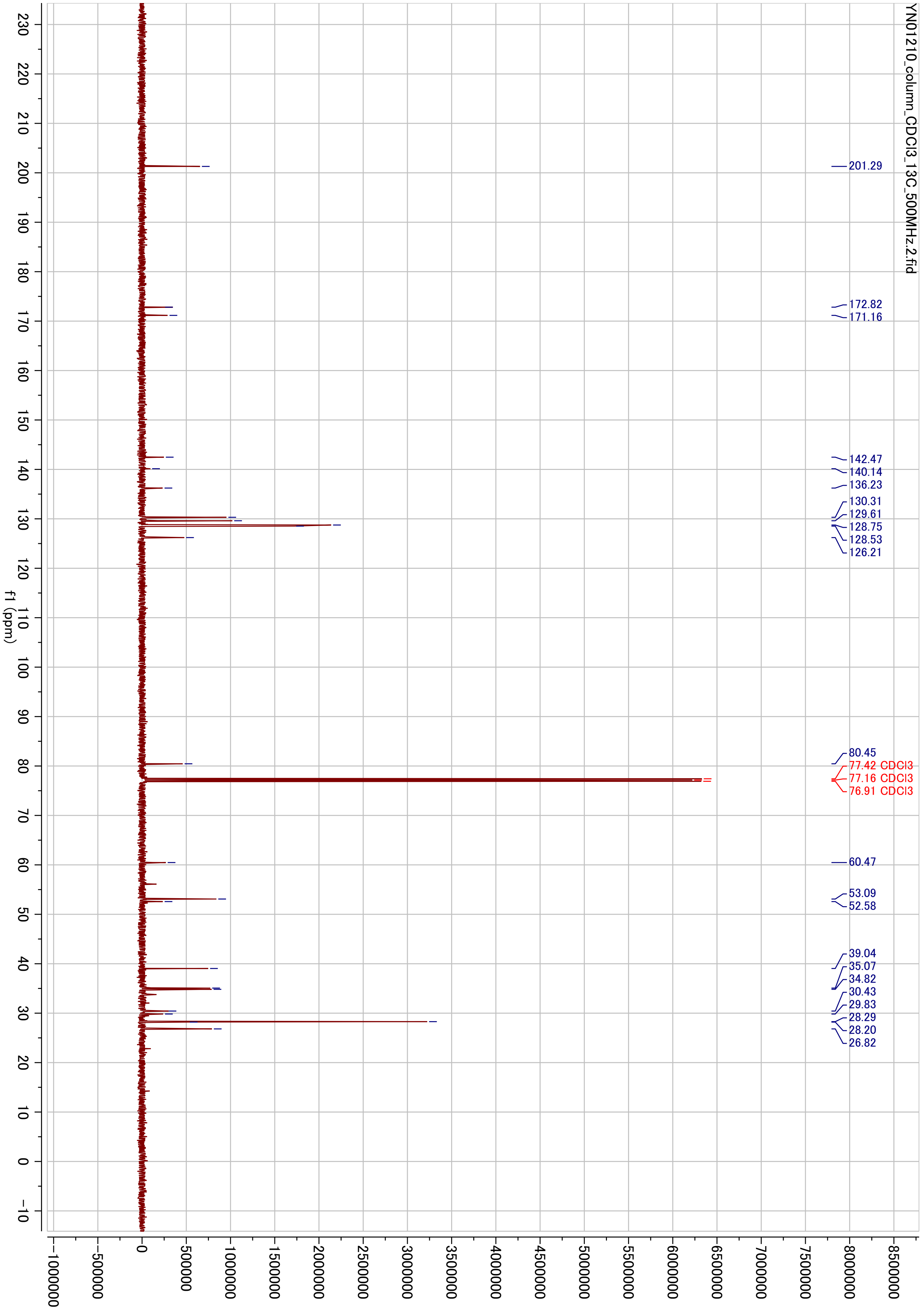
YN01210_column_CDCl3_13C_500MHz.2.fid
201.29
172.82
171.16
142.47
140.14
136.23
130.31
129.61
128.75
128.53
126.21
80.45
77.42 CDCl3
77.16 CDCl3
76.91 CDCl3
60.47
53.09
52.58
39.04
35.07
34.82
30.43
29.83
28.29
28.20
26.82
f1 (ppm)

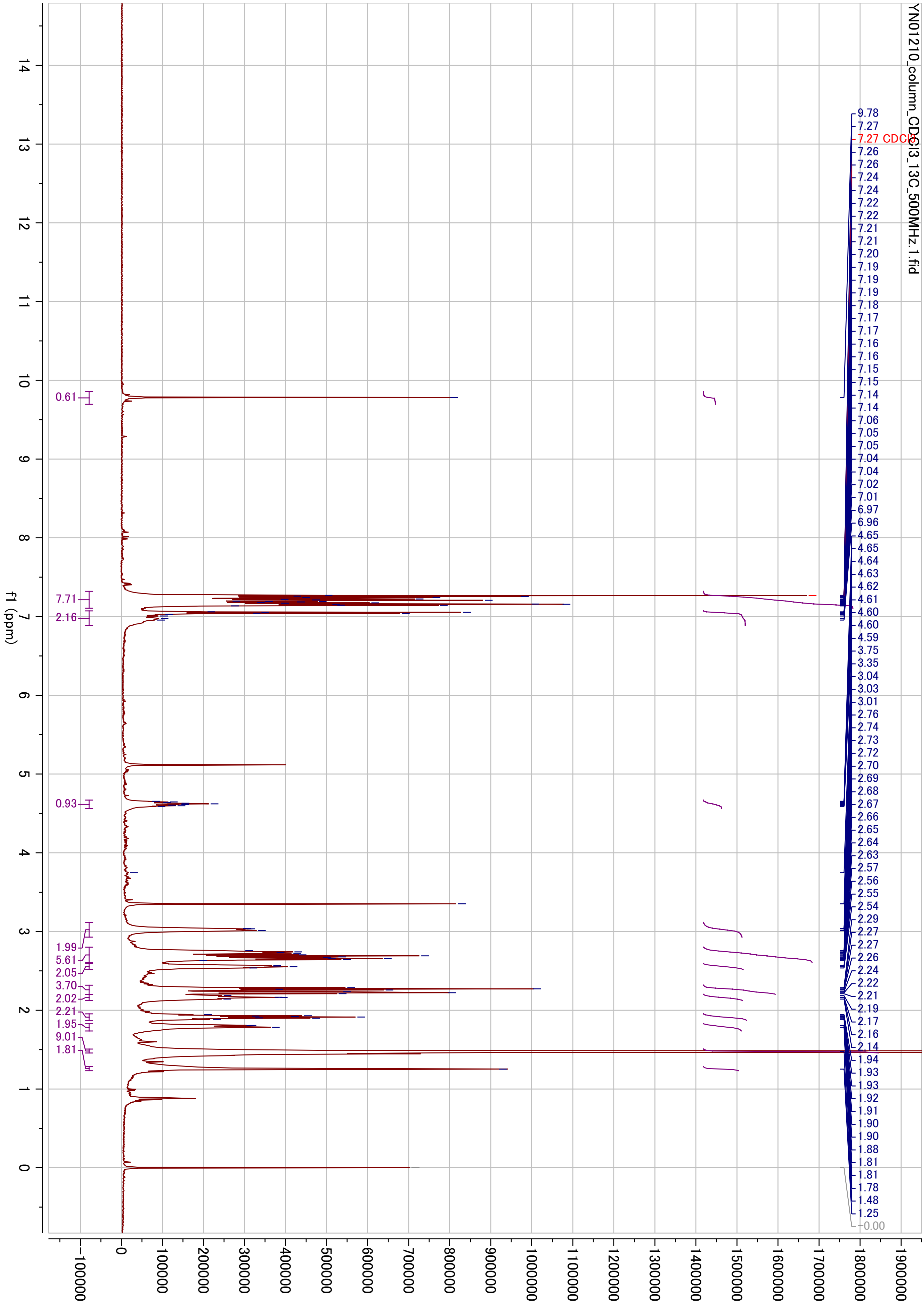
YN01210_column_CDCl3_13C_500MHz.1.fid
9.78
7.27
7.27 CDCl3
7.26
7.26
7.24
7.24
7.22
7.22
7.21
7.21
7.20
7.19
7.19
7.19
7.18
7.17
7.17
7.16
7.16
7.15
7.15
7.14
7.14
7.06
7.05
7.05
7.04
7.04
7.02
7.01
6.97
6.96
4.65
4.65
4.64
4.63
4.62
4.61
4.60
4.60
4.59
3.75
3.35
3.04
3.03
3.01
2.76
2.74
2.73
2.72
2.70
2.69
2.68
2.67
2.66
2.65
2.64
2.63
2.57
2.56
2.55
2.54
2.29
2.27
2.27
2.26
2.24
2.22
2.21
2.19
2.17
2.16
2.14
1.94
1.93
1.93
1.92
1.91
1.90
1.90
1.88
1.81
1.81
1.78
1.48
1.25
-0.00
0.61
7.71
2.16
0.93
1.99
5.61
2.05
3.70
2.02
2.21
1.95
9.01
1.81
f1 (ppm)

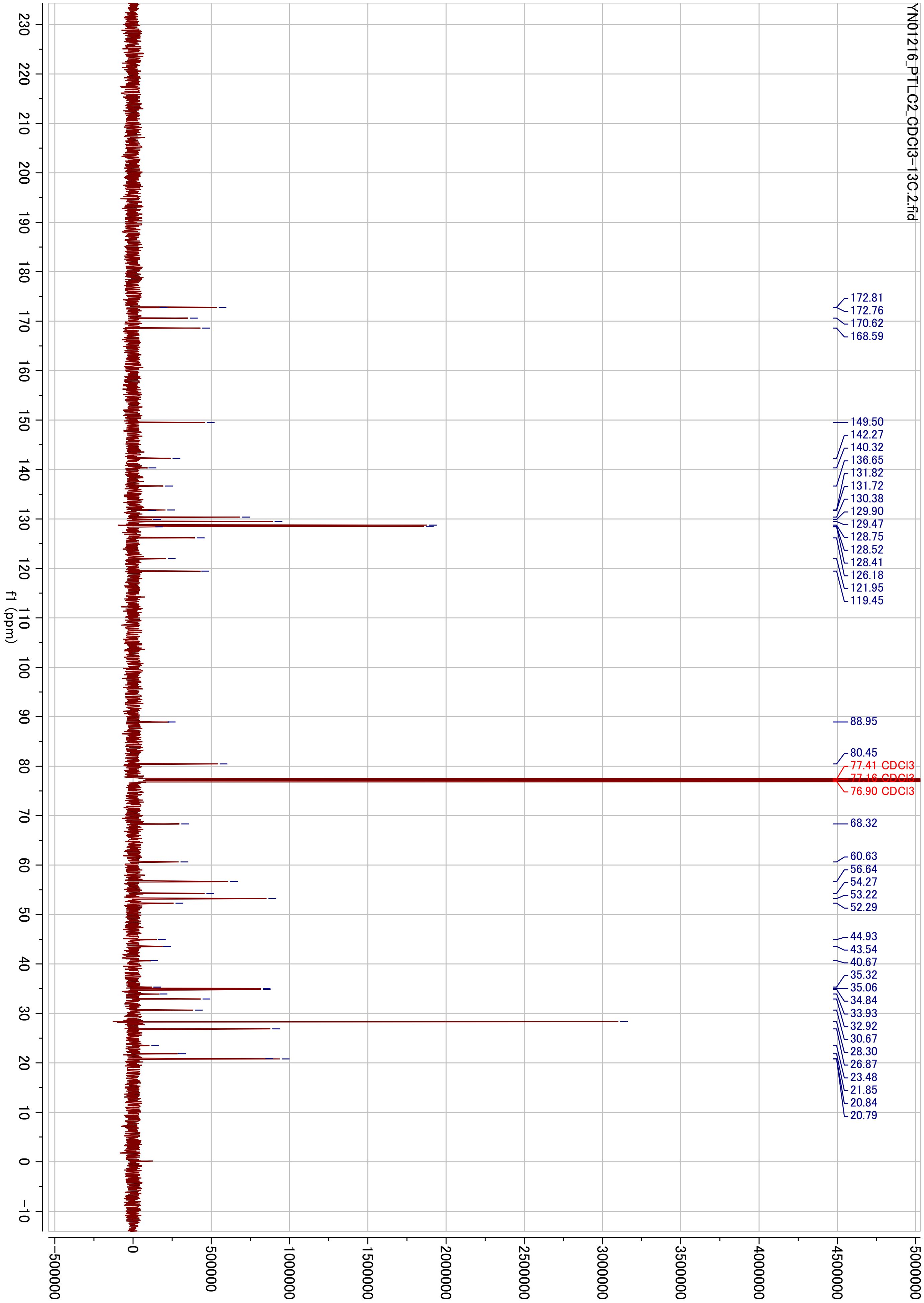
YN01216_PTLC2_CDCl3-13C.2.fid
172.81
172.76
170.62
168.59
149.50
142.27
140.32
136.65
131.82
131.72
130.38
129.90
129.47
128.75
128.52
128.41
126.18
121.95
119.45
88.95
80.45
77.41 CDCl3
77.16 CDCl3
76.90 CDCl3
68.32
60.63
56.64
54.27
53.22
52.29
44.93
43.54
40.67
35.32
35.06
34.84
33.93
32.92
30.67
28.30
26.87
23.48
21.85
20.84
20.79
f1 (ppm)

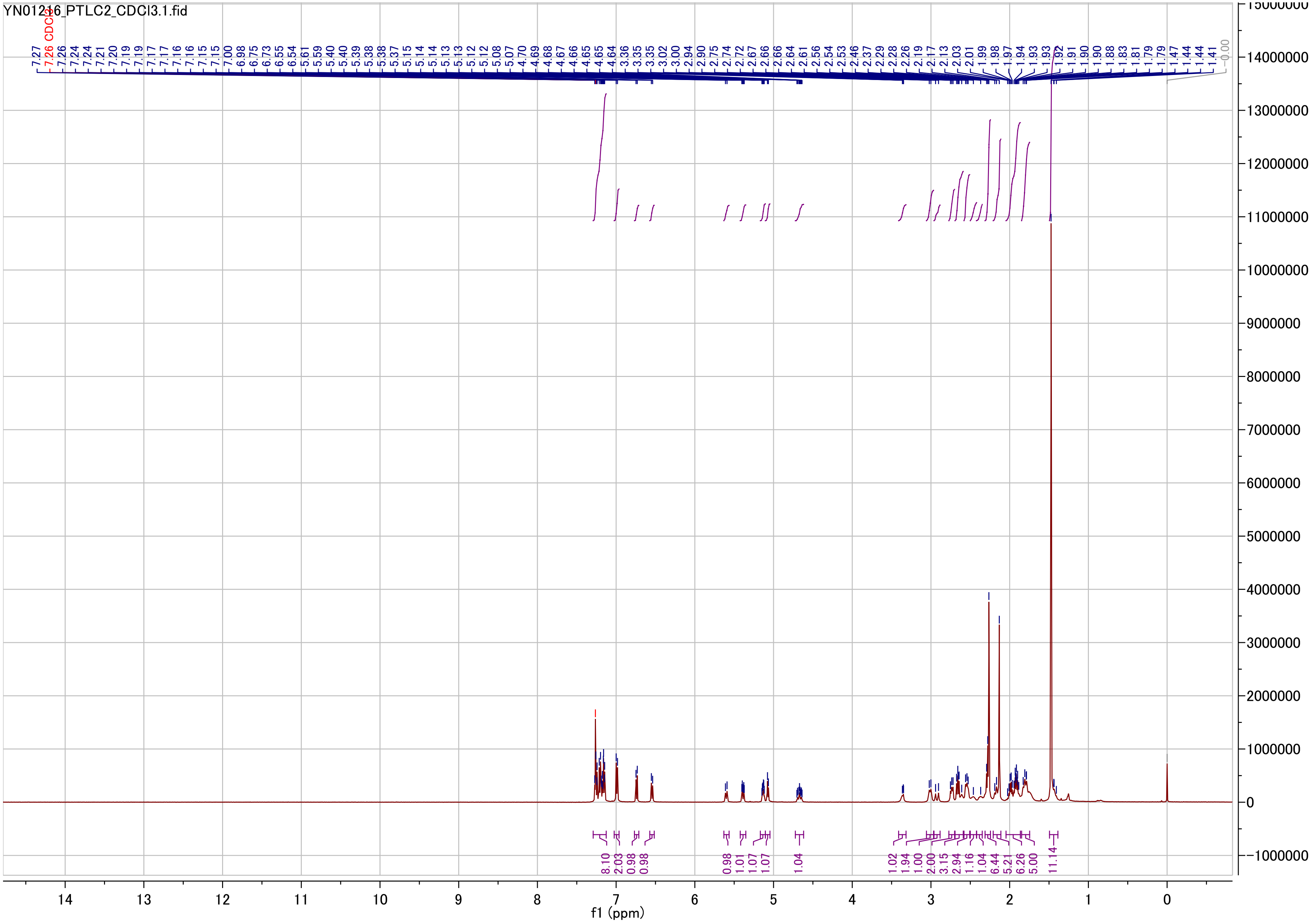
YN01216_PTLC2_CDCl3.1.fid
7.27 7.26 CDCl3 7.26 7.24 7.24 7.21 7.20 7.19 7.19 7.17 7.17 7.16 7.16 7.15 7.15 7.00 6.98 6.75 6.73 6.55 6.54 5.61 5.59 5.40 5.40 5.39 5.38 5.38 5.37 5.15 5.14 5.14 5.13 5.13 5.12 5.12 5.08 5.07 4.70 4.69 4.68 4.67 4.66 4.65 4.65 4.64 3.36 3.35 3.35 3.02 3.00 2.94 2.90 2.75 2.74 2.72 2.67 2.66 2.66 2.64 2.61 2.56 2.54 2.53 2.46 2.37 2.29 2.28 2.26 2.19 2.17 2.13 2.03 2.01 1.99 1.98 1.97 1.94 1.93 1.93 1.92 1.91 1.90 1.90 1.88 1.83 1.81 1.79 1.79 1.47 1.44 1.44 1.41 0.00
8.10 2.03 0.98 0.98 0.98 1.01 1.07 1.07 1.04 1.02 1.94 1.00 2.00 3.15 2.94 1.16 1.04 6.44 5.21 6.26 5.00 11.14
f1 (ppm)

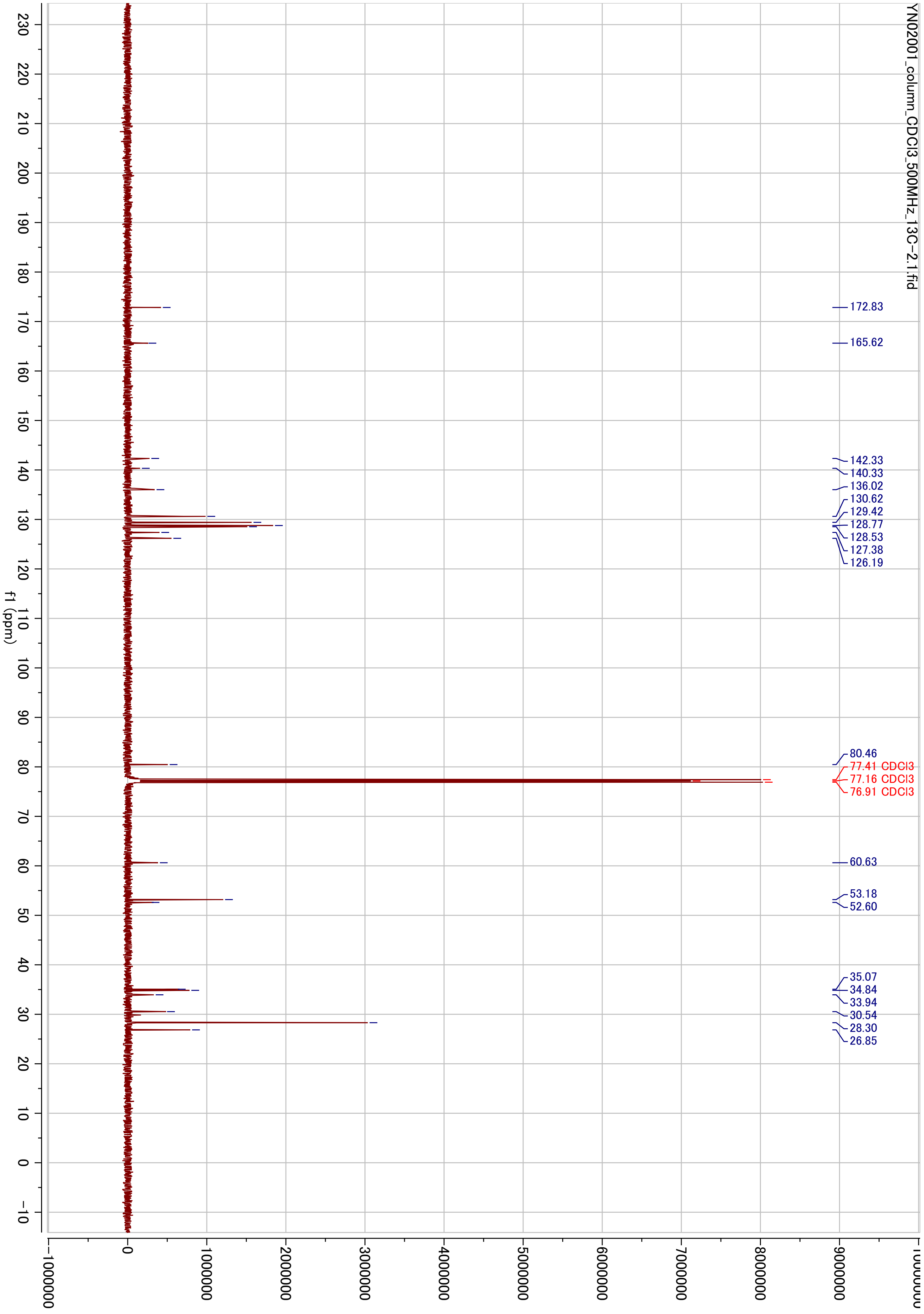

YN02001_column_CDCl3_500MHz_13C-2.1.fid
172.83
165.62
142.33
140.33
136.02
130.62
129.42
128.77
128.53
127.38
126.19
80.46
77.41 CDCl3
77.16 CDCl3
76.91 CDCl3
60.63
53.18
52.60
35.07
34.84
33.94
30.54
28.30
26.85
f1 (ppm)

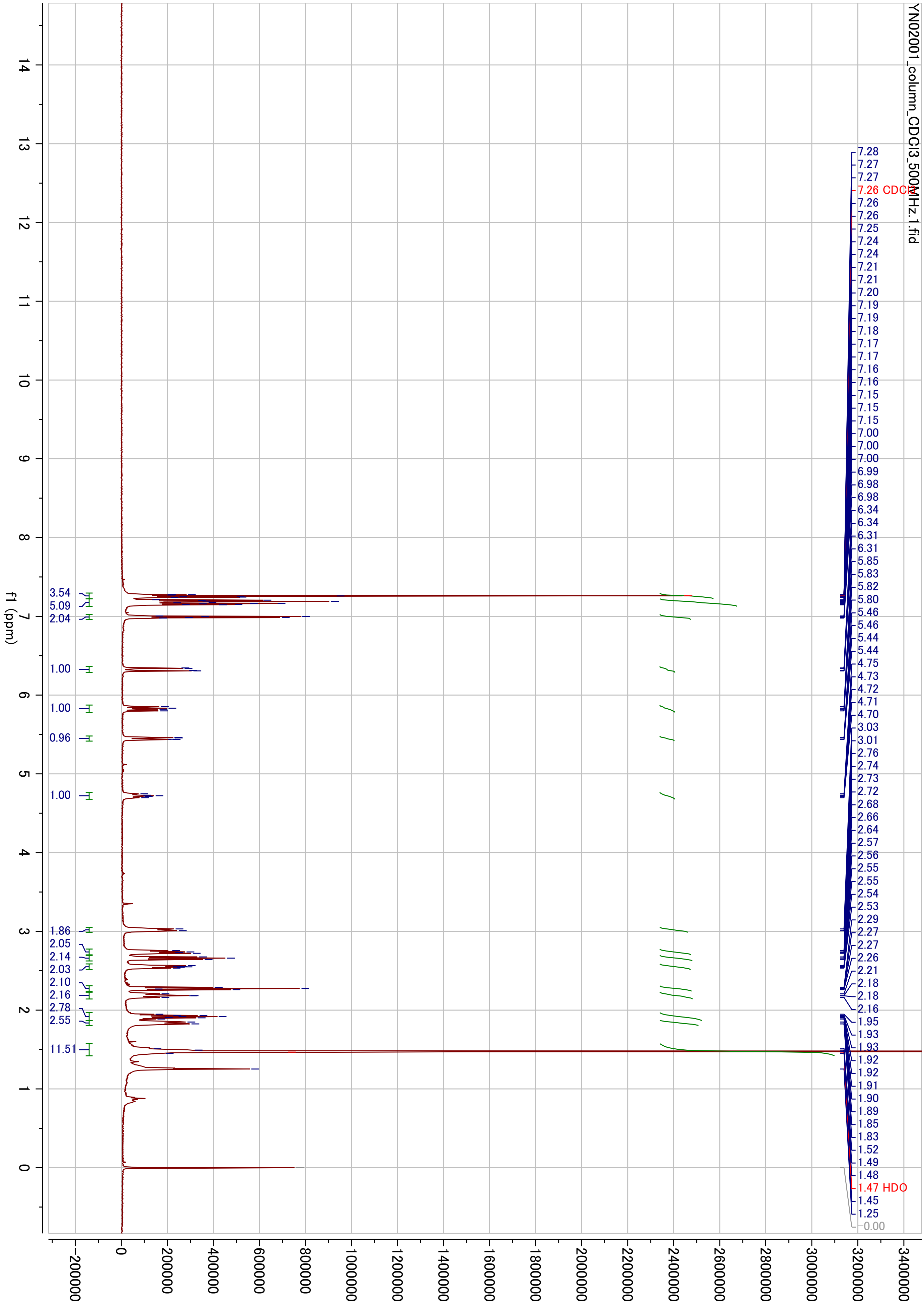
YN02001_column_CDCl3_500MHz.1.fid
7.28
7.27
7.27
7.26 CDCl3
7.26
7.26
7.25
7.24
7.24
7.21
7.21
7.20
7.19
7.19
7.18
7.17
7.17
7.16
7.16
7.15
7.15
7.15
7.00
7.00
7.00
6.99
6.98
6.98
6.34
6.34
6.31
6.31
5.85
5.83
5.82
5.80
5.46
5.46
5.44
5.44
4.75
4.73
4.72
4.71
4.70
3.03
3.01
2.76
2.74
2.73
2.72
2.68
2.66
2.64
2.57
2.56
2.55
2.55
2.54
2.53
2.29
2.27
2.27
2.26
2.21
2.18
2.18
2.16
1.95
1.93
1.93
1.92
1.92
1.91
1.90
1.89
1.85
1.83
1.52
1.49
1.48
1.47 HDO
1.45
1.25
0.00
3.54
5.09
2.04
1.00
1.00
0.96
1.00
1.86
2.05
2.14
2.03
2.10
2.16
2.78
2.55
11.51
f1 (ppm)

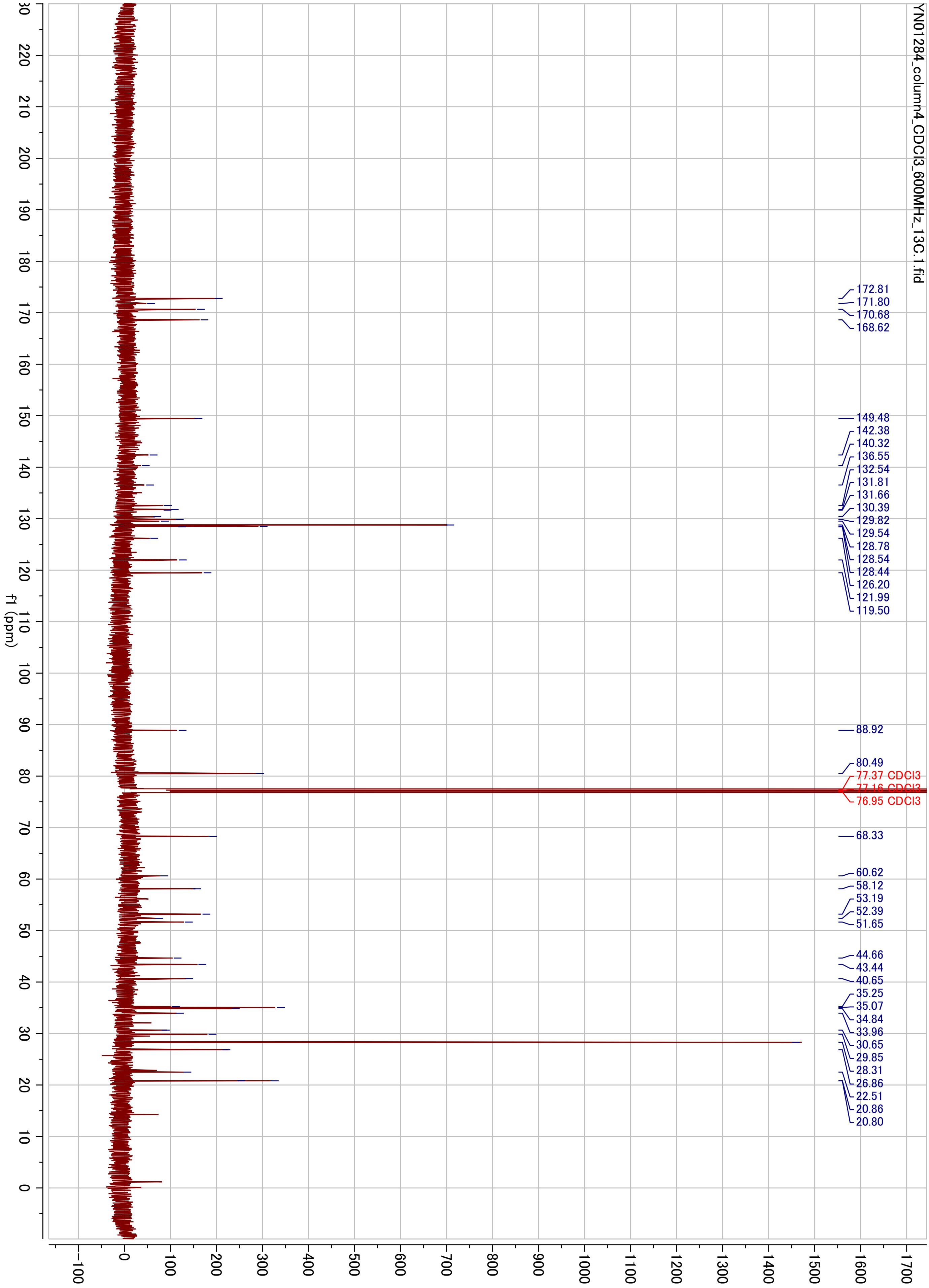
YN01284_column4_CDCl3_600MHz_13C.1.fid
172.81
171.80
170.68
168.62
149.48
142.38
140.32
136.55
132.54
131.81
131.66
130.39
129.82
129.54
128.78
128.54
128.44
126.20
121.99
119.50
88.92
80.49
77.37 CDCl3
77.16 CDCl3
76.95 CDCl3
68.33
60.62
58.12
53.19
52.39
51.65
44.66
43.44
40.65
35.25
35.07
34.84
33.96
30.65
29.85
28.31
26.86
22.51
20.86
20.80
f1 (ppm)

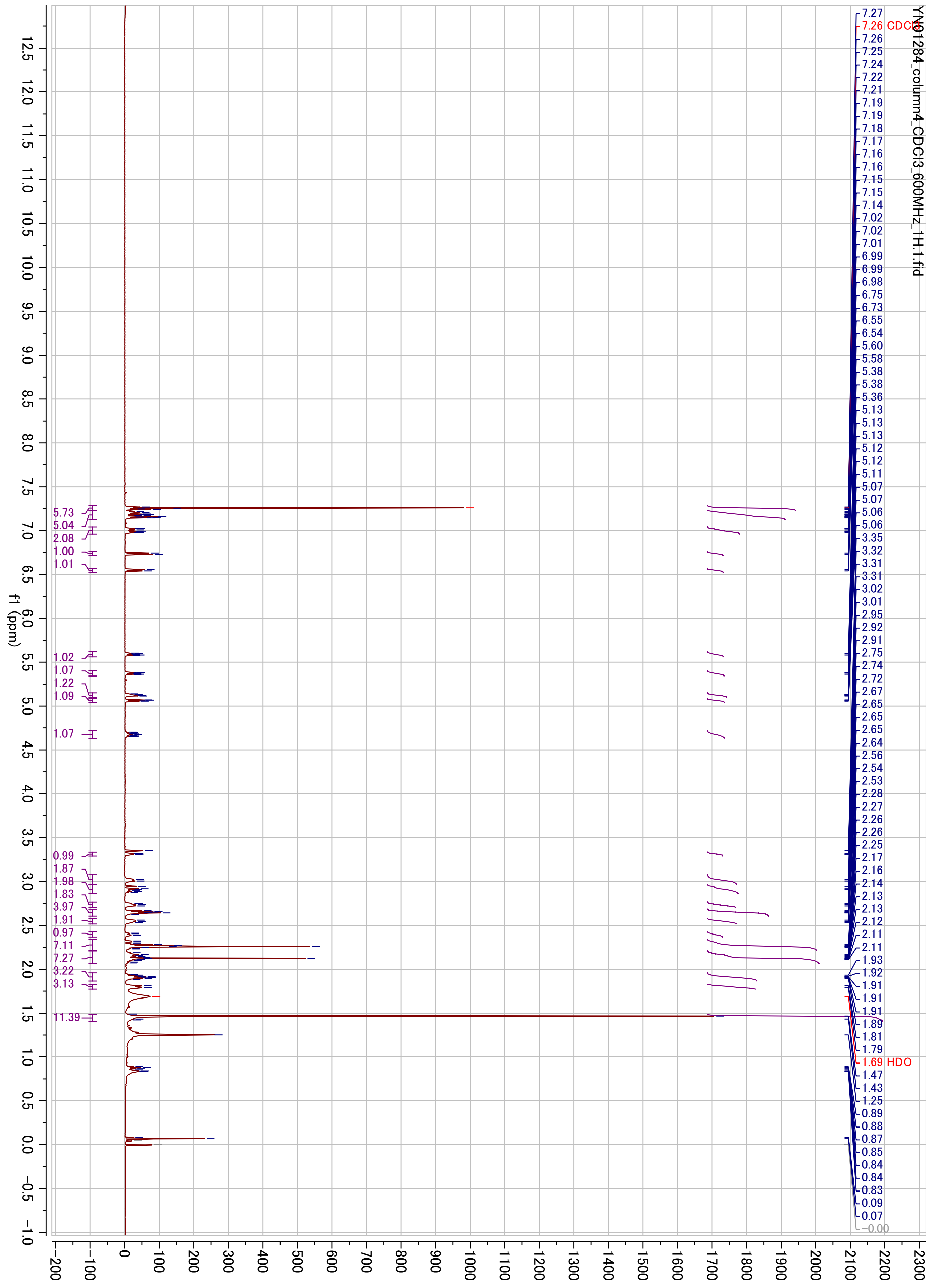
YN01284_column4_CDCl3_600MHz_1H.1.fid
f1 (ppm)
7.26 CDCl3
1.69 HDO

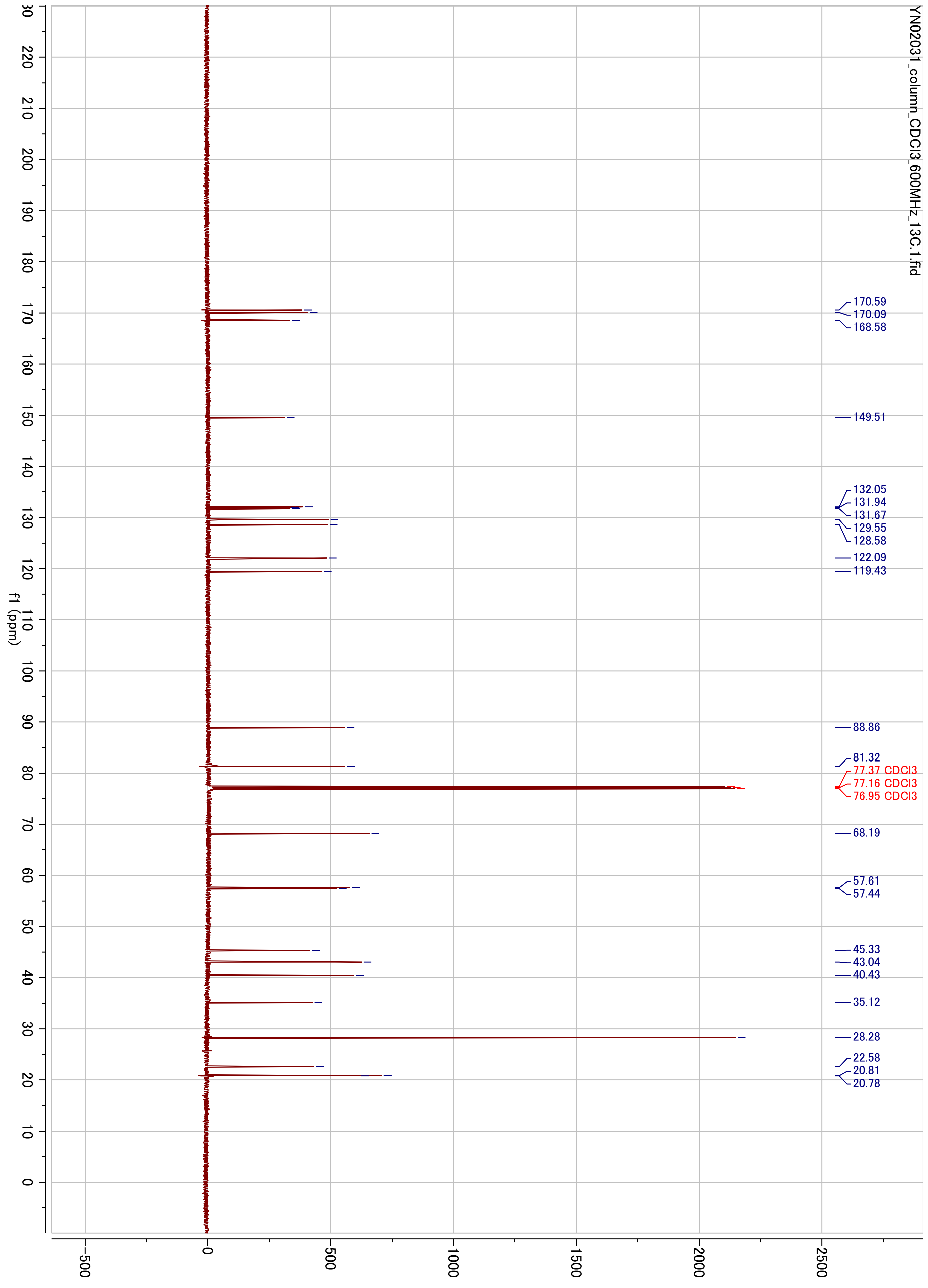

YN02031_column_CDCl3_600MHz_13C.1.fid
170.59
170.09
168.58
149.51
132.05
131.94
131.67
129.55
128.58
122.09
119.43
88.86
81.32
77.37 CDCl3
77.16 CDCl3
76.95 CDCl3
68.19
57.61
57.44
45.33
43.04
40.43
35.12
28.28
22.58
20.81
20.78
f1 (ppm)

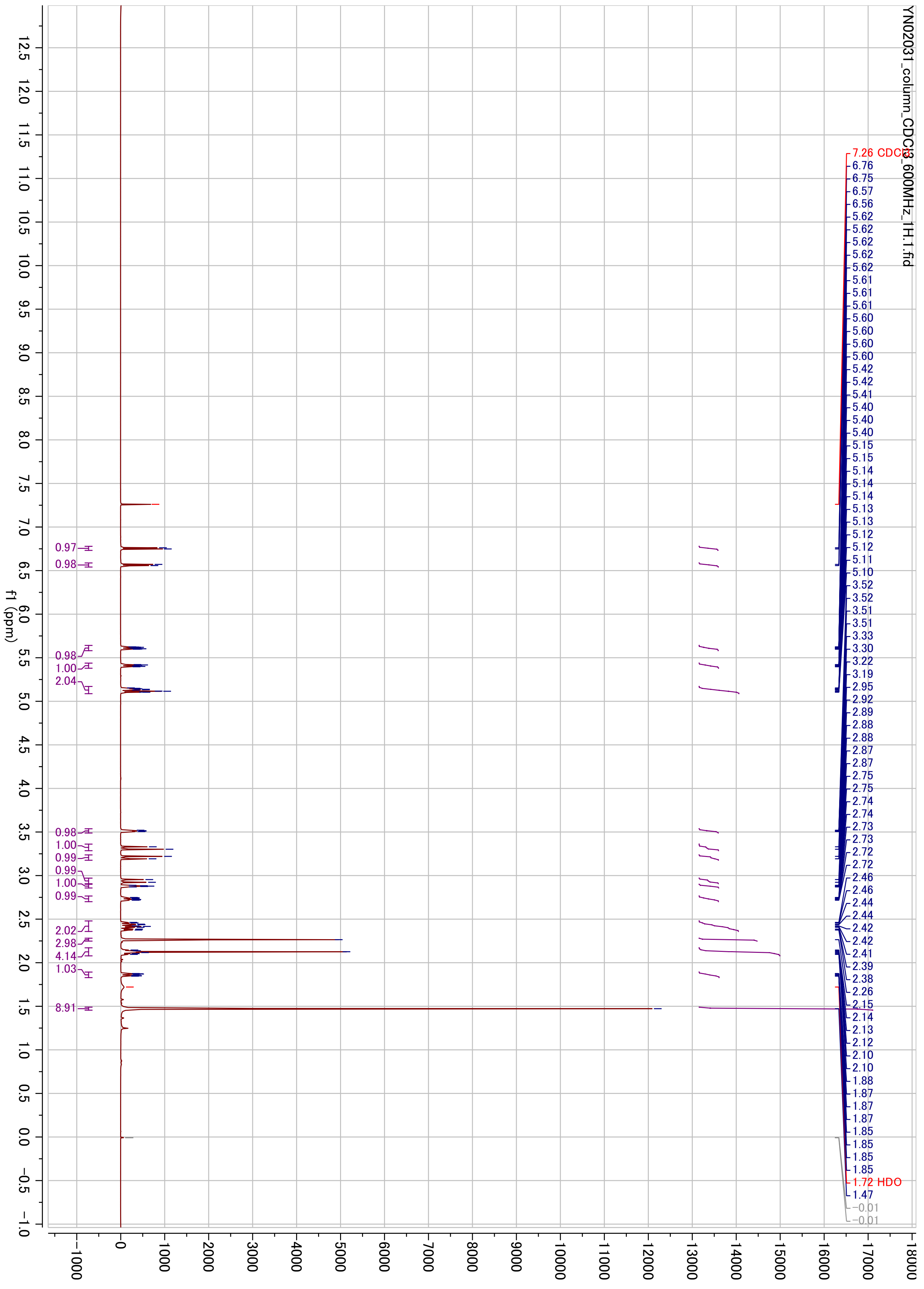

YN02031_column_CDCl3_600MHz_1H.1.fid
f1 (ppm)
7.26 CDCl3
6.76
6.75
6.57
6.56
5.62
5.62
5.62
5.62
5.62
5.61
5.61
5.61
5.60
5.60
5.60
5.60
5.42
5.42
5.41
5.40
5.40
5.40
5.15
5.15
5.14
5.14
5.14
5.13
5.13
5.12
5.12
5.11
5.10
3.52
3.52
3.51
3.51
3.33
3.30
3.22
3.19
2.95
2.92
2.89
2.88
2.88
2.87
2.87
2.75
2.75
2.74
2.74
2.73
2.73
2.72
2.72
2.46
2.46
2.44
2.44
2.42
2.42
2.41
2.39
2.38
2.26
2.15
2.14
2.13
2.12
2.10
2.10
1.88
1.87
1.87
1.87
1.85
1.85
1.85
1.85
1.72 HDO
1.47
−0.01
−0.01
0.97
0.98
0.98
1.00
2.04
0.98
1.00
0.99
0.99
1.00
0.99
2.02
2.98
4.14
1.03
8.91

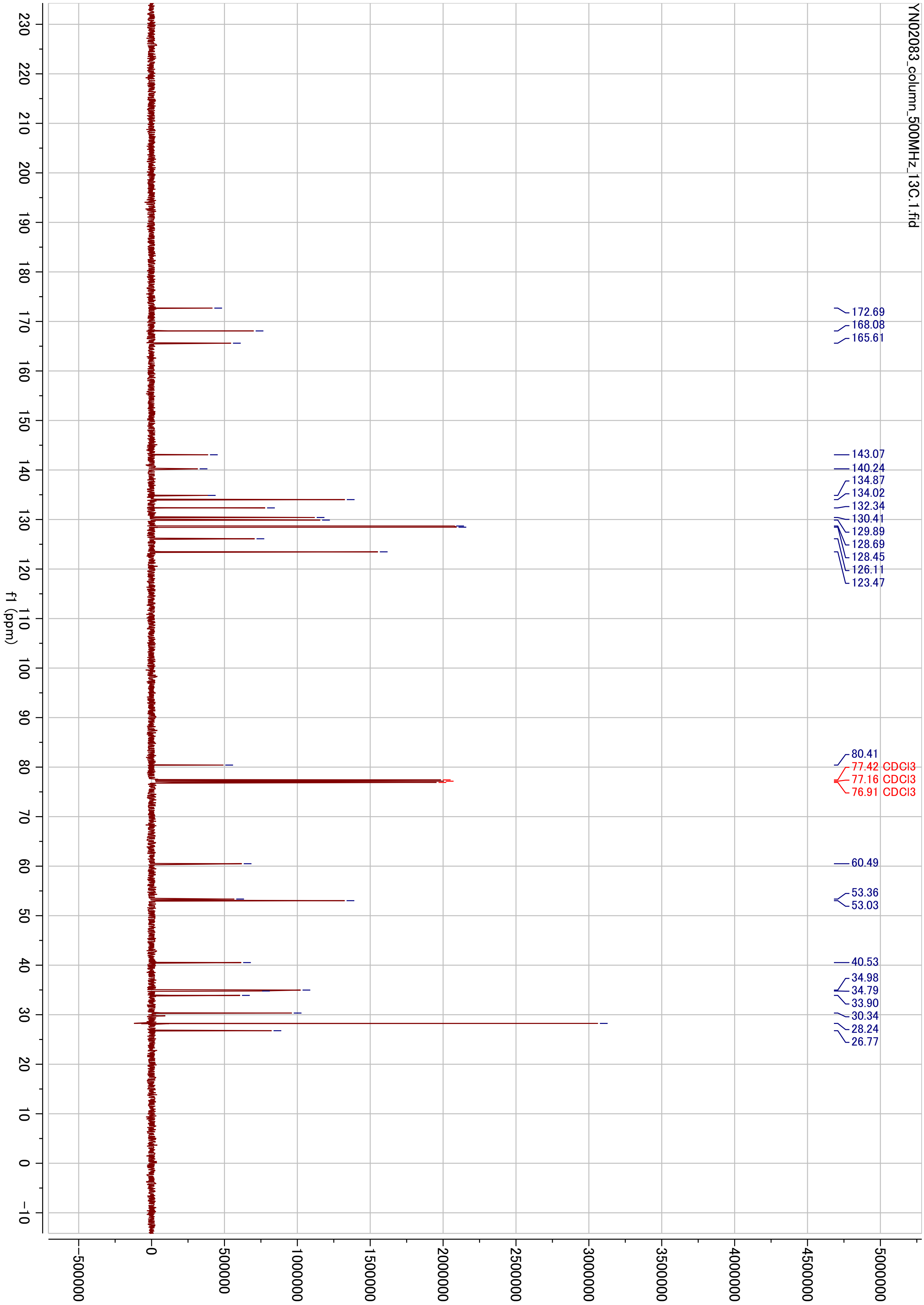
YN02083_column_500MHz_13C.1.fid
172.69
168.08
165.61
143.07
140.24
134.87
134.02
132.34
130.41
129.89
128.69
128.45
126.11
123.47
80.41
77.42 CDCl3
77.16 CDCl3
76.91 CDCl3
60.49
53.36
53.03
40.53
34.98
34.79
33.90
30.34
28.24
26.77
f1 (ppm)

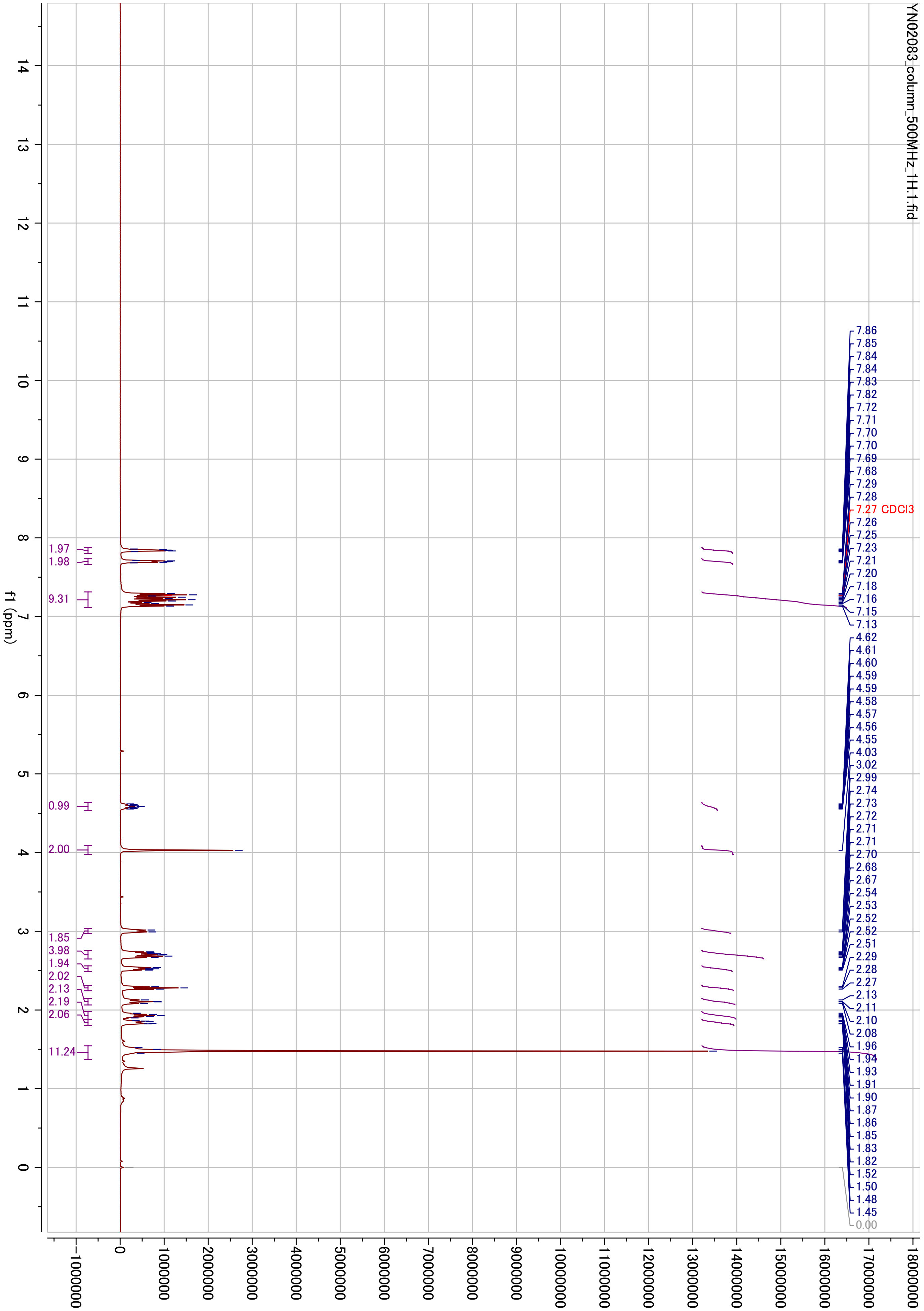
YN02083_column_500MHz_1H.1.fid
7.86
7.85
7.84
7.84
7.83
7.82
7.72
7.71
7.70
7.70
7.69
7.68
7.29
7.28
7.27 CDCl3
7.26
7.25
7.23
7.21
7.20
7.18
7.16
7.15
7.13
4.62
4.61
4.60
4.59
4.59
4.58
4.57
4.56
4.55
4.03
3.02
2.99
2.74
2.73
2.72
2.71
2.71
2.70
2.68
2.67
2.54
2.53
2.52
2.52
2.51
2.29
2.28
2.27
2.13
2.11
2.10
2.08
1.96
1.94
1.93
1.91
1.90
1.87
1.86
1.85
1.83
1.82
1.52
1.50
1.48
1.45
0.00
1.97
1.98
9.31
0.99
2.00
1.85
3.98
1.94
2.02
2.13
2.19
2.06
11.24
f1 (ppm)

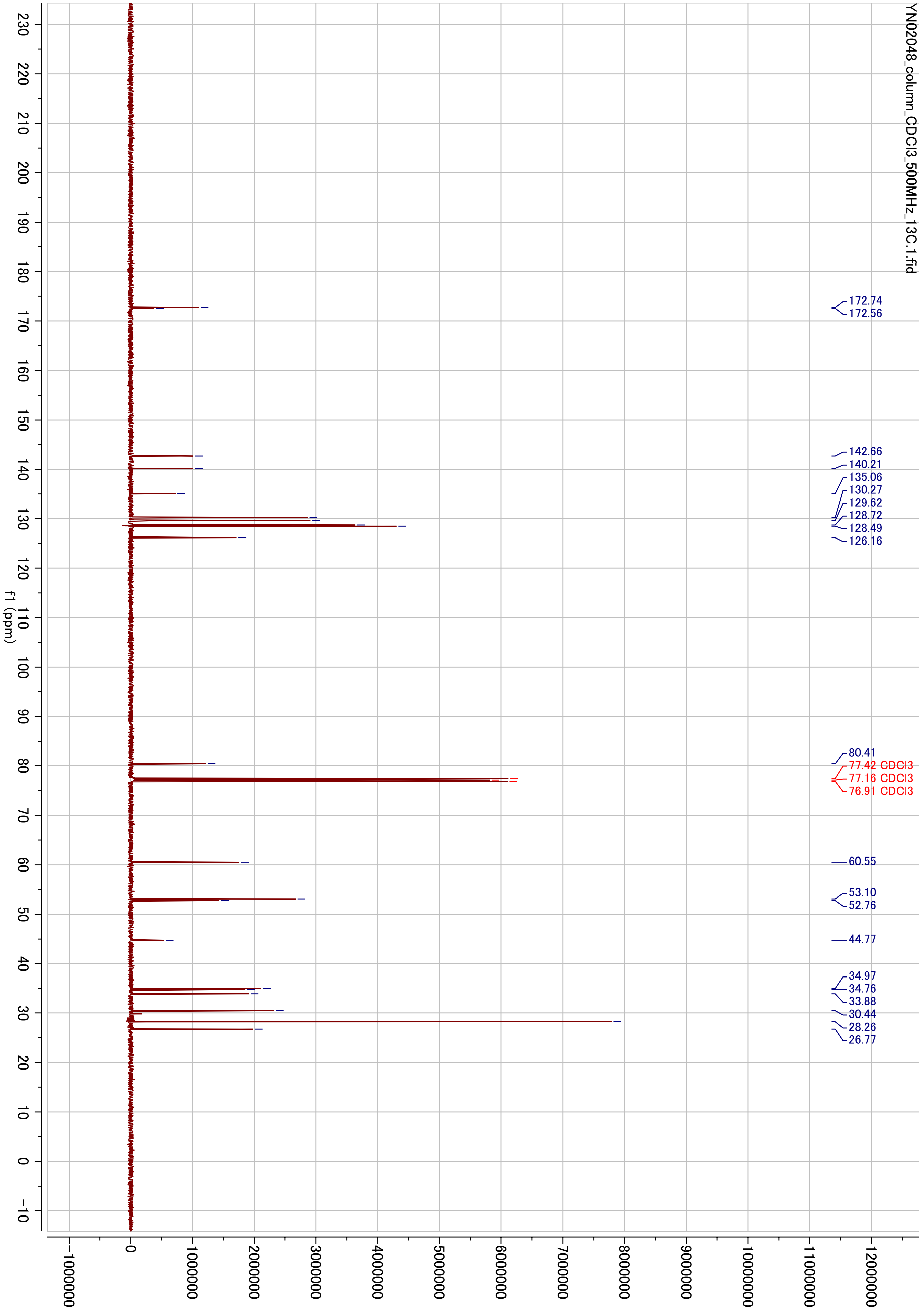
YN02048_column_CDCl3_500MHz_13C.1.fid
172.74
172.56
142.66
140.21
135.06
130.27
129.62
128.72
128.49
126.16
80.41
77.42 CDCl3
77.16 CDCl3
76.91 CDCl3
60.55
53.10
52.76
44.77
34.97
34.76
33.88
30.44
28.26
26.77
f1 (ppm)

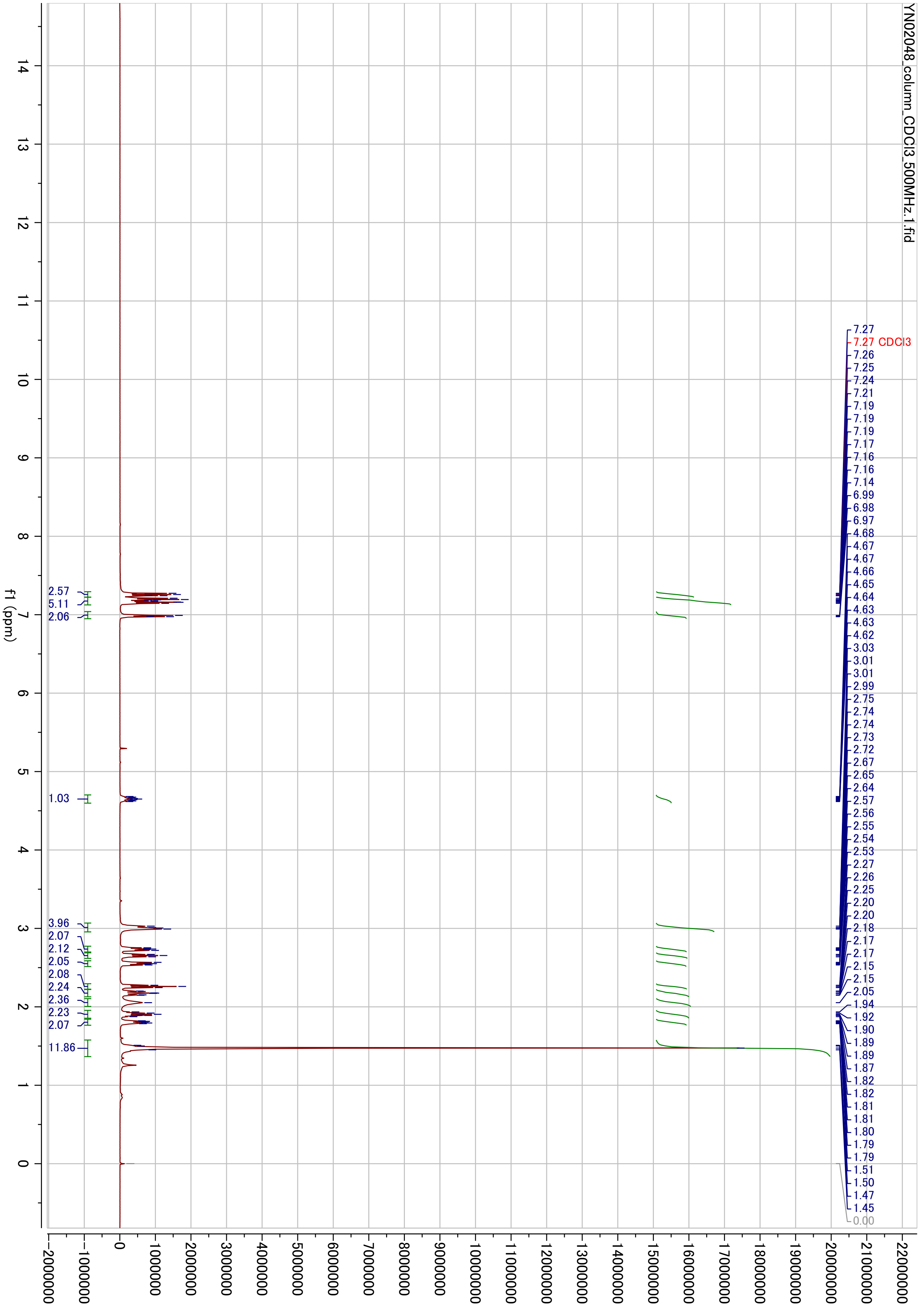
YN02048_column_CDCl3_500MHz.1.fid
f1 (ppm)
7.27 CDCl3
2.57
5.11
2.06
1.03
3.96
2.07
2.12
2.05
2.08
2.24
2.36
2.23
2.07
11.86

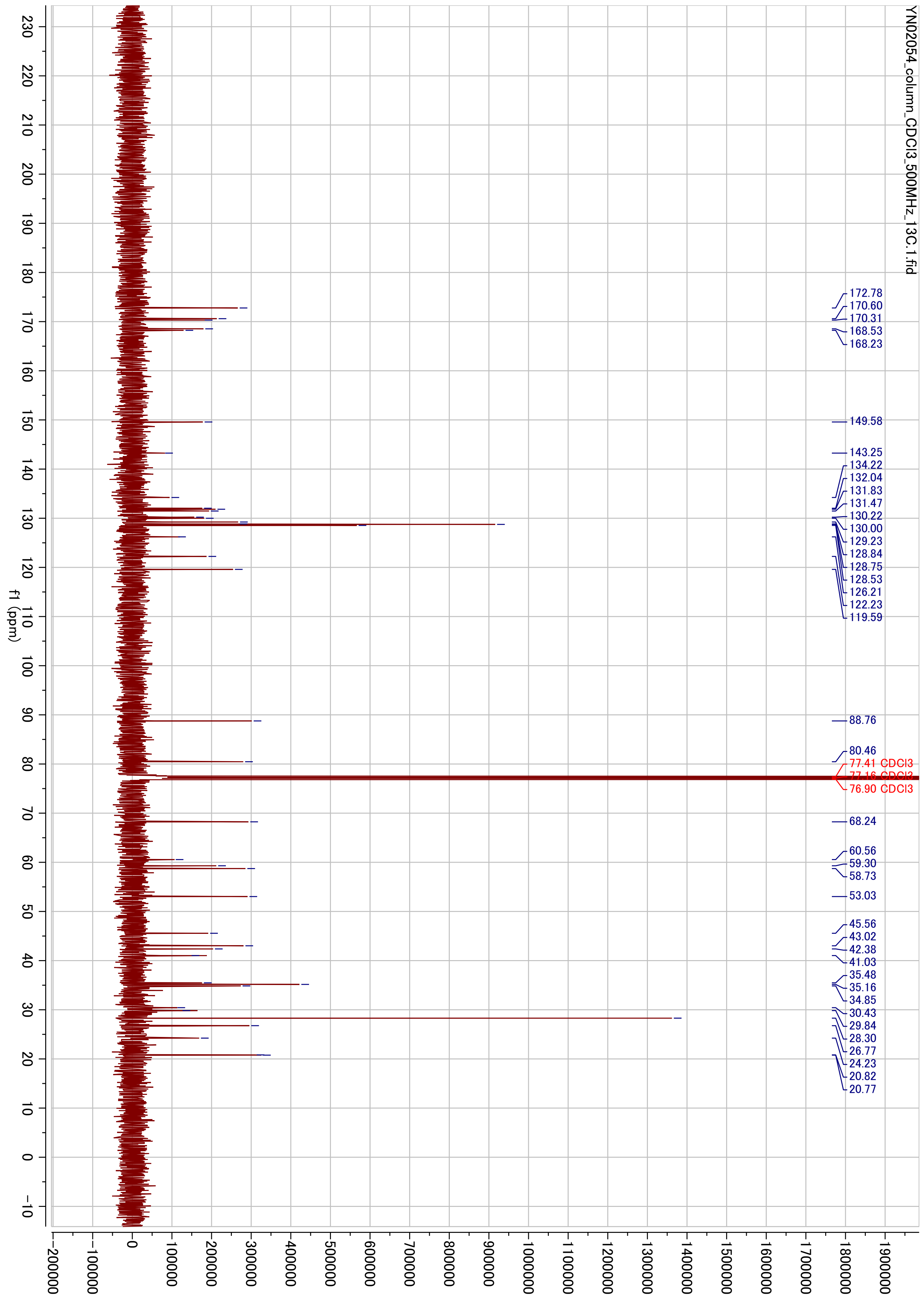
YN02054_column_CDCl3_500MHz_13C.1.fid
172.78
170.60
170.31
168.53
168.23
149.58
143.25
134.22
132.04
131.83
131.47
130.22
130.00
129.23
128.84
128.75
128.53
126.21
122.23
119.59
88.76
80.46
77.41 CDCl3
77.16 CDCl3
76.90 CDCl3
68.24
60.56
59.30
58.73
53.03
45.56
43.02
42.38
41.03
35.48
35.16
34.85
30.43
29.84
28.30
26.77
24.23
20.82
20.77
f1 (ppm)

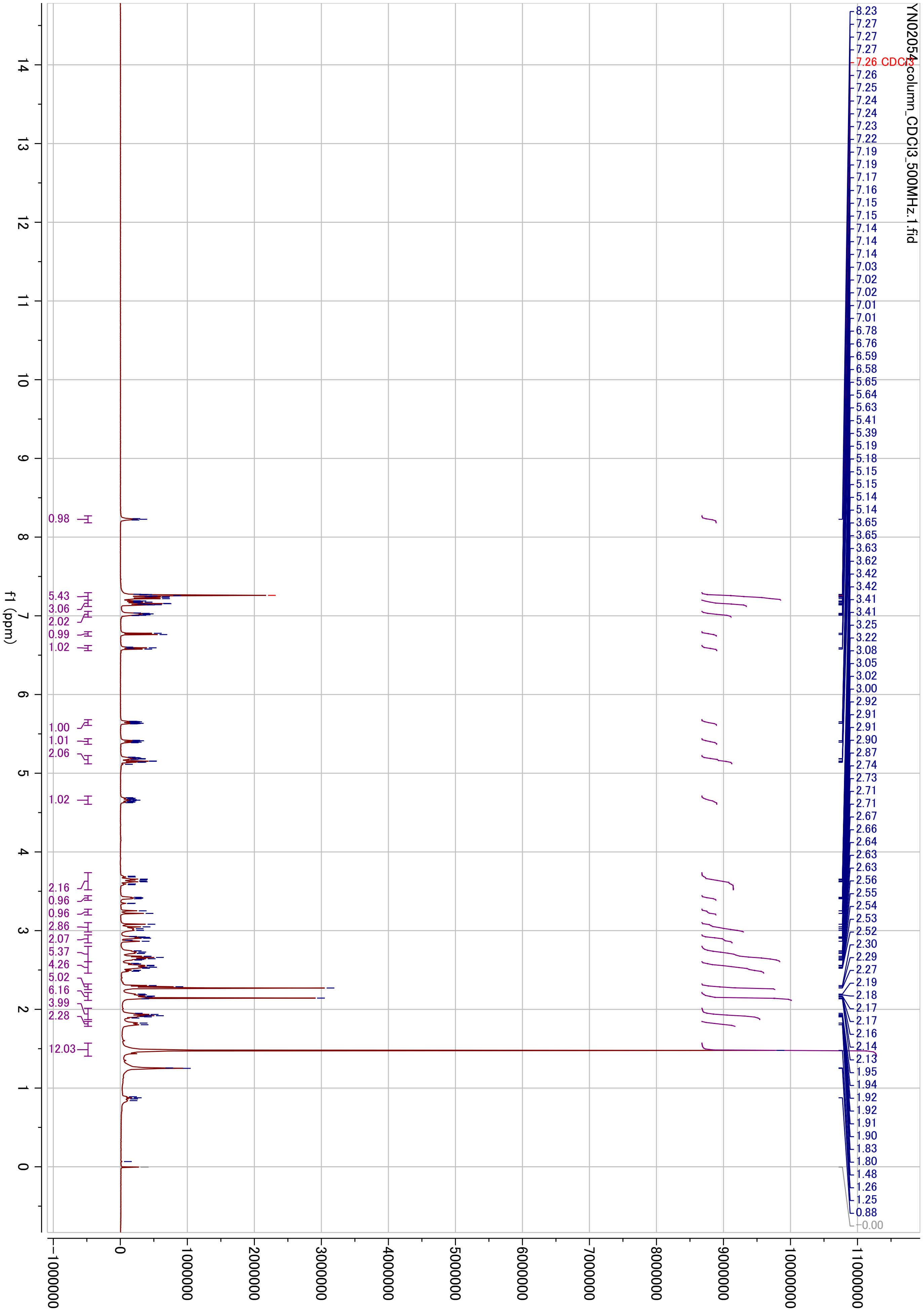
YN02054column_CDCl3_500MHz.1.fid
8.23
7.27
7.27
7.27
7.27
7.26 CDCl3
7.26
7.25
7.24
7.24
7.23
7.22
7.19
7.19
7.17
7.16
7.15
7.15
7.14
7.14
7.14
7.03
7.02
7.02
7.01
7.01
6.78
6.76
6.59
6.58
5.65
5.64
5.63
5.41
5.39
5.19
5.18
5.15
5.15
5.14
5.14
3.65
3.65
3.63
3.62
3.42
3.42
3.41
3.41
3.25
3.22
3.08
3.05
3.02
3.00
2.92
2.91
2.91
2.90
2.87
2.74
2.73
2.71
2.71
2.67
2.66
2.64
2.63
2.63
2.56
2.55
2.54
2.53
2.52
2.30
2.29
2.27
2.19
2.18
2.17
2.17
2.16
2.14
2.13
1.95
1.94
1.92
1.92
1.91
1.90
1.83
1.80
1.48
1.26
1.25
0.88
0.00
0.98
5.43
3.06
2.02
0.99
1.02
1.00
1.01
2.06
1.02
2.16
0.96
0.96
2.86
2.07
5.37
4.26
5.02
6.16
3.99
2.28
12.03
f1 (ppm)
14
13
12
11
10
9
8
7
6
5
4
3
2
1
0
-1000000
0
1000000
2000000
3000000
4000000
5000000
6000000
7000000
8000000
9000000
10000000
11000000

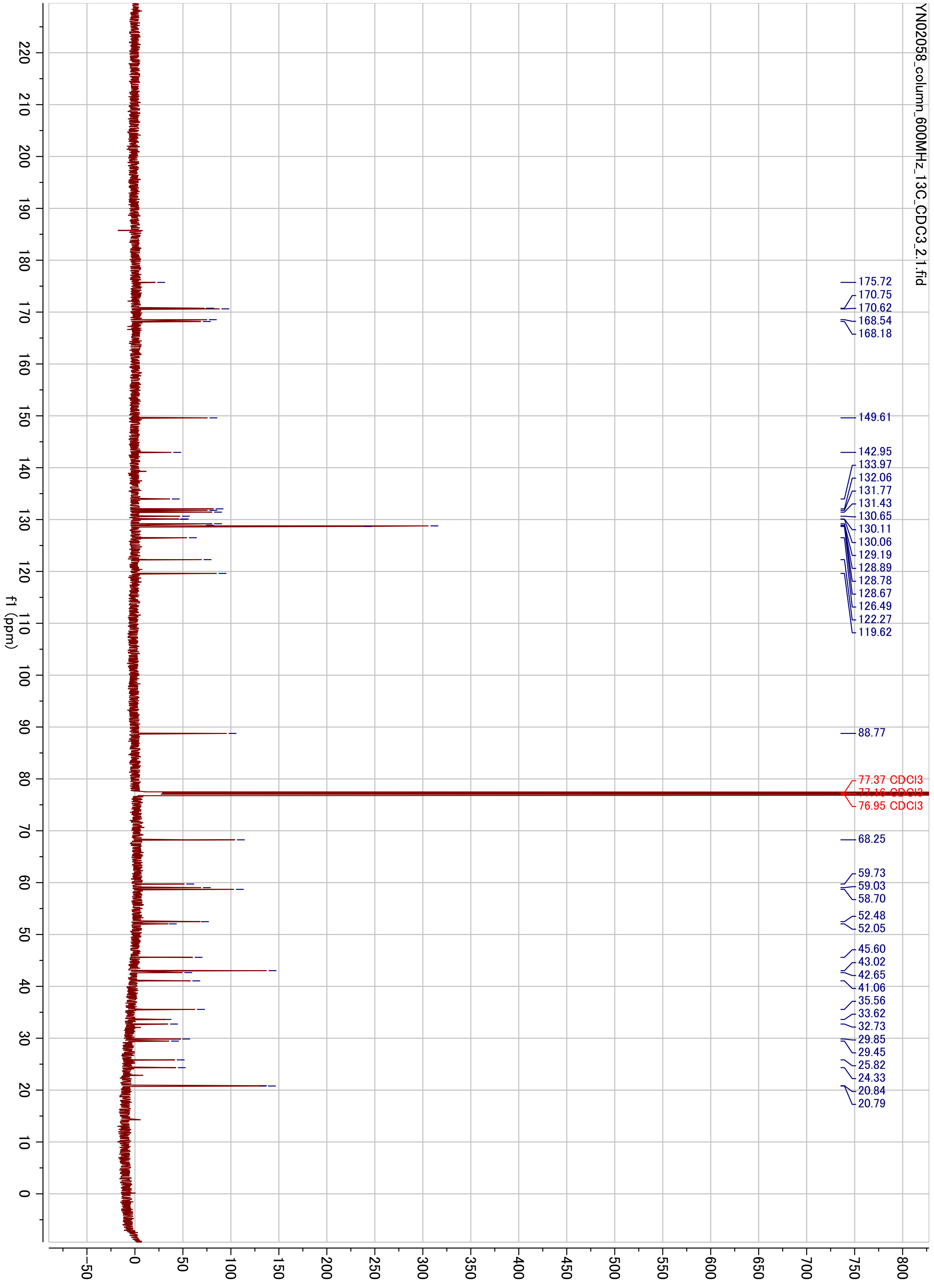
YN02058_column_600MHz_13C_CDC3_2.1.fid
175.72
170.75
170.62
168.54
168.18
149.61
142.95
133.97
132.06
131.77
131.43
130.65
130.11
130.06
129.19
128.89
128.78
128.67
126.49
122.27
119.62
88.77
77.37 CDCl3
77.16 CDCl3
76.95 CDCl3
68.25
59.73
59.03
58.70
52.48
52.05
45.60
43.02
42.65
41.06
35.56
33.62
32.73
29.85
29.45
25.82
24.33
20.84
20.79
f1 (ppm)

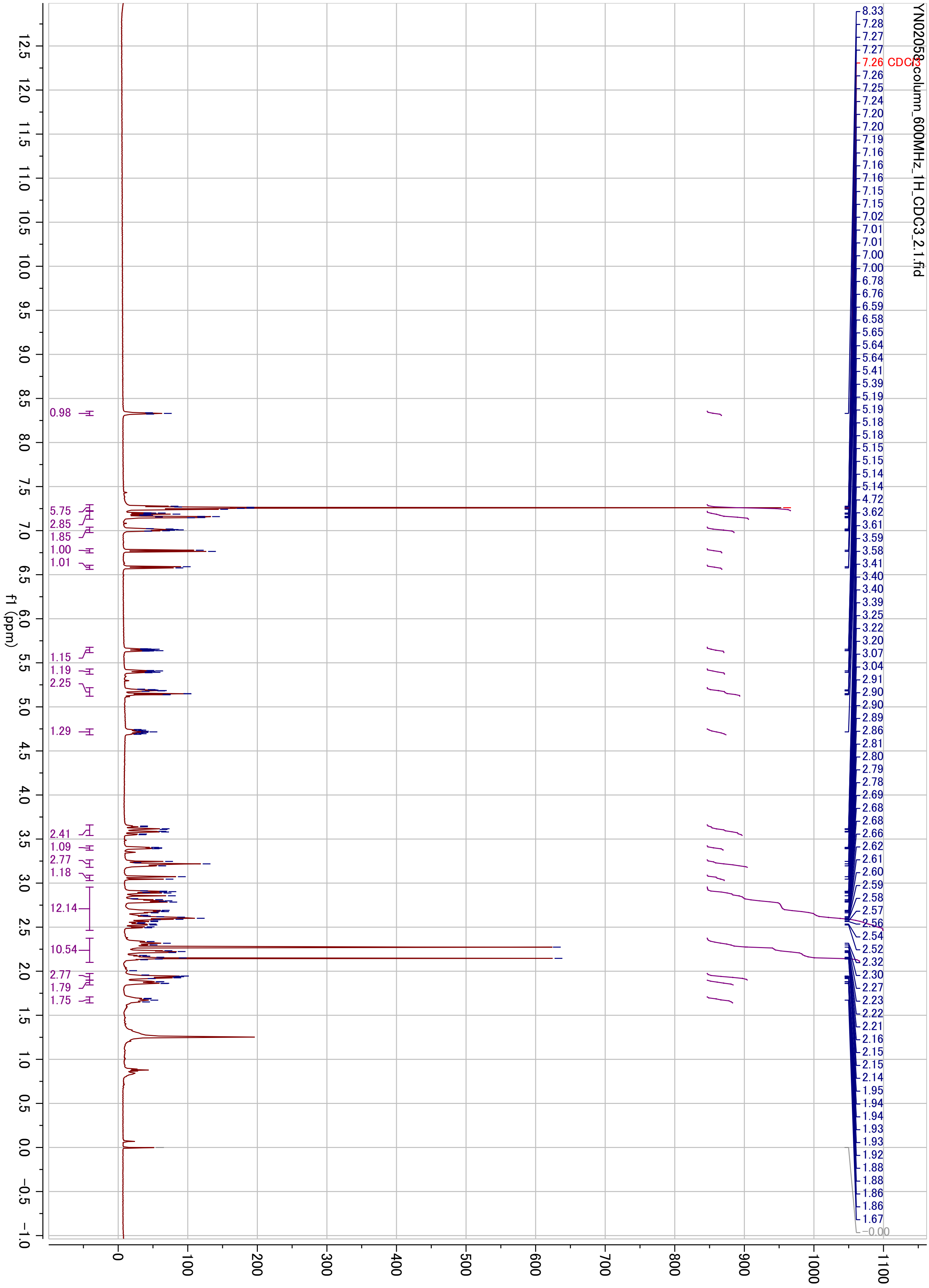
YN02058column_600MHz_1H_CDC3_2.1.fid
f1 (ppm)
7.26 CDCl3

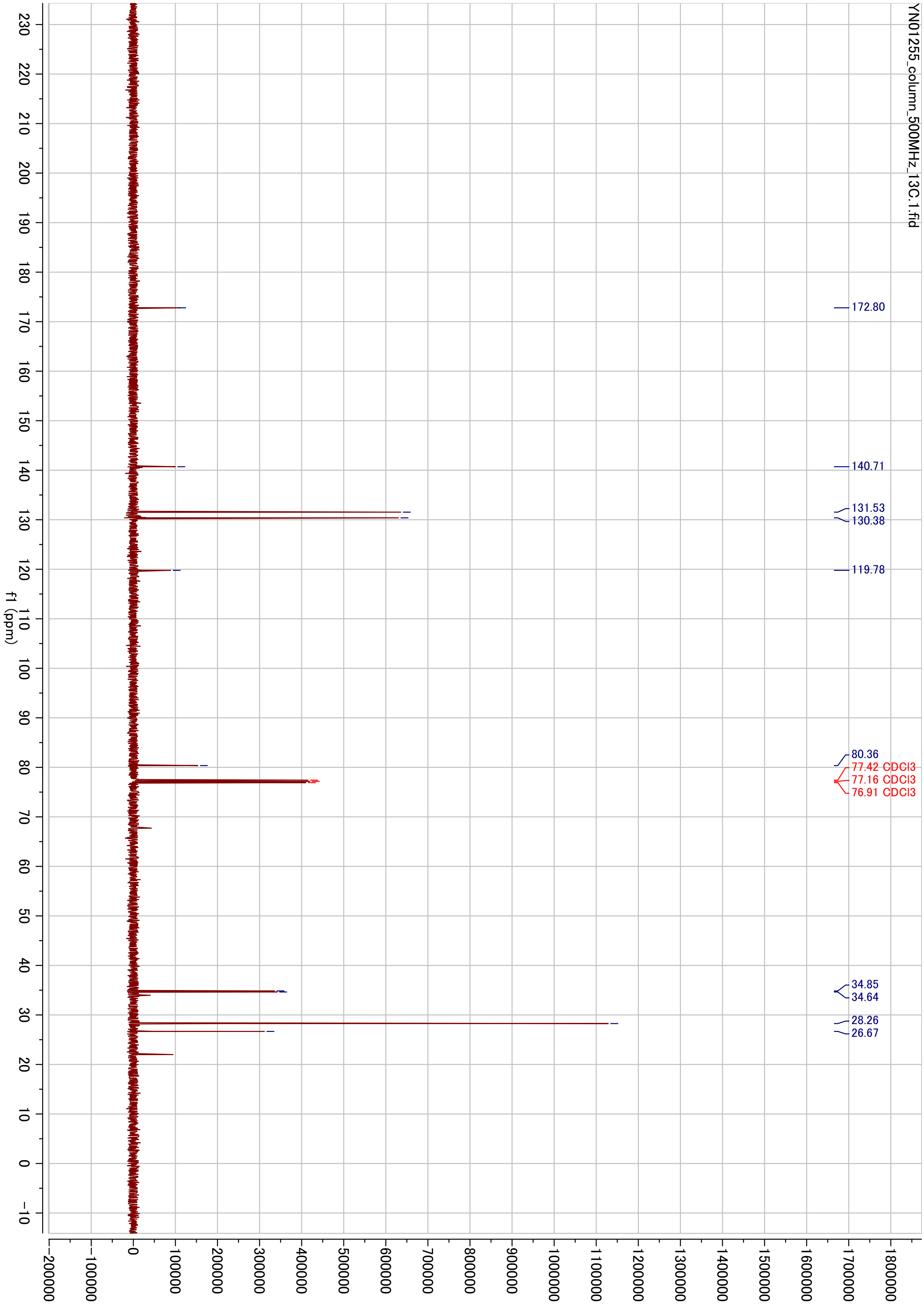
YN01255_column_500MHz_13C.1.fid
172.80
140.71
131.53
130.38
119.78
80.36
77.42 CDCl3
77.16 CDCl3
76.91 CDCl3
34.85
34.64
28.26
26.67
f1 (ppm)

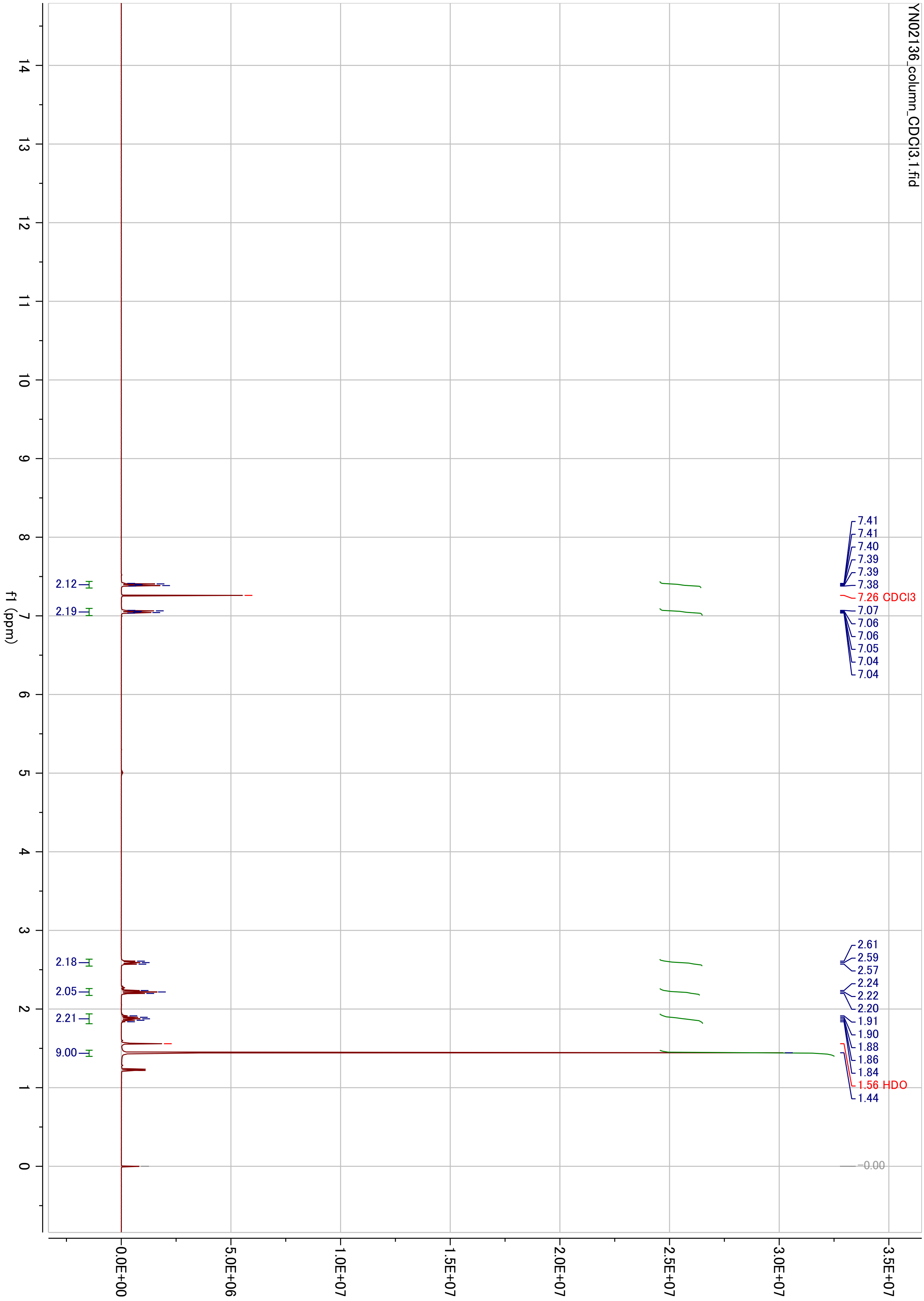
YN02136_column_CDCl3.1.fid
f1 (ppm)
2.12
2.19
2.18
2.05
2.21
9.00
7.41
7.41
7.40
7.39
7.39
7.38
7.26 CDCl3
7.07
7.06
7.06
7.05
7.04
7.04
2.61
2.59
2.57
2.24
2.22
2.20
1.91
1.90
1.88
1.86
1.84
1.56 HDO
1.44
0.00
0.0E+00
5.0E+06
1.0E+07
1.5E+07
2.0E+07
2.5E+07
3.0E+07
3.5E+07

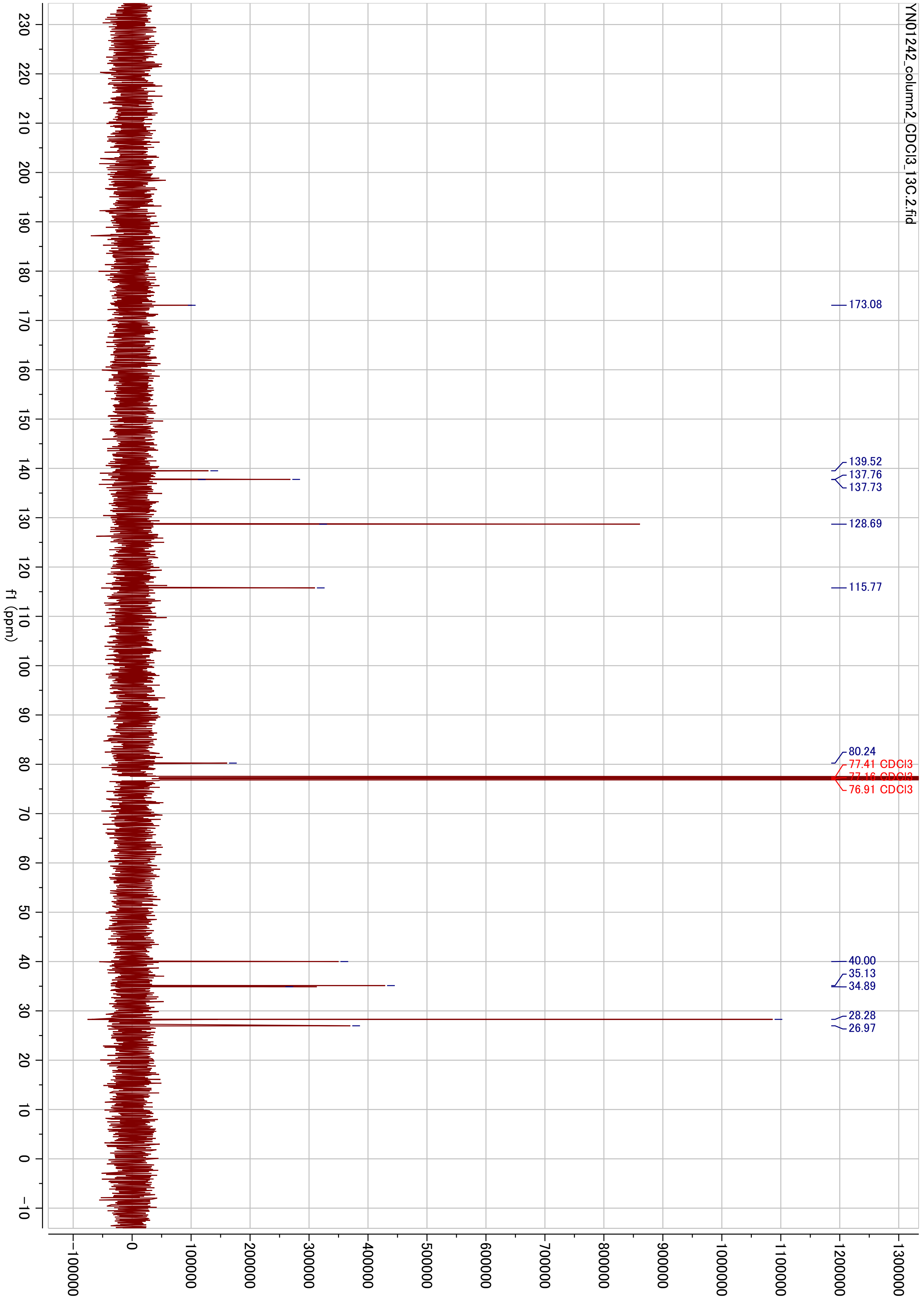
YN01242_column2_CDCl3_13C.2.fid
173.08
139.52
137.76
137.73
128.69
115.77
80.24
77.41 CDCl3
77.16 CDCl3
76.91 CDCl3
40.00
35.13
34.89
28.28
26.97
f1 (ppm)

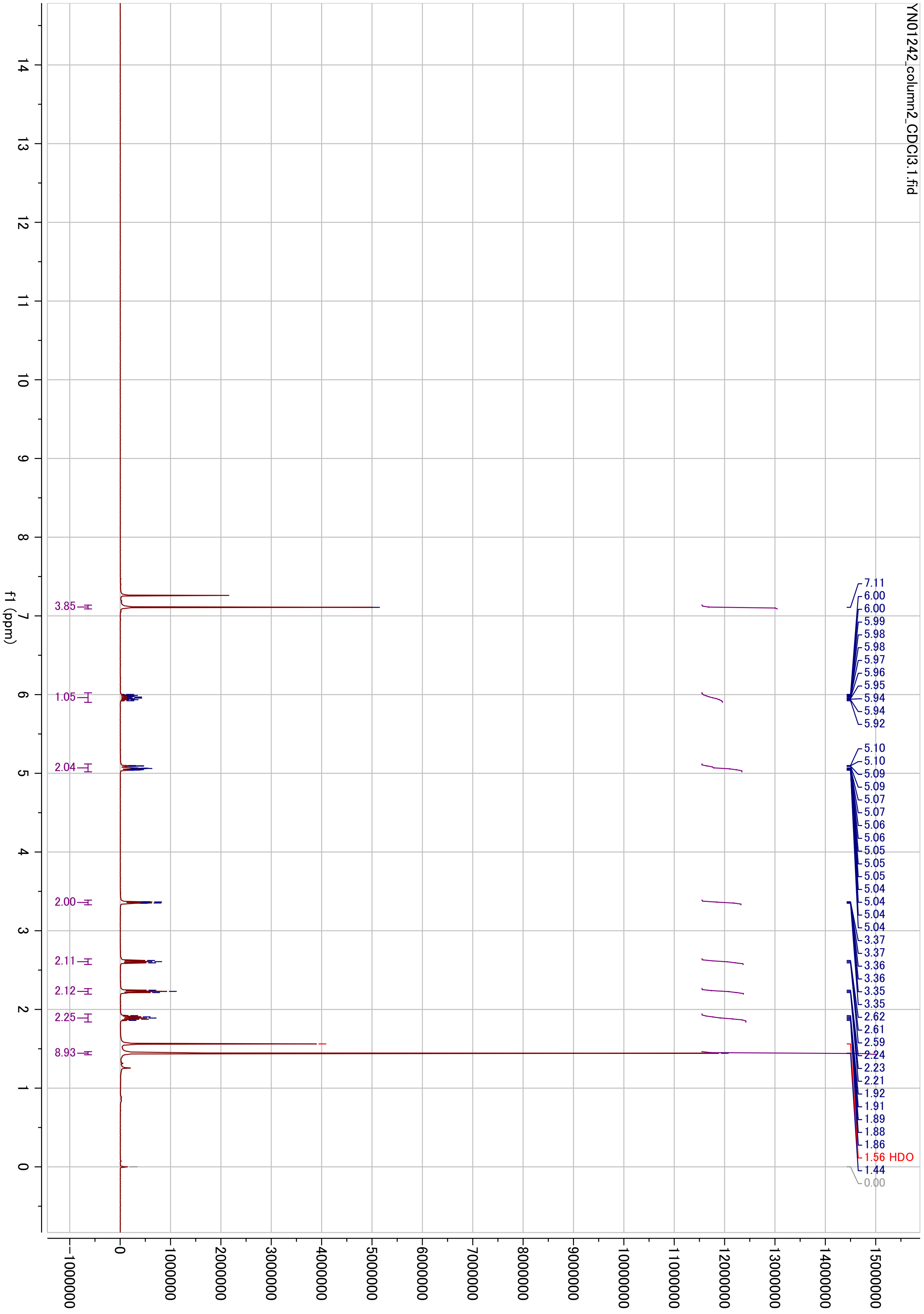

YN01242_column2_CDCl3.1.fid
7.11
6.00
6.00
5.99
5.98
5.98
5.97
5.96
5.95
5.94
5.94
5.92
5.10
5.10
5.09
5.09
5.07
5.07
5.06
5.06
5.05
5.05
5.05
5.04
5.04
5.04
5.04
3.37
3.37
3.36
3.36
3.35
3.35
2.62
2.61
2.59
2.24
2.23
2.21
1.92
1.91
1.89
1.88
1.86
1.56 HDO
1.44
0.00
3.85
1.05
2.04
2.00
2.11
2.12
2.25
8.93
f1 (ppm)

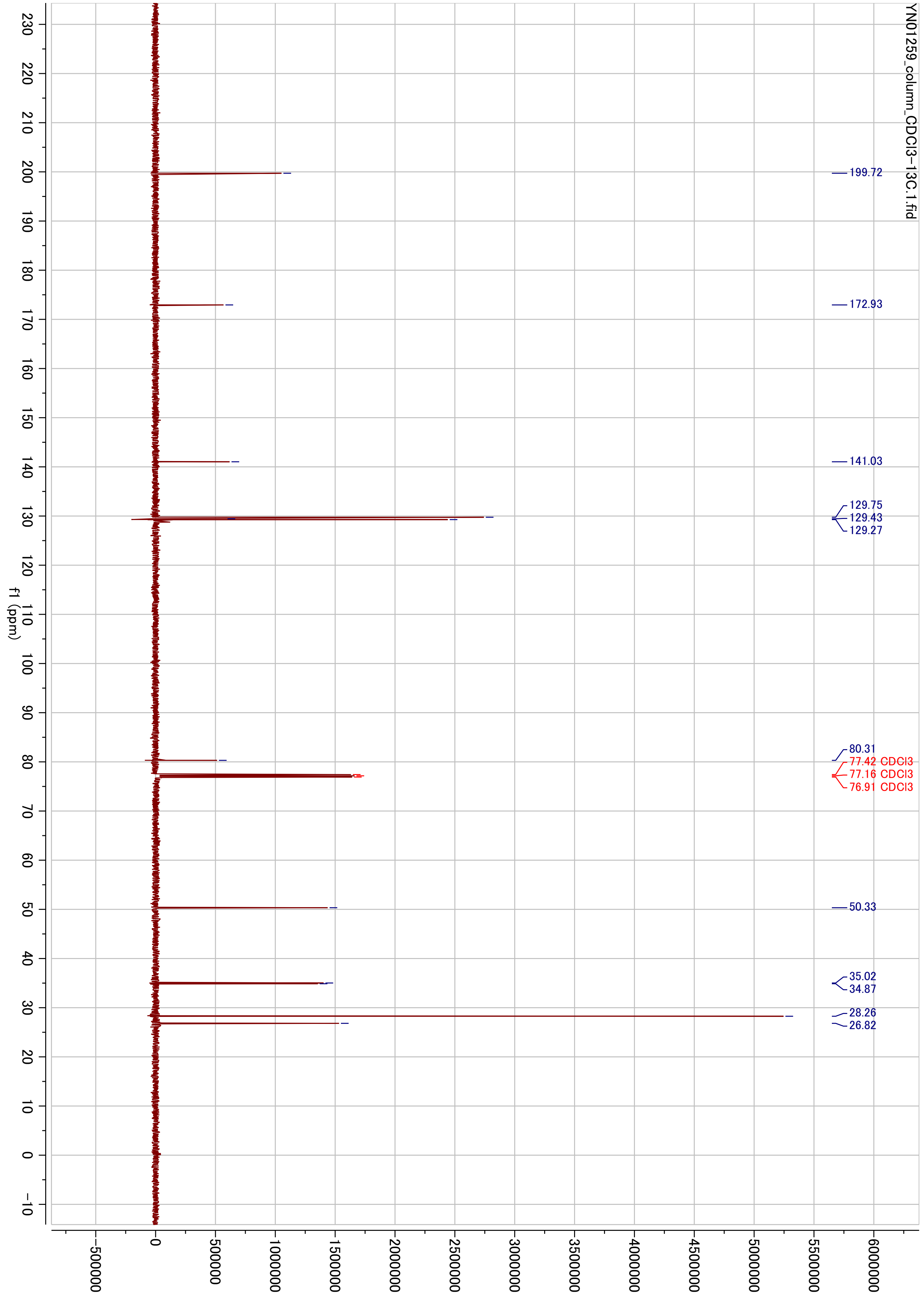
YN01259_column_CDCl3-13C.1.fid
199.72
172.93
141.03
129.75
129.43
129.27
80.31
77.42 CDCl3
77.16 CDCl3
76.91 CDCl3
50.33
35.02
34.87
28.26
26.82
f1 (ppm)

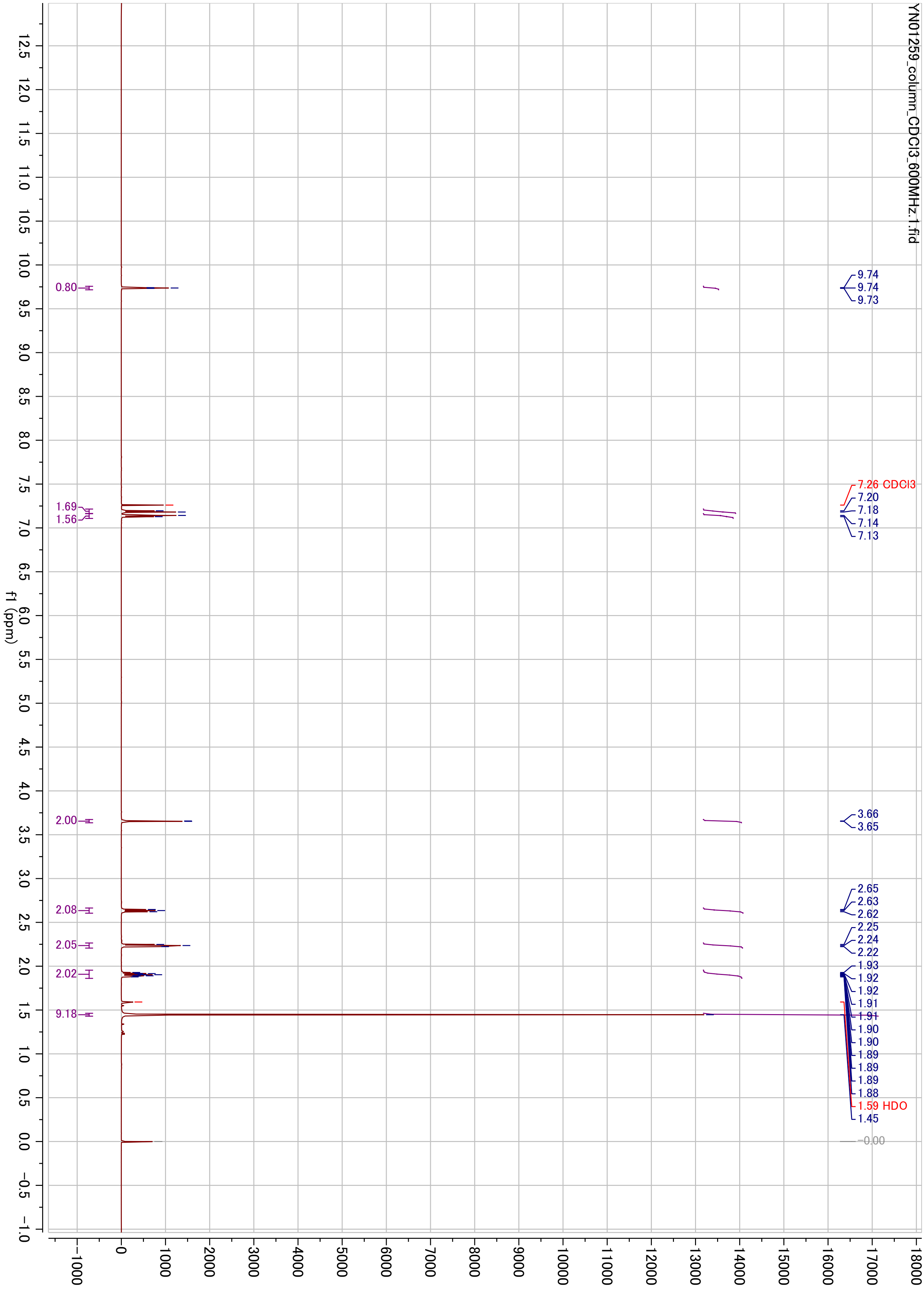

YN01259_column_CDCl3_600MHz.1.fid
9.74
9.74
9.73
7.26 CDCl3
7.20
7.18
7.14
7.13
3.66
3.65
2.65
2.63
2.62
2.25
2.24
2.22
1.93
1.92
1.92
1.91
1.91
1.90
1.90
1.89
1.89
1.89
1.88
1.59 HDO
1.45
-0.00
0.80
1.69
1.56
2.00
2.08
2.05
2.02
9.18
f1 (ppm)

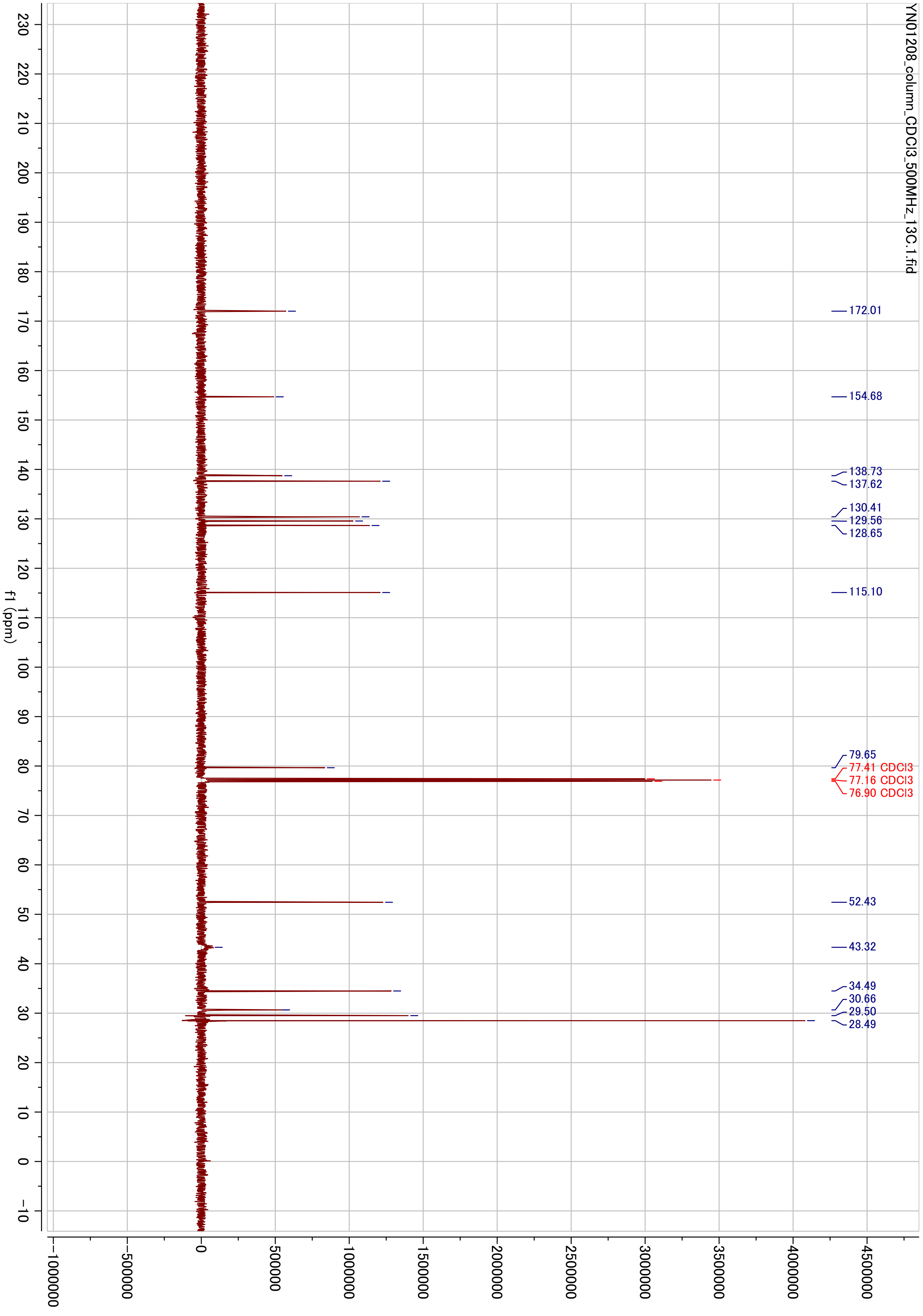
YN01208_column_CDCl3_500MHz_13C.1.fid
172.01
154.68
138.73
137.62
130.41
129.56
128.65
115.10
79.65
77.41 CDCl3
77.16 CDCl3
76.90 CDCl3
52.43
43.32
34.49
30.66
29.50
28.49
f1 (ppm)

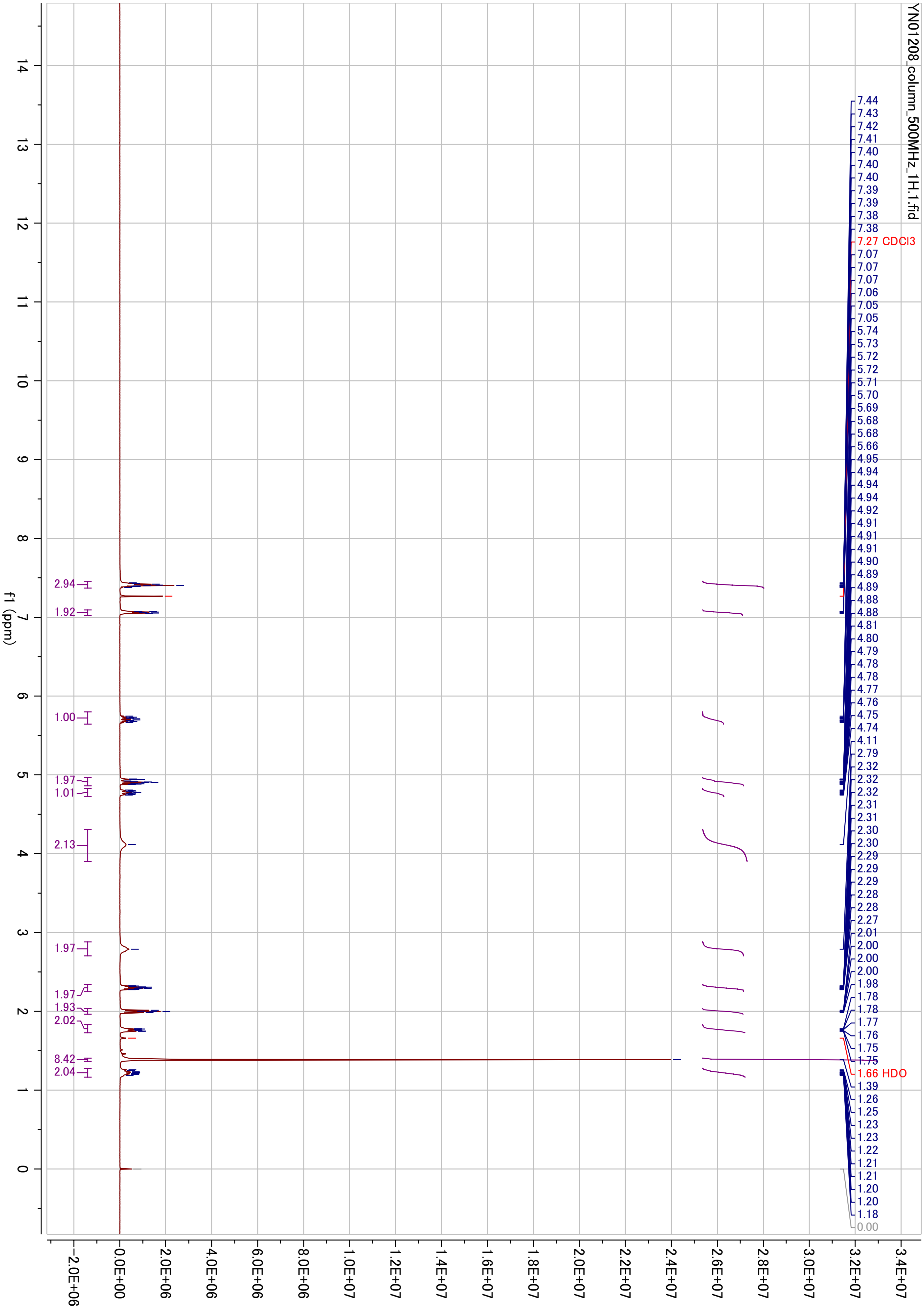

YN01208_column_500MHz_1H.1.fid
7.44
7.43
7.42
7.41
7.40
7.40
7.40
7.39
7.39
7.38
7.38
7.27 CDCl3
7.07
7.07
7.07
7.06
7.05
7.05
5.74
5.73
5.72
5.72
5.71
5.70
5.69
5.68
5.68
5.66
4.95
4.94
4.94
4.94
4.92
4.91
4.91
4.91
4.90
4.89
4.89
4.88
4.88
4.81
4.80
4.79
4.78
4.78
4.77
4.76
4.75
4.74
4.11
2.79
2.32
2.32
2.32
2.31
2.31
2.30
2.30
2.29
2.29
2.29
2.28
2.28
2.27
2.01
2.00
2.00
2.00
1.98
1.78
1.78
1.77
1.76
1.75
1.75
1.66 HDO
1.39
1.26
1.25
1.23
1.23
1.22
1.21
1.21
1.20
1.20
1.18
0.00
2.94
1.92
1.00
1.97
1.01
2.13
1.97
1.97
1.93
2.02
8.42
2.04
f1 (ppm)

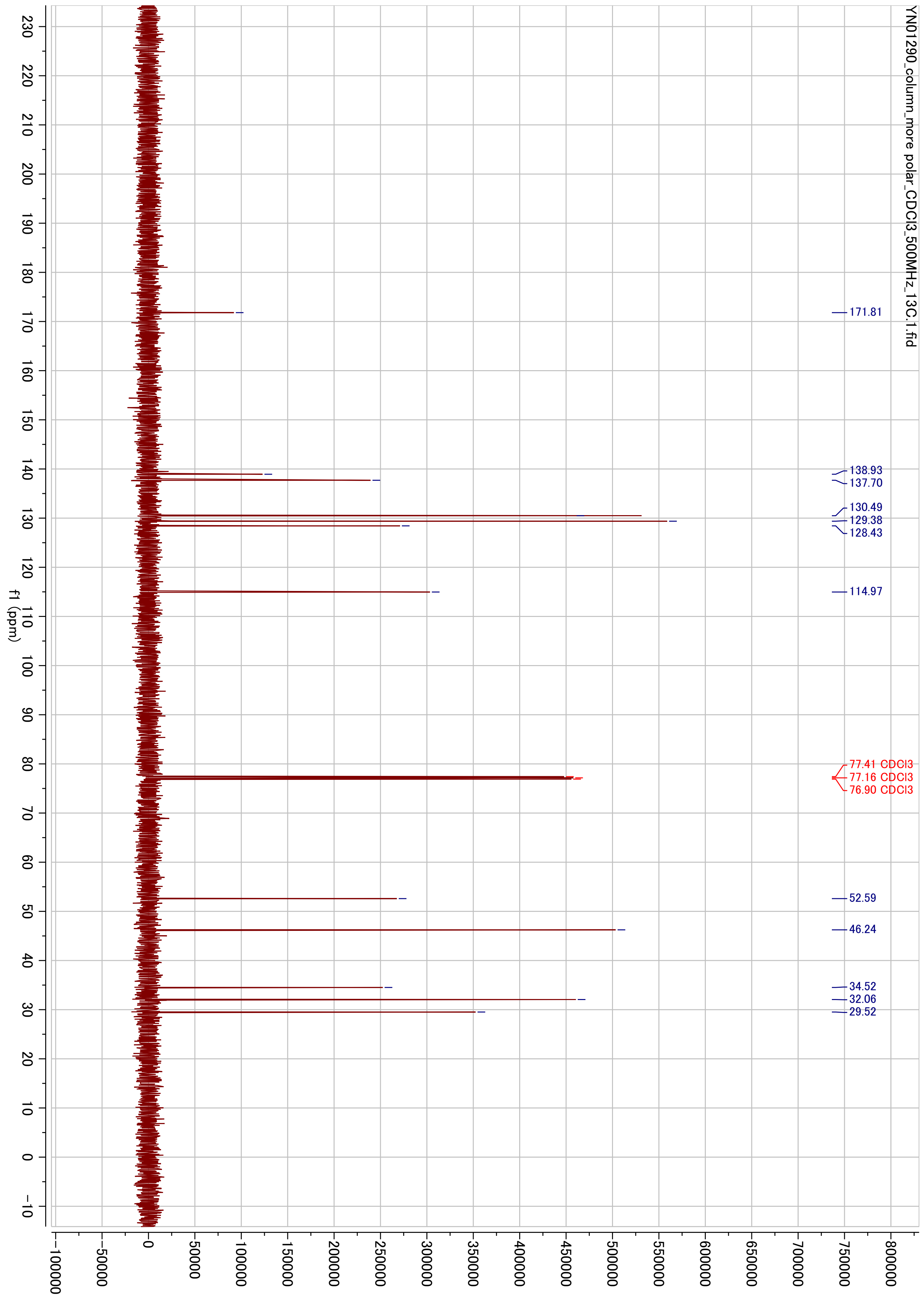
YN01290_column_more polar_CDCl3_500MHz_13C.1.fid
171.81
138.93
137.70
130.49
129.38
128.43
114.97
77.41 CDCl3
77.16 CDCl3
76.90 CDCl3
52.59
46.24
34.52
32.06
29.52
f1 (ppm)
230
220
210
200
190
180
170
160
150
140
130
120
110
100
90
80
70
60
50
40
30
20
10
0
−10
-100000
-50000
0
50000
100000
150000
200000
250000
300000
350000
400000
450000
500000
550000
600000
650000
700000
750000
800000

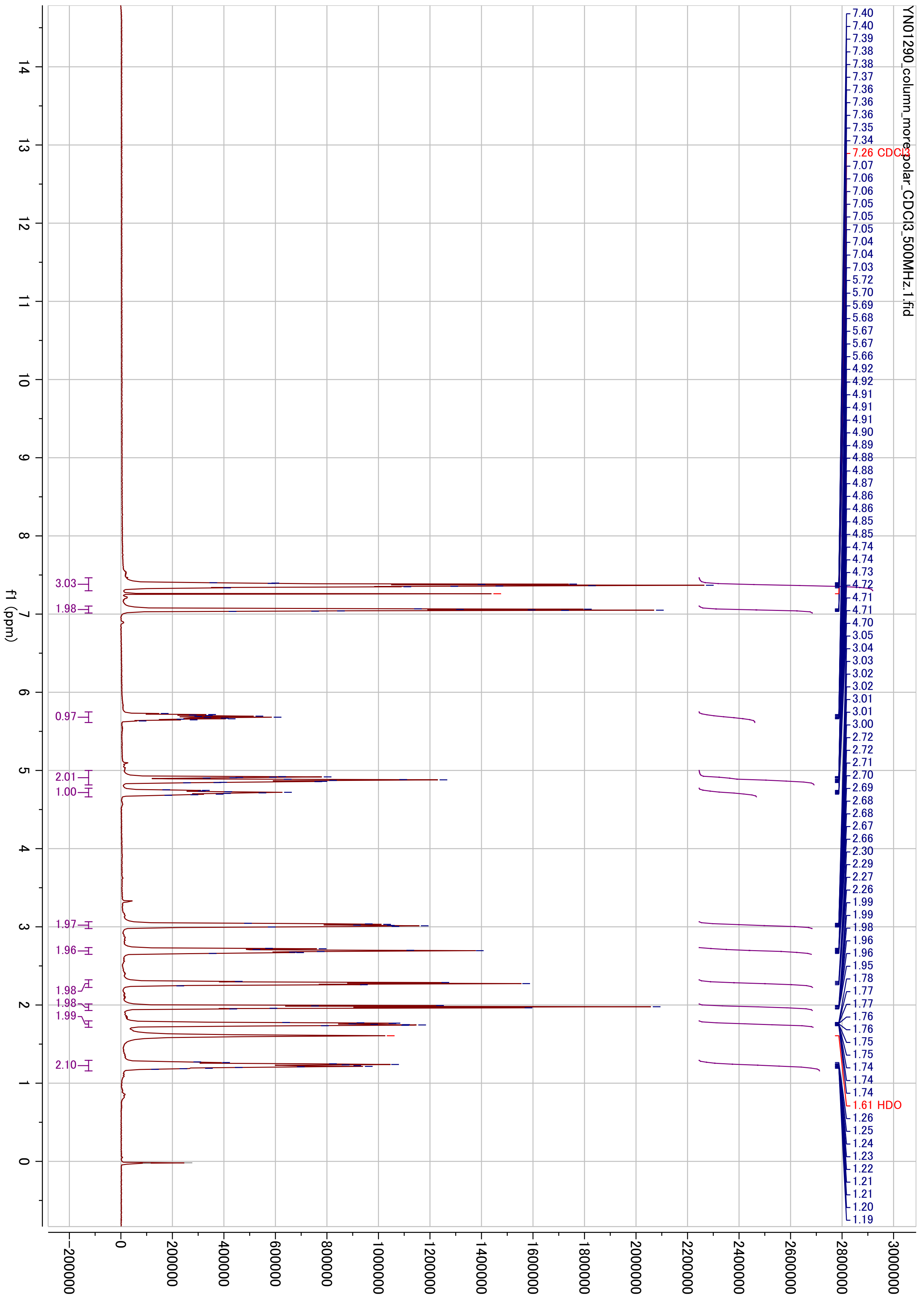
YN01290_column_morepolar_CDCl3_500MHz.1.fid
7.40 7.40 7.39 7.38 7.38 7.37 7.36 7.36 7.36 7.35 7.34
7.26 CDCl3
7.07 7.06 7.06 7.05 7.05 7.05 7.04 7.04 7.03
5.72 5.70 5.69 5.68 5.67 5.67 5.66
4.92 4.92 4.91 4.91 4.91 4.90 4.89 4.88 4.88 4.87 4.86 4.86 4.85 4.85
4.74 4.74 4.73 4.72 4.71 4.71 4.70
3.05 3.04 3.03 3.02 3.02 3.01 3.01 3.00
2.72 2.72 2.71 2.70 2.69 2.68 2.68 2.67 2.66
2.30 2.29 2.27 2.26
1.99 1.99 1.98 1.96 1.96 1.95
1.78 1.77 1.77 1.76 1.76 1.75 1.75 1.74 1.74 1.74
1.61 HDO
1.26 1.25 1.24 1.23 1.22 1.21 1.21 1.20 1.19
3.03
1.98
0.97
2.01
1.00
1.97
1.96
1.98
1.98
1.99
2.10
f1 (ppm)

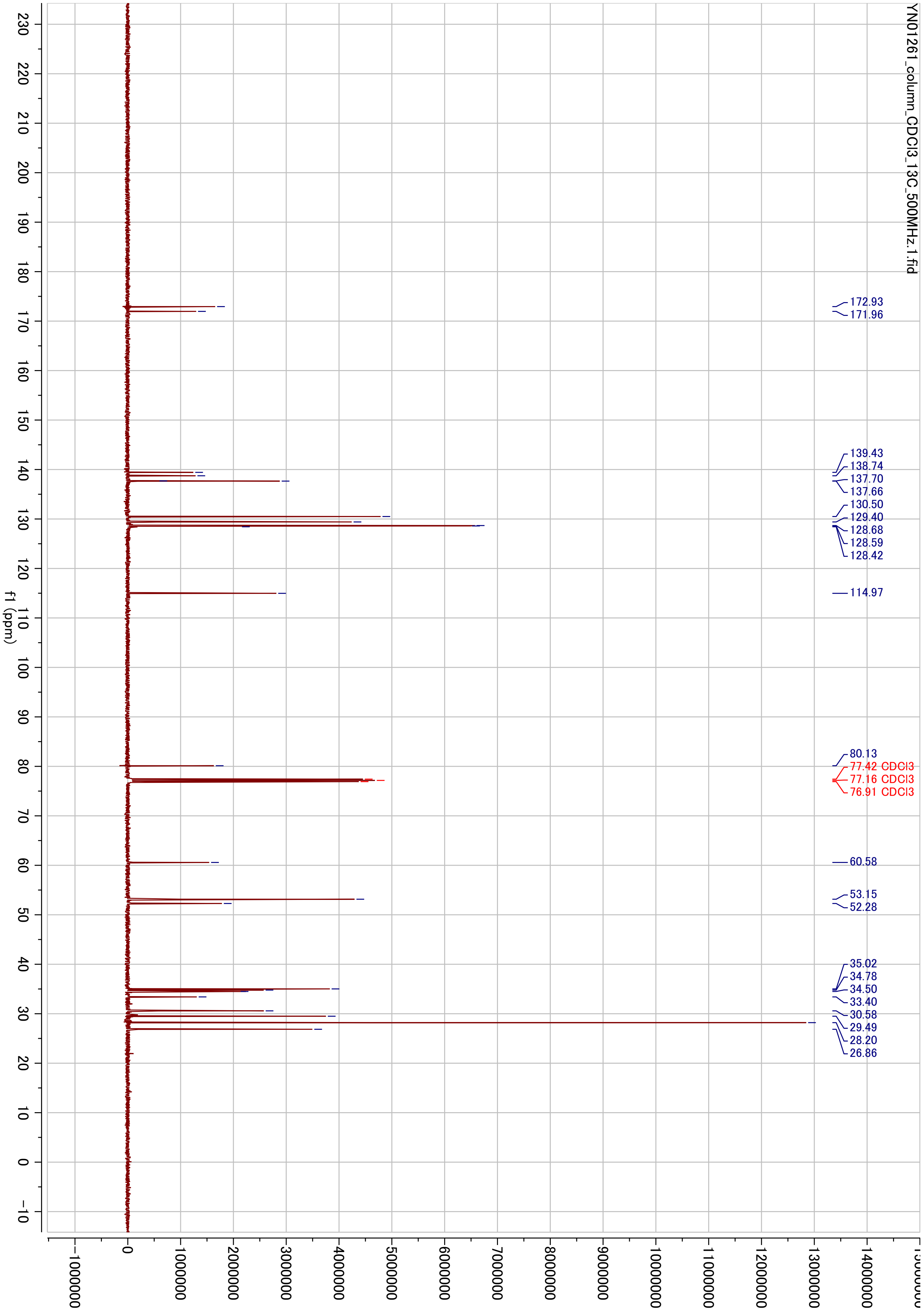

YN01261_column_CDCl3_13C_500MHz.1.fid
172.93
171.96
139.43
138.74
137.70
137.66
130.50
129.40
128.68
128.59
128.42
114.97
80.13
77.42 CDCl3
77.16 CDCl3
76.91 CDCl3
60.58
53.15
52.28
35.02
34.78
34.50
33.40
30.58
29.49
28.20
26.86
f1 (ppm)

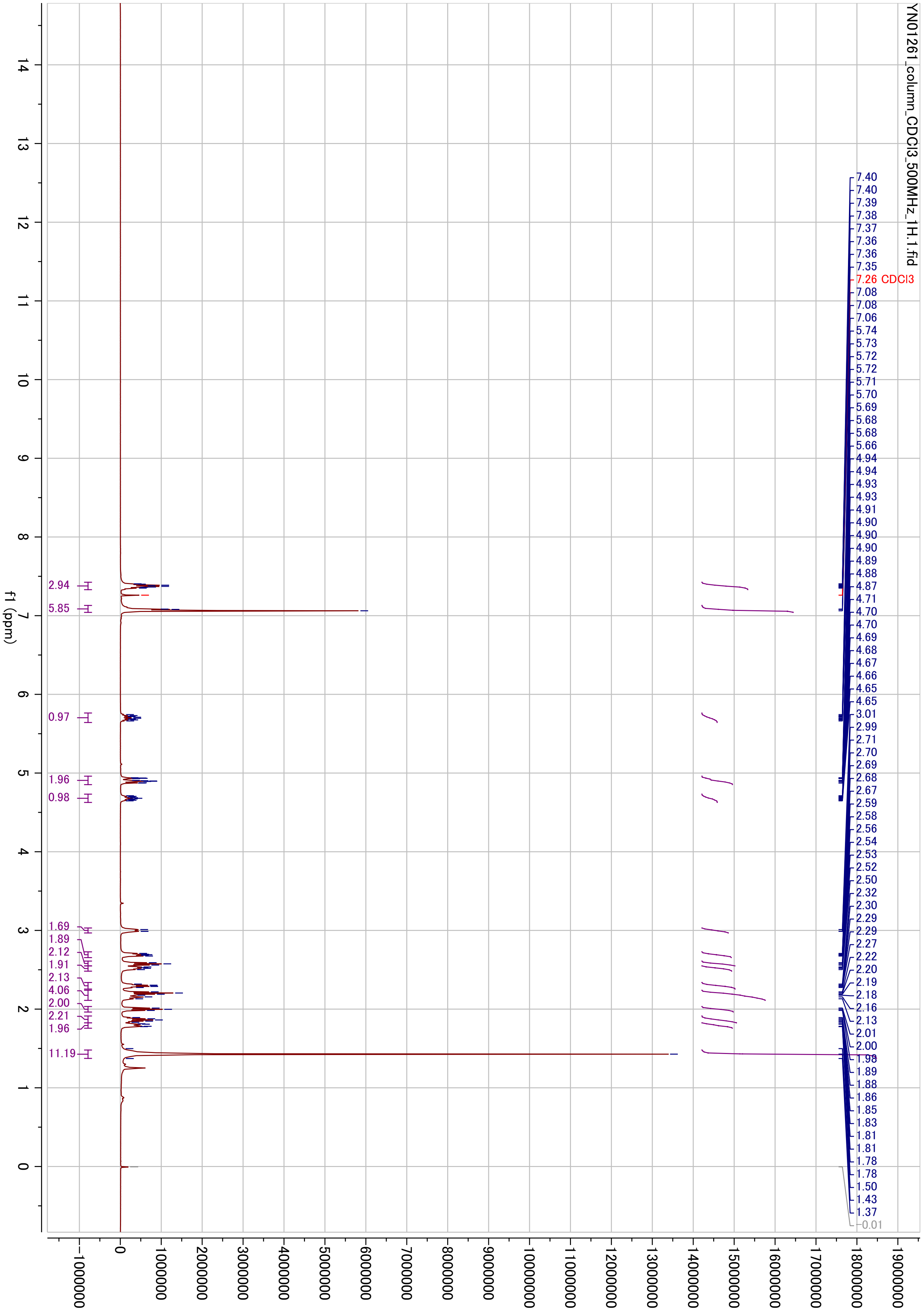

YN01261_column_CDCl3_500MHz_1H.1.fid
7.40
7.40
7.39
7.38
7.37
7.36
7.36
7.35
7.26 CDCl3
7.08
7.08
7.06
5.74
5.73
5.72
5.72
5.71
5.70
5.69
5.68
5.68
5.66
4.94
4.94
4.93
4.93
4.91
4.90
4.90
4.90
4.89
4.88
4.87
4.71
4.70
4.70
4.69
4.68
4.67
4.66
4.65
4.65
3.01
2.99
2.71
2.70
2.69
2.68
2.67
2.59
2.58
2.56
2.54
2.53
2.52
2.50
2.32
2.30
2.29
2.29
2.27
2.22
2.20
2.19
2.18
2.16
2.13
2.01
2.00
1.98
1.89
1.88
1.86
1.85
1.83
1.81
1.81
1.78
1.78
1.50
1.43
1.37
-0.01
2.94
5.85
0.97
1.96
0.98
1.69
1.89
2.12
1.91
2.13
4.06
2.00
2.21
1.96
11.19
f1 (ppm)

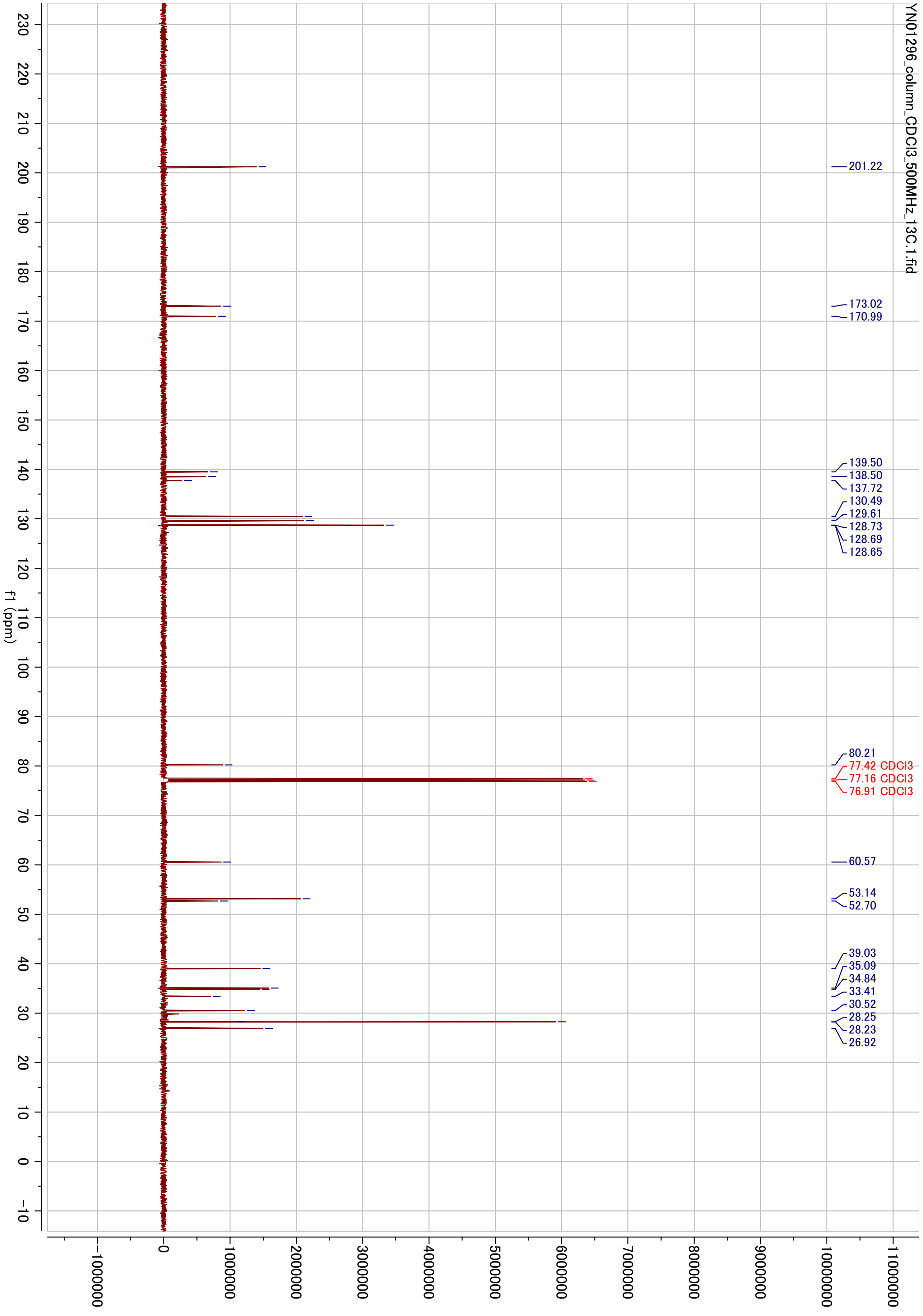
YN01296_column_CDCl3_500MHz_13C.1.fid
201.22
173.02
170.99
139.50
138.50
137.72
130.49
129.61
128.73
128.69
128.65
80.21
77.42 CDCl3
77.16 CDCl3
76.91 CDCl3
60.57
53.14
52.70
39.03
35.09
34.84
33.41
30.52
28.25
28.23
26.92
f1 (ppm)

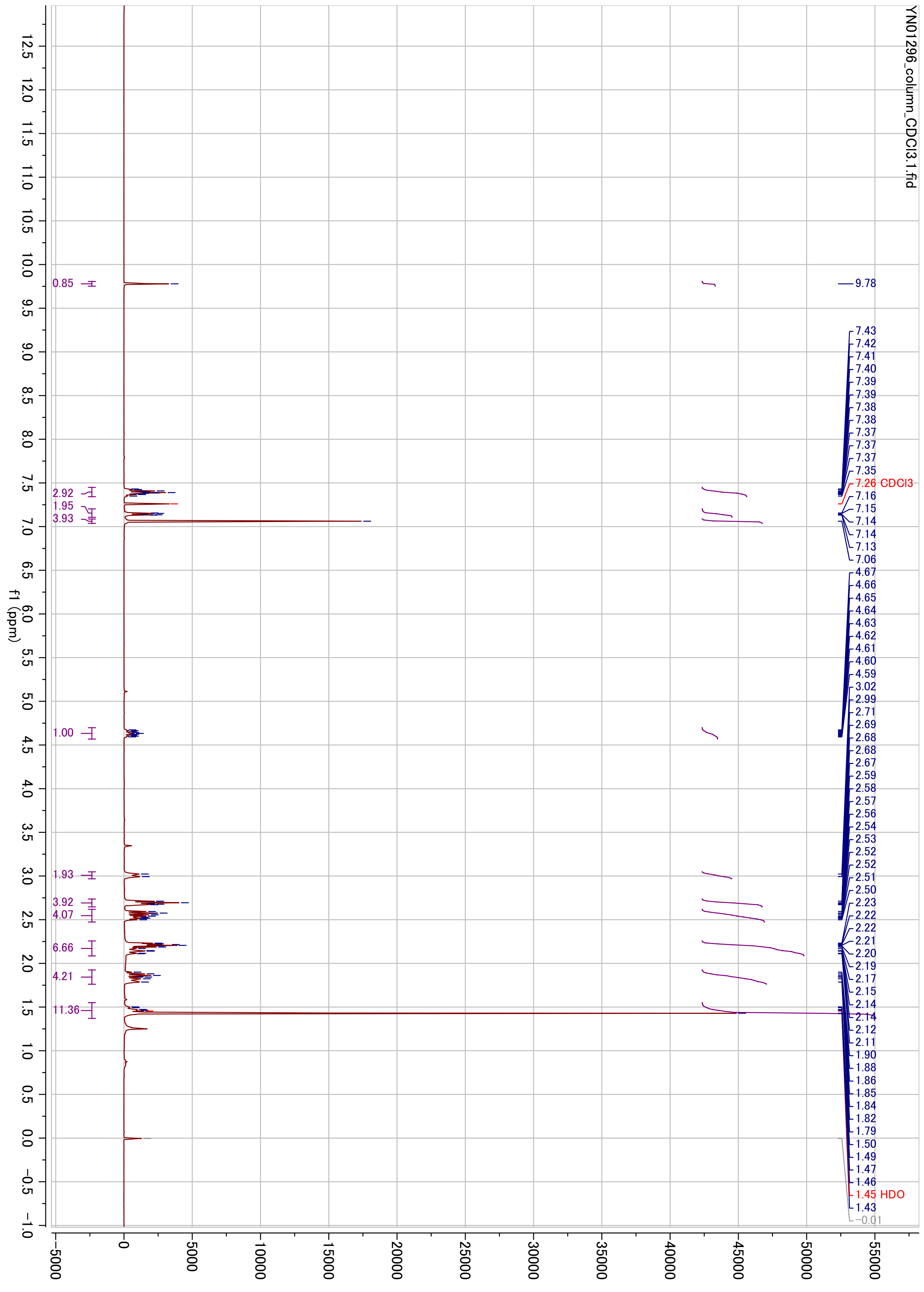
YN01296_column_CDCl3.1.fid
f1 (ppm)
7.26 CDCl3
1.45 HDO

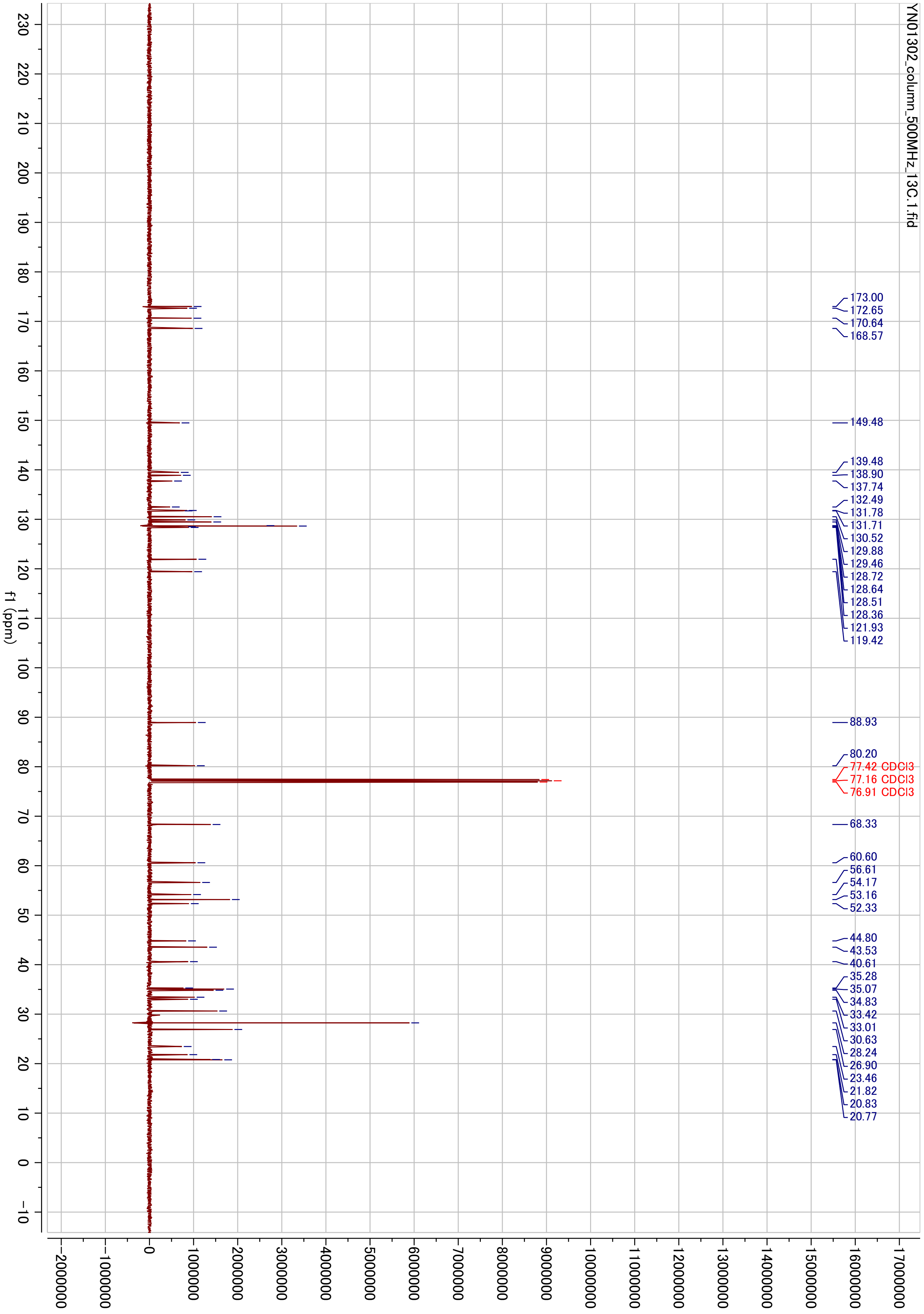
YN01302_column_500MHz_13C.1.fid
173.00
172.65
170.64
168.57
149.48
139.48
138.90
137.74
132.49
131.78
131.71
130.52
129.88
129.46
128.72
128.64
128.51
128.36
121.93
119.42
88.93
80.20
77.42 CDCl3
77.16 CDCl3
76.91 CDCl3
68.33
60.60
56.61
54.17
53.16
52.33
44.80
43.53
40.61
35.28
35.07
34.83
33.42
33.01
30.63
28.24
26.90
23.46
21.82
20.83
20.77
f1 (ppm)

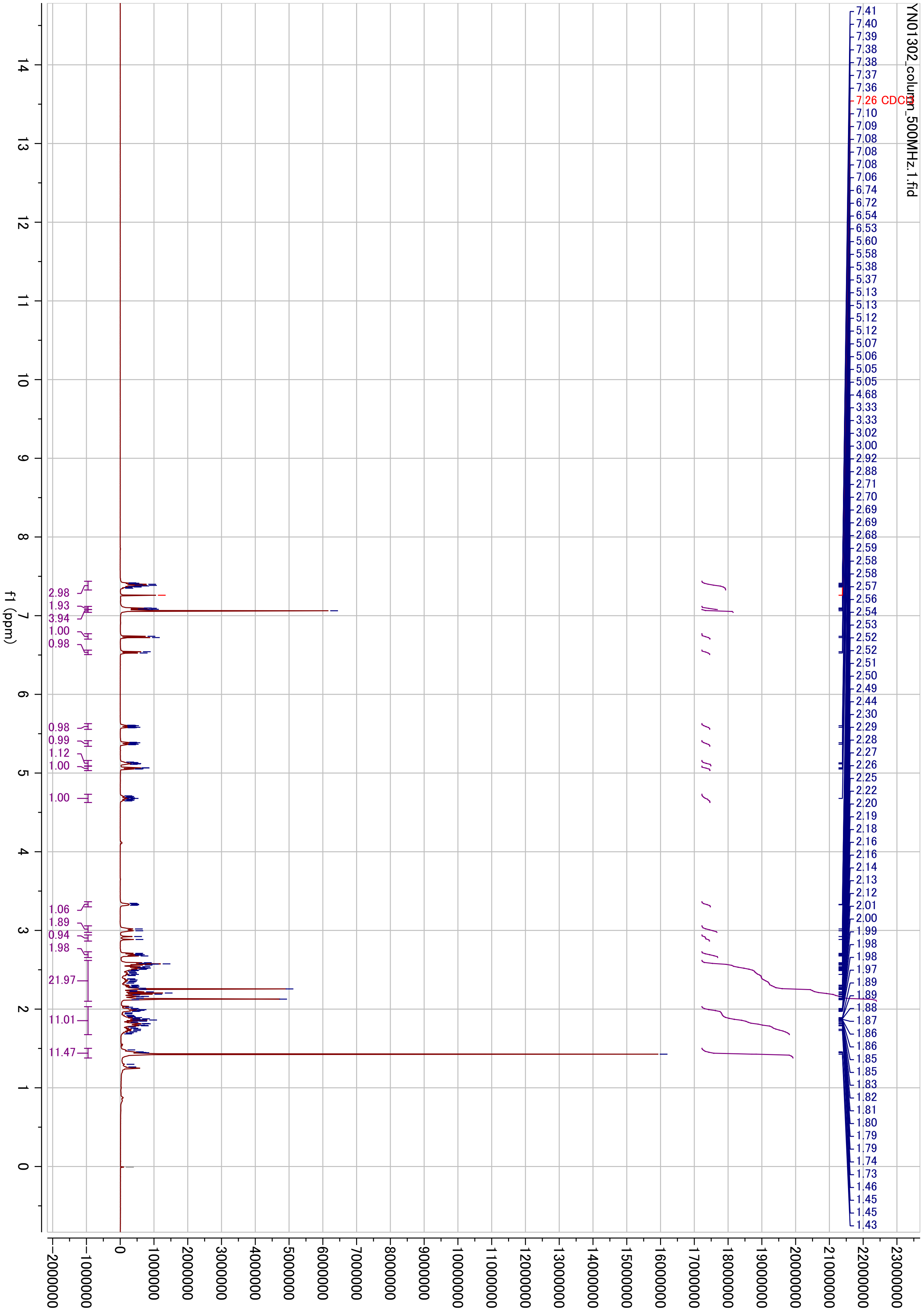
YN01302_column_500MHz.1.fid
7.41
7.40
7.39
7.38
7.38
7.37
7.36
7.26 CDCl3
7.10
7.09
7.08
7.08
7.08
7.06
6.74
6.72
6.54
6.53
5.60
5.58
5.38
5.37
5.13
5.13
5.12
5.12
5.07
5.06
5.05
5.05
4.68
3.33
3.33
3.02
3.00
2.92
2.88
2.71
2.70
2.69
2.69
2.68
2.59
2.58
2.58
2.57
2.56
2.54
2.53
2.52
2.52
2.51
2.50
2.49
2.44
2.30
2.29
2.28
2.27
2.26
2.25
2.22
2.20
2.19
2.18
2.16
2.16
2.14
2.13
2.12
2.01
2.00
1.99
1.98
1.98
1.97
1.89
1.89
1.88
1.87
1.86
1.86
1.85
1.85
1.83
1.82
1.81
1.80
1.79
1.79
1.74
1.73
1.46
1.45
1.45
1.43
2.98
1.93
3.94
1.00
0.98
0.98
0.99
1.12
1.00
1.00
1.06
1.89
0.94
1.98
21.97
11.01
11.47
f1 (ppm)

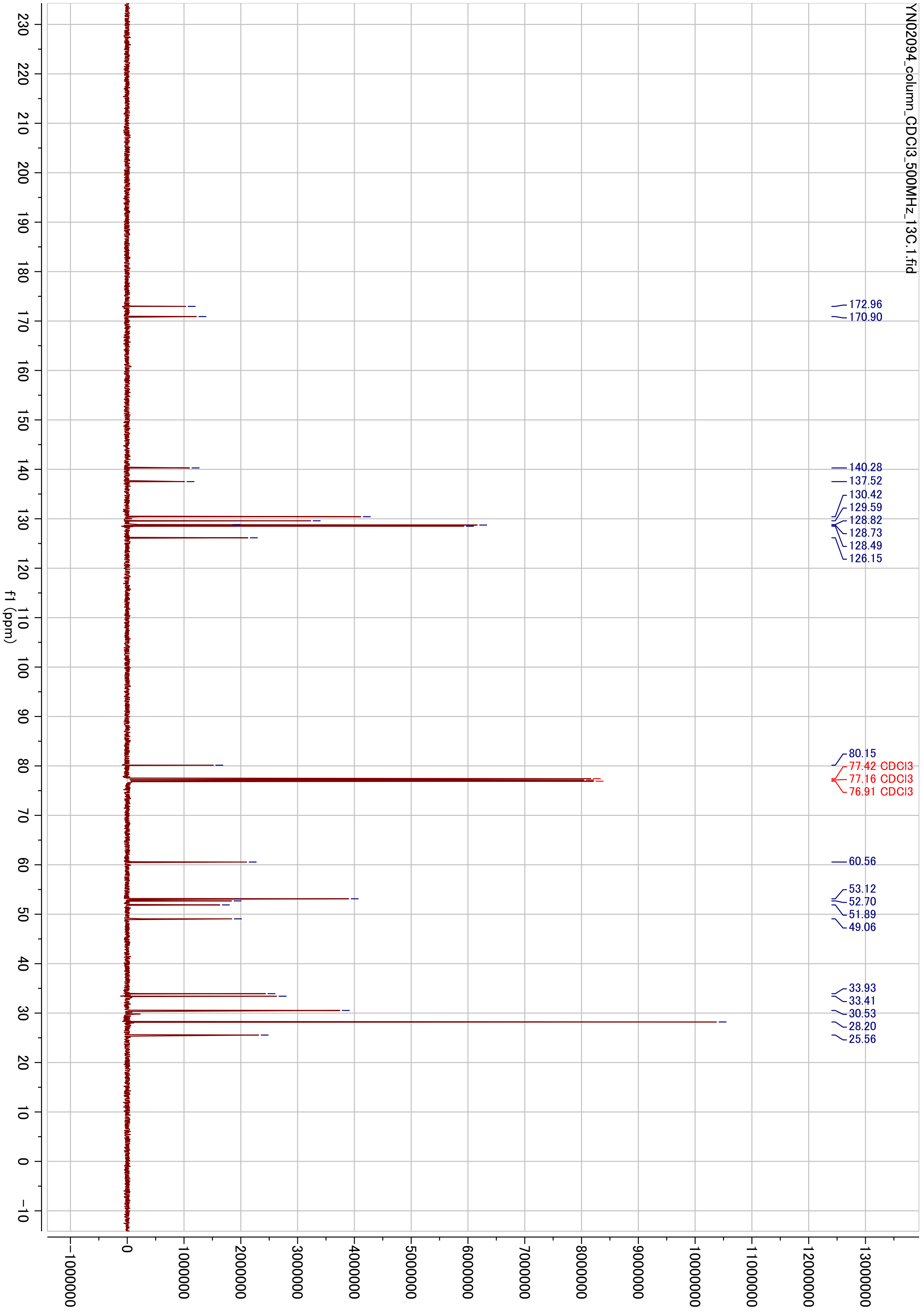

YN02094_column_CDCl3_500MHz_13C.1.fid
172.96
170.90
140.28
137.52
130.42
129.59
128.82
128.73
128.49
126.15
80.15
77.42 CDCl3
77.16 CDCl3
76.91 CDCl3
60.56
53.12
52.70
51.89
49.06
33.93
33.41
30.53
28.20
25.56
f1 (ppm)

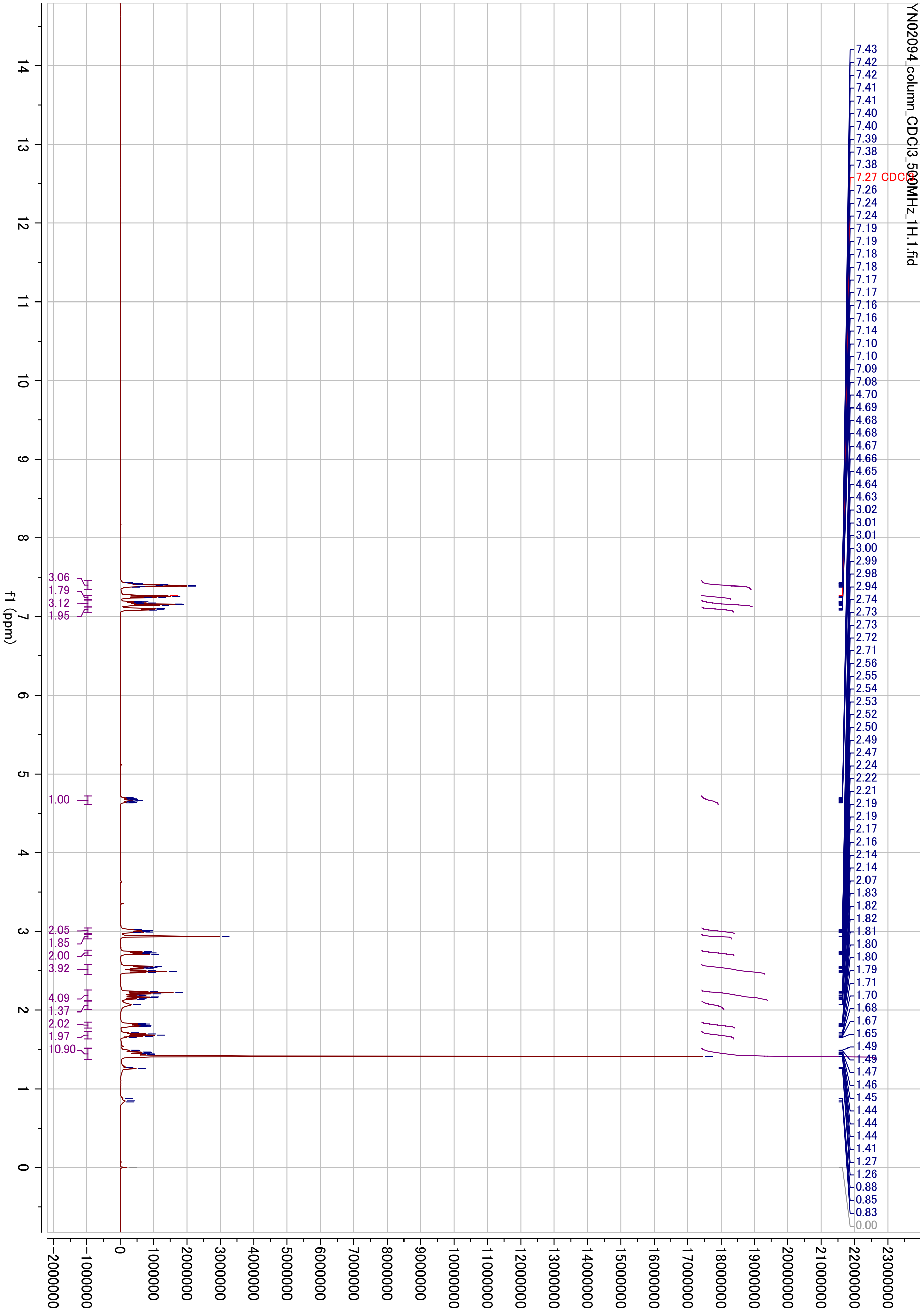
YN02094_column_CDCl3_500MHz_1H.1.fid
7.43
7.42
7.42
7.41
7.41
7.40
7.40
7.39
7.38
7.38
7.27 CDCl3
7.26
7.24
7.24
7.19
7.19
7.18
7.18
7.17
7.17
7.16
7.16
7.14
7.10
7.10
7.09
7.08
4.70
4.69
4.68
4.68
4.67
4.66
4.65
4.64
4.63
3.02
3.01
3.01
3.00
2.99
2.98
2.94
2.74
2.73
2.73
2.72
2.71
2.56
2.55
2.54
2.53
2.52
2.50
2.49
2.47
2.24
2.22
2.21
2.19
2.19
2.17
2.16
2.14
2.14
2.07
1.83
1.82
1.82
1.81
1.80
1.80
1.79
1.71
1.70
1.68
1.67
1.65
1.49
1.49
1.47
1.46
1.45
1.44
1.44
1.44
1.41
1.27
1.26
0.88
0.85
0.83
0.00
3.06
1.79
3.12
1.95
1.00
2.05
1.85
2.00
3.92
4.09
1.37
2.02
1.97
10.90
f1 (ppm)

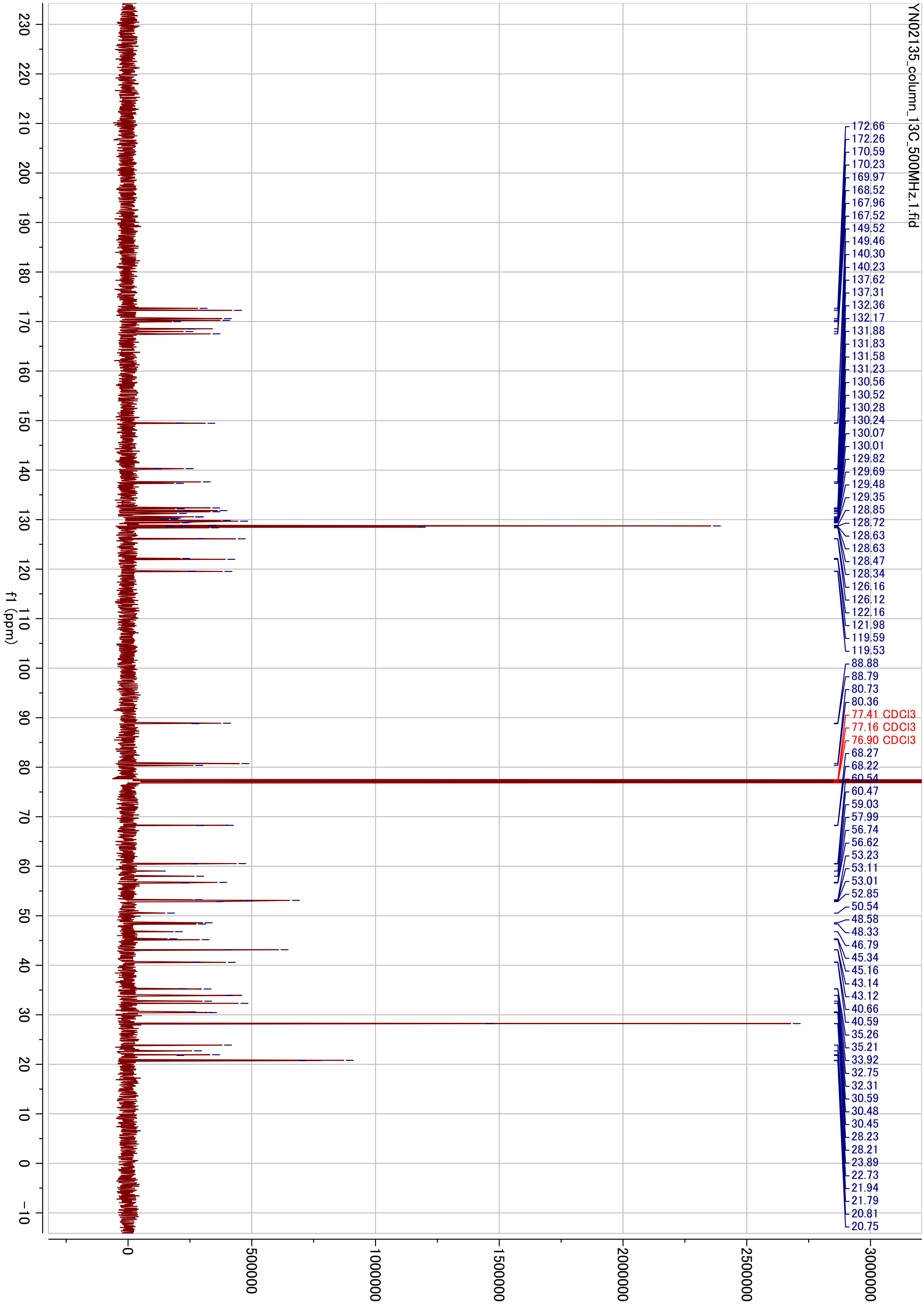

YN02135_column_13C_500MHz.1.fid
172.66
172.26
170.59
170.23
169.97
168.52
167.96
167.52
149.52
149.46
140.30
140.23
137.62
137.31
132.36
132.17
131.88
131.83
131.58
131.23
130.56
130.52
130.28
130.24
130.07
130.01
129.82
129.69
129.48
129.35
128.85
128.72
128.63
128.63
128.47
128.34
126.16
126.12
122.16
121.98
119.59
119.53
88.88
88.79
80.73
80.36
77.41 CDCl3
77.16 CDCl3
76.90 CDCl3
68.27
68.22
60.54
60.47
59.03
57.99
56.74
56.62
53.23
53.11
53.01
52.85
50.54
48.58
48.33
46.79
45.34
45.16
43.14
43.12
40.66
40.59
35.26
35.21
33.92
32.75
32.31
30.59
30.48
30.45
28.23
28.21
23.89
22.73
21.94
21.79
20.81
20.75
f1 (ppm)

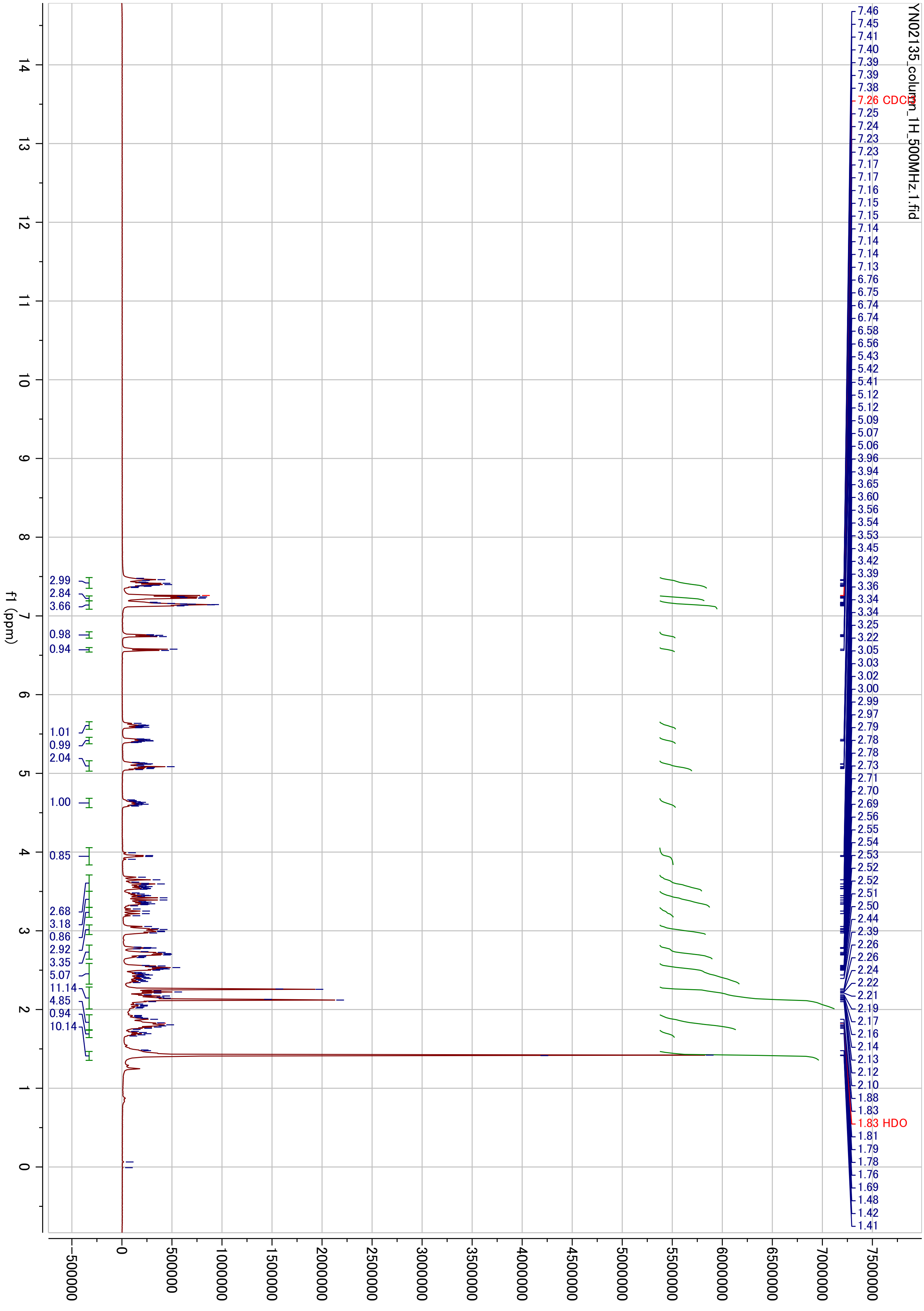
YN02135_column_1H_500MHz.1.fid
7.46
7.45
7.41
7.40
7.39
7.39
7.38
7.26 CDCl3
7.25
7.24
7.23
7.23
7.17
7.17
7.16
7.15
7.15
7.14
7.14
7.14
7.13
6.76
6.75
6.74
6.74
6.58
6.56
5.43
5.42
5.41
5.12
5.12
5.09
5.07
5.06
3.96
3.94
3.65
3.60
3.56
3.54
3.53
3.45
3.42
3.39
3.36
3.34
3.34
3.25
3.22
3.05
3.03
3.02
3.00
2.99
2.97
2.79
2.78
2.78
2.73
2.71
2.70
2.69
2.56
2.55
2.54
2.53
2.52
2.52
2.51
2.50
2.44
2.39
2.26
2.26
2.24
2.22
2.21
2.19
2.17
2.16
2.14
2.13
2.12
2.10
1.88
1.83
1.83 HDO
1.81
1.79
1.78
1.76
1.69
1.48
1.42
1.41
2.99
2.84
3.66
0.98
0.94
1.01
0.99
2.04
1.00
0.85
2.68
3.18
0.86
2.92
3.35
5.07
11.14
4.85
0.94
10.14
f1 (ppm)

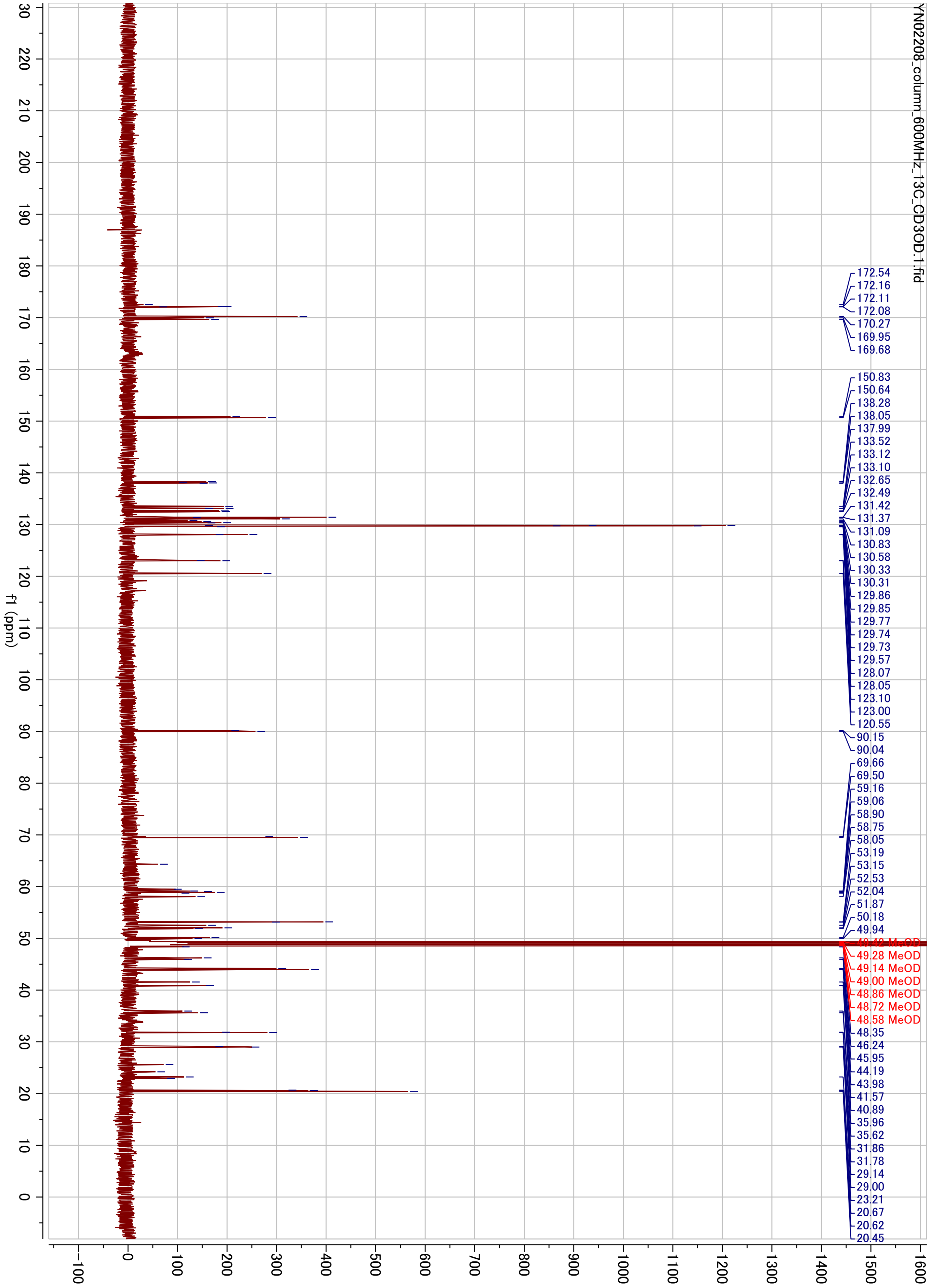
YN02208_column_600MHz_13C_CD3OD.1.fid
172.54
172.16
172.11
172.08
170.27
169.95
169.68
150.83
150.64
138.28
138.05
137.99
133.52
133.12
133.10
132.65
132.49
131.42
131.37
131.09
130.83
130.58
130.33
130.31
129.86
129.85
129.77
129.74
129.73
129.57
128.07
128.05
123.10
123.00
120.55
90.15
90.04
69.66
69.50
59.16
59.06
58.90
58.75
58.05
53.19
53.15
52.53
52.04
51.87
50.18
49.94
49.28 MeOD
49.14 MeOD
49.00 MeOD
48.86 MeOD
48.72 MeOD
48.58 MeOD
48.35
46.24
45.95
44.19
43.98
41.57
40.89
35.96
35.62
31.86
31.78
29.14
29.00
23.21
20.67
20.62
20.45
f1 (ppm)

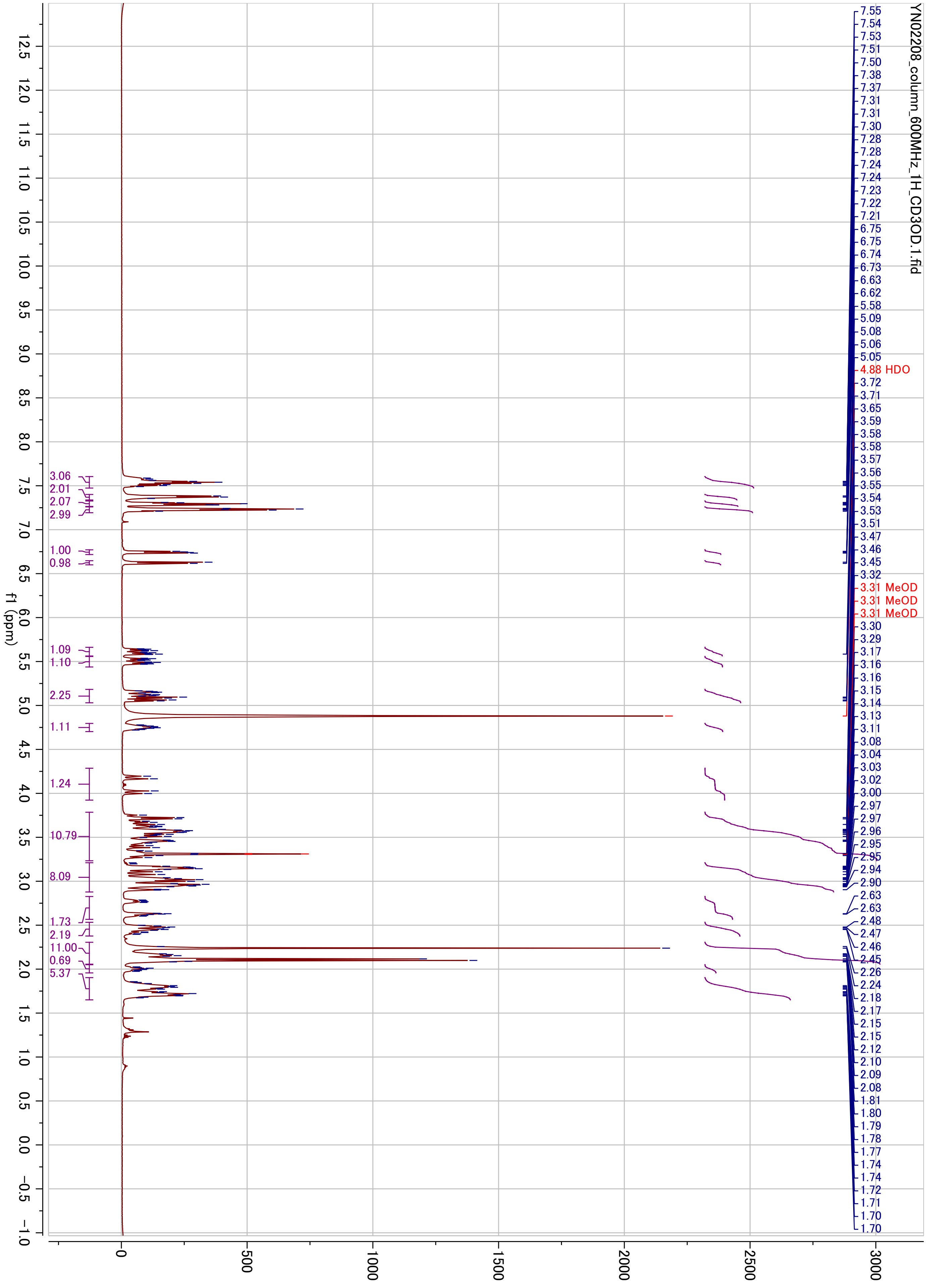
YN02208_column_600MHz_1H_CD3OD.1.fid
7.55
7.54
7.53
7.51
7.50
7.38
7.37
7.31
7.31
7.30
7.28
7.28
7.24
7.24
7.23
7.22
7.21
6.75
6.75
6.74
6.73
6.63
6.62
5.58
5.09
5.08
5.06
5.05
4.88 HDO
3.72
3.71
3.65
3.59
3.58
3.58
3.57
3.56
3.55
3.54
3.53
3.51
3.47
3.46
3.45
3.32
3.31 MeOD
3.31 MeOD
3.31 MeOD
3.30
3.29
3.17
3.16
3.16
3.15
3.14
3.13
3.11
3.08
3.04
3.03
3.02
3.00
2.97
2.97
2.96
2.95
2.95
2.94
2.90
2.63
2.63
2.48
2.47
2.46
2.45
2.26
2.24
2.18
2.17
2.15
2.15
2.12
2.10
2.09
2.08
1.81
1.80
1.79
1.78
1.77
1.74
1.74
1.72
1.71
1.70
1.70
3.06
2.01
2.07
2.99
1.00
0.98
1.09
1.10
2.25
1.11
1.24
10.79
8.09
1.73
2.19
11.00
0.69
5.37
f1 (ppm)

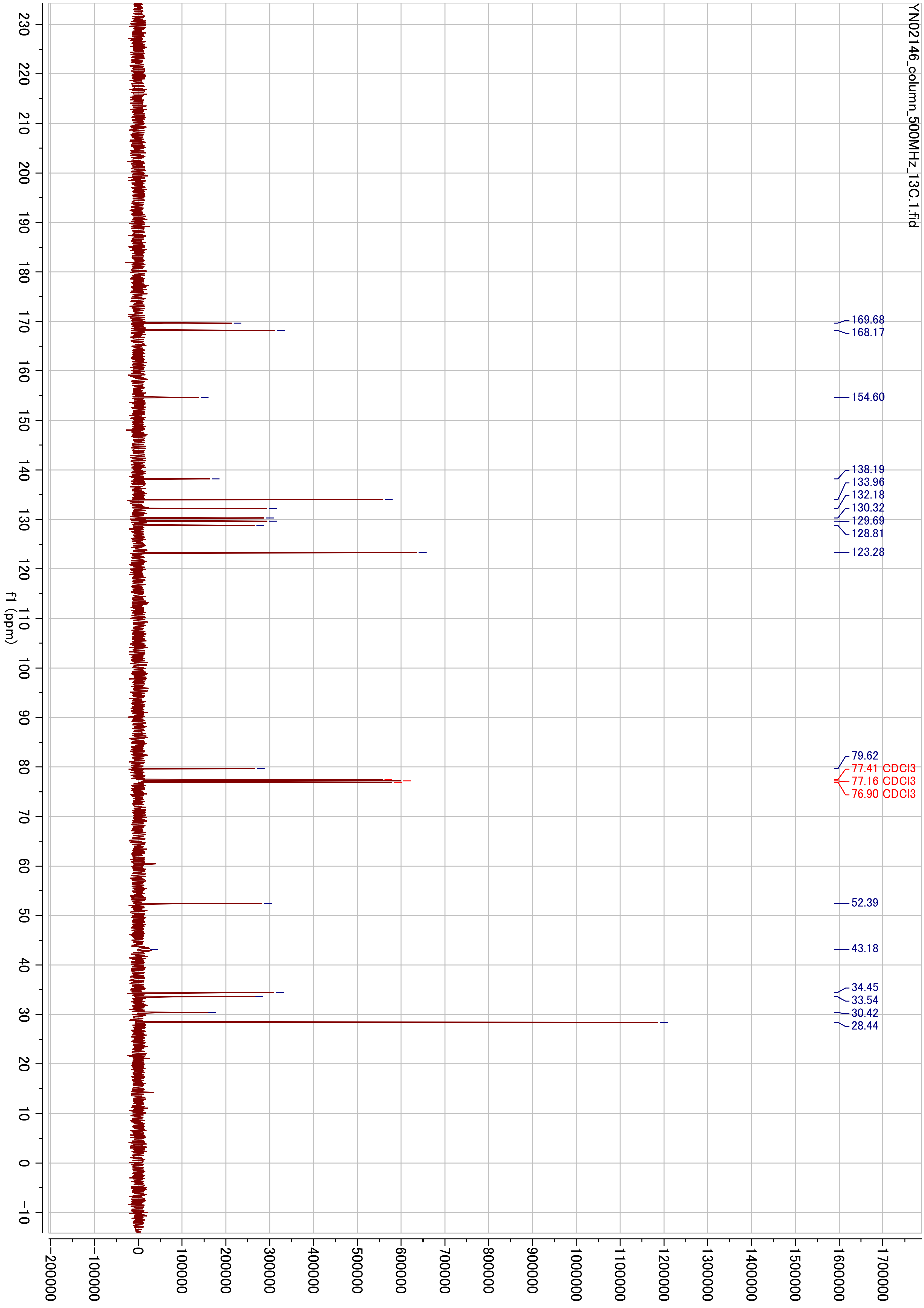
YN02146_column_500MHz_13C.1.fid
169.68
168.17
154.60
138.19
133.96
132.18
130.32
129.69
128.81
123.28
79.62
77.41 CDCl3
77.16 CDCl3
76.90 CDCl3
52.39
43.18
34.45
33.54
30.42
28.44
f1 (ppm)

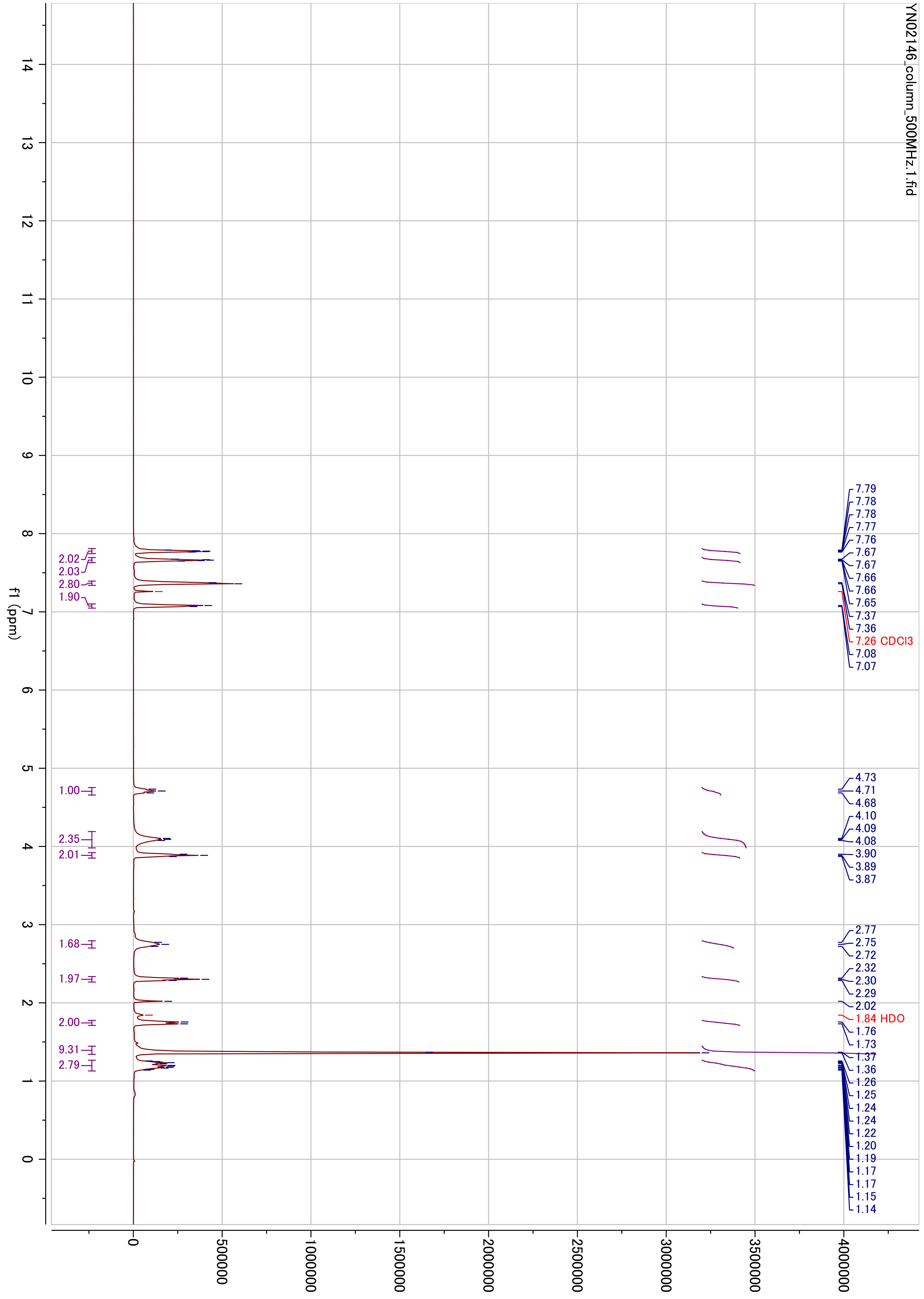
YN02146_column_500MHz.1.fid
7.79
7.78
7.78
7.77
7.76
7.67
7.67
7.66
7.66
7.65
7.37
7.36
7.26 CDCl3
7.08
7.07
4.73
4.71
4.68
4.10
4.09
4.08
3.90
3.89
3.87
2.77
2.75
2.72
2.32
2.30
2.29
2.02
1.84 HDO
1.76
1.73
1.37
1.36
1.26
1.25
1.24
1.24
1.22
1.20
1.19
1.17
1.17
1.15
1.14
2.02
2.03
2.80
1.90
1.00
2.35
2.01
1.68
1.97
2.00
9.31
2.79
f1 (ppm)

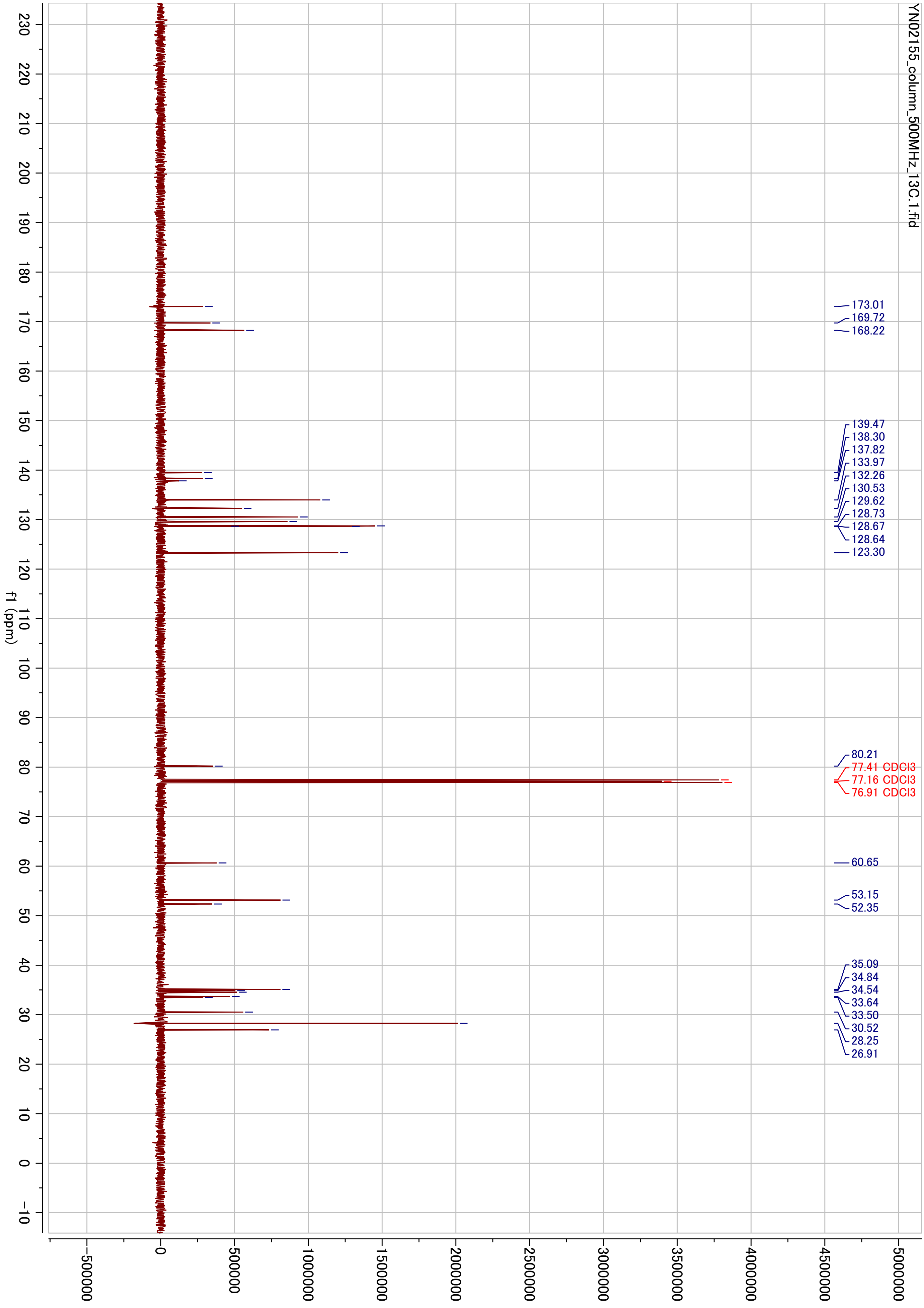
YN02155_column_500MHz_13C.1.fid
173.01
169.72
168.22
139.47
138.30
137.82
133.97
132.26
130.53
129.62
128.73
128.67
128.64
123.30
80.21
77.41 CDCl3
77.16 CDCl3
76.91 CDCl3
60.65
53.15
52.35
35.09
34.84
34.54
33.64
33.50
30.52
28.25
26.91
f1 (ppm)

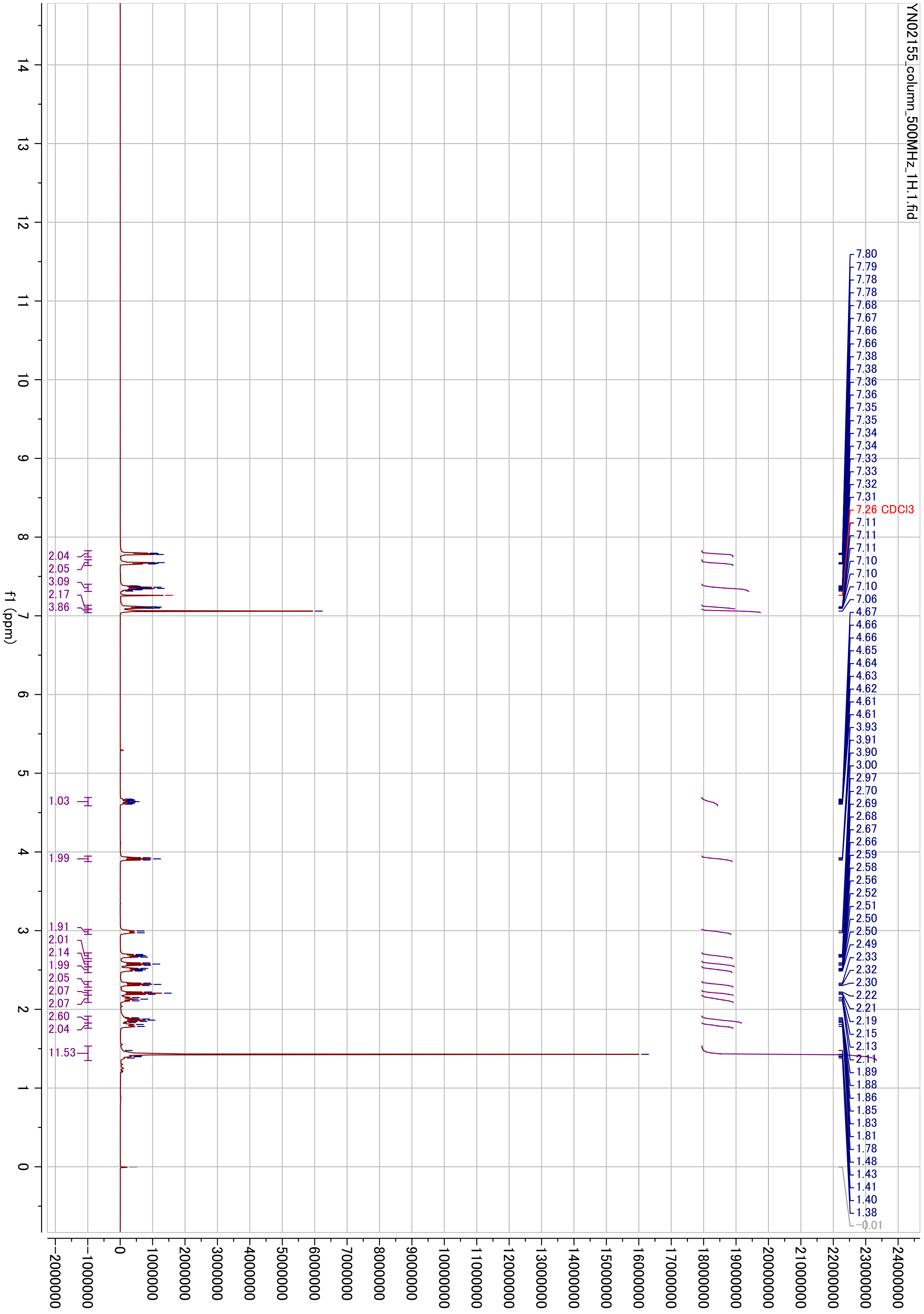
YN02155_column_500MHz_1H.1.fid
f1 (ppm)
7.26 CDCl3

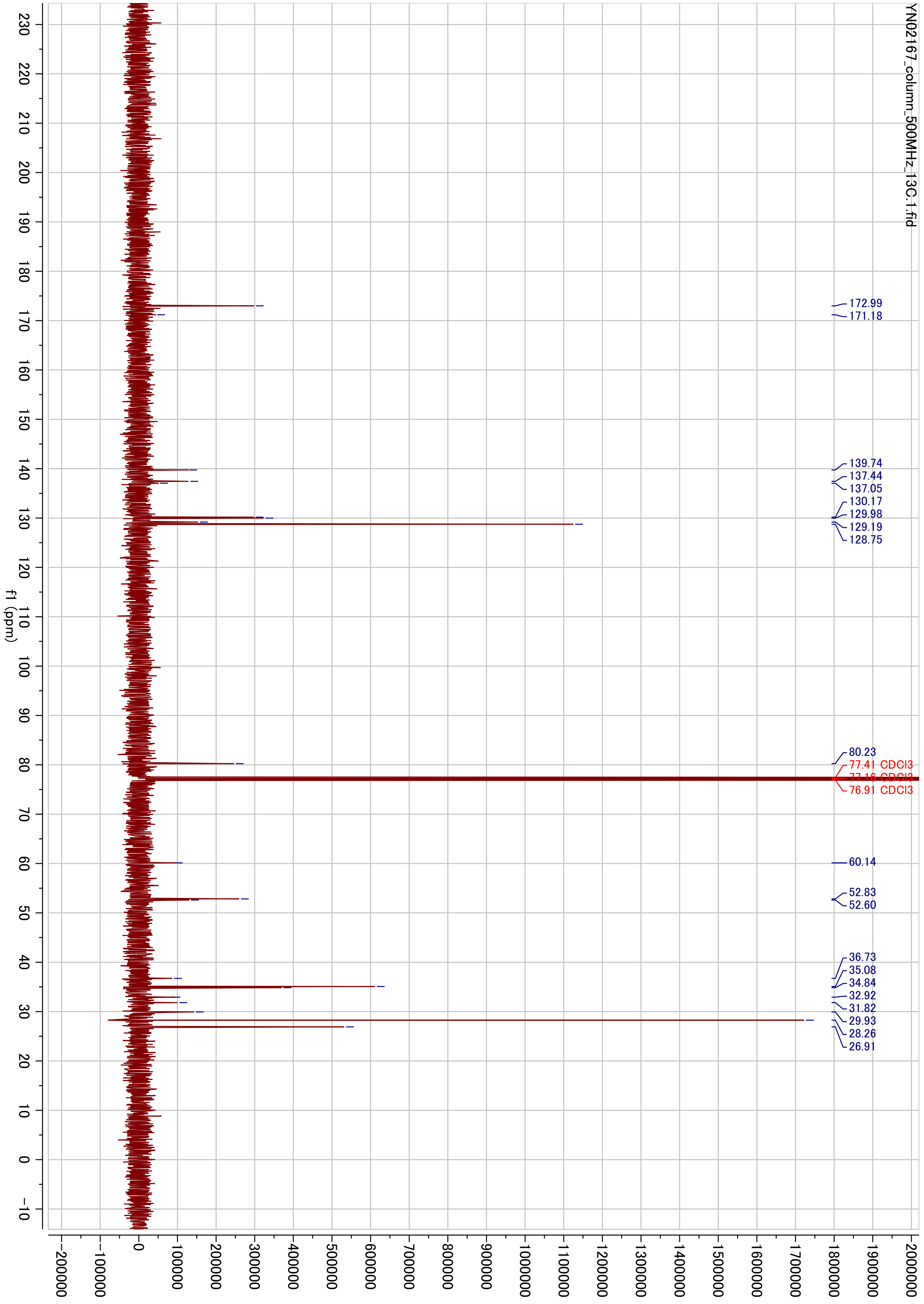
YN02167_column_500MHz_13C.1.fid
172.99
171.18
139.74
137.44
137.05
130.17
129.98
129.19
128.75
80.23
77.41 CDCl3
77.16 CDCl3
76.91 CDCl3
60.14
52.83
52.60
36.73
35.08
34.84
32.92
31.82
29.93
28.26
26.91
f1 (ppm)

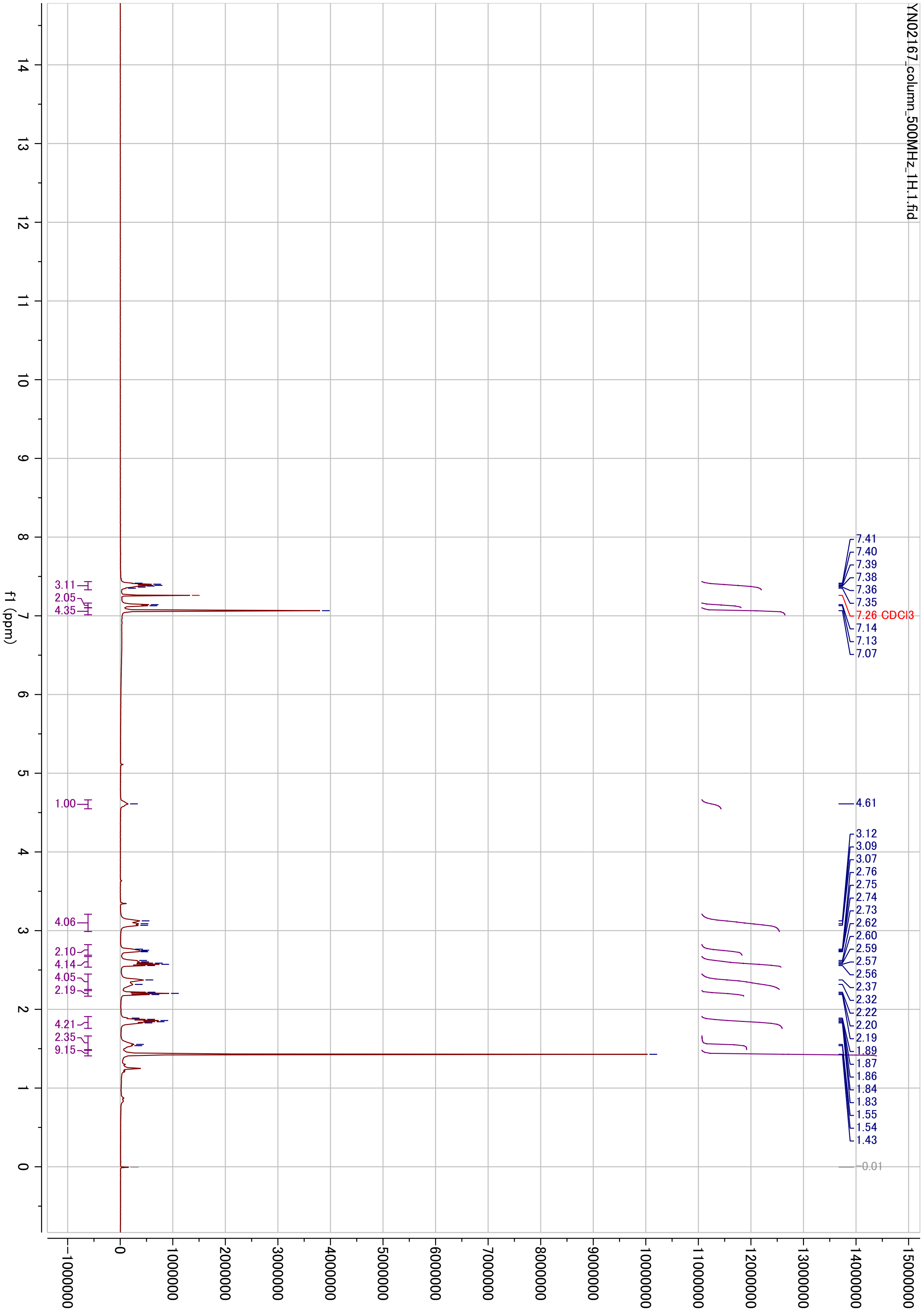
YN02167_column_500MHz_1H.1.fid
7.41
7.40
7.39
7.38
7.36
7.35
7.26 CDCl3
7.14
7.13
7.07
4.61
3.12
3.09
3.07
2.76
2.75
2.74
2.73
2.62
2.60
2.59
2.57
2.56
2.37
2.32
2.22
2.20
2.19
1.89
1.87
1.86
1.84
1.83
1.55
1.54
1.43
-0.01
3.11
2.05
4.35
1.00
4.06
2.10
4.14
4.05
2.19
4.21
2.35
9.15
f1 (ppm)

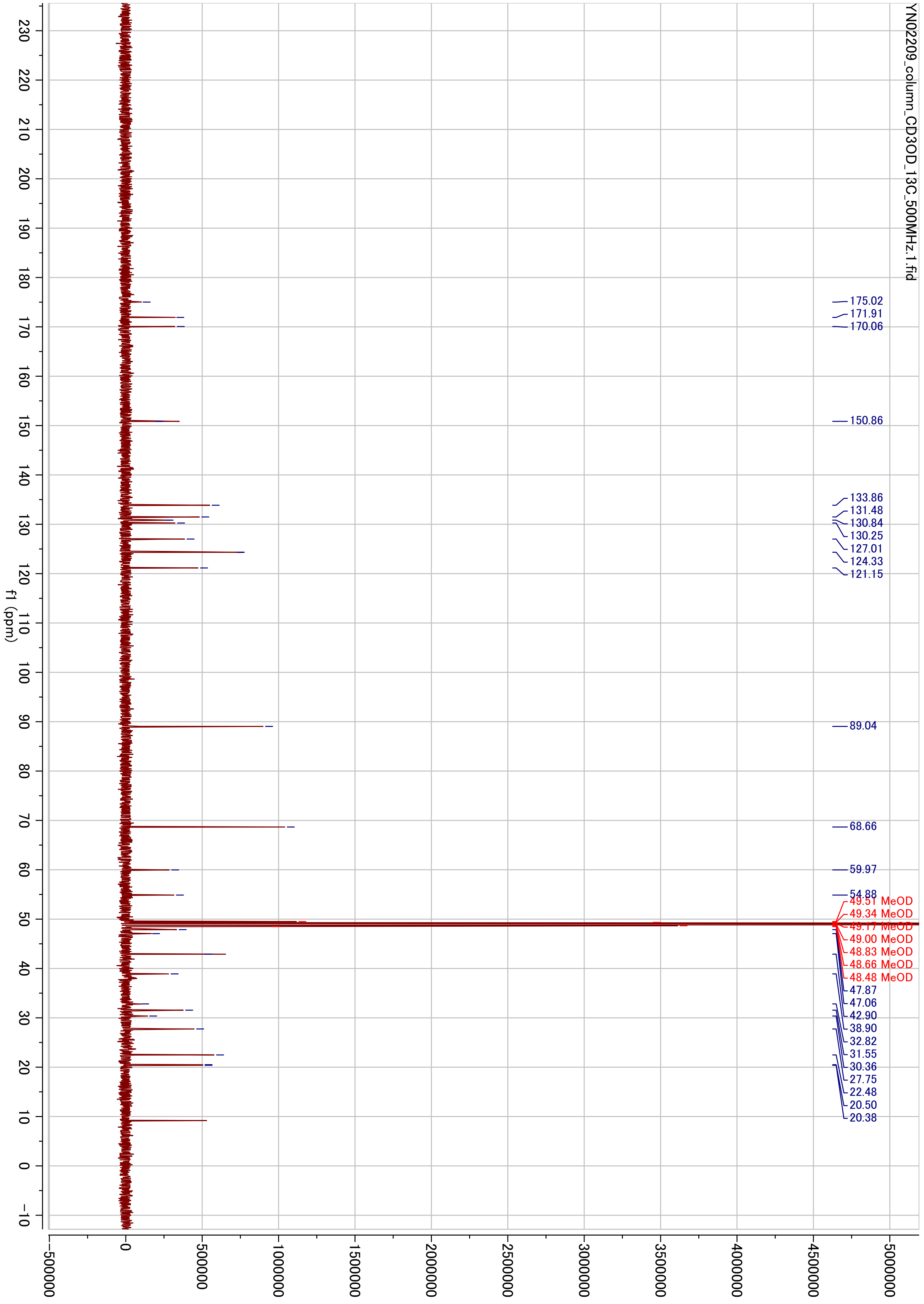
YN02209_column_CD3OD_13C_500MHz.1.fid
175.02
171.91
170.06
150.86
133.86
131.48
130.84
130.25
127.01
124.33
121.15
89.04
68.66
59.97
54.88
49.51 MeOD
49.34 MeOD
49.17 MeOD
49.00 MeOD
48.83 MeOD
48.66 MeOD
48.48 MeOD
47.87
47.06
42.90
38.90
32.82
31.55
30.36
27.75
22.48
20.50
20.38
f1 (ppm)

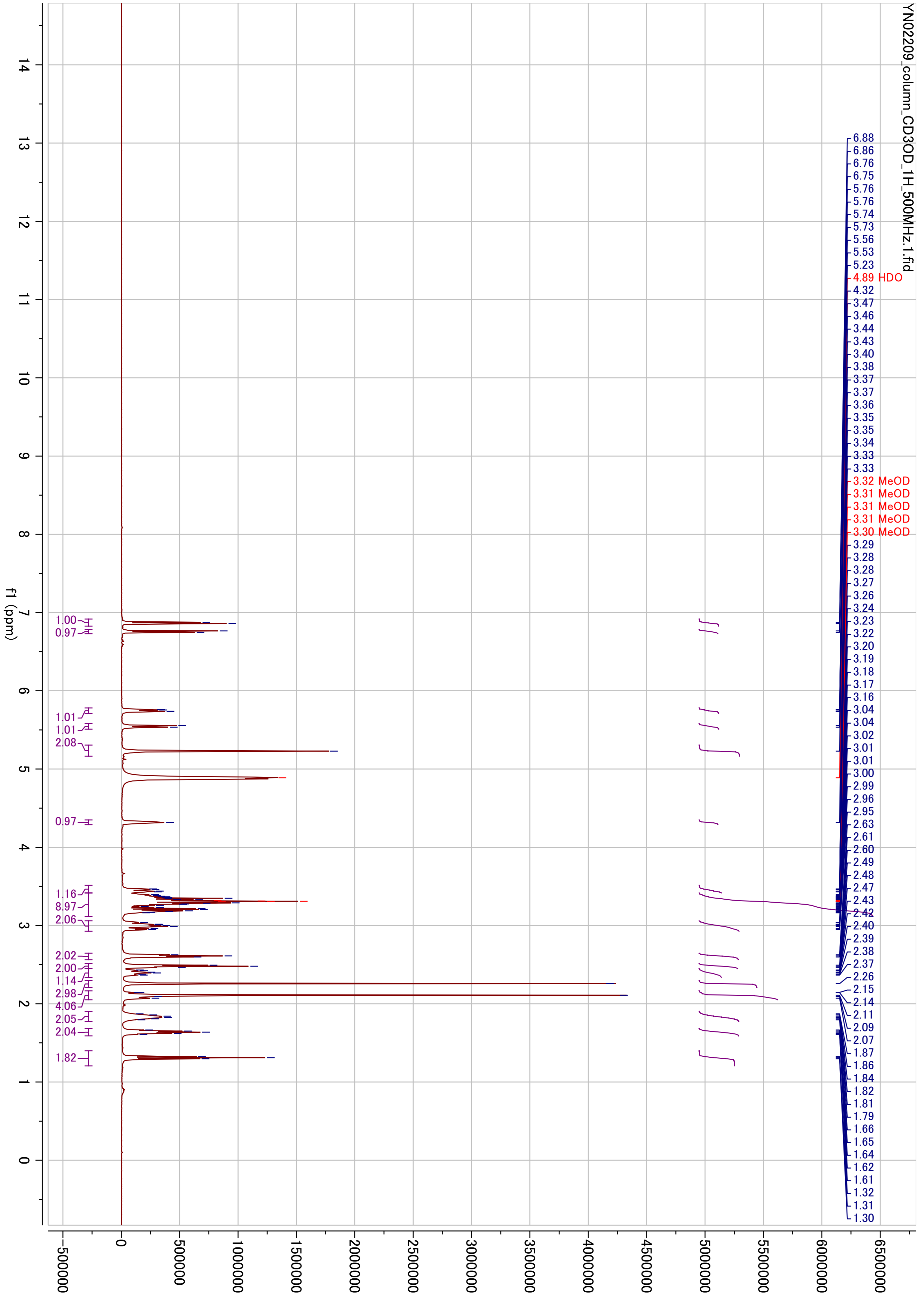

YN02209_column_CD3OD_1H_500MHz.1.fid
f1 (ppm)
6.88
6.86
6.76
6.75
5.76
5.76
5.74
5.73
5.56
5.53
5.23
4.89 HDO
4.32
3.47
3.46
3.44
3.43
3.40
3.38
3.37
3.37
3.36
3.35
3.35
3.34
3.33
3.33
3.32 MeOD
3.31 MeOD
3.31 MeOD
3.31 MeOD
3.30 MeOD
3.29
3.28
3.28
3.27
3.26
3.24
3.23
3.22
3.20
3.19
3.18
3.17
3.16
3.04
3.04
3.02
3.01
3.01
3.00
2.99
2.96
2.95
2.63
2.61
2.60
2.49
2.48
2.47
2.43
2.42
2.40
2.39
2.38
2.37
2.26
2.15
2.14
2.11
2.09
2.07
1.87
1.86
1.84
1.82
1.81
1.79
1.66
1.65
1.64
1.62
1.61
1.32
1.31
1.30
1.00
0.97
1.01
1.01
2.08
0.97
1.16
8.97
2.06
2.02
2.00
1.14
2.98
4.06
2.05
2.04
1.82

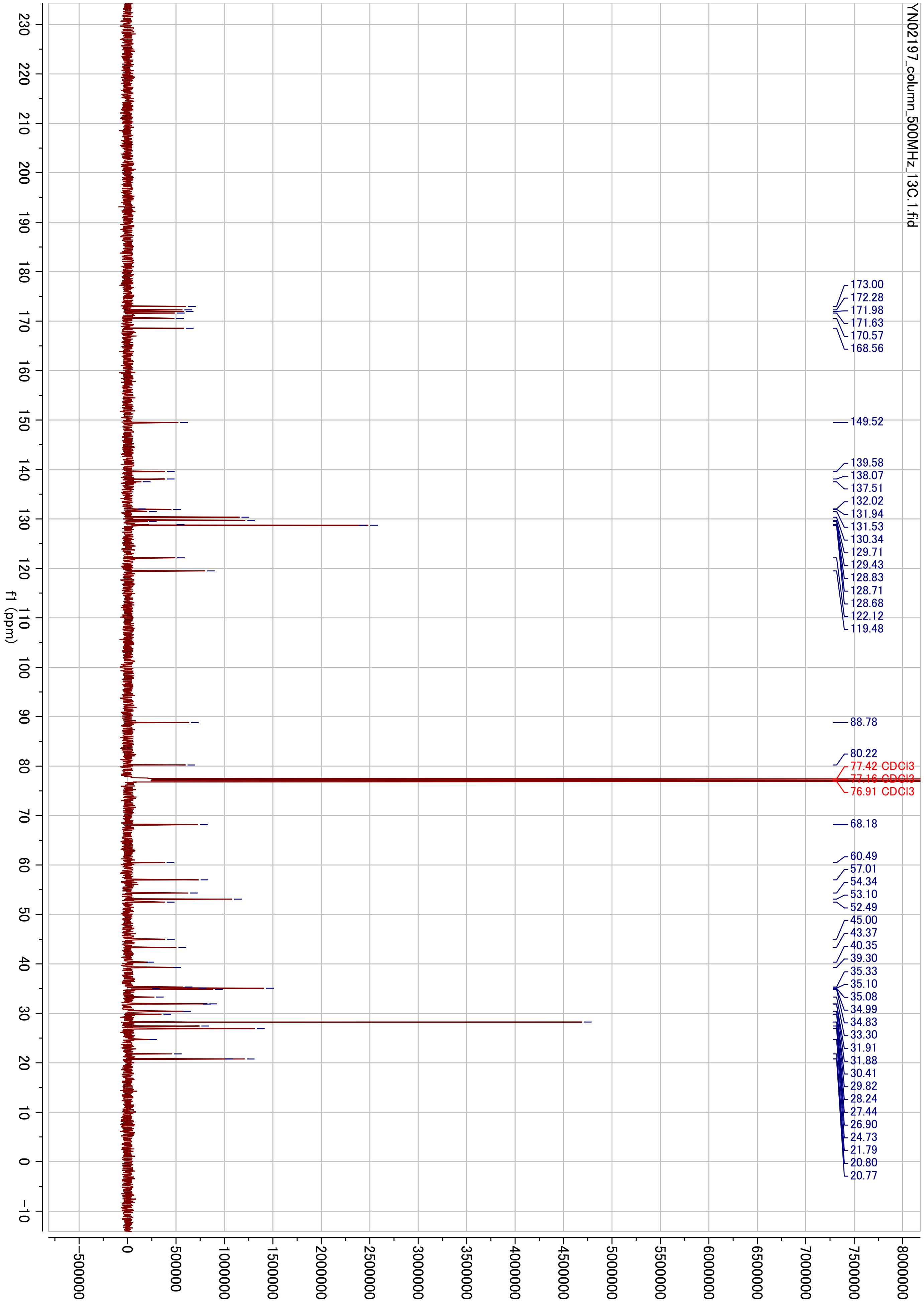
YN02197_column_500MHz_13C.1.fid
173.00
172.28
171.98
171.63
170.57
168.56
149.52
139.58
138.07
137.51
132.02
131.94
131.53
130.34
129.71
129.43
128.83
128.71
128.68
122.12
119.48
88.78
80.22
77.42 CDCl3
77.16 CDCl3
76.91 CDCl3
68.18
60.49
57.01
54.34
53.10
52.49
45.00
43.37
40.35
39.30
35.33
35.10
35.08
34.99
34.83
33.30
31.91
31.88
30.41
29.82
28.24
27.44
26.90
24.73
21.79
20.80
20.77
f1 (ppm)

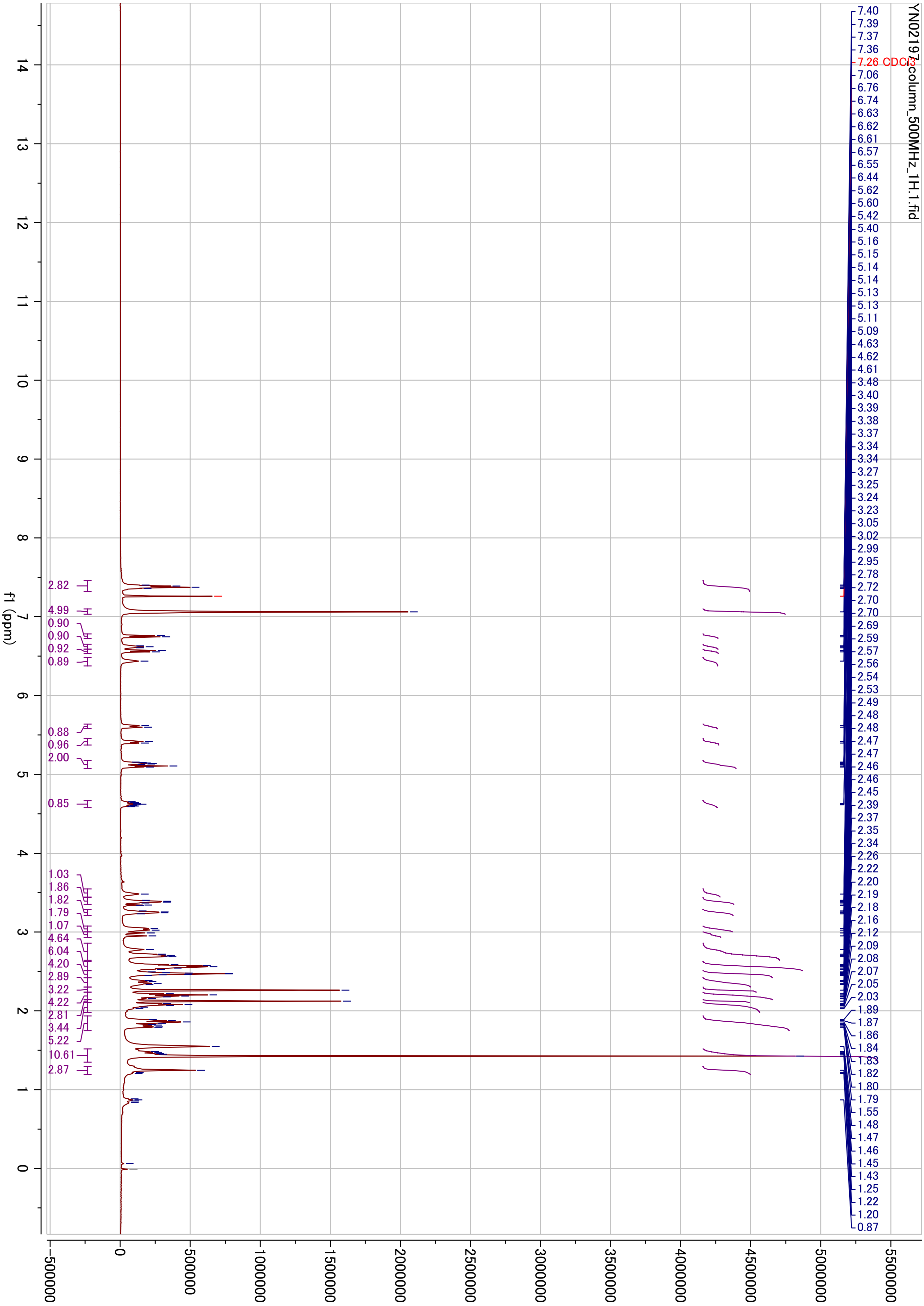
YN0219?_column_500MHz_1H.1.fid
7.40
7.39
7.37
7.36
7.26 CDCl3
7.06
6.76
6.74
6.63
6.62
6.61
6.57
6.55
6.44
5.62
5.60
5.42
5.40
5.16
5.15
5.14
5.14
5.13
5.13
5.11
5.09
4.63
4.62
4.61
3.48
3.40
3.39
3.38
3.37
3.34
3.34
3.27
3.25
3.24
3.23
3.05
3.02
2.99
2.95
2.78
2.72
2.70
2.70
2.69
2.59
2.57
2.56
2.54
2.53
2.49
2.48
2.48
2.47
2.47
2.46
2.46
2.45
2.39
2.37
2.35
2.34
2.26
2.22
2.20
2.19
2.18
2.16
2.12
2.09
2.08
2.07
2.05
2.03
1.89
1.87
1.86
1.84
1.83
1.82
1.80
1.79
1.55
1.48
1.47
1.46
1.45
1.43
1.25
1.22
1.20
0.87
2.82
4.99
0.90
0.90
0.92
0.89
0.88
0.96
2.00
0.85
1.03
1.86
1.82
1.79
1.07
4.64
6.04
4.20
2.89
3.22
4.22
2.81
3.44
5.22
10.61
2.87
f1 (ppm)

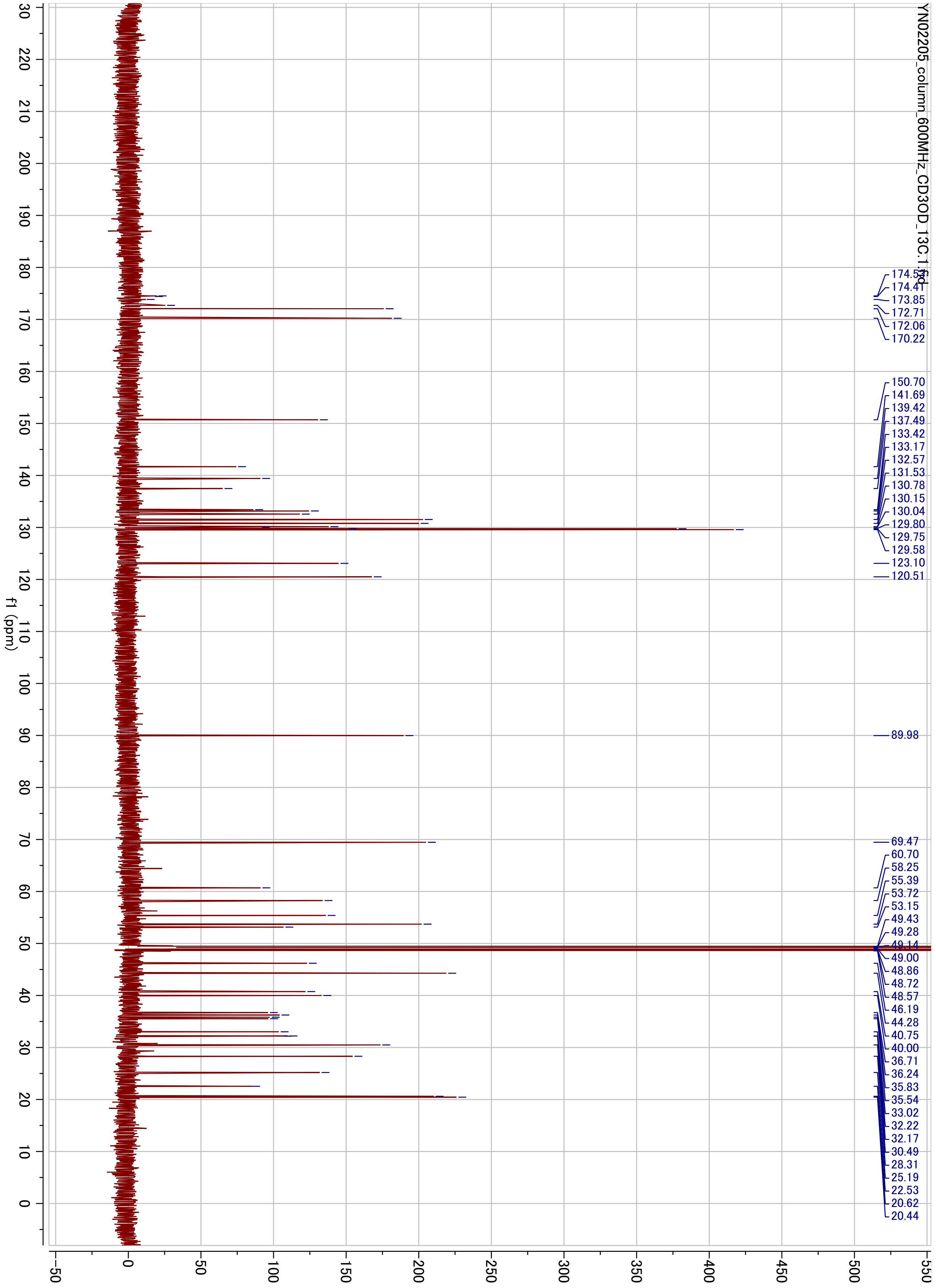

YN02205_column_600MHz_CD3OD_13C.1.fid
174.53
174.41
173.85
172.71
172.06
170.22
150.70
141.69
139.42
137.49
133.42
133.17
132.57
131.53
130.78
130.15
130.04
129.80
129.75
129.58
123.10
120.51
89.98
69.47
60.70
58.25
55.39
53.72
53.15
49.43
49.28
49.14
49.00
48.86
48.72
48.57
46.19
44.28
40.75
40.00
36.71
36.24
35.83
35.54
33.02
32.22
32.17
30.49
28.31
25.19
22.53
20.62
20.44
f1 (ppm)

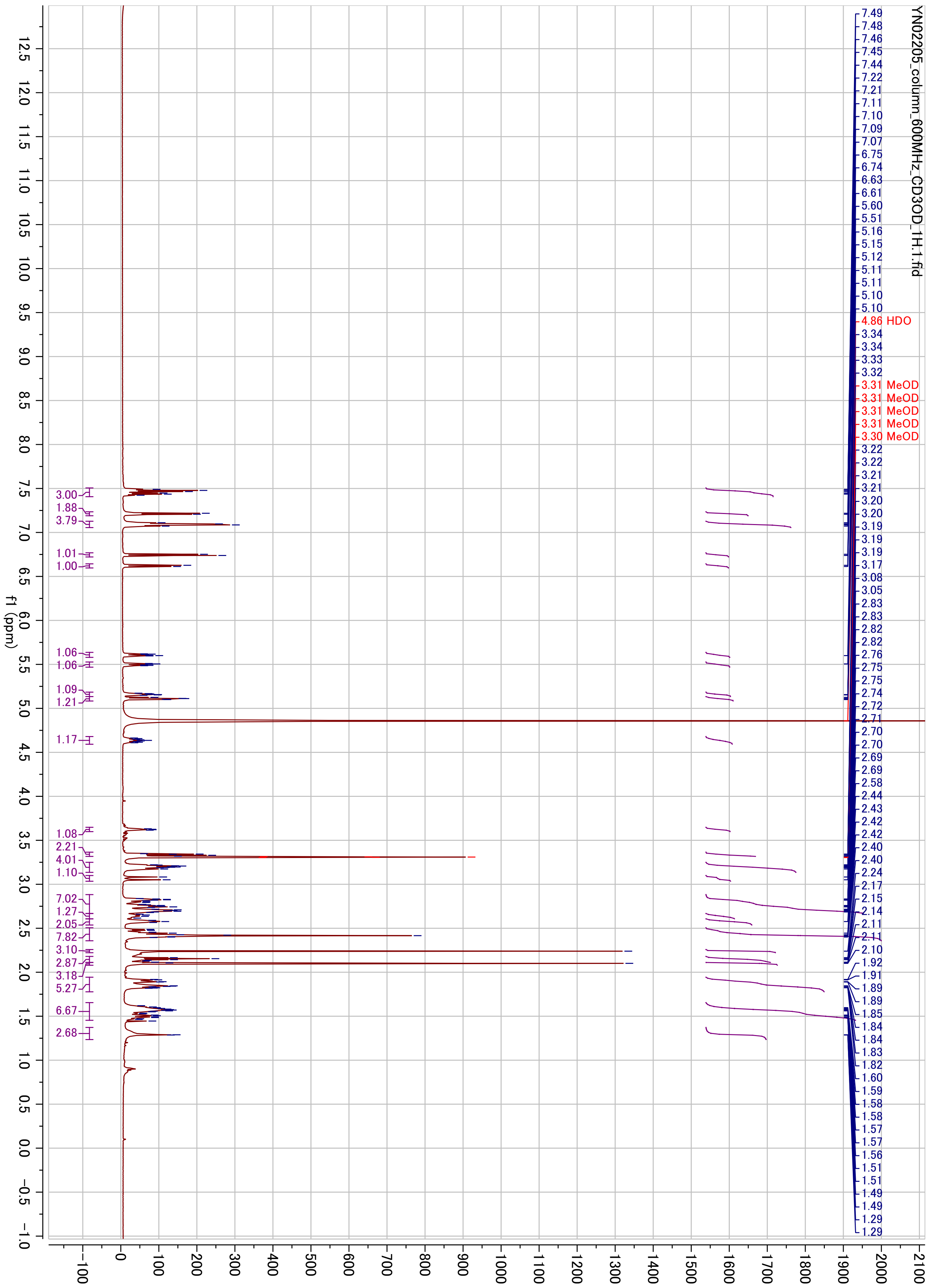
YN02205_column_600MHz_CD3OD_1H.1.fid
f1 (ppm)
4.86 HDO
3.31 MeOD
3.30 MeOD

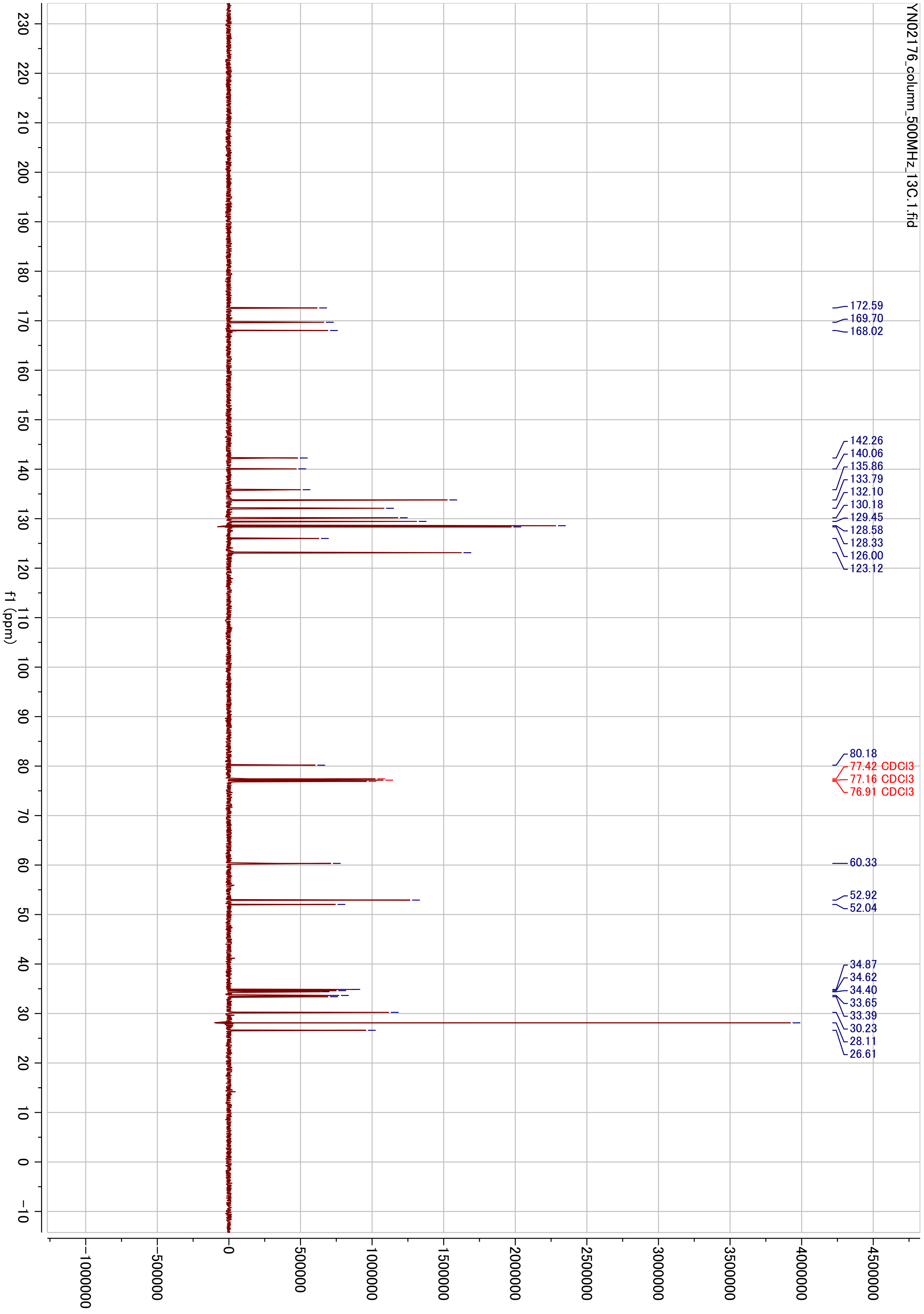
YN02176_column_500MHz_13C.1.fid
172.59
169.70
168.02
142.26
140.06
135.86
133.79
132.10
130.18
129.45
128.58
128.33
126.00
123.12
80.18
77.42 CDCl3
77.16 CDCl3
76.91 CDCl3
60.33
52.92
52.04
34.87
34.62
34.40
33.65
33.39
30.23
28.11
26.61
f1 (ppm)

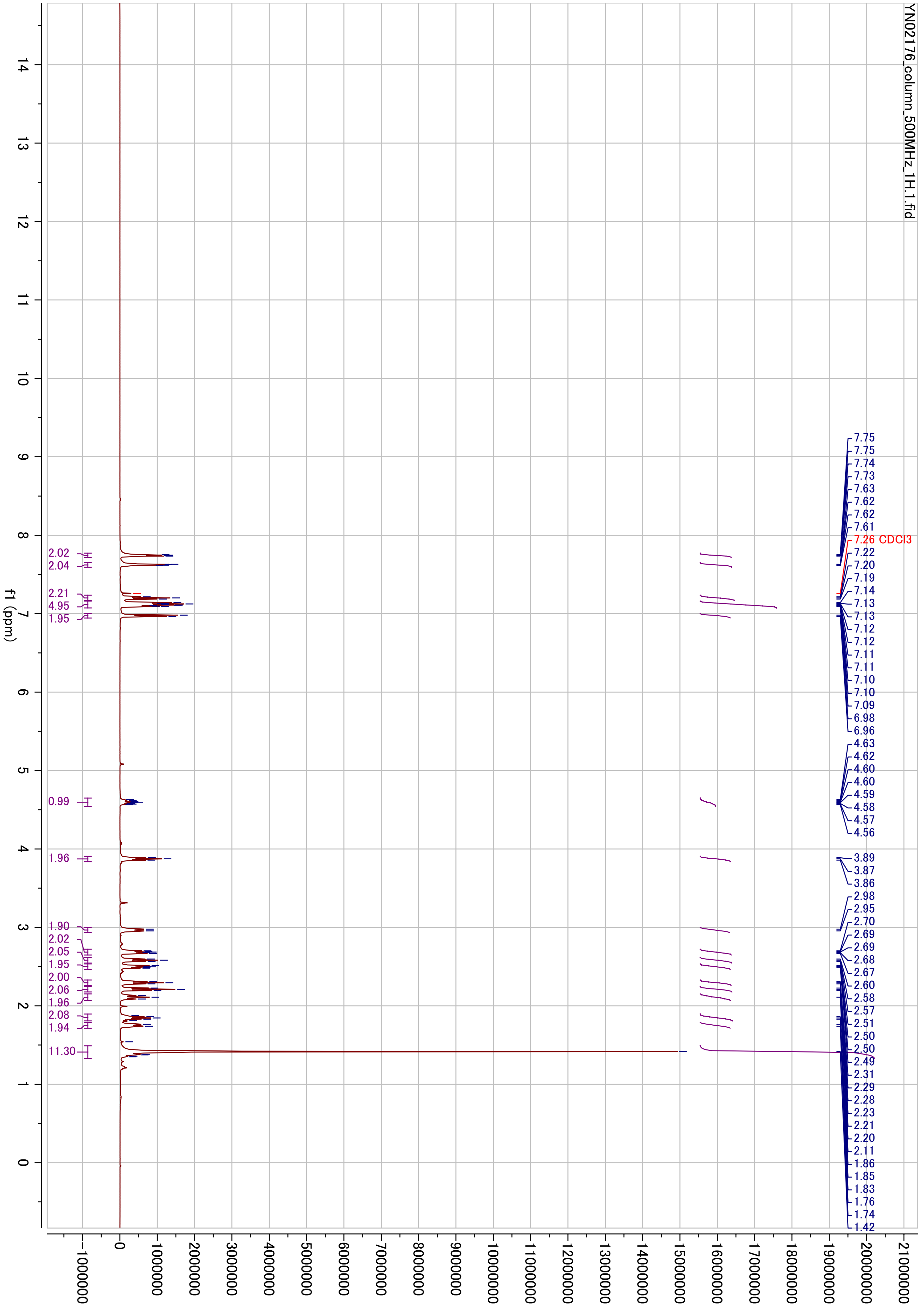
YN02176_column_500MHz_1H.1.fid
f1 (ppm)
2.02
2.04
2.21
4.95
1.95
0.99
1.96
1.90
2.02
2.05
1.95
2.00
2.06
1.96
2.08
1.94
11.30
7.75
7.75
7.74
7.73
7.63
7.62
7.62
7.61
7.26 CDCl3
7.22
7.20
7.19
7.14
7.13
7.13
7.12
7.12
7.11
7.11
7.10
7.10
7.09
6.98
6.96
4.63
4.62
4.60
4.60
4.59
4.58
4.57
4.56
3.89
3.87
3.86
2.98
2.95
2.70
2.69
2.69
2.68
2.67
2.60
2.58
2.57
2.51
2.50
2.50
2.49
2.31
2.29
2.28
2.23
2.21
2.20
2.11
1.86
1.85
1.83
1.76
1.74
1.42

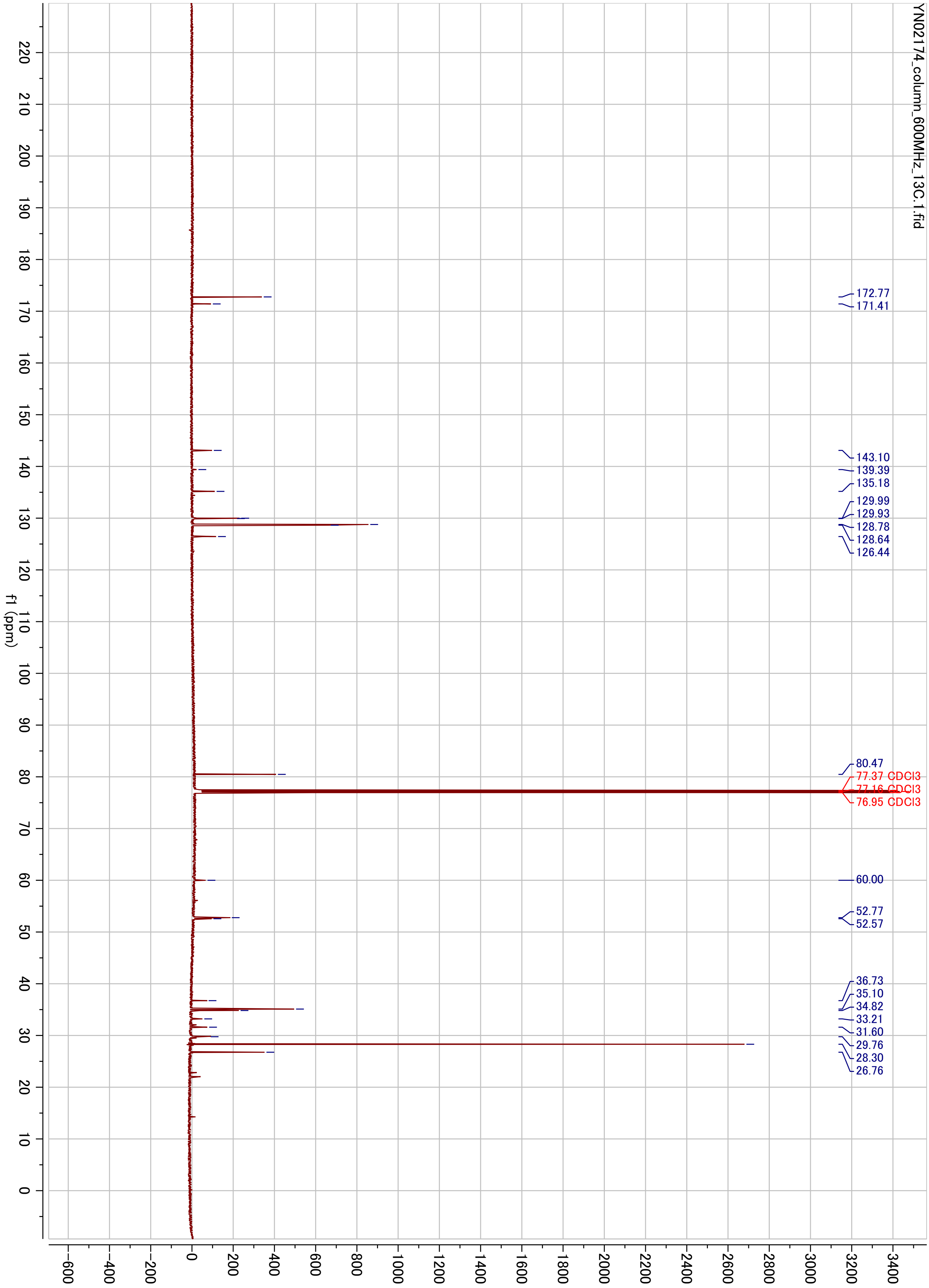
YN02174_column_600MHz_13C.1.fid
172.77
171.41
143.10
139.39
135.18
129.99
129.93
128.78
128.64
126.44
80.47
77.37 CDCl3
77.16 CDCl3
76.95 CDCl3
60.00
52.77
52.57
36.73
35.10
34.82
33.21
31.60
29.76
28.30
26.76
f1 (ppm)

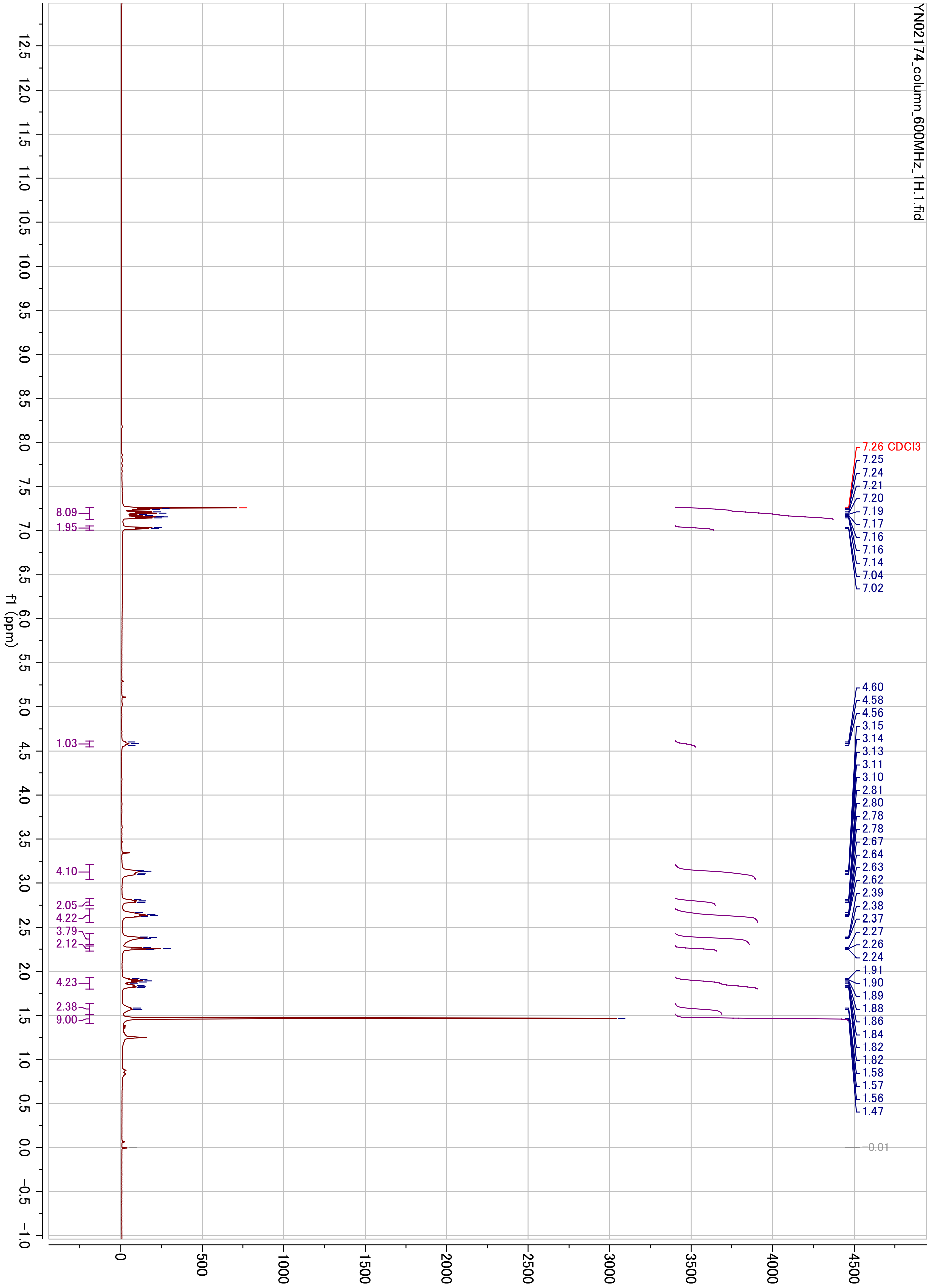
YN02174_column_600MHz_1H.1.fid
7.26 CDCl3
7.25
7.24
7.21
7.20
7.19
7.17
7.16
7.16
7.14
7.04
7.02
4.60
4.58
4.56
3.15
3.14
3.13
3.11
3.10
2.81
2.80
2.78
2.78
2.67
2.64
2.63
2.62
2.39
2.38
2.37
2.27
2.26
2.24
1.91
1.90
1.89
1.88
1.86
1.84
1.82
1.82
1.58
1.57
1.56
1.47
-0.01
8.09
1.95
1.03
4.10
2.05
4.22
3.79
2.12
4.23
2.38
9.00
f1 (ppm)

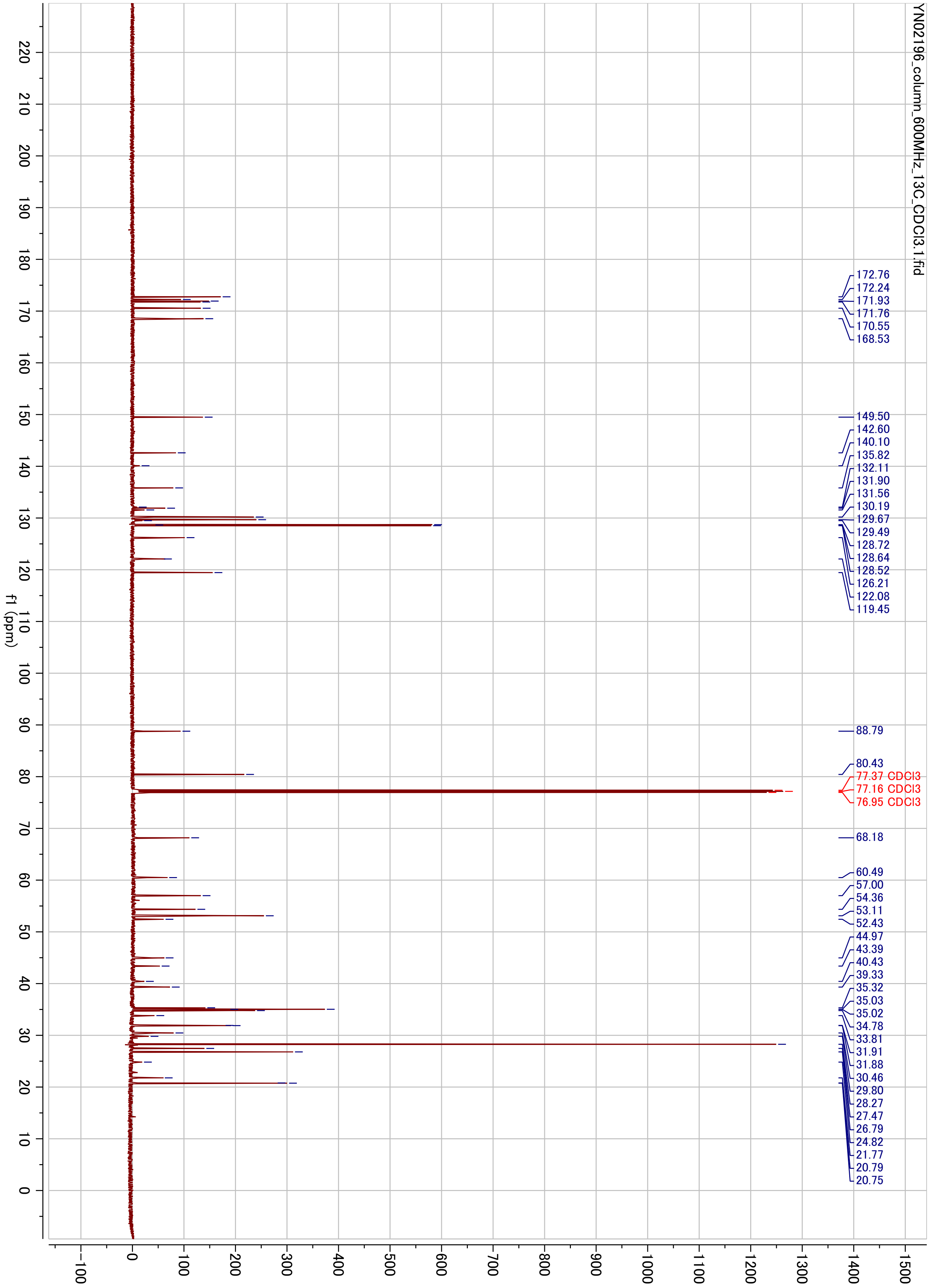
YN02196_column_600MHz_13C_CDCl3.1.fid
172.76
172.24
171.93
171.76
170.55
168.53
149.50
142.60
140.10
135.82
132.11
131.90
131.56
130.19
129.67
129.49
128.72
128.64
128.52
126.21
122.08
119.45
88.79
80.43
77.37 CDCl3
77.16 CDCl3
76.95 CDCl3
68.18
60.49
57.00
54.36
53.11
52.43
44.97
43.39
40.43
39.33
35.32
35.03
35.02
34.78
33.81
31.91
31.88
30.46
29.80
28.27
27.47
26.79
24.82
21.77
20.79
20.75
f1 (ppm)

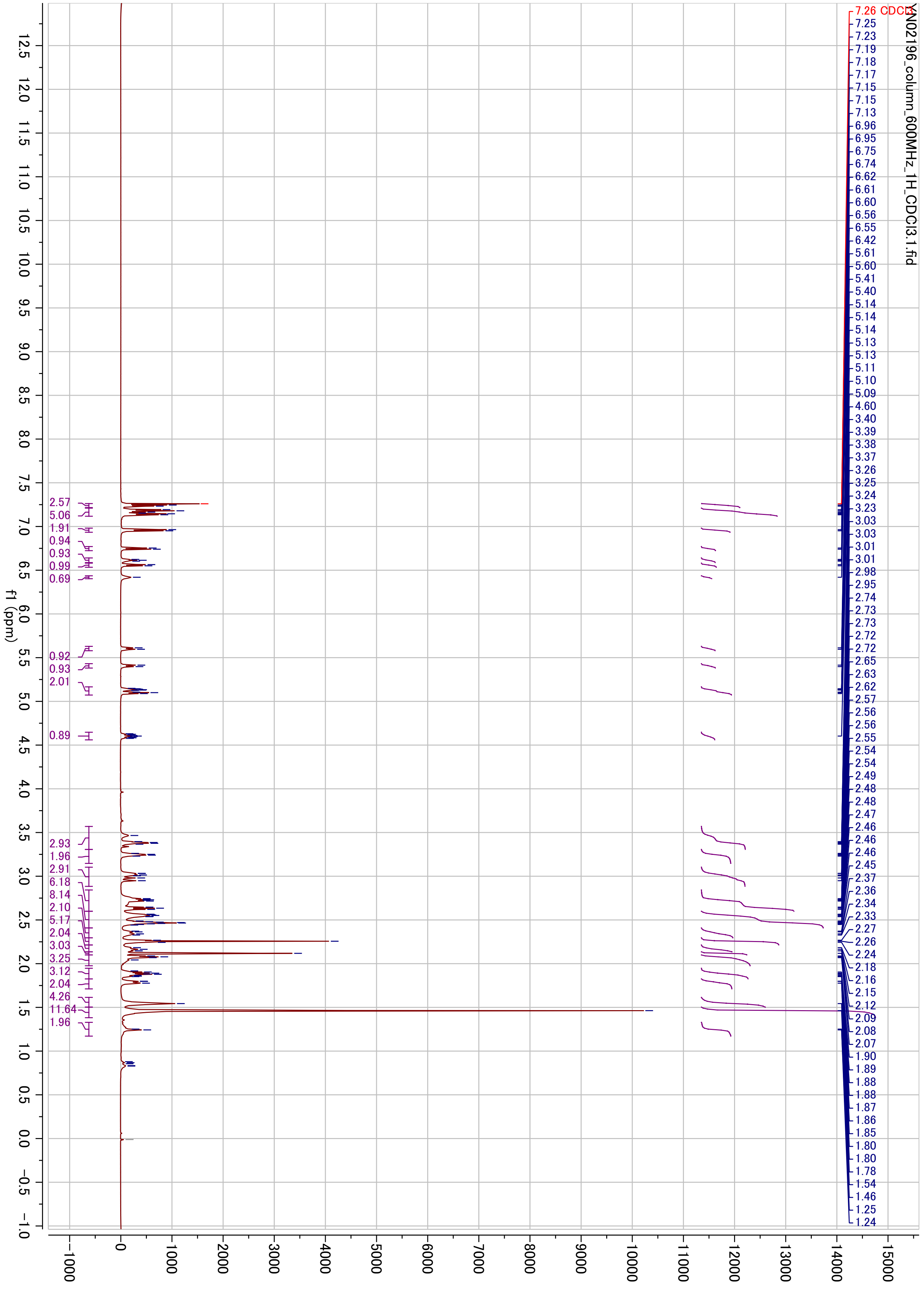
N02196_column_600MHz_1H_CDCl3.1.fid
f1 (ppm)

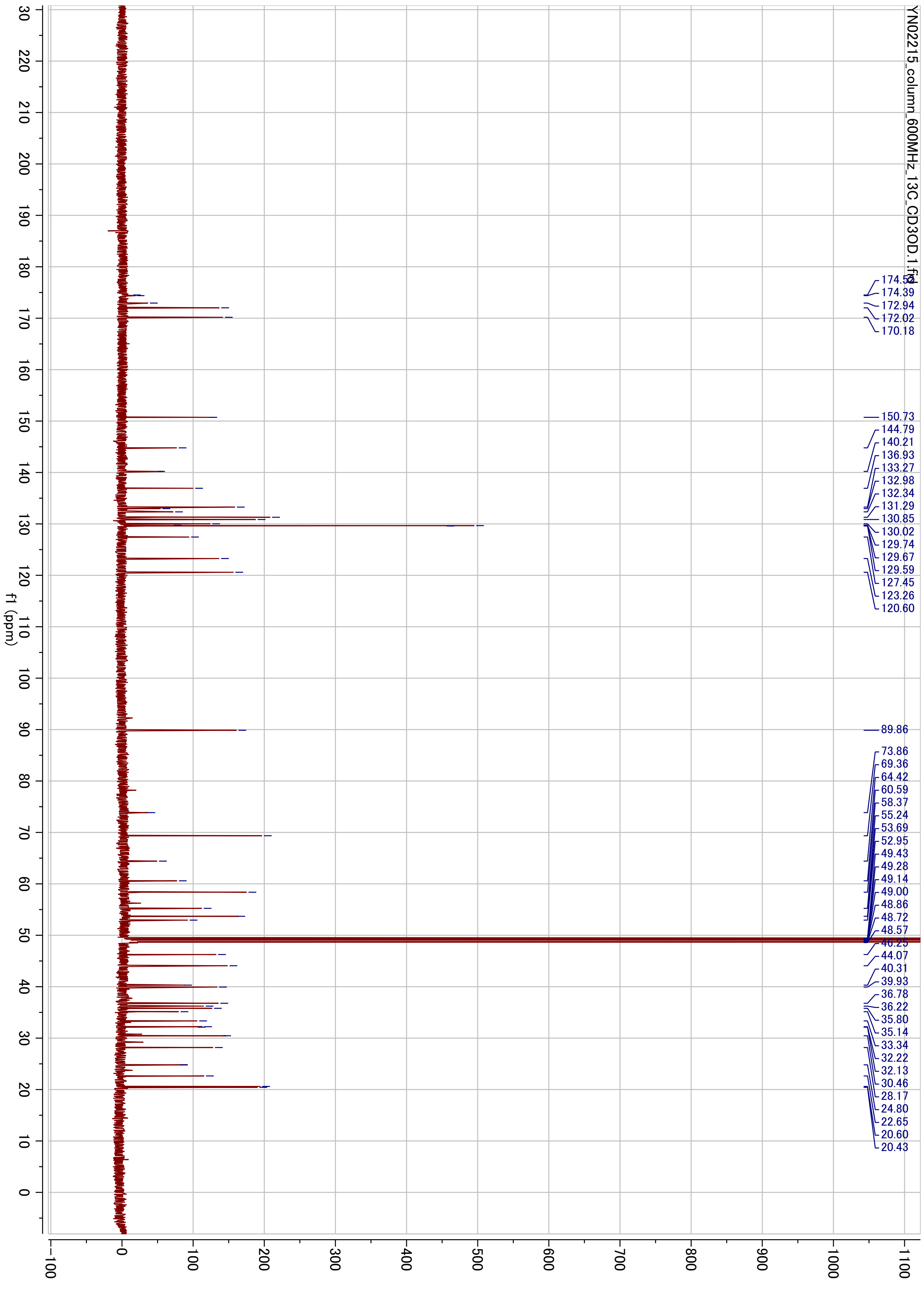

YN02215_column_600MHz_13C_CD3OD.1.fid
174.52
174.39
172.94
172.02
170.18
150.73
144.79
140.21
136.93
133.27
132.98
132.34
131.29
130.85
130.02
129.74
129.67
129.59
127.45
123.26
120.60
89.86
73.86
69.36
64.42
60.59
58.37
55.24
53.69
52.95
49.43
49.28
49.14
49.00
48.86
48.72
48.57
46.25
44.07
40.31
39.93
36.78
36.22
35.80
35.14
33.34
32.22
32.13
30.46
28.17
24.80
22.65
20.60
20.43
f1 (ppm)

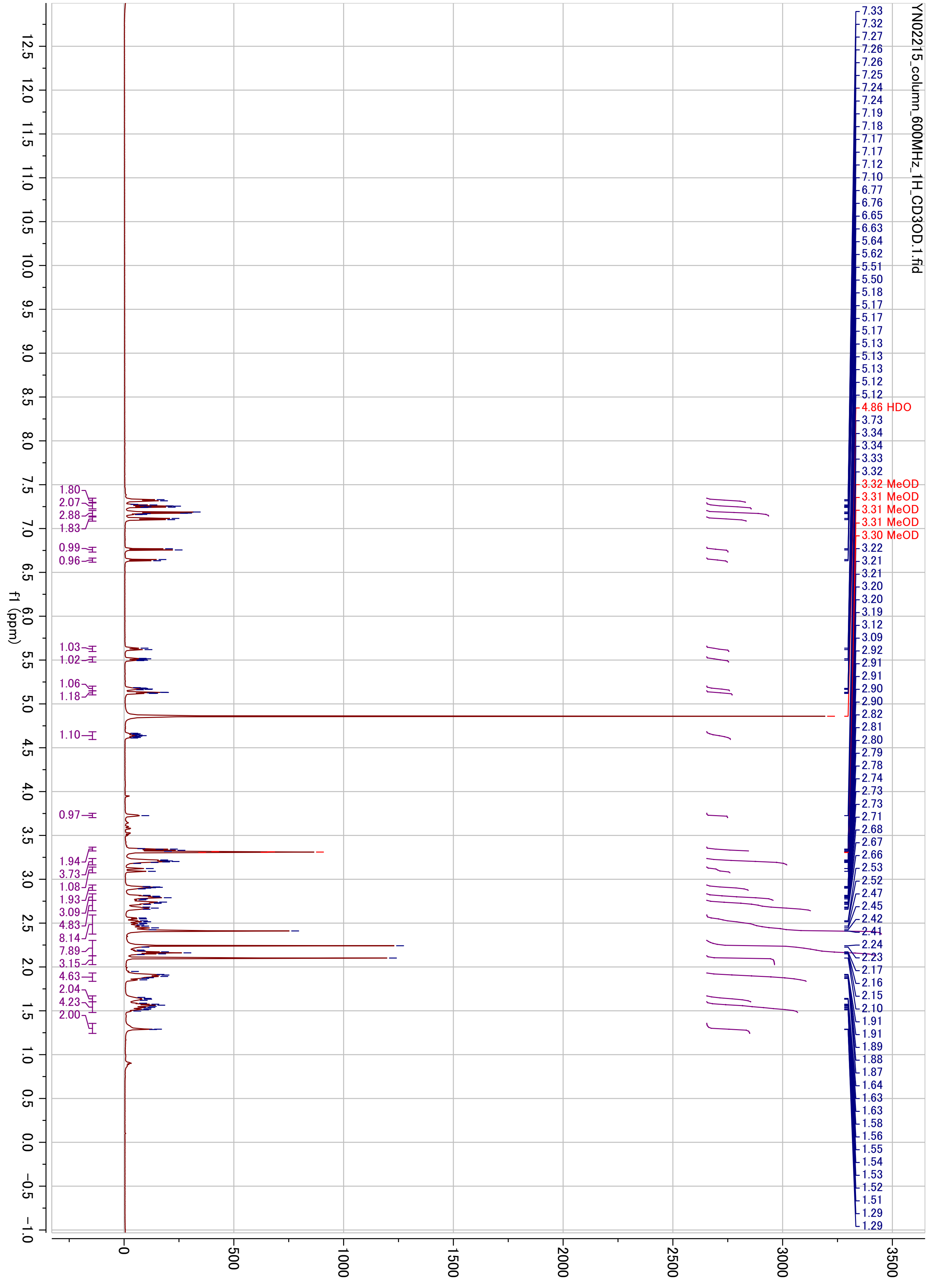
YN02215_column_600MHz_1H_CD3OD.1.fid
7.33
7.32
7.27
7.26
7.26
7.25
7.24
7.24
7.19
7.18
7.17
7.17
7.12
7.10
6.77
6.76
6.65
6.63
5.64
5.62
5.51
5.50
5.18
5.17
5.17
5.17
5.13
5.13
5.13
5.12
5.12
4.86 HDO
3.73
3.34
3.34
3.33
3.32
3.32 MeOD
3.31 MeOD
3.31 MeOD
3.31 MeOD
3.30 MeOD
3.22
3.21
3.21
3.20
3.20
3.19
3.12
3.09
2.92
2.91
2.91
2.90
2.90
2.82
2.81
2.80
2.79
2.78
2.74
2.73
2.73
2.71
2.68
2.67
2.66
2.53
2.52
2.47
2.45
2.42
2.41
2.24
2.23
2.17
2.16
2.15
2.10
1.91
1.91
1.89
1.88
1.87
1.64
1.63
1.63
1.58
1.56
1.55
1.54
1.53
1.52
1.51
1.29
1.29
1.80
2.07
2.88
1.83
0.99
0.96
1.03
1.02
1.06
1.18
1.10
0.97
1.94
3.73
1.08
1.93
3.09
4.83
8.14
7.89
3.15
4.63
2.04
4.23
2.00
f1 (ppm)

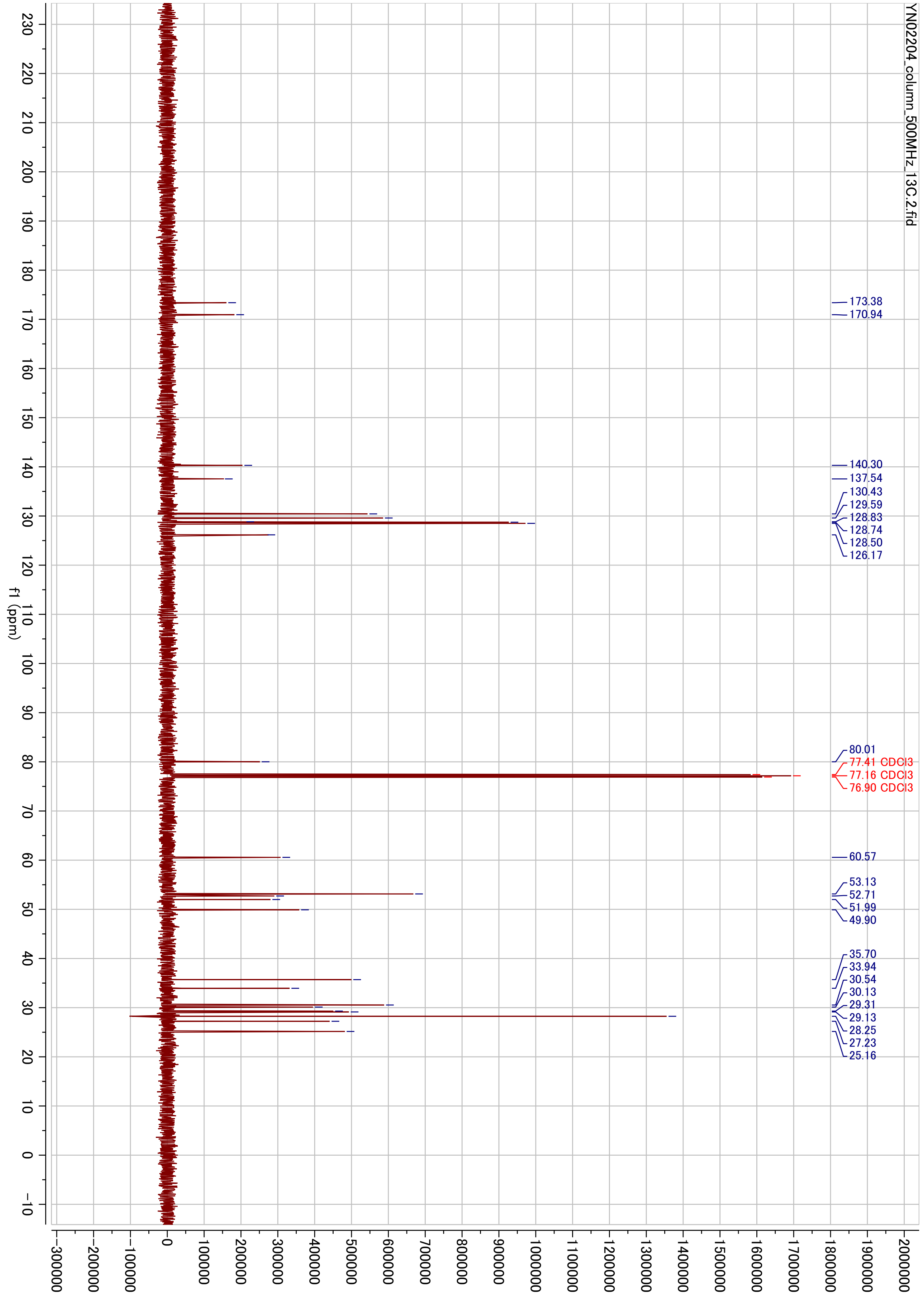
YN02204_column_500MHz_13C.2.fid
173.38
170.94
140.30
137.54
130.43
129.59
128.83
128.74
128.50
126.17
80.01
77.41 CDCl3
77.16 CDCl3
76.90 CDCl3
60.57
53.13
52.71
51.99
49.90
35.70
33.94
30.54
30.13
29.31
29.13
28.25
27.23
25.16
f1 (ppm)

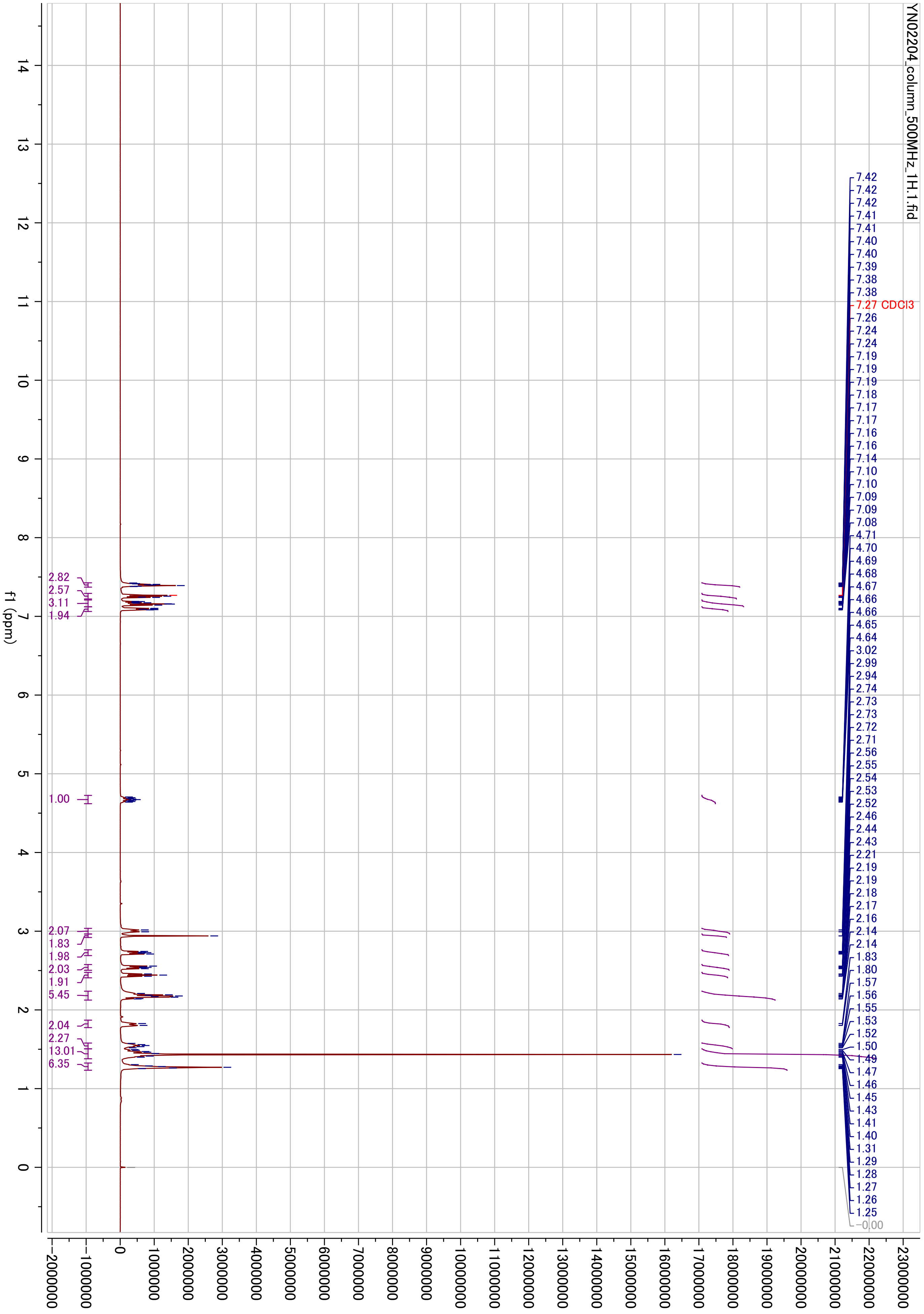

YN02204_column_500MHz_1H.1.fid
7.42
7.42
7.42
7.41
7.41
7.40
7.40
7.39
7.38
7.38
7.27 CDCl3
7.26
7.24
7.24
7.19
7.19
7.19
7.18
7.17
7.17
7.16
7.16
7.14
7.10
7.10
7.09
7.09
7.08
4.71
4.70
4.69
4.68
4.67
4.66
4.66
4.65
4.64
3.02
2.99
2.94
2.74
2.73
2.73
2.72
2.71
2.56
2.55
2.54
2.53
2.52
2.46
2.44
2.43
2.21
2.19
2.19
2.18
2.17
2.16
2.14
2.14
1.83
1.80
1.57
1.56
1.55
1.53
1.52
1.50
1.49
1.47
1.46
1.45
1.43
1.41
1.40
1.31
1.29
1.28
1.27
1.26
1.25
0.00
2.82
2.57
3.11
1.94
1.00
2.07
1.83
1.98
2.03
1.91
5.45
2.04
2.27
13.01
6.35
f1 (ppm)

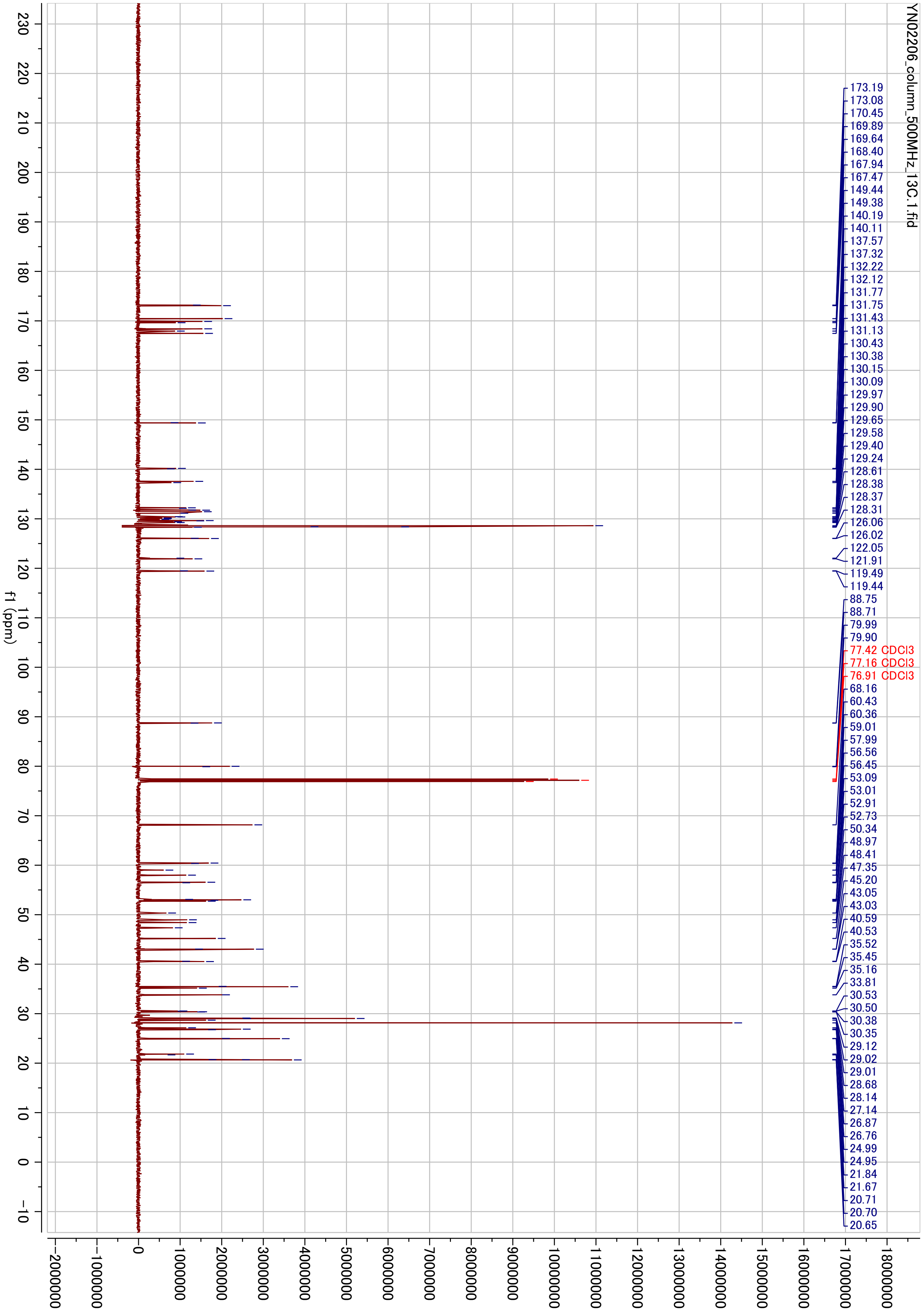
YN02206_column_500MHz_13C.1.fid
173.19
173.08
170.45
169.89
169.64
168.40
167.94
167.47
149.44
149.38
140.19
140.11
137.57
137.32
132.22
132.12
131.77
131.75
131.43
131.13
130.43
130.38
130.15
130.09
129.97
129.90
129.65
129.58
129.40
129.24
128.61
128.38
128.37
128.31
126.06
126.02
122.05
121.91
119.49
119.44
88.75
88.71
79.99
79.90
77.42 CDCl3
77.16 CDCl3
76.91 CDCl3
68.16
60.43
60.36
59.01
57.99
56.56
56.45
53.09
53.01
52.91
52.73
50.34
48.97
48.41
47.35
45.20
43.05
43.03
40.59
40.53
35.52
35.45
35.16
33.81
30.53
30.50
30.38
30.35
29.12
29.02
29.01
28.68
28.14
27.14
26.87
26.76
24.99
24.95
21.84
21.67
20.71
20.70
20.65
f1 (ppm)

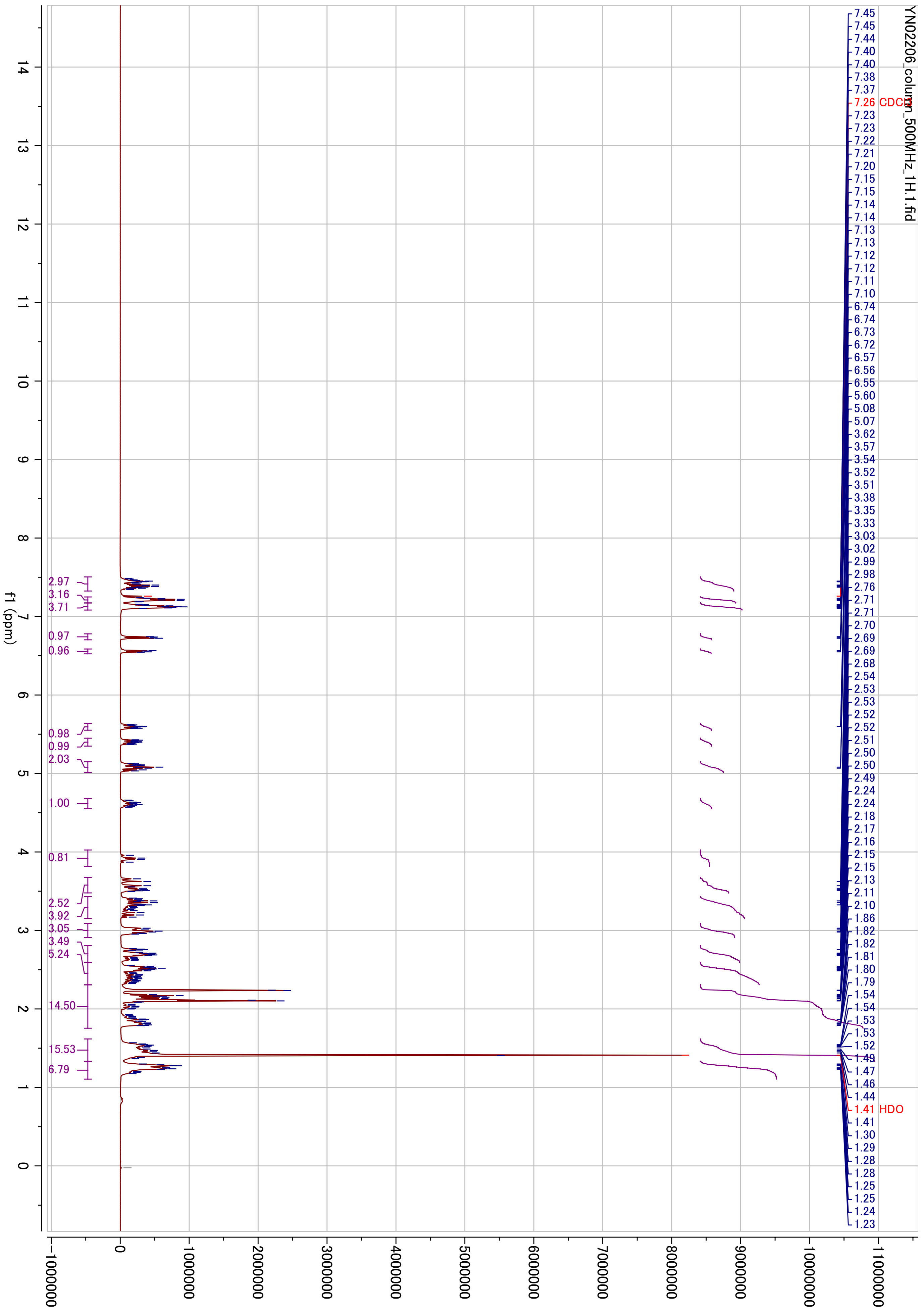
YN02206_column_500MHz_1H.1.fid
7.45
7.45
7.44
7.40
7.40
7.38
7.37
7.26 CDCl3
7.23
7.23
7.22
7.21
7.20
7.15
7.15
7.14
7.14
7.13
7.13
7.12
7.12
7.11
7.10
6.74
6.74
6.73
6.72
6.57
6.56
6.55
5.60
5.08
5.07
3.62
3.57
3.54
3.52
3.51
3.38
3.35
3.33
3.03
3.02
2.99
2.98
2.76
2.71
2.71
2.70
2.69
2.69
2.68
2.54
2.53
2.53
2.52
2.52
2.51
2.50
2.50
2.49
2.24
2.24
2.18
2.17
2.16
2.15
2.15
2.13
2.11
2.10
1.86
1.82
1.82
1.81
1.80
1.79
1.54
1.54
1.53
1.53
1.52
1.49
1.47
1.46
1.44
1.41 HDO
1.41
1.30
1.29
1.28
1.28
1.25
1.25
1.24
1.23
2.97
3.16
3.71
0.97
0.96
0.98
0.99
2.03
1.00
0.81
2.52
3.92
3.05
3.49
5.24
14.50
15.53
6.79
f1 (ppm)

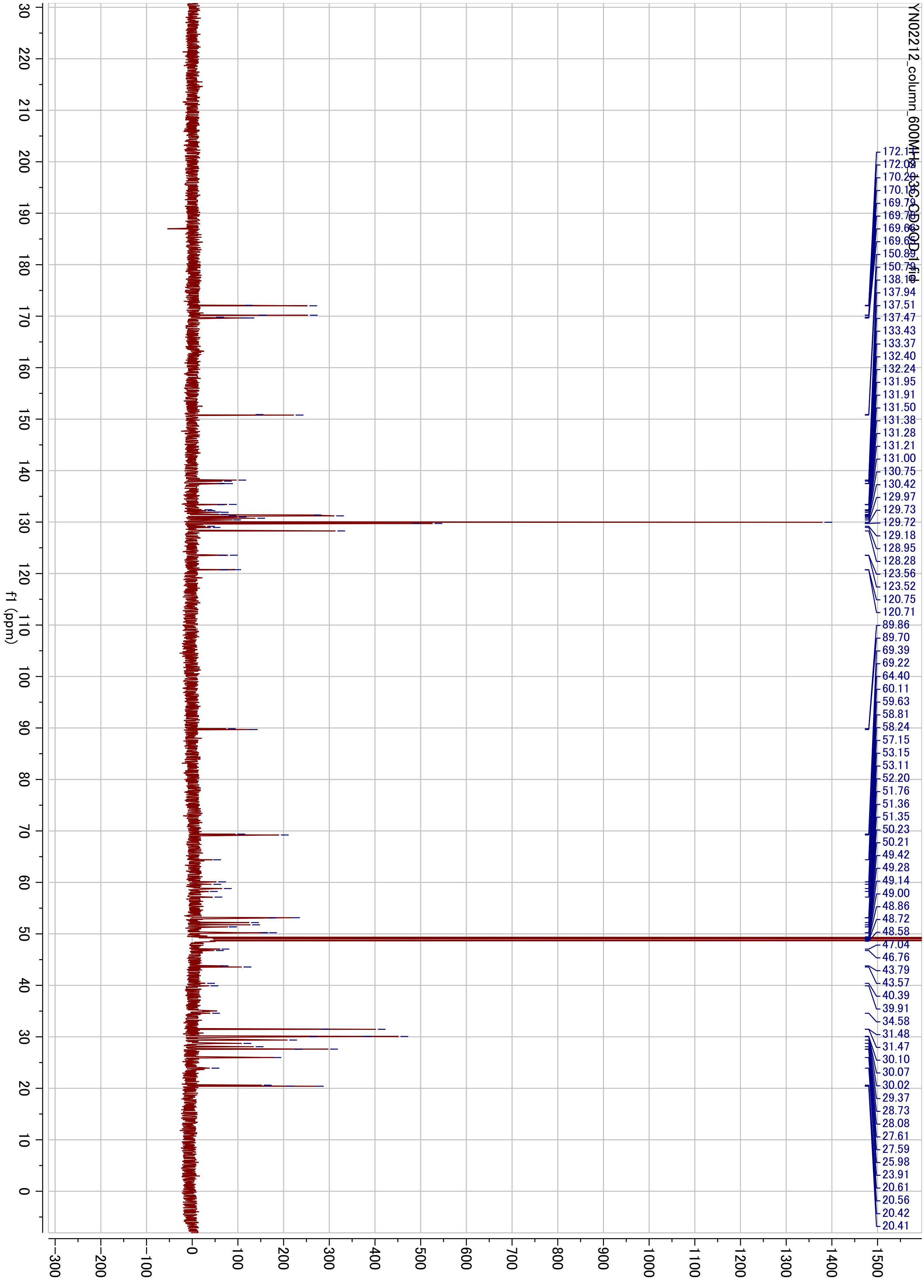
f1 (ppm)
137.94
137.51
137.47
133.43
133.37
132.40
132.24
131.95
131.91
131.50
131.38
131.28
131.21
131.00
130.75
130.42
129.97
129.73
129.72
129.18
128.95
128.28
123.56
123.52
120.75
120.71
89.86
89.70
69.39
69.22
64.40
60.11
59.63
58.81
58.24
57.15
53.15
53.11
52.20
51.76
51.36
51.35
50.23
50.21
49.42
49.28
49.14
49.00
48.86
48.72
48.58
47.04
46.76
43.79
43.57
40.39
39.91
34.58
31.48
31.47
30.10
30.07
30.02
29.37
28.73
28.08
27.61
27.59
25.98
23.91
20.61
20.56
20.42
20.41

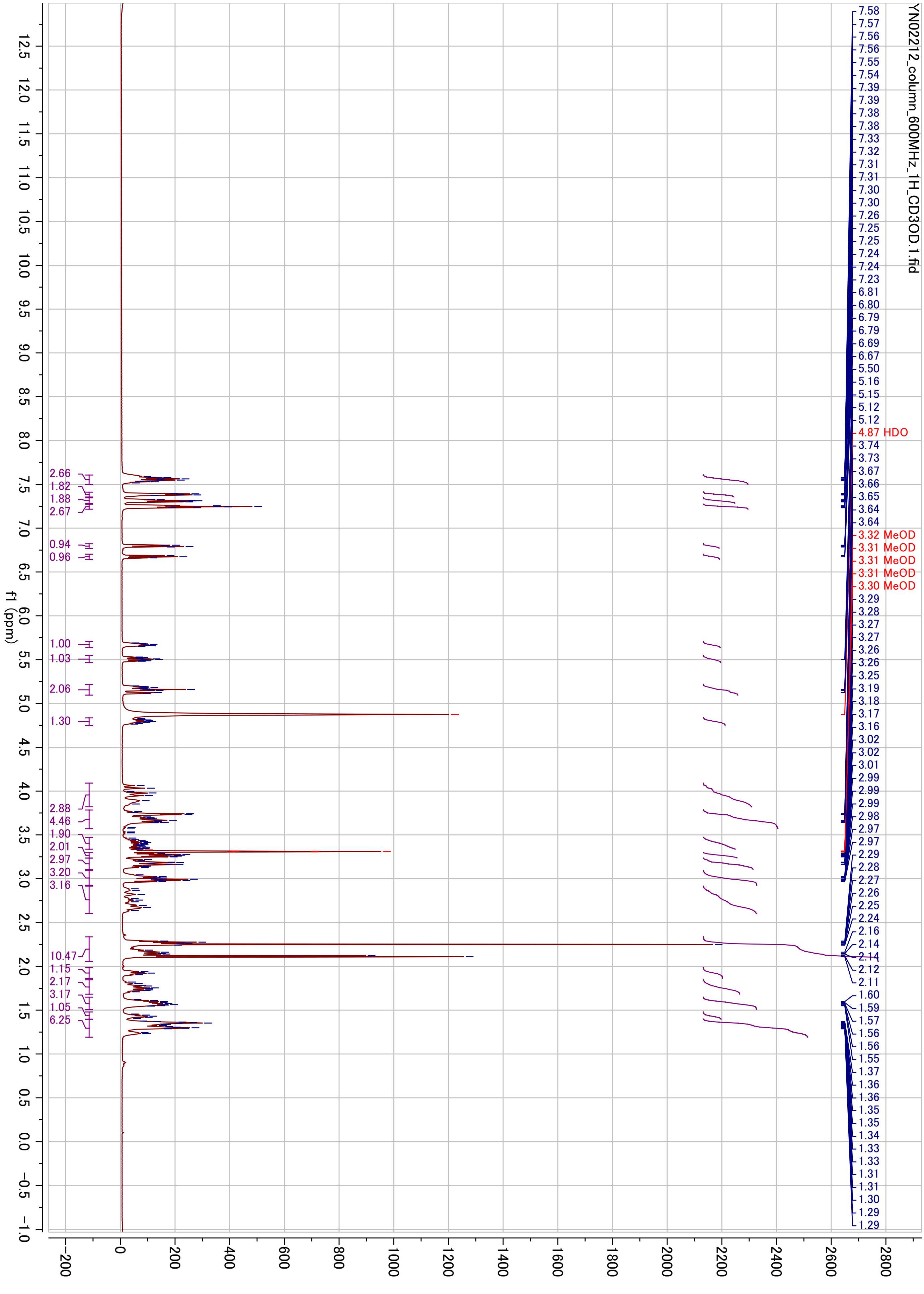
YN02212_column_600MHz_1H_CD3OD.1.fid
7.58
7.57
7.56
7.56
7.55
7.54
7.39
7.39
7.38
7.38
7.33
7.32
7.31
7.31
7.30
7.30
7.26
7.25
7.25
7.24
7.24
7.23
6.81
6.80
6.79
6.79
6.69
6.67
5.50
5.16
5.15
5.12
5.12
4.87 HDO
3.74
3.73
3.67
3.66
3.65
3.64
3.64
3.32 MeOD
3.31 MeOD
3.31 MeOD
3.31 MeOD
3.30 MeOD
3.29
3.28
3.27
3.27
3.26
3.26
3.25
3.19
3.18
3.17
3.16
3.02
3.02
3.01
2.99
2.99
2.99
2.98
2.97
2.97
2.29
2.28
2.27
2.26
2.25
2.24
2.16
2.14
2.14
2.12
2.11
1.60
1.59
1.57
1.56
1.56
1.55
1.37
1.36
1.36
1.35
1.35
1.34
1.33
1.33
1.31
1.31
1.30
1.29
1.29
2.66
1.82
1.88
2.67
0.94
0.96
1.00
1.03
2.06
1.30
2.88
4.46
1.90
2.01
2.97
3.20
3.16
10.47
1.15
2.17
3.17
1.05
6.25
f1 (ppm)

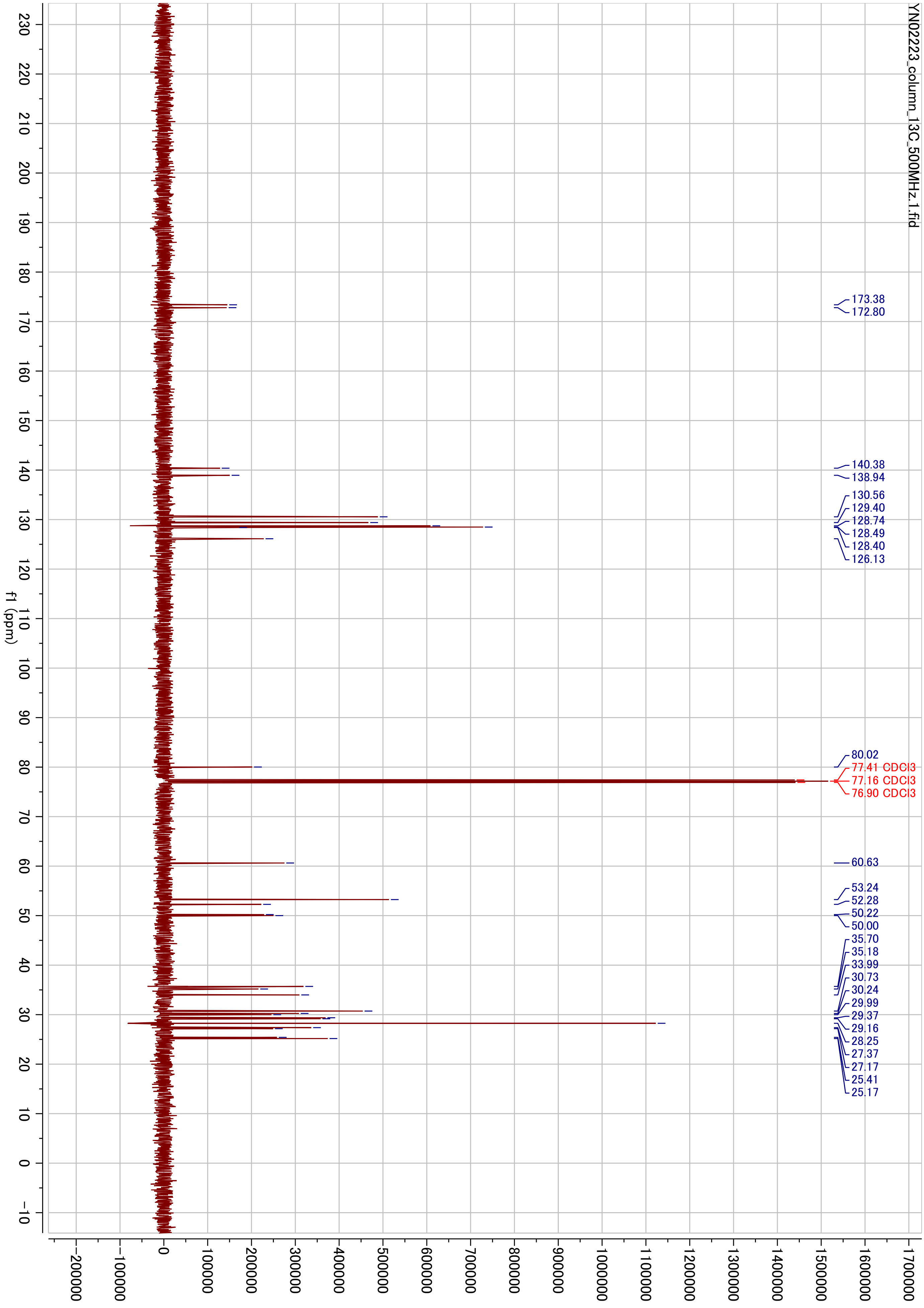
YN02223_column_13C_500MHz.1.fid
173.38
172.80
140.38
138.94
130.56
129.40
128.74
128.49
128.40
126.13
80.02
77.41 CDCl3
77.16 CDCl3
76.90 CDCl3
60.63
53.24
52.28
50.22
50.00
35.70
35.18
33.99
30.73
30.24
29.99
29.37
29.16
28.25
27.37
27.17
25.41
25.17
f1 (ppm)

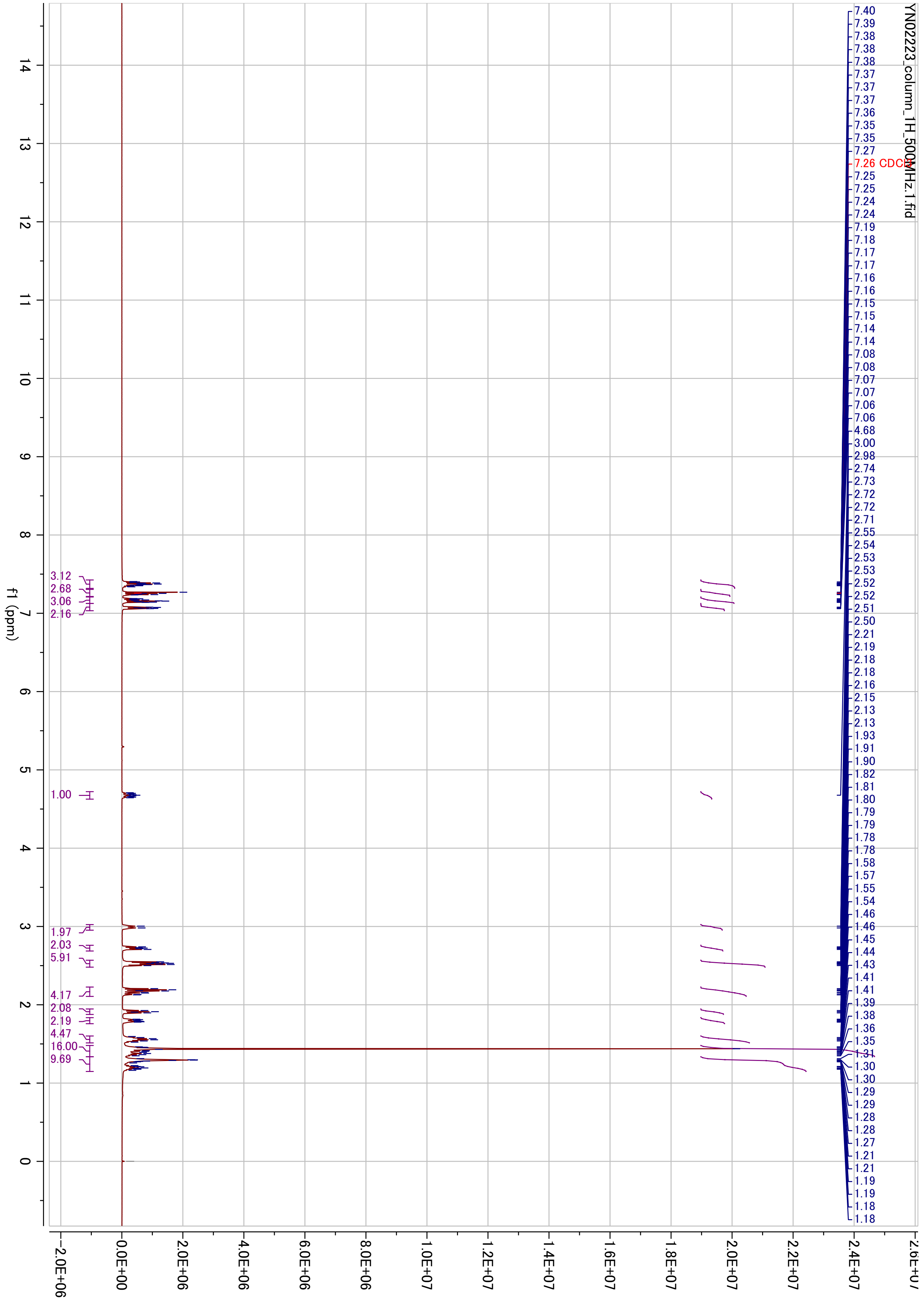
YN02223_column_1H_500MHz.1.fid
7.40 7.39 7.38 7.38 7.38 7.37 7.37 7.37 7.36 7.35 7.35 7.27
7.26 CDCl3
7.25 7.25 7.24 7.24 7.19 7.18 7.17 7.17 7.16 7.16 7.15 7.15 7.14 7.14 7.08 7.08 7.07 7.07 7.06 7.06 4.68 3.00 2.98 2.74 2.73 2.72 2.72 2.71 2.55 2.54 2.53 2.53 2.52 2.52 2.51 2.50 2.21 2.19 2.18 2.18 2.16 2.15 2.13 2.13 1.93 1.91 1.90 1.82 1.81 1.80 1.79 1.79 1.78 1.78 1.58 1.57 1.55 1.54 1.46 1.46 1.45 1.44 1.43 1.41 1.41 1.39 1.38 1.36 1.35 1.31 1.30 1.30 1.29 1.29 1.28 1.28 1.27 1.21 1.21 1.19 1.19 1.18 1.18
3.12 2.68 3.06 2.16 1.00 1.97 2.03 5.91 4.17 2.08 2.19 4.47 16.00 9.69
f1 (ppm)

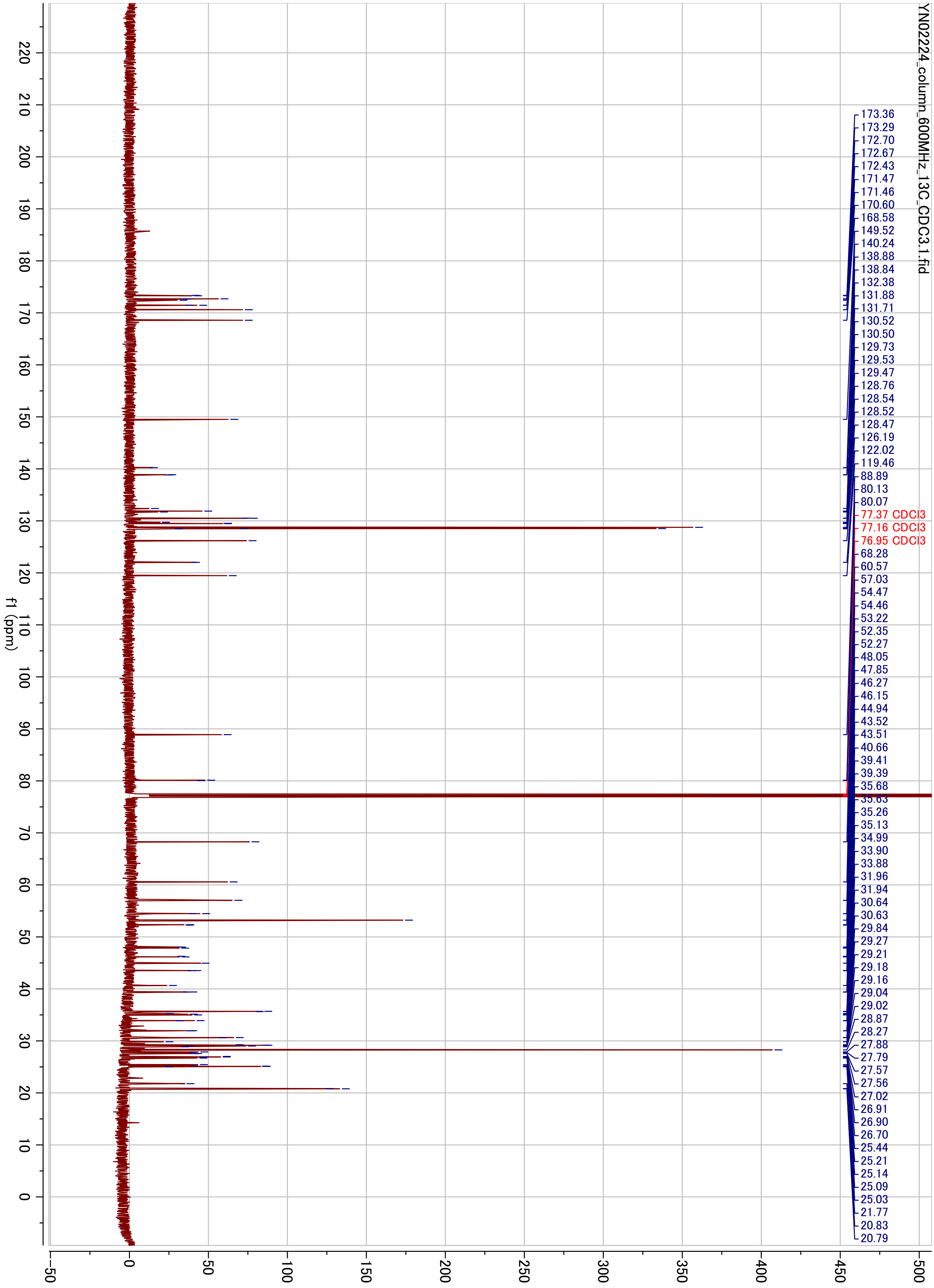
YN02224_column_600MHz_13C_CDC3.1.fid
173.36
173.29
172.70
172.67
172.43
171.47
171.46
170.60
168.58
149.52
140.24
138.88
138.84
132.38
131.88
131.71
130.52
130.50
129.73
129.53
129.47
128.76
128.54
128.52
128.47
126.19
122.02
119.46
88.89
80.13
80.07
77.37 CDCl3
77.16 CDCl3
76.95 CDCl3
68.28
60.57
57.03
54.47
54.46
53.22
52.35
52.27
48.05
47.85
46.27
46.15
44.94
43.52
43.51
40.66
39.41
39.39
35.68
35.63
35.26
35.13
34.99
33.90
33.88
31.96
31.94
30.64
30.63
29.84
29.27
29.21
29.18
29.16
29.04
29.02
28.87
28.27
27.88
27.79
27.57
27.56
27.02
26.91
26.90
26.70
25.44
25.21
25.14
25.09
25.03
21.77
20.83
20.79
f1 (ppm)

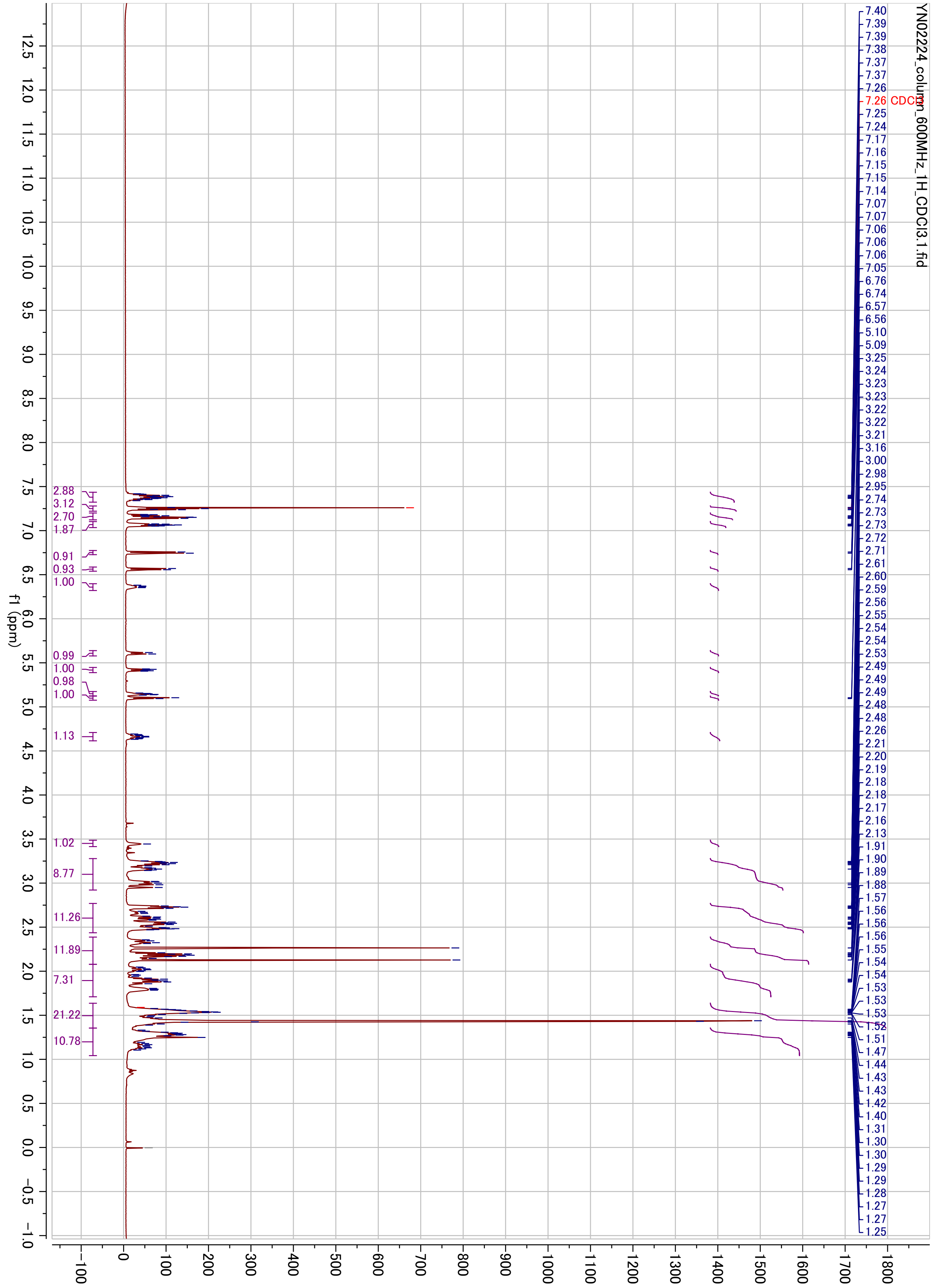
YN02224_column_600MHz_1H_CDCl3.1.fid
7.40
7.39
7.39
7.38
7.37
7.37
7.26
7.26 CDCl3
7.25
7.24
7.17
7.16
7.15
7.15
7.14
7.07
7.07
7.06
7.06
7.06
7.05
6.76
6.74
6.57
6.56
5.10
5.09
3.25
3.24
3.23
3.23
3.22
3.22
3.21
3.16
3.00
2.98
2.95
2.74
2.73
2.73
2.72
2.71
2.61
2.60
2.59
2.56
2.55
2.54
2.54
2.53
2.49
2.49
2.49
2.48
2.48
2.26
2.21
2.20
2.19
2.18
2.18
2.17
2.16
2.13
1.91
1.90
1.89
1.88
1.57
1.56
1.56
1.56
1.55
1.54
1.54
1.53
1.53
1.53
1.52
1.51
1.47
1.44
1.43
1.43
1.42
1.40
1.31
1.30
1.30
1.29
1.29
1.28
1.27
1.27
1.25
2.88
3.12
2.70
1.87
0.91
0.93
1.00
0.99
1.00
0.98
1.00
1.13
1.02
8.77
11.26
11.89
7.31
21.22
10.78
f1 (ppm)

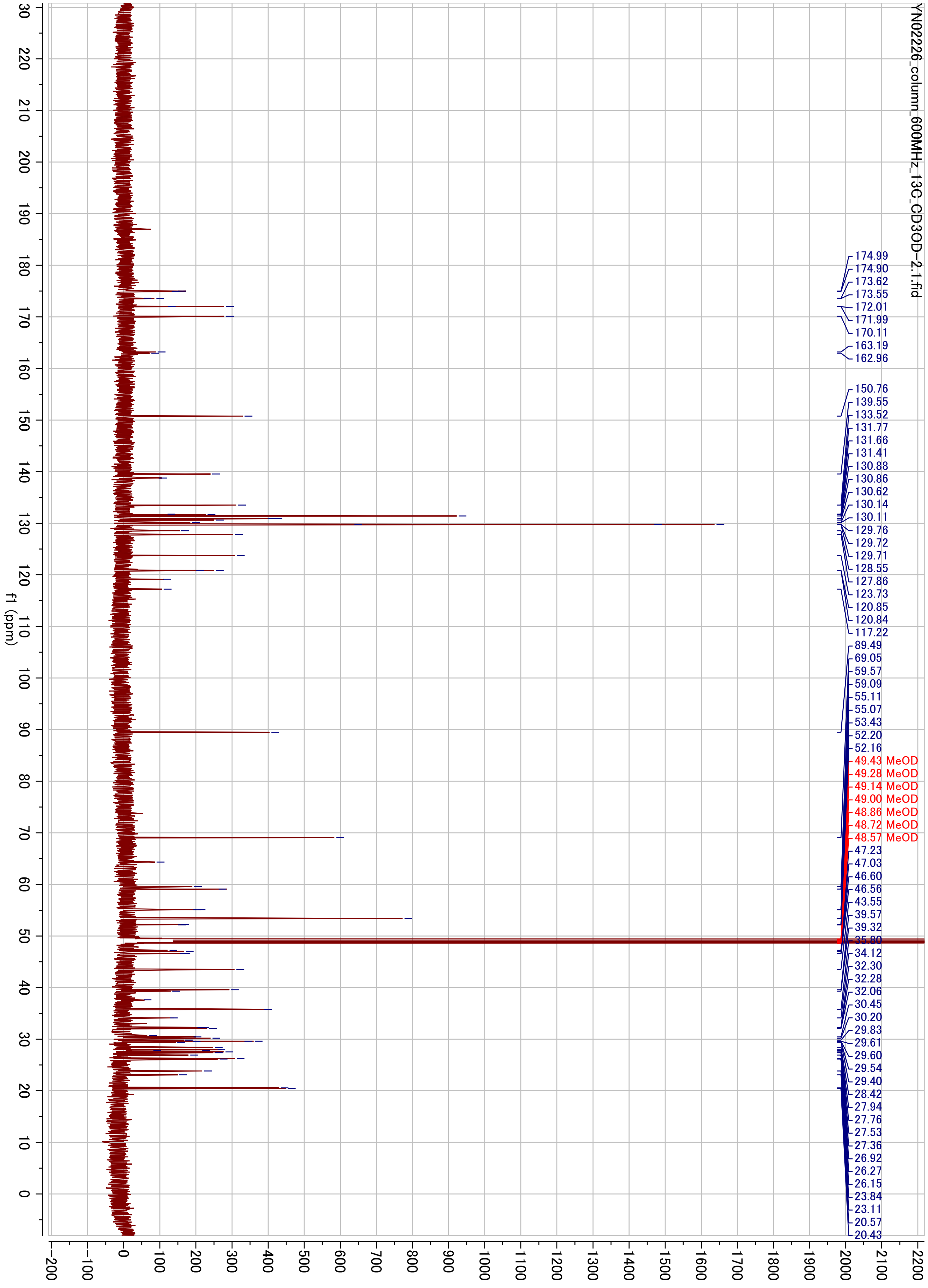
YN02226_column_600MHz_13C_CD3OD-2.1.fid
174.99
174.90
173.62
173.55
172.01
171.99
170.11
163.19
162.96
150.76
139.55
133.52
131.77
131.66
131.41
130.88
130.86
130.62
130.14
130.11
129.76
129.72
129.71
128.55
127.86
123.73
120.85
120.84
117.22
89.49
69.05
59.57
59.09
55.11
55.07
53.43
52.20
52.16
49.43 MeOD
49.28 MeOD
49.14 MeOD
49.00 MeOD
48.86 MeOD
48.72 MeOD
48.57 MeOD
47.23
47.03
46.60
46.56
43.55
39.57
39.32
35.80
34.12
32.30
32.28
32.06
30.45
30.20
29.83
29.61
29.60
29.54
29.40
28.42
27.94
27.76
27.53
27.36
26.92
26.27
26.15
23.84
23.11
20.57
20.43
f1 (ppm)

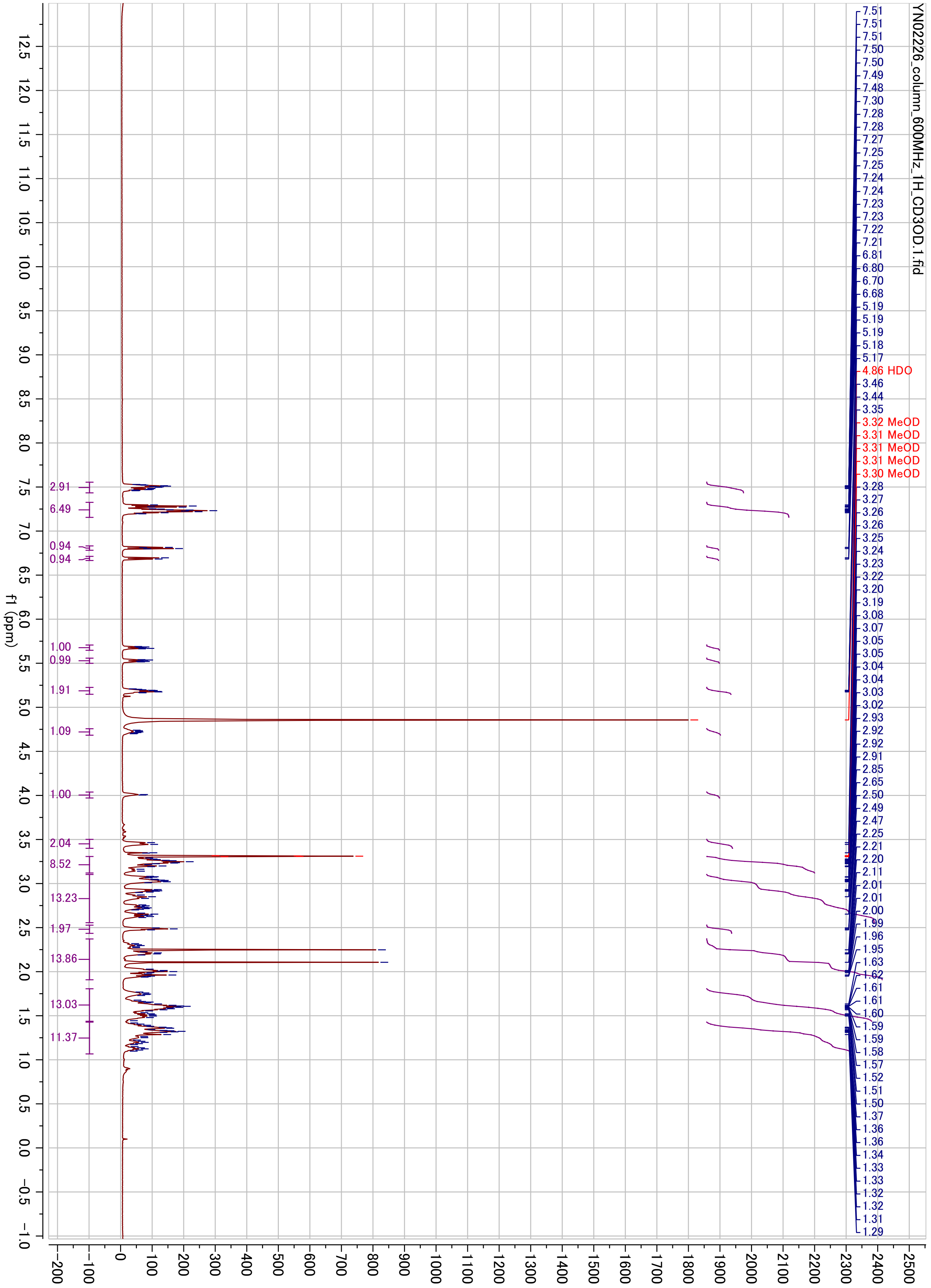
YN02226_column_600MHz_1H_CD3OD.1.fid
f1 (ppm)
4.86 HDO
3.32 MeOD
3.31 MeOD
3.31 MeOD
3.31 MeOD
3.30 MeOD

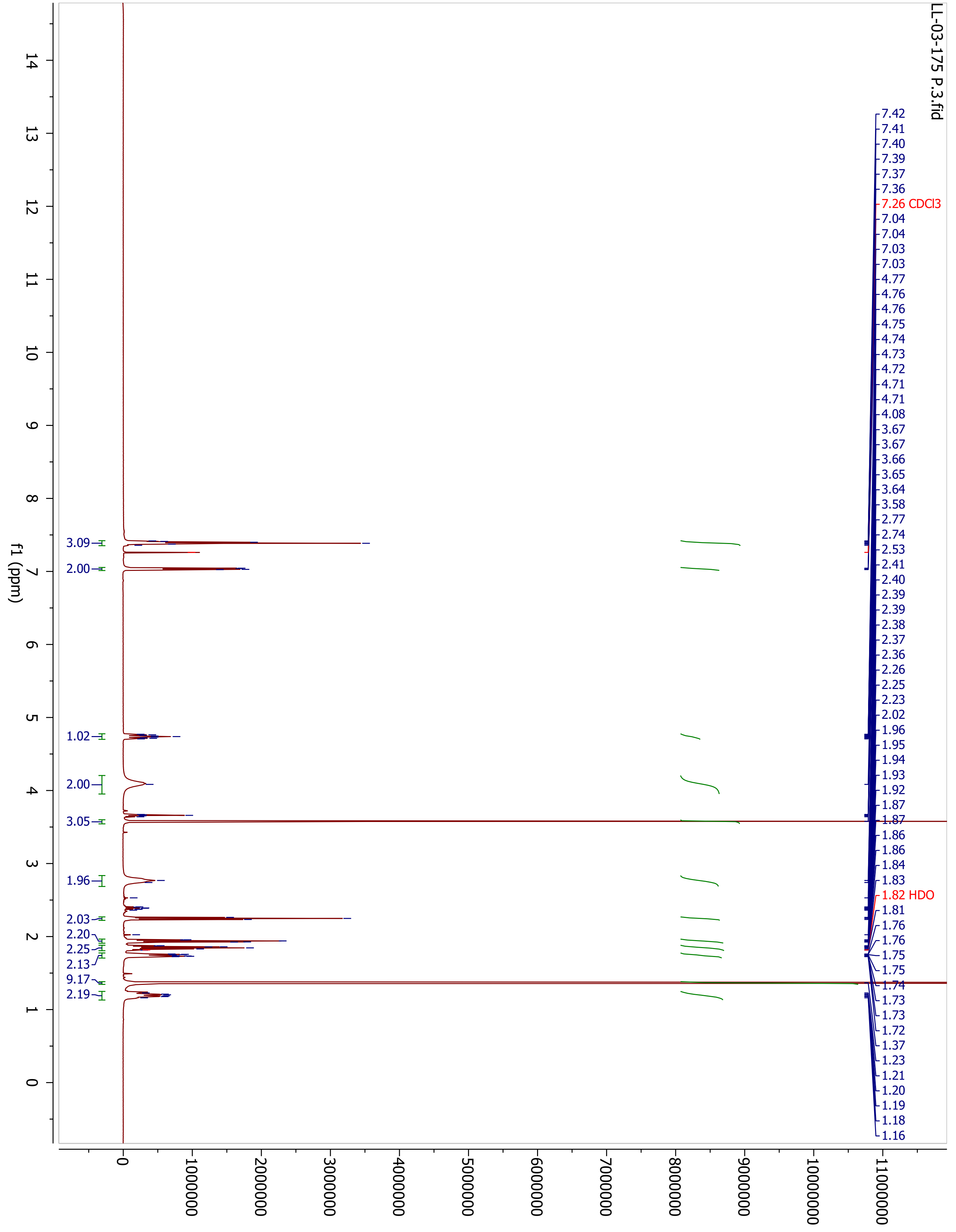

LL-03-175 P.3.fid
7.42
7.41
7.40
7.39
7.37
7.36
7.26 CDCl3
7.04
7.04
7.03
7.03
4.77
4.76
4.76
4.75
4.74
4.73
4.72
4.71
4.71
4.08
3.67
3.67
3.66
3.65
3.64
3.58
2.77
2.74
2.53
2.41
2.40
2.39
2.39
2.38
2.37
2.36
2.26
2.25
2.23
2.02
1.96
1.95
1.94
1.93
1.92
1.87
1.87
1.86
1.86
1.84
1.83
1.82 HDO
1.81
1.76
1.76
1.75
1.75
1.74
1.73
1.73
1.72
1.37
1.23
1.21
1.20
1.19
1.18
1.16
3.09
2.00
1.02
2.00
3.05
1.96
2.03
2.20
2.25
2.13
9.17
2.19
f1 (ppm)

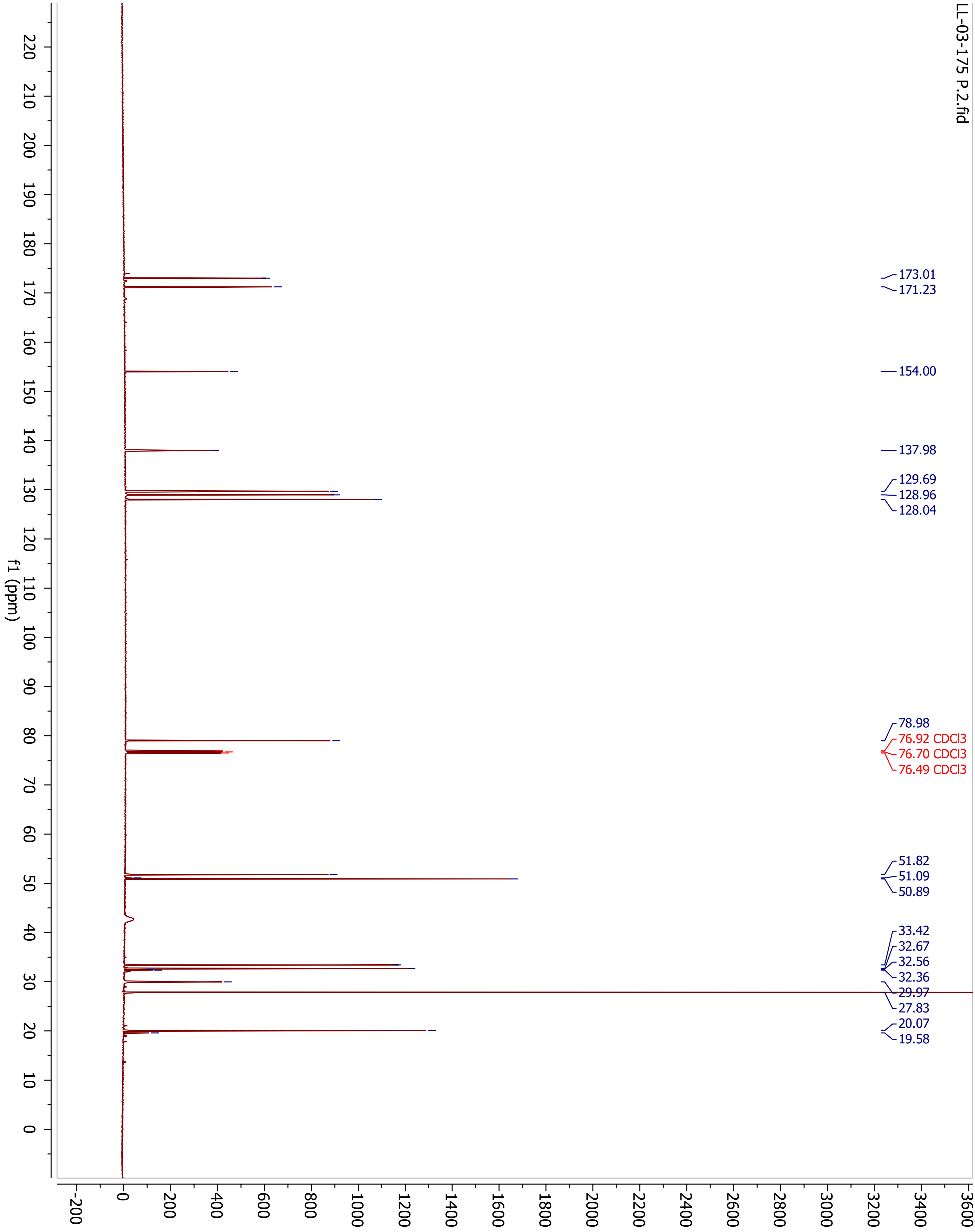
LL-03-175 P.2.fid
173.01
171.23
154.00
137.98
129.69
128.96
128.04
78.98
76.92 CDCl3
76.70 CDCl3
76.49 CDCl3
51.82
51.09
50.89
33.42
32.67
32.56
32.36
29.97
27.83
20.07
19.58
f1 (ppm)

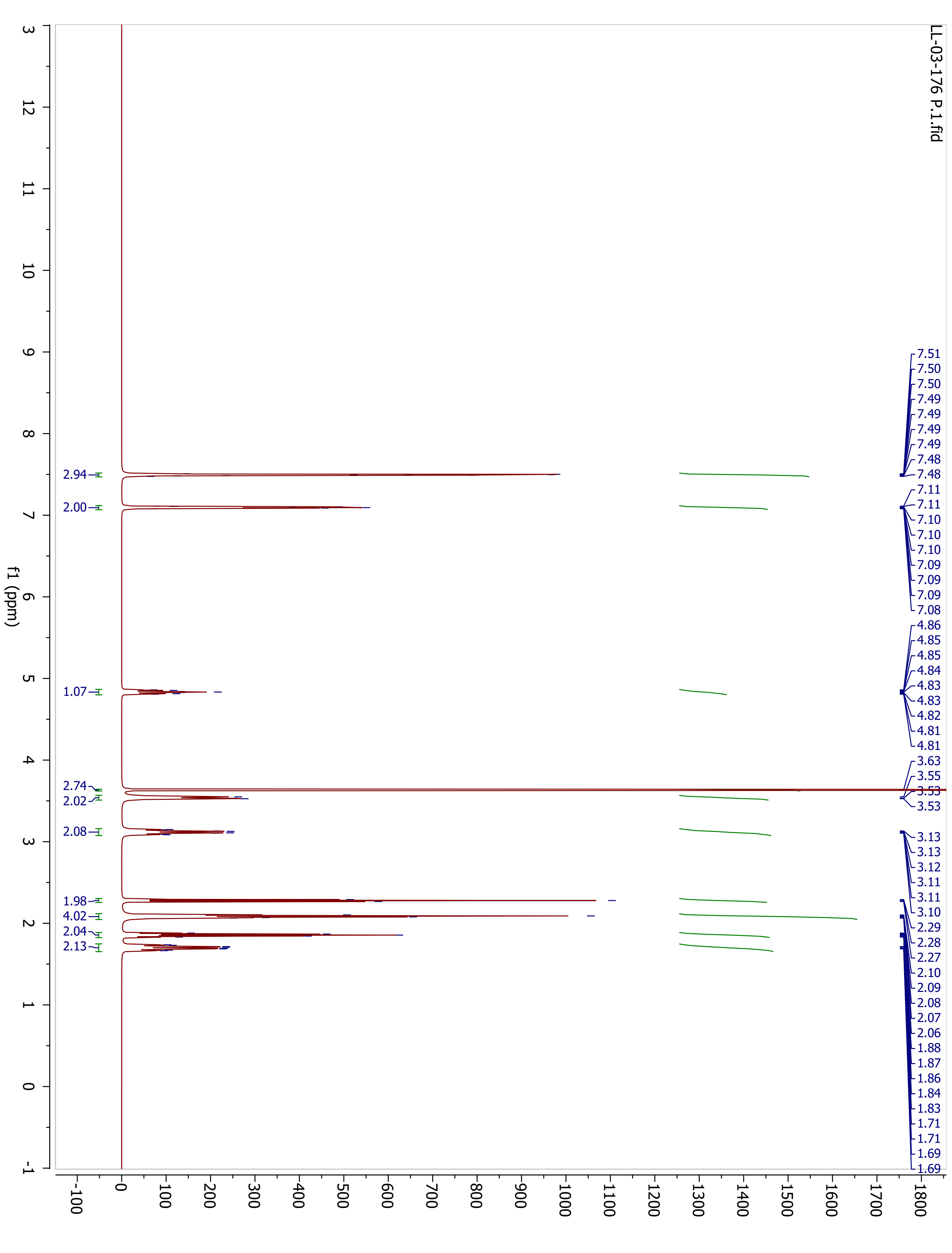
LL-03-176 P.1.fid
7.51
7.50
7.50
7.49
7.49
7.49
7.49
7.48
7.48
7.11
7.11
7.10
7.10
7.10
7.09
7.09
7.09
7.08
4.86
4.85
4.85
4.84
4.83
4.83
4.82
4.81
4.81
3.63
3.55
3.53
3.53
3.13
3.13
3.12
3.11
3.11
3.10
2.29
2.28
2.27
2.10
2.09
2.08
2.07
2.06
1.88
1.87
1.86
1.84
1.83
1.71
1.71
1.69
1.69
2.94
2.00
1.07
2.74
2.02
2.08
1.98
4.02
2.04
2.13
f1 (ppm)

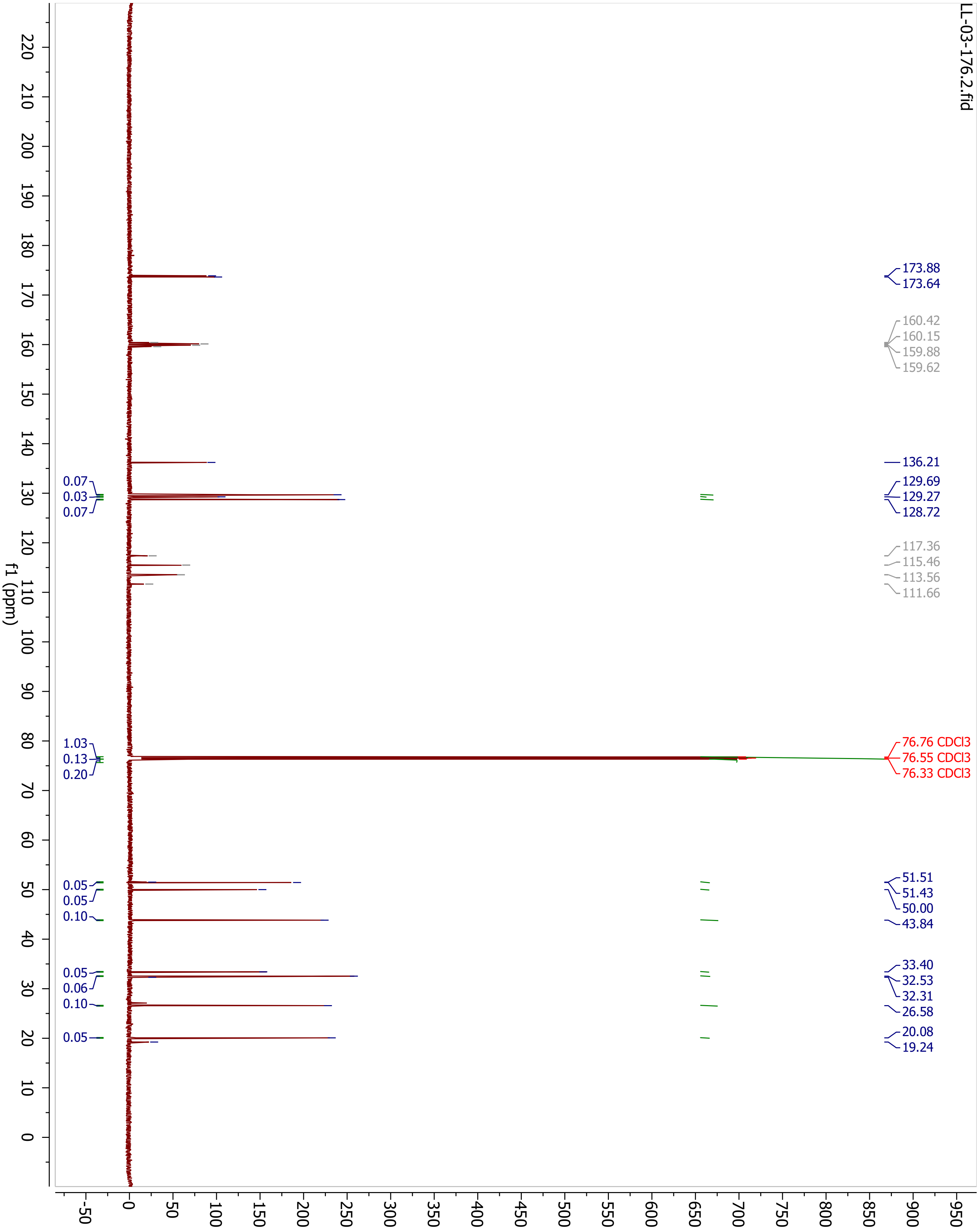
LL-03-176.2.fid
173.88
173.64
160.42
160.15
159.88
159.62
136.21
129.69
129.27
128.72
117.36
115.46
113.56
111.66
76.76 CDCl3
76.55 CDCl3
76.33 CDCl3
51.51
51.43
50.00
43.84
33.40
32.53
32.31
26.58
20.08
19.24
0.07
0.03
0.07
1.03
0.13
0.20
0.05
0.05
0.10
0.05
0.06
0.10
0.05
f1 (ppm)

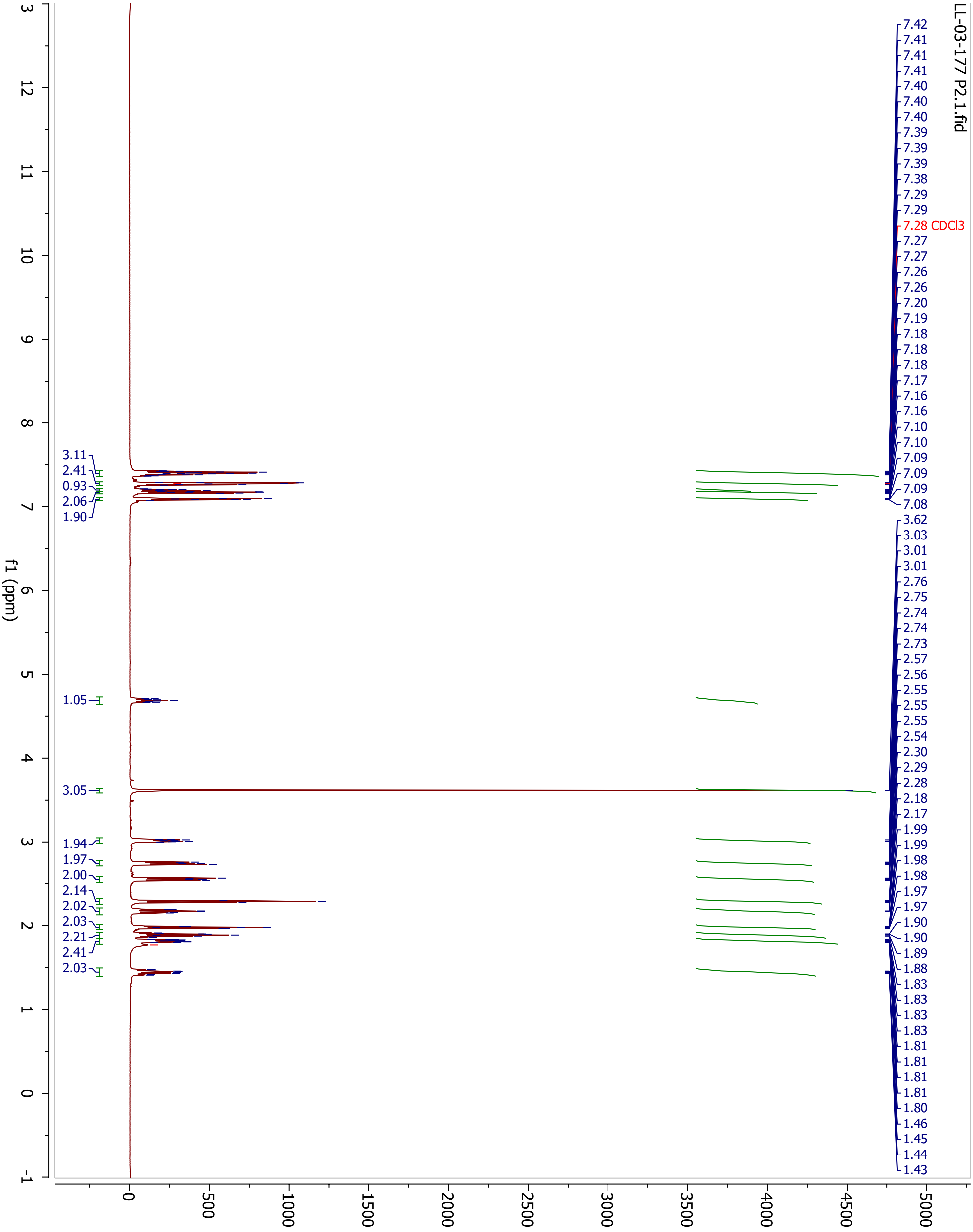

LL-03-177 P2.1.fid
7.42
7.41
7.41
7.41
7.40
7.40
7.40
7.39
7.39
7.39
7.38
7.29
7.29
7.28 CDCl3
7.27
7.27
7.26
7.26
7.20
7.19
7.18
7.18
7.18
7.17
7.16
7.16
7.10
7.10
7.09
7.09
7.09
7.08
3.62
3.03
3.01
3.01
2.76
2.75
2.74
2.74
2.73
2.57
2.56
2.55
2.55
2.55
2.54
2.30
2.29
2.28
2.18
2.17
1.99
1.99
1.98
1.98
1.97
1.97
1.90
1.90
1.89
1.88
1.83
1.83
1.83
1.83
1.81
1.81
1.81
1.81
1.80
1.46
1.45
1.44
1.43
3.11
2.41
0.93
2.06
1.90
1.05
3.05
1.94
1.97
2.00
2.14
2.02
2.03
2.21
2.41
2.03
f1 (ppm)

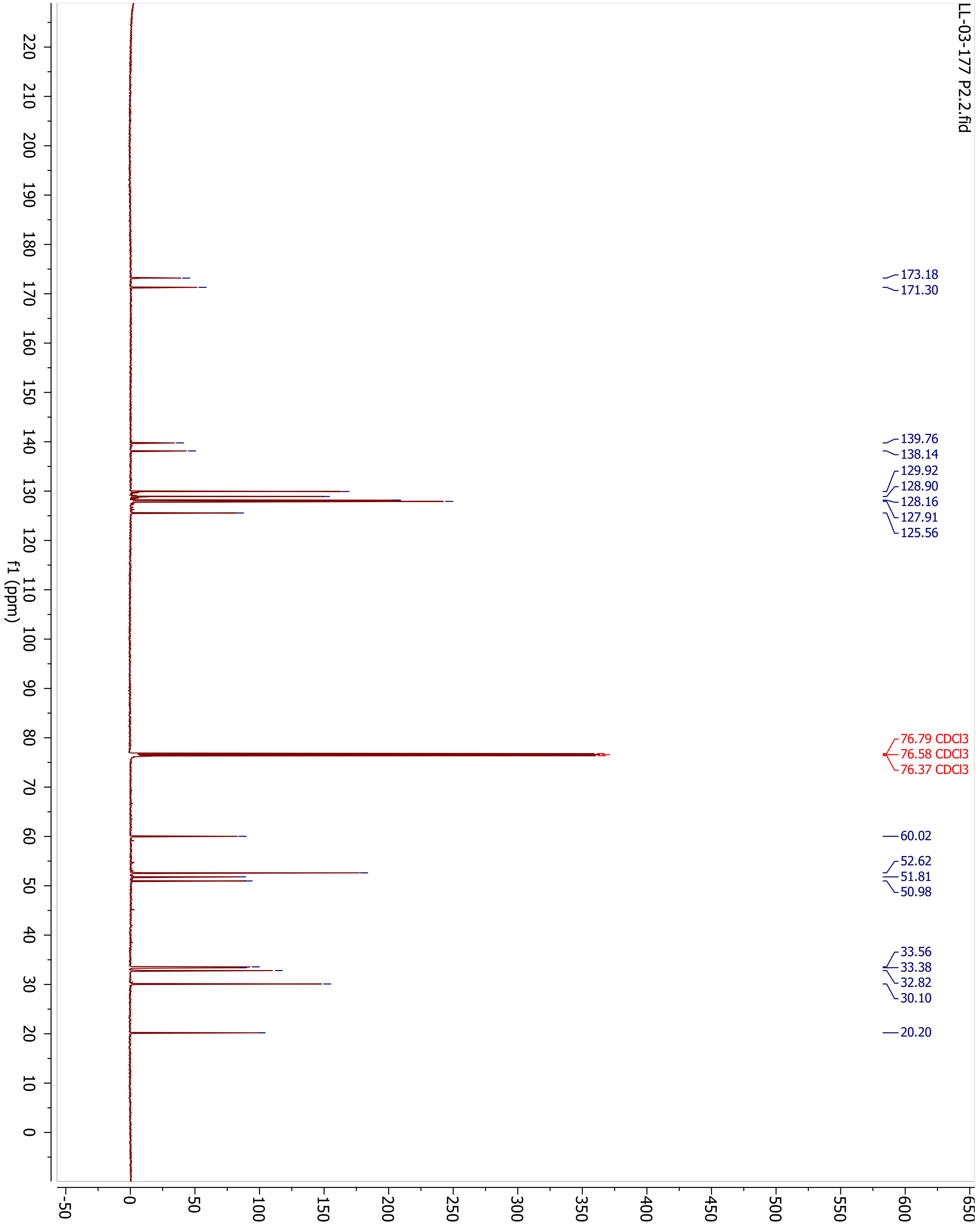
LL-03-177 P2.2.fid
173.18
171.30
139.76
138.14
129.92
128.90
128.16
127.91
125.56
76.79 CDCl3
76.58 CDCl3
76.37 CDCl3
60.02
52.62
51.81
50.98
33.56
33.38
32.82
30.10
20.20
f1 (ppm)

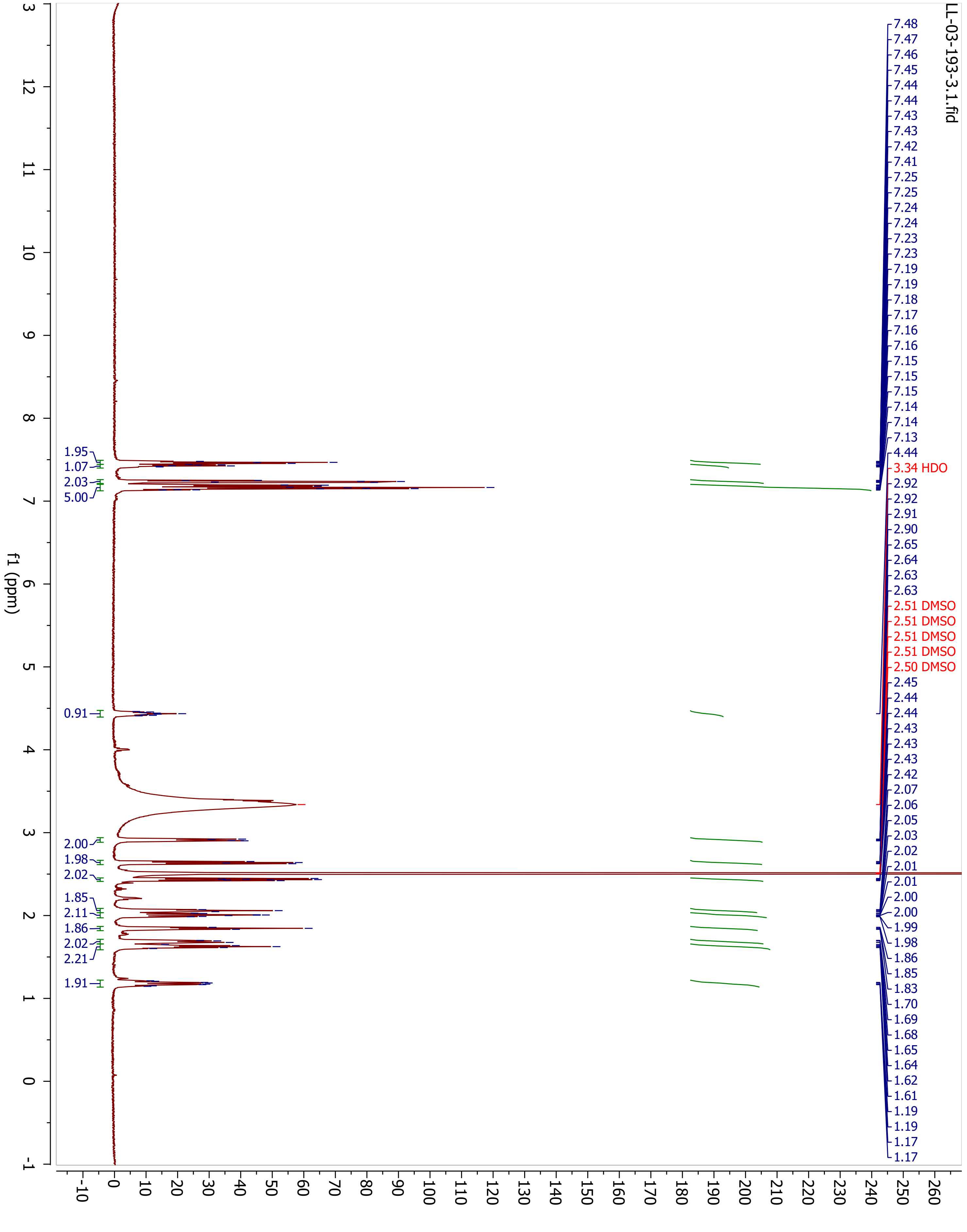
LL-03-193-3.1.fid
7.48
7.47
7.46
7.45
7.44
7.44
7.43
7.43
7.42
7.41
7.25
7.25
7.24
7.24
7.23
7.23
7.19
7.19
7.18
7.17
7.16
7.16
7.15
7.15
7.15
7.14
7.14
7.13
4.44
3.34 HDO
2.92
2.92
2.91
2.90
2.65
2.64
2.63
2.63
2.51 DMSO
2.51 DMSO
2.51 DMSO
2.51 DMSO
2.50 DMSO
2.45
2.44
2.44
2.43
2.43
2.42
2.07
2.06
2.05
2.03
2.02
2.01
2.01
2.00
2.00
1.99
1.98
1.86
1.85
1.83
1.70
1.69
1.68
1.65
1.64
1.62
1.61
1.19
1.19
1.17
1.17
1.95
1.07
2.03
5.00
0.91
2.00
1.98
2.02
1.85
2.11
1.86
2.02
2.21
1.91
f1 (ppm)

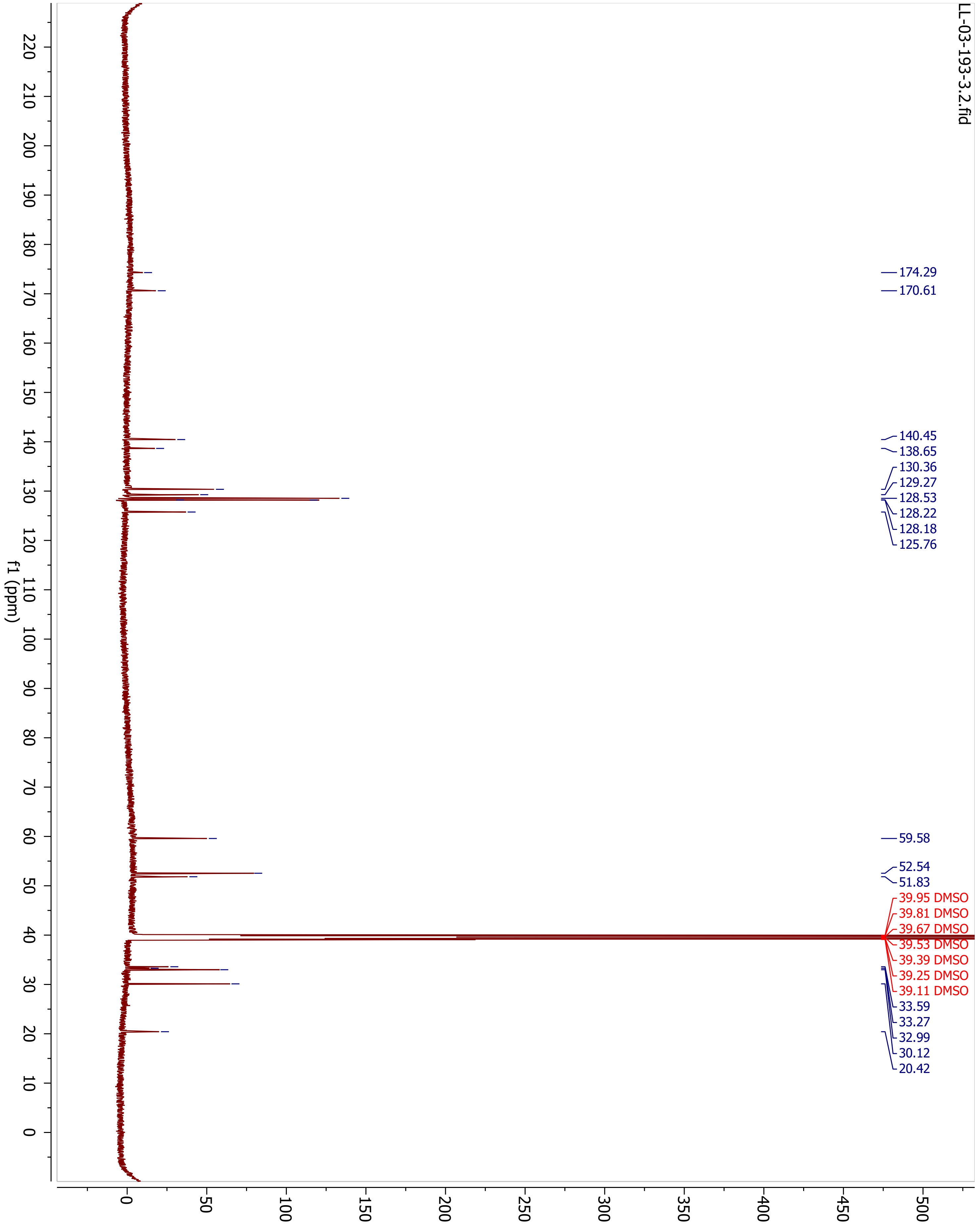
LL-03-193-3.2.fid
174.29
170.61
140.45
138.65
130.36
129.27
128.53
128.22
128.18
125.76
59.58
52.54
51.83
39.95 DMSO
39.81 DMSO
39.67 DMSO
39.53 DMSO
39.39 DMSO
39.25 DMSO
39.11 DMSO
33.59
33.27
32.99
30.12
20.42
f1 (ppm)
